# Supplementary material for: Defluorosilylation of fluoroarenes and fluoroalkanes
Source: Nat Commun. 2018 Oct 22;9:4393. doi: 10.1038/s41467-018-06830-w (PMC6197272; doi:10.1038/s41467-018-06830-w)
Supplement: Supplementary file 1 — Supplementary Information [file 41467_2018_6830_MOESM1_ESM.pdf]

# Supplementary Information

# Defluorosilylation of Fluoroarenes and Fluoroalkanes

Benqiang Cui,[1] Shichong Jia,[1] Etsuko Tokunaga,[1] and Norio Shibata [1,2*]

[1]Department of Nanopharmaceutical Sciences and Department of Life Science and Applied Chemistry, Nagoya Institute of Technology, Gokiso, Showa-ku, Nagoya 466-8555, Japan.
[2]Institute of Advanced Fluorine-Containing Materials, Zhejiang Normal University, 688 Yingbin Avenue, 321004 Jinhua, China

Correspondence to: nozshiba@nitech.ac.jp

**This PDF file includes:**

Supplementary Methods 1
Supplementary Table 1-7
Supplementary Figure 1-93
Supplementary References

## Supplementary Methods

### General information

All reactions were performed in oven-dried glassware under a positive pressure of nitrogen. Solvents were transferred via syringe and were introduced into the reaction vessels with a rubber septum. Column chromatography was carried out on a column packed with silica-gel 60N spherical neutral size 63-210 µm. Analytical thin-layer chromatography (TLC) was performed on precoated (0.25 mm) silica-gel plates (Merch, Merck Silica Gel 60 $F_{254}$). Preparative TLC was carried out using precoated silica-gel plates (0.5 mm : Merck, Merck Silica Gel 60 $F_{254}$). NMR spectra were recorded on a JEOL JNM-ECZ700R, Bruker Avance 500, Bruker Avance 400 and Varian Mercury 300 instruments and are calibrated using residual undeutetrated solvent ($CHCl_3$ at 7.26 ppm $^1$H NMR, 77.16 ppm $^{13}$C NMR). The $CFCl_3$ [$\delta$ = 0.00 ($CDCl_3$)] was used as internal standard for $^{19}$F NMR. Mass spectra were recorded on a Shimadzu GCMS-QP5050A (EI-MS) and Shimadzu LCMS-2020 (ESI-MS). High resolution mass spectrometry were recorded on a Waters Synapt G2 HDMS (ESI-MS) and Waters, GCT Premier (EI-MS). GC analysis was carried out on a Shimadzu GC-2014 using GC Capillary Column CBP5-M25-025 (25 m, 0.22 mm ID, 0.25 µm df). Infrared spectra were recorded on a JASCOFT/IR-4100 spectrometer. Melting points were recorded on a BUCHI M-565. Commercially available chemicals were obtained from Acro Organics, Aldrich Chemical Co., Alfa Aesar, TCI, Kanto Chemical Co., Fujifilm Wako Pure Chemical Co., Ark Farm and used as received unless otherwise stated.

### Optimization study of nickel-catalyzed *ipso*-silylation (Supplementary Table 1-7)

General procedure for optimization study of *ipso*-silylation of fluoroarenes **1a**.

To a flame-dried screw-capped test tube were added a fluoroarene **1a** (0.20 mmol, 1 equiv), nickel source, a ligand, silylborane (**2a**, 0.30 mmol, 1.5 equiv), a base, an additive and a solvent (0.8 ml) in a glovebox filled with argon gas. The tube with the mixture was sealed and removed from the glovebox. The solution was stirred for the time and at temperature indicated in the table. The reaction tube was cooled to room temperature, and was added $Et_2O$ (20 ml), saturated aqueous ammonium chloride (1 ml), followed by *n*-decane (10 µl, 0.05 mmol) as an internal standard. After stirring the mixture vigorously, GC analysis was conducted using a portion of the resulting organic phase.

GC conditions for analyses of the silylated product **1a**: Constant linear column flow was adjusted to 64.2 ml/min. Temperatures of the injector and the detector were held at 300 °C respectively, and the GC oven temperature program was set as follows: initially held at 75 °C for 2 min, heated to 300 °C at the rate of 16 °C/min, and held at 300 °C for 2 min. Retention time: *n*-decane (4.48 min), 4-fluorobiphenyl (8.88 min), biphenyl (8.92 min) and **2a** (14.31 min).

### Preparation of triethyl(4,4,5,5-tetramethyl-1,3,2-dioxaborolan-2-yl)silane

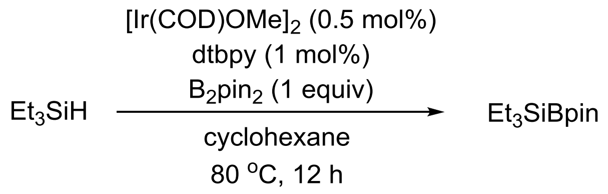

Following a literature procedure[1], an oven-dried vial was charged with $[Ir(COD)OMe]_2$ (26.5 mg, 0.04 mmol), dtbpy (21.6 mg, 0.08 mmol), $B_2pin_2$(2.052 g, 8.08 mmol), cyclohexane (8.0 mL), and triethylsilane (3.758 g, 32.32 mmol) inside a nitrogen filled glovebox. The resulting dark brown solution was heated at 80 °C overnight outside the glovebox. After being cooled to room temperature, the crude reaction mixture was concentrated in vacuo, and the residue was purified by flash column chromatography to afford the $Et_3SiBpin$ as colorless oil (1.18 g, 60%).
$^1H$ NMR (300 MHz, $CDCl_3$) δ 1.23 (s, 12H), 0.97 (t, *J* = 7.9 Hz, 9H), 0.59 (dd, *J* = 15.8, 7.9 Hz, 6H).
$^{11}B$ NMR (225 MHz, $CDCl_3$) δ 34.24.

**Synthesis of starting materials**

Previous reported methods for the preparation of compounds **1c**[2], **1d**[2], **1e**[2], **1f**[2], **1g**[3], **1h**[4], **1k**[4], **1p**[2], **1q**[5], **1r**[6], **1s**[7], and **1v**[8] have been followed.

**3-Fluoro-1,1'-biphenyl (1i)**

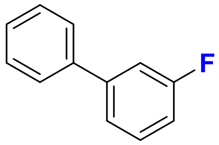


A dried flask was charged with 1-fluoro-3-iodobenzene (1.11 g, 5 mmol), phenylboronic acid (670 mg, 5.5 mmol), $(PPh_3)_4Pd$ (288 mg, 0.25 mmol) and dioxane (30 mL). The mixture was stirred at room temperature for 20 mins, then saturated aqueous $NaHCO_3$ (7 mL) was added. Then the mixture was heated at 110 °C and the reaction progress was monitored by TLC. After stirring for 18 h, the mixture was cooled to room temperature. To this was added water 3 ml, and then extracted with EtOAc (5 ml × 3). The combined organic phases was washed with brine (5ml), and dried over $Na_2SO_4$. After filtration, the filtrate was concentrated under reduced pressure. The residue was purified by column chromatography on silica gel (hexane) to give compound 3-fluoro-1,1'-biphenyl as white solid (645.0 mg, 75 %).
$^1H$ NMR (300 MHz, $CDCl_3$) δ 7.57 (d, *J* = 7.1 Hz, 2H), 7.51 – 7.33 (m, 5H), 7.29 (dd, *J* = 10.8, 1.6 Hz, 1H), 7.10 – 6.98 (m, 1H).
$^{19}F$ NMR (282 MHz, $CDCl_3$) δ -113.04 – -113.35 (m).
$^{13}C$ NMR (126 MHz, $CDCl_3$) δ 163.32 (d, *J* = 245.5 Hz), 143.62 (d, *J* = 7.7 Hz), 140.06 (d, *J* = 2.2 Hz), 130.34 (d, *J* = 8.5 Hz), 129.01, 127.97, 127.23, 122.89 (d, *J* = 2.8 Hz), 114.24 (d, *J* = 2.3 Hz), 114.07 (d, *J* = 3.2 Hz).
The chemical shifts were consist with those reported in the literature[9].

**2-Fluoro-1,1'-biphenyl (1j)**

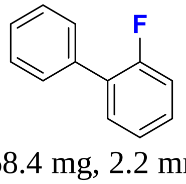


To a solution of phenylboronic acid (268.4 mg, 2.2 mmol), $Pd(OAc)_2$ (6.7 mg, 0.03 mmol) and $K_3PO_4$ (849 mg, 4 mmol) in DMF (5 ml) and $H_2O$ (5 ml) added 1-fluoro-2-iodobenzene (444 mg, 2 mmol), and then the mixture was stirred at room temperature under nitrogen gas. After stirring for 12 h, the mixture was cooled to room temperature. To this was added water 3 ml, and then extracted with $Et_2O$ (5 ml × 3). The combined organic phases was washed with brine (5ml), and

dried over $Na_2SO_4$. After filtration, the filtrate was concentrated under reduced pressure. The residue was purified by column chromatography on silica gel (hexane) to give compound 2-fluoro-1,1´-biphenyl as colorless liquid (337.0 mg, 98%).
$^1$H NMR (300 MHz, $CDCl_3$) δ 7.55 (d, *J* = 7.3 Hz, 2H), 7.44 (t, *J* = 6.7 Hz, 3H), 7.39 – 7.26 (m, 2H), 7.25 – 7.10 (m, 2H).
$^{19}$F NMR (282 MHz, $CDCl_3$) δ -118.12 (s).
The chemical shifts were consistent with those reported in the literature[10].

**(3-(4-Fluorophenoxy)propoxy)triisopropylsilane (1t)**

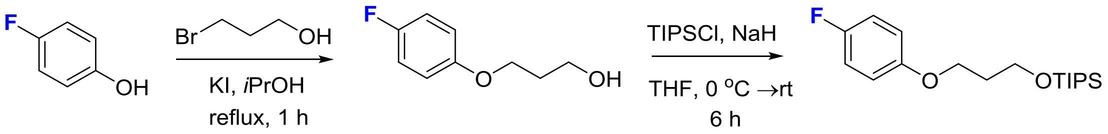


A solution of 4-fluorophenol (280 mg, 2.5 mmol), 3-bromo1-propanol (0.52 mg, 3.7 mmol) and KI (12 mg) in *i*PrOH (5 ml) was stirred and heated to reflux. After 1 h, the mixture was cooled to room temperature. To this was added water (3 ml) and then extracted with EtOAc (5 ml × 3). The combined organic extract was washed with brine (5 ml) and dried over $Na_2SO_4$. After filtration, the filtrate was concentrated under reduced pressure to give a crude mixture that contained 3-(4-fluorophenoxy)-1-propanol, which was used for the next step without further purification.

To a solution of the crude mixture in THF (10 ml) was added NaH (0.12 g, 3.0 mmol, 60% in mineral oil) at 0 °C. Then TIPSCl (0.74 ml, 3.5 mmol) was added at 0 °C and the reaction was stirred at room temperature overnight. After that, the reaction was quenched with water (5 ml) and extracted with EtOAc (5ml × 3). The combined organic extract was washed with brine (5 ml) and dried over $Na_2SO_4$. After filtration, the filtrate was concentrated under reduced pressure. The crude reaction mixture was purified by column chromatography on silica gel (hexane/EtOAc 40:1) to give (3-(4-fluorophenoxy)propoxy)triisopropylsilane as colorless liquid in 80 % yield (0.65 g).
$^1$H NMR (300 MHz, $CDCl_3$) δ 6.96 (t, *J* = 8.7 Hz, 2H), 6.87 – 6.79 (m, 2H), 4.05 (t, *J* = 6.2 Hz, 2H), 3.87 (t, *J* = 6.0 Hz, 2H), 2.05 – 1.93 (m, 2H), 1.10 – 0.98 (m, 21H).
$^{19}$F NMR (282 MHz, $CDCl_3$) δ -124.57 (ddd, *J* = 12.1, 8.1, 4.2 Hz).
$^{13}$C NMR (126 MHz, $CDCl_3$) δ 157.22 (d, *J* = 237.6 Hz), 155.32 (d, *J* = 2.0 Hz), 115.83 (d, *J* = 23.0 Hz), 115.49 (d, *J* = 7.9 Hz), 65.29, 59.85, 32.72, 18.13, 12.08.
IR (KBr) 2942, 2867, 1508, 1467, 1249, 1220, 1101, 883, 827.
HRMS (ESI) [$C_{18}H_{32}FO_2Si$] $(M+H)^+$ *calcd*. 327.2165, *found* 327.2155.

***N*-Benzyl-1-(3-fluorophenyl)-*N*-methylmethanamine (1w)**

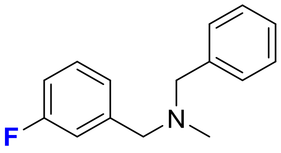


To the solution of 1-(3-fluorophenyl)-*N*-methylmethanamine (370.0 mg, 2.7 mmol) in THF (14 ml) was added NaH (130.0 mg, 3.2 mmol, 60% in mineral oil) at 0 °C. Then benzylbromide (0.39 ml, 3.2 mmol) was added at 0 °C. The reaction mixture was stirred at room temperature for 2 h. After that, the reaction was quenched with water (5 ml) and extracted with EtOAc (5ml × 3). The combined organic extract was washed with brine (5 ml) and dried over $Na_2SO_4$. After filtration, the filtrate was concentrated under reduced pressure. The crude reaction mixture was purified by

column chromatography on silica gel (hexane/EtOAc 10:1) to give *N*-benzyl-1-(3-fluorophenyl)-*N*-methylmethanamine as colorless liquid in 95% yield (588.0 mg).
$^{1}$H NMR (300 MHz, $CDCl_3$) δ 7.40 – 7.30 (m, 4H), 7.29 – 7.22 (m, 2H), 7.12 (d, *J* = 7.5 Hz, 2H), 6.98 – 6.88 (m, 1H), 3.53 (s, 2H), 3.50 (s, 2H), 2.18 (s, 3H).
$^{19}$F NMR (282 MHz, $CDCl_3$) δ -113.80 – -114.02 (m).
$^{13}$C NMR (126 MHz, $CDCl_3$) δ 163.11 (d, *J* = 245.2 Hz), 142.32, 139.18, 129.74 (d, *J* = 8.2 Hz), 129.03, 128.43, 127.19, 124.46 (d, *J* = 2.5 Hz), 115.67 (d, *J* = 21.3 Hz), 113.95 (d, *J* = 21.2 Hz), 62.04, 61.35, 42.38.
IR (KBr) 2955, 2786, 1589, 1486, 1452, 1365, 1253, 1025, 782, 738.
HRMS (ESI) [$C_{15}H_{17}FN$] $(M+H)^+$ *calcd.* 230.1345, *found* 230.1342.

**4-Fluoro-2-phenylpyridine (1x)**

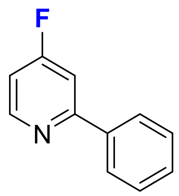


The solution of phenylboronic acid (268.4mg, 2.2 mmol), 2-bromo-4-fluoropyridine (352.0 mg, 2.0 mmol), and $(PPh_3)_4Pd$ (115.5 mg, 5 mol%) in dioxane (15 ml) was stirred at room temperature for 20 mins , then saturated aqueous $NaHCO_3$ (3.5 mL) was added. Then the mixture was heated at 105 $^{o}$C and the reaction progress was monitored by TLC. After stirring for 18 h, the mixture was cooled to room temperature. To this was added water (3 ml), and then extracted with EtOAc (5 ml × 3). The combined organic extract was washed with brine (5ml), and dried over $Na_2SO_4$. After filtration, the filtrate was concentrated under reduced pressure. The residue was purified by column chromatography on silica gel (hexane/EtOAc 10:1) to give 4-fluoro-2-phenylpyridine as colorless liquid in 70% yield (243.0 mg).
$^{1}$H NMR (300 MHz, $CDCl_3$) δ 8.65 (dd, *J* = 8.8, 5.6 Hz, 1H), 7.97 (dd, *J* = 8.0, 1.4 Hz, 2H), 7.54 – 7.38 (m, 4H), 7.01 – 6.91 (m, 1H).
$^{19}$F NMR (282 MHz, $cdcl_3$) δ -102.57 (dd, *J* = 18.6, 8.9 Hz).
The chemical shifts were consistent with those reported in the literature[11].

**General Procedure A**

To the solution of alcohol (1 equiv) in the DCM (0.4 M) was added DAST (1.1 equiv) dropwise at -20 $^{o}$C. Then the mixture was moved to room temperature and stirred for 6 h. After that, the reaction mixture was poured into cooled water, neutralized with aq. $NaHCO_3$, and extracted with DCM. The combined organic phase was dried over $MgSO_4$, filtered and evaporated under reduced pressure. The crude was purified by column chromatography on silica gel (hexane) to give fluorides.

**2-(Fluoromethyl)naphthalene (4a)**

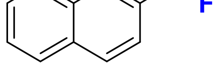


To the solution of 2-(bromomethyl)naphthalene (442.2 mg, 2.0 mmol) and pentaethylene glycol (238.0 mg, 1.0 mmol) in MeCN (10 ml) was added CsF (911.0 mg, 6.0 mmol) and heated for 1.5 h at 100 $^{o}$C. The reaction time was determined by TLC. The mixture was filtered and washed with

Et$_2$O (10 ml) and the filtrate was evaporated under reduced pressure. The residue was purified by column chromatography on silica gel (hexane) to give 2-(Fluoromethyl)naphthalene as colorless liquid in 89% yield (285.3 mg).
$^1$H NMR (300 MHz, CDCl$_3$) δ 8.07 (d, *J* = 7.9 Hz, 1H), 7.92 – 7.84 (m, 2H), 7.62 – 7.41 (m, 4H), 5.85 (d, *J* = 47.9 Hz, 2H).
$^{19}$F NMR (282 MHz, CDCl$_3$) δ -206.70 (t, *J* = 47.9 Hz).
$^{13}$C NMR (126 MHz, CDCl$_3$) δ 133.76 (d, *J* = 1.4 Hz), 131.86 (d, *J* = 15.4 Hz), 131.47 (d, *J* = 1.9 Hz), 129.99 (d, *J* = 3.3 Hz), 128.78, 126.98 (d, *J* = 8.4 Hz), 126.84 (d, *J* = 0.8 Hz), 126.22, 125.29 (d, *J* = 1.8 Hz), 123.67, 83.45 (d, *J* = 165.6 Hz).
The chemical shifts were consistent with those reported in the literature[12].

**1-(Fluoromethyl)naphthalene (4b)**

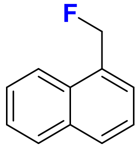


To the solution of 1-(bromomethyl)naphthalene (442.2 mg, 2.0 mmol) and pentaethylene glycol (238.0 mg, 1.0 mmol) in MeCN (10 ml) was added CsF (911.0 mg, 6.0 mmol) and heated for 1.5 h at 100 $^{o}$C. The reaction time was determined by TLC. The mixture was filtered and washed with Et$_2$O (10 ml) and the filtrate was evaporated under reduced pressure. The residue was purified by column chromatography on silica gel (hexane) to give 1-(fluoromethyl)naphthalene as colorless liquid in 71% yield (227.0 mg).
$^1$H NMR (300 MHz, CDCl$_3$) δ 8.07 (d, *J* = 7.8 Hz, 1H), 7.90 (d, *J* = 7.4 Hz, 2H), 7.69 – 7.38 (m, 4H), 5.85 (d, *J* = 47.9 Hz, 2H).
$^{19}$F NMR (282 MHz, CDCl$_3$) δ -206.17 (t, *J* = 47.9 Hz).
The chemical shifts were consistent with those reported in the literature[13].

**4-(Fluoromethyl)-1,1'-biphenyl (4c)**

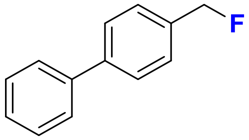


Following the general procedure **A**. Purification by column chromatography on silica gel (hexane) afforded **4c** as colorless liquid (800.3 mg, 5.0 mmol scale, 86% yield).
$^1$H NMR (300 MHz, CDCl$_3$) δ 7.62 (t, *J* = 7.9 Hz, 4H), 7.45 (t, *J* = 7.1 Hz, 4H), 7.41 – 7.33 (m, 1H), 5.43 (d, *J* = 47.8 Hz, 2H).
$^{19}$F NMR (282 MHz, CDCl$_3$) δ -206.15 (t, *J* = 47.9 Hz).
The chemical shifts were consistent with those reported in the literature[13].

**1-(*tert*-Butyl)-4-(fluoromethyl)benzene (4d)**

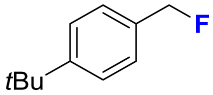


Following the general procedure **A**. Purification by column chromatography on silica gel (hexane) afforded **4d** as colorless liquid (756.1 mg, 5.0 mmol scale, 91% yield).
$^1$H NMR (300 MHz, CDCl$_3$) δ 7.43 (d, *J* = 7.9 Hz, 2H), 7.33 (dd, *J* = 8.3, 1.8 Hz, 2H), 5.35 (d, *J* = 48.1 Hz, 2H), 1.33 (s, 9H).

$^{19}$F NMR (282 MHz, $CDCl_3$) δ -204.29 (t, *J* = 48.1 Hz).
The chemical shifts were consistent with those reported in the literature[14].

**1-(Fluoromethyl)-4-(trifluoromethoxy)benzene (4e)**

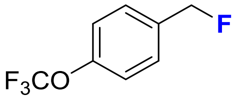


Following the general procedure **A**. Purification by column chromatography on silica gel (hexane) afforded **4e** as colorless liquid (815.0 mg, 5.0 mmol scale, 84% yield).
$^{1}$H NMR (300 MHz, $CDCl_3$) δ 7.43 (d, *J* = 1.7 Hz, 1H), 7.40 (d, *J* = 1.6 Hz, 1H), 7.26 (s, 1H), 7.23 (s, 1H), 5.39 (d, *J* = 47.6 Hz, 2H).
$^{19}$F NMR (282 MHz, $CDCl_3$) δ -57.90 (s), -207.76 (t, *J* = 47.5 Hz).
$^{13}$C NMR (126 MHz, $CDCl_3$) δ 149.56, 135.00 (d, *J* = 17.6 Hz), 129.06 (d, *J* = 5.9 Hz), 121.25, 120.56 (d, *J* = 256.9 Hz), 83.76 (d, *J* = 167.3 Hz).
IR (KBr) 2967, 2904, 1513, 1265, 1166, 993, 850, 673.
HRMS (EI) [$C_8H_6F4O$] ($M^+$) *calcd*. 194.0355, *found* 194.0352.

**(1-Fluoroethyl)benzene (4f)**

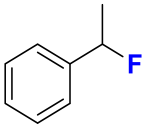


Following the general procedure **A**. Purification by column chromatography on silica gel (hexane) afforded **4f** as colorless liquid (477.6 mg, 5.0 mmol scale, 77% yield).
$^{1}$H NMR (300 MHz, $CDCl_3$) δ 7.45 – 7.26 (m, 5H), 5.62 (dq, *J* = 47.7, 6.4 Hz, 1H), 1.64 (dd, *J* = 23.9, 6.4 Hz, 3H).
$^{19}$F NMR (282 MHz, $CDCl_3$) δ -167.04 (dq, *J* = 47.8, 23.9 Hz).
The chemical shifts were consistent with those reported in the literature[14].

**(Cyclohexylfluoromethyl)benzene (4g)**

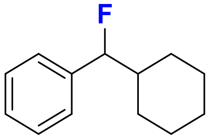


Following the general procedure **A**. Purification by column chromatography on silica gel (hexane) afforded **4g** as colorless liquid (738.5 mg, 5.0 mmol scale, 77% yield).
$^{1}$H NMR (300 MHz, $CDCl_3$) δ 7.49 – 7.16 (m, 5H), 5.11 (dd, *J* = 47.1, 7.1 Hz, 1H), 1.95 (d, *J* = 12.3 Hz, 1H), 1.87 – 1.57 (m, 4H), 1.40 (d, *J* = 12.4 Hz, 1H), 1.30 – 0.93 (m, 5H).
$^{19}$F NMR (282 MHz, $CDCl_3$) δ -180.13 (dd, *J* = 47.2, 16.1 Hz).
$^{13}$C NMR (126 MHz, $CDCl_3$) δ 139.44 (d, *J* = 20.5 Hz), 128.31, 128.20 (d, *J* = 1.8 Hz), 126.33 (d, *J* = 7.1 Hz), 98.85 (d, *J* = 172.6 Hz), 43.96 (d, *J* = 22.1 Hz), 28.42 (dd, *J* = 60.7, 4.8 Hz), 26.11 (d, *J* = 72.6 Hz), 26.00.
The chemical shifts were consistent with those reported in the literature[15].

**(1-Fluorobutyl)benzene (4h)**

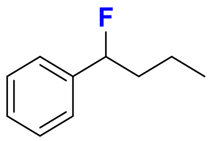

Following the general procedure **A**. Purification by column chromatography on silica gel (hexane) afforded **4h** as colorless liquid (690.5 mg, 5.0 mmol scale, 91% yield).
$^{1}$H NMR (300 MHz, $CDCl_3$) δ 7.46 – 7.27 (m, 4H), 5.43 (ddd, *J* = 47.9, 8.1, 4.9 Hz, 1H), 2.07 – 1.66 (m, 2H), 1.58 – 1.32 (m, 2H), 0.96 (t, *J* = 7.4 Hz, 3H).
$^{19}$F NMR (282 MHz, $CDCl_3$) δ -174.30 – -174.88 (m).
$^{13}$C NMR (126 MHz, $CDCl_3$) δ 140.75 (d, *J* = 19.7 Hz), 128.53, 128.28 (d, *J* = 2.0 Hz), 125.69 (d, *J* = 6.8 Hz), 94.63 (d, *J* = 169.9 Hz), 39.45 (d, *J* = 23.5 Hz), 18.55 (d, *J* = 4.6 Hz), 13.99.
IR (KBr) 2962, 2937, 2837, 1455, 952, 757, 698.
HRMS (EI) [$C_{10}H_{13}F$] ($M^+$) *calcd.* 152.1001, *found* 152.1006.

**9-Fluoro-9*H*-fluorene (4i)**

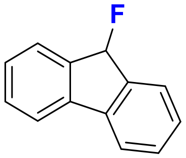

Following the general procedure **A**. Purification by column chromatography on silica gel (hexane) afforded **4j** as colorless liquid (828.0 mg, 5.0 mmol scale, 90% yield).
$^{1}$H NMR (300 MHz, $CDCl_3$) δ 7.71 – 7.55 (m, 4H), 7.42 (t, *J* = 7.4 Hz, 2H), 7.32 (t, *J* = 7.4 Hz, 2H), 6.31 (d, *J* = 54.2 Hz, 1H).
$^{19}$F NMR (282 MHz, $CDCl_3$) δ -187.06 (d, *J* = 54.2 Hz).
The chemical shifts were consistent with those reported in the literature[16].

**4-(1-Fluoroethyl)pyridine (4j)**

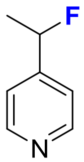

Following the general procedure **A**. Purification by column chromatography on silica gel (hexane) afforded **4j** as colorless liquid (506.5 mg, 5.0 mmol scale, 81% yield).
$^{1}$H NMR (300 MHz, $CDCl_3$) δ 8.63 (d, *J* = 5.3 Hz, 2H), 7.25 (d, *J* = 6.5 Hz, 2H), 5.63 (ddd, *J* = 48.1, 13.1, 6.6 Hz, 1H), 1.64 (dd, *J* = 24.2, 6.5 Hz, 3H).
$^{19}$F NMR (282 MHz, $CDCl_3$) δ -177.36 (dq, *J* = 48.1, 24.1 Hz).
The chemical shifts were consistent with those reported in the literature[17].

**(1-Fluorocyclobutyl)benzene (4k)**

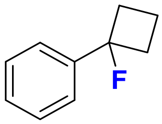

Following the general procedure **A**. Purification by column chromatography on silica gel (hexane) afforded **4k** as colorless liquid (600.0 mg, 5.0 mmol scale, 80% yield).

$^{1}H$ NMR (300 MHz, cdcl$_3$) δ 7.52 – 7.32 (m, 5H), 2.69 – 2.53 (m, 3H), 2.16 – 2.05 (m, 1H), 1.83 – 1.68 (m, 1H), 1.35 – 1.22 (m, 1H).
$^{19}F$ NMR (282 MHz, CDCl$_3$) δ -126.22 – -126.56 (m).
The chemical shifts were consistent with those reported in the literature[18].

**(4-Fluorobutyl)benzene (4l)**

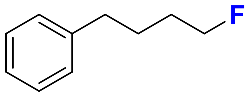


Following the general procedure **A**. Purification by column chromatography on silica gel (hexane) afforded **4l** as colorless liquid (676.8 mg, 5.0 mmol scale, 89% yield).
$^{1}H$ NMR (300 MHz, CDCl$_3$) δ 7.35 – 7.24 (m, 2H), 7.18 (d, *J* = 7.4 Hz, 3H), 4.61 – 4.31 (m, 2H), 2.66 (t, *J* = 6.9 Hz, 2H), 1.87 – 1.60 (m, 4H).
$^{19}F$ NMR (282 MHz, CDCl$_3$) δ -218.01 – -218.74 (m).
The chemical shifts were consistent with those reported in the literature[19].

**Silylation of *sp*$^2$ C− and *sp*$^3$ C−F bonds**

**General procedure B**

To a flame-dried screw-capped test tube were sequentially added a fluoroarene **1** (0.20 mmol, 1 equiv), silylborane **2** (0.30 mmol, 1.5 equiv), Ni(COD)$_2$ (5.5 mg, 0.02 mmol, 10 mol%), KO*t*Bu (56.1 mg, 0.5 mmol, 2.5 equiv) and *c*-hexane/THF (1/2, v/v, 0.8 ml) in a glovebox filled with argon gas. The tube with the mixture was sealed and removed from the glovebox, and stirred at room temperature for 2-12 h. The reaction progress was monitored by TLC. Then, the mixture was added saturated aqueous ammonium chloride (3 ml) and extracted with EtOAc (3 × 3 ml). The combined organic extract was dried over MgSO$_4$ and filtrated. The filtrate was concentrated under reduced pressure. The residue was purified to give the silanes.

**General procedure C**

To a flame-dried screw-capped test tube were sequentially added a fluoroalkanes **1** (0.20 mmol, 1 equiv), silylborane **2** (0.30 mmol, 1.5 equiv), KO*t*Bu (56.1 mg, 0.5 mmol, 2.5 equiv) and *c*-hexane/THF (1/2, v/v, 0.8 ml) in a glovebox filled with argon gas. The tube with the mixture was sealed and removed from the glovebox, and stirred at room temperature for 2-12 h. The reaction progress was monitored by TLC. The mixture was then added saturated aqueous ammonium chloride (3 ml) and extracted with EtOAc (3 × 3 ml). The combined organic extract was dried over MgSO$_4$ and filtrated. The filtrate was concentrated under reduced pressure. The residue was purified to give the silanes.

**[1,1'-Biphenyl]-4-yltriethylsilane (3a)**

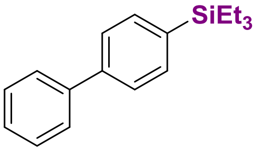


Following the general procedure **B**. Purification by column chromatography on silica gel (hexane) afforded **3a** as colorless liquid (38.0 mg, 71% yield).
$^{1}H$ NMR (300 MHz, CDCl$_3$) δ 7.63 – 7.55 (m, 6H), 7.44 (t, *J* = 7.4 Hz, 2H), 7.36 – 7.32 (m, 1H), 1.03 – 0.97 (m, 9H), 0.86 – 0.78 (m, 6H).

The chemical shifts were consistent with those reported in the literature[20].

**[1,1'-Biphenyl]-4-yldimethyl(phenyl)silane (3b)**

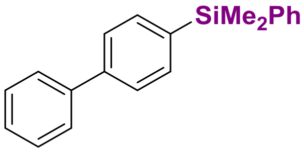


Following the general procedure **B**, **1a** (34.4 mg, 0.2 mmol), $PhMe_2SiBpin$ (78.6 mg, 0.3 mmol), $Ni(COD)_2$ (5.5 mg, 10 mol%), KO*t*Bu (56.1 mg, 0.5 mmol), *c*-heane-THF (0.8 ml, v/v, 1/2). Purification by column chromatography on silica gel (hexane) afforded **3b** as colorless liquid (32.0 mg, 55% yield).
$^1H$ NMR (300 MHz, $CDCl_3$) δ 7.59 (s, 7H), 7.48 – 7.28 (m, 7H), 0.59 (s, 6H).
The chemical shifts were consistent with those reported in the literature[21].

**Triethyl(4'-methoxy-[1,1'-biphenyl]-4-yl)silane (3c)**

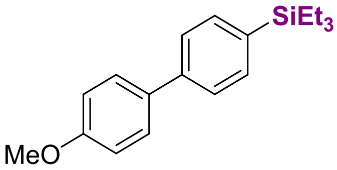


Following the general procedure **B**. Purification by column chromatography on silica gel (pentane) afforded **3c** as colorless liquid (38.0 mg, 63% yield).
$^1H$ NMR (300 MHz, $CDCl_3$) δ 7.59 – 7.49 (m, 6H), 7.01 – 6.93 (m, 2H), 3.84 (s, 3H), 0.99 (t, *J* = 7.7 Hz, 9H), 0.87 – 0.76 (m, 6H).
$^{13}C$ NMR (126 MHz, $CDCl_3$) δ 159.28, 141.13, 135.54, 134.81, 133.80, 128.25, 126.09, 114.30, 55.44, 7.59, 3.53.
The chemical shifts were consistent with those reported in the literature[22].

**Triethyl(4'-methyl-[1,1'-biphenyl]-4-yl)silane (3d)**

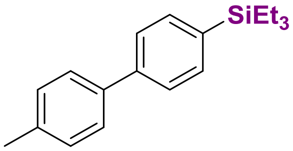


Following the general procedure **B**. Purification by column chromatography on silica gel (pentane) afforded **3d** as colorless liquid (47.6 mg, 84% yield).
$^1H$ NMR (300 MHz, $CDCl_3$) δ 7.60 – 7.54 (m, 4H), 7.53 – 7.47 (m, 2H), 7.28 – 7.20 (m, 2H), 2.39 (s, 3H), 1.05 – 0.93 (m, 9H), 0.87 – 0.77 (m, 6H).
$^{13}C$ NMR (126 MHz, $CDCl_3$) δ 141.47, 138.41, 137.19, 135.97, 134.79, 129.60, 127.09, 126.33, 21.27, 7.61, 3.54.
The chemical shifts were consistent with those reported in the literature[23].

**(4-(Benzo[*d*][1,3]dioxol-5-yl)phenyl)triethylsilane (3e)**

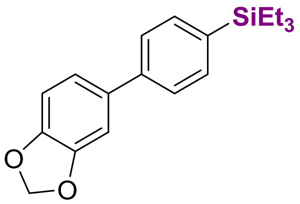


Following the general procedure **B**, reaction time 12 h. Purification by column chromatography on silica gel (hexane/EtOAc = 20/1) afforded **3e** as colorless oil (31.3 mg, 51% yield).
Mp 52-55 $^{o}$C.
$^{1}$H NMR (300 MHz, $CDCl_3$) δ 7.57 – 7.46 (m, 4H), 7.08 (dd, *J* = 6.9, 1.7 Hz, 2H), 6.92 – 6.85 (m, 1H), 6.00 (s, 2H), 0.99 (t, *J* = 7.7 Hz, 9H), 0.86 – 0.77 (m, 6H).
$^{13}$C NMR (126 MHz, $CDCl_3$) δ 148.22, 147.21, 141.22, 135.99, 135.68, 134.82, 126.24, 120.77, 108.72, 107.75, 101.27, 7.59, 3.52.
IR (KBr) 2952, 2873, 1479, 1226, 1016, 808, 738.
HRMS (EI) [$C_{19}H_{24}O_2Si$] ($M^+$) *calcd*. 312.1546, *found* 312.1557.

**_N_-(4-(Triethylsilyl)phenyl)pyrrole (3f)**

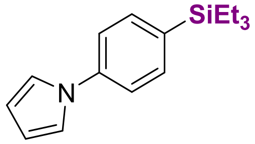


Following the general procedure **B**. Purification by column chromatography on silica gel (pentane) afforded **3f** as colorless liquid (32.5 mg, 62% yield).
$^{1}$H NMR (300 MHz, $CDCl_3$) δ 7.58 – 7.49 (m, 2H), 7.42 – 7.33 (m, 2H), 7.14 – 7.06 (m, 2H), 6.34 (d, *J* = 1.1 Hz, 2H), 0.98 (dd, *J* = 8.2, 7.2 Hz, 9H), 0.87 – 0.76 (m, 6H).
$^{13}$C NMR (126 MHz, $CDCl_3$) δ 141.16, 135.59, 134.60, 119.81, 119.29, 110.51, 7.54, 3.51.
IR (KBr) 2952, 2875, 1596, 1330, 1070, 817, 721.
HRMS (ESI) [$C_{16}H_{25}NSi$] $(M+H)^+$ *calcd*. 258.1678, *found* 258.1680.

**(4-(Benzo[_b_]thiophen-3-yl)phenyl)triethylsilane (3g)**

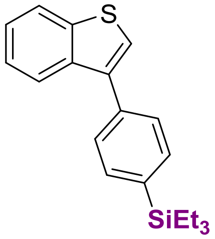


Following the general procedure **B**. Purification by column chromatography on silica gel (hexane) afforded **3g** as colorless oil (35.2 mg, 54% yield).
$^{1}$H NMR (300 MHz, $CDCl_3$) δ 8.00 – 7.87 (m, 2H), 7.59 (q, *J* = 7.9 Hz, 3H), 7.47 – 7.34 (m, 4H), 1.02 (t, *J* = 7.7 Hz, 9H), 0.89 – 0.83 (m, 6H).
$^{13}$C NMR (126 MHz, $CDCl_3$) δ 140.86, 138.28, 138.00, 136.88, 136.39, 134.74, 128.04, 124.53, 124.43, 123.59, 123.19, 123.06, 7.61, 3.53.
IR (KBr) 3058, 2952, 2908, 2875, 1457, 1236, 821, 759, 730.
HRMS (EI) [$C_{20}H_{24}SSi$] ($M^+$) *calcd*. 324.1368, *found* 324.1357.

**1-Methyl-2-(4-(triethylsilyl)phenyl)-1_H_-indole (3h)**

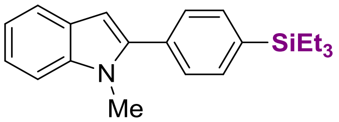


Following the general procedure **B**. Purification by column chromatography on silica gel (hexane) afforded **3h** as white solid (43.0 mg, 67% yield).
Mp 70-72 °C.
$^1$H NMR (300 MHz, $CDCl_3$) δ 7.68 – 7.56 (m, 3H), 7.50 (d, *J* = 8.1 Hz, 1H), 7.37 (d, *J* = 8.1 Hz, 1H), 7.29 – 7.21 (m, 2H), 7.17 – 7.10 (m, 1H), 6.58 (s, 1H), 3.78 (s, 3H), 1.05 – 0.96 (m, 9H), 0.89 – 0.81 (m, 6H).
$^{13}$C NMR (126 MHz, $CDCl_3$) δ 141.82, 138.55, 137.35, 134.45, 133.12, 128.60, 128.11, 121.77, 120.61, 119.96, 109.74, 101.81, 31.45, 7.60, 3.50.
IR (KBr) 3430, 2942, 2873, 1467, 1103, 1014, 732.
HRMS (ESI) [$C_{21}H_{28}NSi$] $(M+H)^+$ *calcd*. 322.1991, *found* 322.1991.

**[1,1'-Biphenyl]-3-yltriethylsilane (3i)**

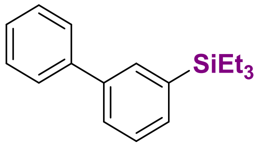


Following the general procedure **B**. Purification by column chromatography on silica gel (pentane) afforded **3i** as colorless liquid (40.2 mg, 76% yield).
$^1$H NMR (300 MHz, $CDCl_3$) δ 7.69 (s, 1H), 7.62 – 7.53 (m, 3H), 7.51 – 7.38 (m, 4H), 7.38 – 7.30 (m, 1H), 1.05 – 0.93 (m, 9H), 0.88 – 0.77 (m, 6H).
$^{13}$C NMR (126 MHz, $CDCl_3$) δ 141.83, 140.52, 138.16, 133.30, 133.11, 128.87, 128.17, 127.81, 127.43, 127.27, 7.61, 3.51.
The chemical shifts were consistent with those reported in the literature[20].

**[1,1'-Biphenyl]-2-yltriethylsilane (3j)**

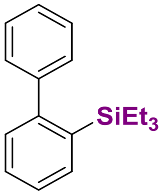


Following the general procedure **B**. Purification by column chromatography on silica gel (pentane) afforded **3j** as colorless liquid (40.2 mg, 69% yield).
$^1$H NMR (300 MHz, $CDCl_3$) δ 7.59 – 7.53 (m, 1H), 7.39 – 7.32 (m, 4H), 7.31 – 7.19 (m, 4H), 0.81 (t, *J* = 7.8 Hz, 9H), 0.46 (q, *J* = 7.8 Hz, 6H).
$^{13}$C NMR (126 MHz, $CDCl_3$) δ 149.79, 144.71, 135.92, 135.24, 129.79, 129.30, 128.42, 127.70, 127.18, 126.19, 7.65, 4.31.
The chemical shifts were consistent with those reported in the literature[20].

**1-Methyl-5-(triethylsilyl)-1*H*-indole (3k)**

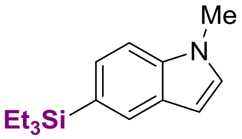

Following the general procedure **B**. Purification by PLC (hexane) afforded **3k** as colorless liquid (30.0 mg, 61% yield).
$^{1}$H NMR (300 MHz, $CDCl_3$) δ 7.62 (dd, *J* = 7.9, 0.7 Hz, 1H), 7.44 (d, *J* = 0.7 Hz, 1H), 7.22 (dd, *J* = 7.9, 0.8 Hz, 1H), 7.04 (d, *J* = 3.1 Hz, 1H), 6.46 (dd, *J* = 3.1, 0.8 Hz, 1H), 3.82 (s, 3H), 1.04 – 0.95 (m, 9H), 0.90 – 0.80 (m, 6H).
$^{13}$C NMR (126 MHz, $CDCl_3$) δ 136.74, 129.16, 129.09, 129.06, 124.82, 120.34, 115.04, 100.84, 32.93, 7.70, 3.86.
IR (KBr) 2952, 2873, 1509, 1332, 1241, 1008, 717.
HRMS (EI) [$C_{15}H_{23}NSi$] ($M^+$) *calcd*. 245.1600, *found* 245.1596.

**Triethyl(naphthalene-1yl)silane (3l)**

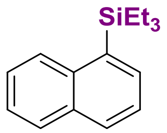


Following the general procedure **B**. Purification by column chromatography on silica gel (hexane) afforded **3l** as colorless liquid (30.0 mg, 61% yield).
$^{1}$H NMR (300 MHz, $CDCl_3$) δ 8.09 (d, *J* = 7.5 Hz, 1H), 7.85 (d, *J* = 7.9 Hz, 2H), 7.67 (dd, *J* = 6.8, 1.2 Hz, 1H), 7.53 – 7.39 (m, 3H), 1.12 – 0.89 (m, 15H).
$^{13}$C NMR (126 MHz, $CDCl_3$) δ 137.64, 135.32, 134.60, 133.50, 129.71, 129.20, 128.03, 125.64, 125.36, 125.18, 7.82, 4.65.
The chemical shifts were consistent with those reported in the literature[20].

**Triethyl(phenyl)silane (3m)**

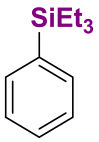


Following the general procedure **B**. Purification by PLC (hexane) afforded **3m** as colorless liquid (28.5 mg, 75% yield).
$^{1}$H NMR (300 MHz, $CDCl_3$) δ 7.54 – 7.44 (m, 2H), 7.40 – 7.29 (m, 3H), 1.03 – 0.90 (m, 9H), 0.79 (dd, *J* = 15.5, 7.7 Hz, 6H).
$^{13}$C NMR (126 MHz, $CDCl_3$) δ 137.58, 134.33, 128.83, 127.78, 7.55, 3.45.
The chemical shifts were consistent with those reported in the literature[20].

**Triethyl(*p*-tolyl)silane (3n)**

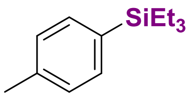


Following the general procedure **B**. Purification by column chromatography on silica gel (hexane) afforded **3n** as colorless liquid (25.8 mg, 62% yield).
$^{1}$H NMR (300 MHz, $CDCl_3$) δ 7.39 (d, *J* = 7.9 Hz, 2H), 7.17 (d, *J* = 8.0 Hz, 2H), 2.35 (s, 3H), 1.01 – 0.91 (m, 9H), 0.83 – 0.71 (m, 6H).
$^{13}$C NMR (126 MHz, $CDCl_3$) δ 138.63, 134.38, 133.82, 128.67, 21.61, 7.57, 3.53.
IR (KBr) 2954, 2915, 2881, 1459, 1105, 1008, 798, 721.
HRMS (EI) [$C_{13}H_{22}Si$] ($M^+$) *calcd*. 206.1491, *found* 206.1487.
The chemical shifts were consistent with those reported in the literature[24].

**(4-Methoxy)phenyltriethylsilane (3o)**

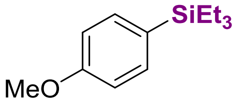


Following the general procedure **B**. Purification by column chromatography on silica gel (hexane) afforded **3o** as colorless liquid (32.0 mg, 70% yield).
$^1$H NMR (300 MHz, $CDCl_3$) δ 7.42 (d, $J$ = 8.6 Hz, 2H), 6.91 (d, $J$ = 8.6 Hz, 2H), 3.81 (s, 3H), 1.01 – 0.90 (m, 9H), 0.83 – 0.70 (m, 6H).
$^{13}$C NMR (126 MHz, $CDCl_3$) δ 160.28, 135.70, 128.27, 113.58, 55.09, 7.56, 3.63.
IR (KBr) 2954, 2908, 2875, 2834, 1594, 1275, 1247, 809, 734.
HRMS (EI) [$C_{13}H_{22}OSi$] $(M-CH_2CH_3)^+$ *calcd*. 193.1049, *found* 193.1044.

**Triethyl(4-(methoxymethoxy)-phenyl)silane (3p)**

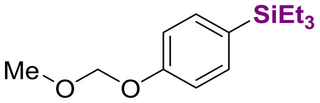


Following the general procedure **B**. Purification by column chromatography on silica gel (hexane) afforded **3p** as colorless liquid (30.0 mg, 58% yield).
$^1$H NMR (300 MHz, $CDCl_3$) δ 7.44 – 7.38 (m, 2H), 7.06 – 7.00 (m, 2H), 5.19 (s, 2H), 3.48 (s, 3H), 0.98 – 0.92 (m, 10H), 0.81 – 0.73 (m, 6H).
$^{13}$C NMR (126 MHz, $CDCl_3$) δ 157.97, 135.71, 129.82, 115.70, 94.32, 56.20, 7.56, 3.60.
IR (KBr) 2954, 2875, 1594, 1153, 1006, 734.
HRMS (EI) [$C_{14}H_{24}O_2Si$] ($M^+$) *calcd*. 252.1546, *found* 252.1543.

**Triethyl(4-phenoxyphenyl)silane (3q)**

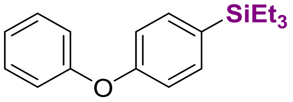


Following the general procedure **B**. Purification by column chromatography on silica gel (hexane) afforded **3q** as colorless liquid (36.8 mg, 65% yield).
$^1$H NMR (300 MHz, $CDCl_3$) δ 7.47 – 7.41 (m, 2H), 7.37 – 7.29 (m, 2H), 7.15 – 7.07 (m, 1H), 7.06 – 6.95 (m, 4H), 1.03 – 0.90 (m, 9H), 0.84 – 0.71 (m, 6H).
$^{13}$C NMR (126 MHz, $CDCl_3$) δ 158.21, 156.94, 135.85, 131.43, 129.87, 123.54, 119.43, 117.98, 7.55, 3.58.
IR (KBr) 2954, 2875, 1583, 1488, 1241, 1105, 734.
HRMS (EI) [$C_{18}H_{24}OSi$] ($M^+$) *calcd*. 284.1596, *found* 284.1605.

***N*,*N*-Dimethyl-4-(triethylsilyl)aniline (3r)**

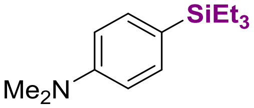


Following the general procedure **B**. Purification by PLC (hexane/EtOAc = 40/1) afforded **3r** as colorless liquid (36.0 mg, 77% yield).
$^1$H NMR (300 MHz, $CDCl_3$) δ 7.36 (d, $J$ = 7.7 Hz, 1H), 6.73 (d, $J$ = 7.7 Hz, 2H), 2.95 (s, 6H), 1.06 – 0.88 (m, 9H), 0.84 – 0.66 (m, 6H).
$^{13}$C NMR (126 MHz, $CDCl_3$) δ 150.91, 135.40, 122.49, 112.00, 40.36, 7.65, 3.73.

The chemical shifts were consistent with those reported in the literature[20].

**(4-Benzylphenyl)triethylsilane (3s)**

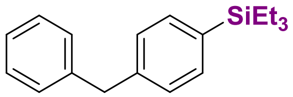


Following the general procedure **B**. Purification by column chromatography on silica gel (hexane) afforded **3s** as colorless liquid (34.6 mg, 61% yield).
$^{1}$H NMR (300 MHz, $CDCl_3$) δ 7.40 (d, *J* = 8.0 Hz, 2H), 7.33 – 7.25 (m, 2H), 7.23 – 7.14 (m, 5H), 3.97 (s, 2H), 1.01 – 0.89 (m, 9H), 0.82 – 0.70 (m, 6H).
$^{13}$C NMR (126 MHz, $CDCl_3$) δ 141.71, 141.11, 134.79, 134.53, 129.16, 128.59, 128.38, 126.21, 42.08, 7.57, 3.51.
The chemical shifts were consistent with those reported in the literature[20].

**Triethyl(4-(3-((triisopropylsilyl)oxy)propoxy)phenyl)silane (3t)**

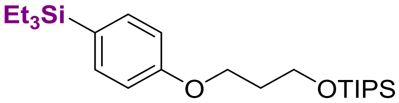


Following the general procedure **B**. Purification by PLC (hexane/EtOAc = 40/1) afforded **3t** as colorless liquid (61.0 mg, 72% yield).
$^{1}$H NMR (300 MHz, $CDCl_3$) δ 7.39 (d, *J* = 8.5 Hz, 2H), 6.90 (d, *J* = 8.4 Hz, 2H), 4.10 (t, *J* = 6.3 Hz, 2H), 3.87 (t, *J* = 6.0 Hz, 2H), 2.06 – 1.95 (m, 2H), 1.09 – 1.02 (m, 21H), 0.95 (t, *J* = 7.7 Hz, 9H), 0.76 (q, *J* = 7.7 Hz, 6H).
$^{13}$C NMR (126 MHz, $CDCl_3$) δ 159.82, 135.65, 127.96, 114.14, 64.48, 60.07, 32.80, 18.14, 12.09, 7.57, 3.64.
IR (KBr) 2950, 2869, 1594, 1502, 1465, 1272, 1245, 1182, 1108, 1016, 970, 883, 823,
HRMS (ESI) [$C_{24}H_{46}NaO_2Si_2$] $(M+Na)^+$ *calcd.* 445.2934, *found* 444.2941.

**(2-Methylphenyl)triethylsilane (3u)**

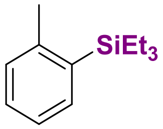


Following the general procedure **B**. Purification by PLC (hexane) afforded **3u** as colorless liquid (25.5 mg, 61% yield).
$^{1}$H NMR (300 MHz, $CDCl_3$) δ 7.42 (d, *J* = 7.4 Hz, 1H), 7.30 – 7.22 (m, 1H), 7.15 (t, *J* = 6.4 Hz, 2H), 2.43 (s, 3H), 0.97 – 0.92 (m, 9H), 0.90 – 0.83 (m, 6H).
$^{13}$C NMR (126 MHz, $CDCl_3$) δ 144.02, 135.59, 129.89, 129.07, 124.84, 23.14, 7.74, 4.10.
The chemical shifts were consistent with those reported in the literature[25].

**Triethyl(3-(4,4,5,5-tetramethyl-1,3,2-dioxaborolan-2-yl)phenyl)silane (3v)**

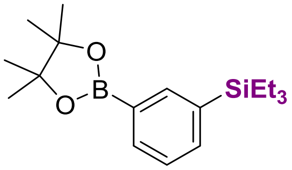


Following the general procedure **B**. Purification by PLC (hexane) afforded **3v** as colorless liquid (42.0 mg, 65% yield).

$^{1}H$ NMR (300 MHz, $CDCl_3$) δ 7.91 (s, 1H), 7.79 (dd, *J* = 7.1, 5.8 Hz, 1H), 7.54 (dd, *J* = 24.6, 7.4 Hz, 1H), 7.34 (t, *J* = 7.4 Hz, 1H), 1.34 (s, 12H), 1.00 – 0.91 (m, 9H), 0.87 – 0.76 (m, 6H).
$^{13}C$ NMR (126 MHz, $CDCl_3$) δ 140.64, 137.29, 135.39, 133.86, 133.68, 127.03, 83.80, 25.02, 7.58, 3.43.
IR (KBr) 2954, 2875, 1355, 1307, 1145, 1126, 1076, 730.
HRMS (EI) [$C_{18}H_{31}BO_2Si$] ($M^+$) *calcd*. 318.2186, *found* 318.2194.

**_N_-Benzyl-_N_-methyl-1-(3-(triethylsilyl)phenyl)methanamine (3w)**

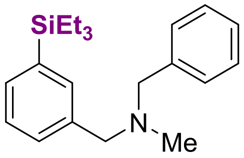


Following the general procedure **B**. Purification by PLC (hexane/EtOAc = 40/1) afforded **3w** as colorless liquid (35.0 mg, 53% yield).
$^{1}H$ NMR (300 MHz, $CDCl_3$) δ 7.47 (s, 1H), 7.40 – 7.22 (m, 8H), 3.52 (d, *J* = 9.3 Hz, 4H), 2.20 (s, 3H), 0.96 (t, *J* = 7.5 Hz, 9H), 0.79 (dd, *J* = 15.1, 7.4 Hz, 6H).
$^{13}C$ NMR (126 MHz, $CDCl_3$) δ 139.60, 138.30, 137.29, 134.93, 132.97, 129.57, 129.04, 128.32, 127.69, 127.01, 62.19, 61.71, 42.47, 7.59, 3.51.
IR (KBr) 2952, 2875, 1455, 1018, 736.
HRMS (ESI) [$C_{21}H_{32}NSi$] $(M+H)^+$ *calcd*. 326.2304, *found* 326.2301.

**2-Phenyl-4-(triethylsilyl)pyridine (3x)**

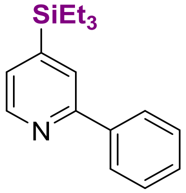


Following the general procedure **B**. Purification by PLC (hexane/EtOAc = 40/1) afforded **3x** as red color liquid (35.0 mg, 53% yield).
$^{1}H$ NMR (300 MHz, $CDCl_3$) δ 8.65 (d, *J* = 4.2 Hz, 1H), 7.97 (d, *J* = 7.2 Hz, 2H), 7.79 (s, 1H), 7.56 – 7.37 (m, 3H), 7.31 (d, *J* = 4.2 Hz, 1H), 1.00 (t, *J* = 7.3 Hz, 9H), 0.85 (dd, *J* = 14.5, 6.8 Hz, 6H).
$^{13}C$ NMR (126 MHz, $CDCl_3$) δ 156.34, 148.60, 148.55, 140.04, 128.89, 128.86, 127.65, 127.19, 126.12, 77.41, 77.16, 76.91, 7.43, 3.02.
IR (KBr) 2954, 2875, 1587, 1523, 1369, 1010, 721.
HRMS (ESI) [$C_{17}H_{24}NSi$] $(M+H)^+$ *calcd*. 270.1678, *found* 270.1676.

**3-Fluoro-5-(triethylsilyl)pyridine (3aa)**

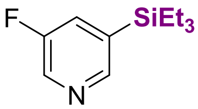


Following the general procedure **B,** using $Et_3SiBpin$ (1.1 equiv). Purification by PLC (hexane) afforded **3aa** as colorless liquid (22.4 mg, 55% yield).
$^{1}H$ NMR (300 MHz, $CDCl_3$) δ 8.38 (s, 2H), 7.29 (dd, *J* = 7.9, 3.2 Hz, 1H), 0.97 (t, *J* = 7.0 Hz, 9H), 0.88 (t, *J* = 7.1 Hz, 6H).
$^{19}F$ NMR (282 MHz, $CDCl_3$) δ -114.04.

$^{13}$C NMR (126 MHz, $CDCl_3$) δ 164.09 (d, *J* = 250.3 Hz), 144.98 (d, *J* = 3.7 Hz), 137.39 (d, *J* = 29.2 Hz), 133.11 (d, *J* = 28.6 Hz), 129.87 (d, *J* = 7.7 Hz), 7.30, 3.11.
IR (KBr) 2956, 2877, 1402, 1268, 1006, 833, 717.
HRMS (ESI) [$C_{11}H_{19}FNSi$] $(M+H)^+$ *calcd*. 212.1271, *found* 212.1270.

**Triethyl(2-fluorophenyl)silane (3bb)**

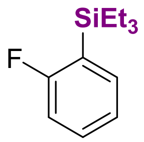


Following the general procedure **B,** using $Et_3SiBpin$ (1.1 equiv). Purification by PLC (hexane) afforded **3bb** as colorless liquid (29.5 mg, 70% yield).
$^1$H NMR (300 MHz, $CDCl_3$) δ 7.44 – 7.29 (m, 2H), 7.12 (t, *J* = 7.3 Hz, 1H), 6.98 (t, *J* = 8.4 Hz, 1H), 1.01 – 0.91 (m, 9H), 0.90 – 0.80 (m, 6H).
$^{19}$F NMR (282 MHz, $CDCl_3$) δ -99.50.
$^{13}$C NMR (126 MHz, $CDCl_3$) δ 167.60 (d, *J* = 240.6 Hz), 136.13 (d, *J* = 11.9 Hz), 131.22 (d, *J* = 8.3 Hz), 123.84 (d, *J* = 2.9 Hz), 123.31 (d, *J* = 31.5 Hz), 114.82 (d, *J* = 26.3 Hz), 7.49, 3.53 (d, *J* = 1.4 Hz).
IR (KBr) 2956, 2877, 1469, 1438, 1203, 1006, 821, 757, 734.
HRMS (EI) [$C_{12}H_{19}FSi$] ($M^+$) *calcd*. 210.1240, *found* 210.1241.

**Triethyl(2,3,4-trifluorophenyl)silane (3cc)**

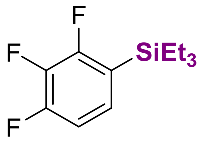


Following the general procedure **B,** using $Et_3SiBpin$ (1.1 equiv). Purification by PLC (hexane) afforded **3cc** as colorless liquid (40.0 mg, 81% yield).
$^1$H NMR (300 MHz, $CDCl_3$) δ 7.11 (ddd, *J* = 18.4, 9.2, 5.2 Hz, 1H), 6.82 – 6.68 (m, 1H), 1.06 – 0.79 (m, 15H).
$^{19}$F NMR (282 MHz, $CDCl_3$) δ -102.39 – -103.10 (m, 1F), -121.85 (dd, *J* = 23.3, 8.9 Hz, 1F), -143.16 – -143.96 (m, 1F).
$^{13}$C NMR (126 MHz, $CDCl_3$) δ 162.01 (ddd, *J* = 240.0, 13.9, 2.5 Hz), 154.04 (ddd, *J* = 244.5, 17.0, 12.9 Hz), 147.16 (ddd, *J* = 245.5, 17.1, 3.9 Hz), 118.58 (ddd, *J* = 19.6, 10.9, 1.8 Hz), 113.76 (ddd, *J* = 38.0, 31.3, 2.2 Hz), 110.92 (ddd, *J* = 30.3, 5.5, 3.9 Hz), 7.40 (s), 4.15 (t, *J* = 2.7 Hz).
IR (KBr) 2958, 2879, 1465, 1232, 1176, 1004, 991, 811, 738, 703.
HRMS (EI) [$C_{10}H_{12}F_3Si$] $(M-CH_2CH_3)^+$ *calcd*. 217.0660, *found* 217.0664.

**Triethyl(naphthalene-2-ylmethyl)silane (5a)**

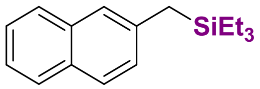


Following the general procedure **C**. Purification by column chromatography on silica gel (hexane) afforded **5a** as colorless liquid (40.5 mg, 79% yield).

$^{1}H$ NMR (300 MHz, $CDCl_3$) δ 8.00 – 7.92 (m, 1H), 7.85 – 7.77 (m, 1H), 7.59 (d, *J* = 8.1 Hz, 1H), 7.50 – 7.41 (m, 2H), 7.38 – 7.30 (m, 1H), 7.18 (d, *J* = 7.1 Hz, 1H), 2.57 (s, 2H), 0.87 (t, *J* = 7.9 Hz, 9H), 0.51 (q, *J* = 7.9 Hz, 6H).
$^{13}C$ NMR (126 MHz, $CDCl_3$) δ 137.61, 134.02, 131.94, 128.74, 125.65, 125.44, 125.41, 125.12, 124.70, 124.67, 18.51, 7.46, 3.66.
The chemical shifts were consistent with those reported in the literature[20].

**Triethyl(naphthalen-1-ylmethyl)silane (5b)**

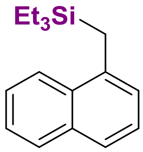


Following the general procedure **C**. Purification by column chromatography on silica gel (hexane) afforded **5b** as colorless liquid (36.0 mg, 70% yield).
$^{1}H$ NMR (300 MHz, $CDCl_3$) δ 8.03 – 7.95 (m, 1H), 7.87 – 7.79 (m, 1H), 7.61 (d, *J* = 8.1 Hz, 1H), 7.53 – 7.43 (m, 2H), 7.40 – 7.32 (m, 1H), 7.20 (d, *J* = 7.0 Hz, 1H), 2.59 (s, 2H), 0.89 (t, *J* = 7.9 Hz, 9H), 0.60 – 0.47 (m, 6H).
$^{13}C$ NMR (126 MHz, $CDCl_3$) δ 137.60, 134.02, 131.94, 128.74, 125.65, 125.44, 125.41, 125.12, 124.70, 124.67, 18.51, 7.46, 3.66.
The chemical shifts were consistent with those reported in the literature[20].

**([1,1'-Biphenyl]-4-ylmethyl)triethylsilane (5c)**

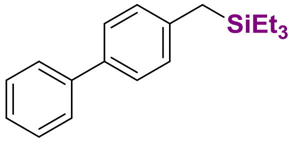


Following the general procedure **C**. Purification by column chromatography on silica gel (hexane) afforded **5c** as colorless liquid (45.3 mg, 81% yield).
$^{1}H$ NMR (300 MHz, $CDCl_3$) δ 7.63 – 7.53 (m, 2H), 7.42 (dd, *J* = 16.5, 8.0 Hz, 4H), 7.28 (dd, *J* = 11.6, 4.3 Hz, 1H), 7.08 (d, *J* = 8.2 Hz, 2H), 2.13 (s, 2H), 0.94 (t, *J* = 7.9 Hz, 9H), 0.53 (q, *J* = 7.8 Hz, 6H).
$^{13}C$ NMR (126 MHz, $CDCl_3$) δ 141.32, 140.07, 136.76, 128.80, 128.63, 126.96, 126.90, 126.86, 21.43, 7.48, 3.11.
IR (KBr) 2952, 2875, 1486, 1413, 1211, 1153, 1008, 838, 752.
HRMS (EI) [$C_{19}H_{26}Si$] ($M^+$) *calcd*. 282.1804, *found* 282.1793.

**(4-(*tert*-Butyl)benzyl)triethylsilane (5d)**

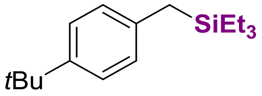


Following the general procedure **C**. Purification by column chromatography on silica gel (hexane) afforded **5d** as colorless liquid (37.5 mg, 72% yield).
$^{1}H$ NMR (300 MHz, $CDCl_3$) δ 7.21 (d, *J* = 8.1 Hz, 2H), 6.94 (d, *J* = 8.0 Hz, 2H), 2.05 (s, 2H), 1.29 (s, 9H), 0.92 (t, *J* = 7.9 Hz, 9H), 0.50 (q, *J* = 7.9 Hz, 6H).
$^{13}C$ NMR (126 MHz, $CDCl_3$) δ 146.59, 137.42, 127.88, 125.12, 34.32, 31.60, 20.91, 7.47, 3.09.
IR (KBr) 2954, 2910, 2875, 1511, 1014, 831, 773.
HRMS (EI) [$C_{17}H_{30}Si$] ($M^+$) *calcd*. 262.2117, *found* 262.2111.

**Triethyl(4-(trifluoromethoxy)benzyl)silane (5e)**

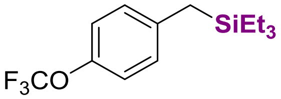


Following the general procedure **C**. Purification by PLC (hexane) afforded **5e** as colorless liquid (37.7 mg, 65% yield).
$^{1}$H NMR (300 MHz, $CDCl_3$) δ 7.03 (q, *J* = 8.6 Hz, 4H), 2.10 (s, 2H), 0.91 (t, *J* = 7.9 Hz, 9H), 0.50 (q, *J* = 7.9 Hz, 6H).
$^{13}$C NMR (126 MHz, $CDCl_3$) δ 146.10 (d, *J* = 1.8 Hz), 139.76, 129.12, 120.97, 120.69 (q, *J* = 256.1 Hz), 21.24, 7.39, 3.00.
IR (KBr) 2956, 2911, 1506, 1261, 1164, 1016, 773, 727.
HRMS (EI) [$C_{14}H_{21}F_3OSi$] ($M^+$) *calcd*. 290.1314, *found* 290.1320.

**Triethyl(1-phenylethyl)silane (5f)**

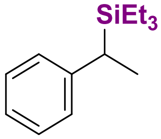


Following the general procedure **C**. Purification by PLC (hexane) afforded **5f** as colorless liquid (33.5 mg, 76% yield).
$^{1}$H NMR (300 MHz, $CDCl_3$) δ 7.28 – 7.19 (m, 2H), 7.07 (d, *J* = 7.7 Hz, 3H), 2.31 (q, *J* = 7.5 Hz, 1H), 1.37 (d, *J* = 7.6 Hz, 3H), 0.89 (t, *J* = 7.9 Hz, 9H), 0.51 (q, *J* = 7.9 Hz, 6H).
$^{13}$C NMR (126 MHz, $CDCl_3$) δ 146.43, 128.13, 127.25, 124.30, 26.93, 15.53, 7.64, 2.17.
IR (KBr) 2952, 2875, 1490, 1452, 1006, 786, 763, 700.
HRMS (EI) [$C_{14}H_{24}Si$] ($M^+$) *calcd*. 2201647, *found* 220.1654.

**(Cyclohexyl(phenyl)methyl)triethylsilane (5g)**

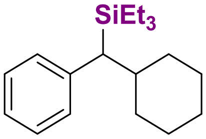


Following the general procedure **C**. Purification by PLC (pentane) afforded **5g** as colorless liquid (31.2 mg, 54% yield).
$^{1}$H NMR (300 MHz, $CDCl_3$) δ 7.20 (t, *J* = 7.4 Hz, 2H), 7.08 (d, *J* = 7.1 Hz, 1H), 7.01 (d, *J* = 7.2 Hz, 2H), 1.95 (s, 1H), 1.92 (s, 1H), 1.77 (dd, *J* = 23.0, 12.2 Hz, 2H), 1.64 – 1.58 (m, 1H), 1.52 (dd, *J* = 3.2, 1.7 Hz, 1H), 1.36 – 1.19 (m, 2H), 1.16 – 1.01 (m, 3H), 0.86 (t, *J* = 7.9 Hz, 9H), 0.55 – 0.45 (m, 6H).
$^{13}$C NMR (126 MHz, $CDCl_3$) δ 144.40, 129.11, 128.03, 124.37, 41.84, 40.50, 34.07 (d, *J* = 37.0 Hz), 26.92 (d, *J* = 21.0 Hz), 26.62, 7.87, 4.22.
IR (KBr) 3021, 2921, 2875, 2852, 1448, 1010, 771, 701.
HRMS (EI) [C17H27Si] ($M-CH_2CH_3)^+$ *calcd*. 259.1882, *found* 259.1884.

**(1-Phenylbutyl)triethylsilane (5h)**

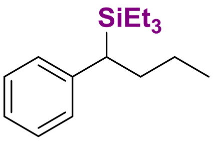


Following the general procedure **C**. Purification by PLC (Hexane) afforded **5h** as colorless liquid (34.8 mg, 70% yield).
$^1$H NMR (300 MHz, $CDCl_3$) δ 7.22 (dd, *J* = 13.4, 6.1 Hz, 3H), 7.06 (t, *J* = 8.4 Hz, 2H), 2.17 (dd, *J* = 12.3, 2.9 Hz, 1H), 1.92 – 1.76 (m, 1H), 1.71 – 1.56 (m, 1H), 1.40 – 1.23 (m, 1H), 1.19 – 1.03 (m, 1H), 1.00 – 0.81 (m, 12H), 0.50 (q, *J* = 7.9 Hz, 6H).
$^{13}$C NMR (126 MHz, $CDCl_3$) δ 144.21, 128.13, 128.08, 124.27, 33.86, 32.09, 22.47, 14.02, 7.67, 2.46.
IR (KBr) 2954, 2875, 1457, 1012, 804, 769, 700.
HRMS (ESI) [$C_{16}H_{28}NaSi$] $(M+Na)^+$ *calcd*. 271.1858, *found* 271.1857.

**(9*H*-Fluoren-9-yl)triethylsilane (5i)**

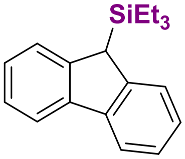


Following the general procedure **B**. Purification by PLC (Hexane) afforded **5i** as colorless liquid (41.0 mg, 73% yield).
$^1$H NMR (300 MHz, $CDCl_3$) δ 7.86 (d, *J* = 7.4 Hz, 2H), 7.50 (d, *J* = 7.2 Hz, 2H), 7.39 – 7.27 (m, 4H), 4.02 (s, 1H), 0.82 (t, *J* = 7.8 Hz, 9H), 0.49 (q, *J* = 7.9 Hz, 6H).
$^{13}$C NMR (126 MHz, $CDCl_3$) δ 145.97, 140.64, 126.06, 125.26, 124.18, 120.02, 39.38, 7.48, 2.37.
IR (KBr) 3060, 2952, 1446, 1238, 1049, 1004, 781, 736.
HRMS (EI) [$C_{19}H_{24}Si$] ($M^+$) *calcd*. 280.1647, *found* 280.1653.

**4-(1-(Triethylsilyl)ethyl)pyridine (5j)**

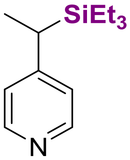


Following the general procedure **C**. Purification by PLC (Hexane) afforded **5j** as colorless liquid (34.0 mg, 77% yield).
$^1$H NMR (300 MHz, $CDCl_3$) δ 8.40 (d, *J* = 4.7 Hz, 2H), 6.99 (d, *J* = 4.3 Hz, 2H), 2.33 (dd, *J* = 14.9, 7.6 Hz, 1H), 1.38 (d, *J* = 7.2 Hz, 3H), 0.90 (t, *J* = 7.8 Hz, 9H), 0.52 (dd, *J* = 15.6, 7.8 Hz, 6H).
$^{13}$C NMR (126 MHz, $CDCl_3$) δ 155.97, 149.34, 122.71, 27.20, 14.49, 7.57, 2.01.
IR (KBr) 2954, 2875, 1594, 1459, 1014, 827, 796, 717.
HRMS (ESI) [$C_{13}H_{23}NSi$] $(M+H)^+$ *calcd*. 222.1678, *found* 222.1683.

**(2-Phenylcyclobutyl)triethylsilane (5k)**

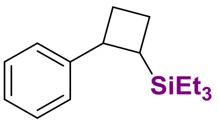

Following the general procedure **B**. Purification by PLC (Hexane) afforded **5k** as colorless liquid (20.0 mg, 40% yield).
$^1$H NMR (300 MHz, $CDCl_3$) δ 7.28 (d, *J* = 4.3 Hz, 4H), 7.20 – 7.13 (m, 1H), 3.43 (dd, *J* = 17.5, 8.9 Hz, 1H), 2.44 – 2.34 (m, 1H), 2.33 – 2.23 (m, 1H), 2.06 (dd, *J* = 14.7, 5.6 Hz, 1H), 2.00 – 1.88 (m, 2H), 0.96 – 0.84 (m, 9H), 0.63 – 0.42 (m, 6H).
$^{13}$C NMR (126 MHz, $CDCl_3$) δ 146.76, 128.29, 126.86, 125.92, 42.79, 32.12, 29.49, 20.05, 7.72, 2.57.
IR (KBr) 2952, 2875, 1459, 1236, 1014, 730.
HRMS (EI) [$C_{16}H_{26}Si$] ($M^+$) *calcd*. 246.1804, *found* 246.1808.

**(4-Phenylbutyl)triethylsilane (5l)**

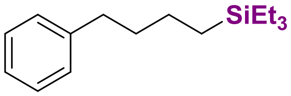

Following the general procedure **C**. Purification by PLC (Hexane) afforded **5l** as colorless liquid (35.0 mg, 70% yield)
$^1$H NMR (300 MHz, $CDCl_3$) δ 7.32 – 7.23 (m, 2H), 7.18 (d, *J* = 7.0 Hz, 3H), 2.66 – 2.56 (m, 2H), 1.63 (dt, *J* = 15.2, 7.4 Hz, 2H), 1.35 (dt, *J* = 15.8, 7.7 Hz, 2H), 0.92 (t, *J* = 7.9 Hz, 9H), 0.64 – 0.39 (m, 8H).
$^{13}$C NMR (126 MHz, $CDCl_3$) δ 143.09, 128.52, 128.35, 125.65, 35.87, 35.80, 23.77, 11.32, 7.63, 3.45.
IR (KBr) 2950, 2873, 1452, 1413, 1014, 723,698.
HRMS (ESI) [$C_{16}H_{28}NaSi$] $(M+Na)^+$ *calcd*. 271.1858, *found* 271.1857.

**Defluorosilylation of the fluroing-containing pharmaceuticals**

**Synthesis of 3-fluoroestrone derivative (6)**

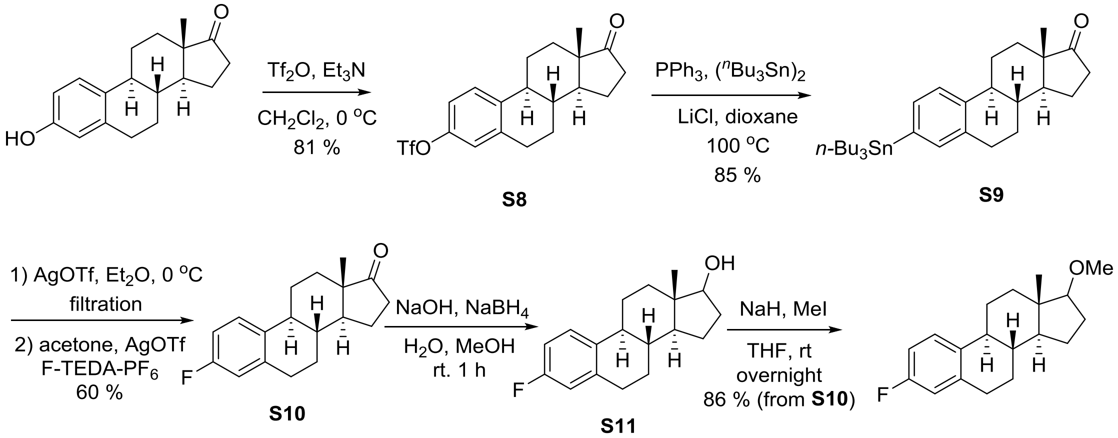


Synthetic intermediates **S8**, **S9** and **S10** were prepared according to procedures described in the literatures: Furuya, T., Strom, A. E. & Ritter. T. *J. Am. Chem. Soc*. **131**, 1662–1663 (2009).

**3-Fluoro-17-methoxyl-estra-1,3,5(10)-triene (6)**

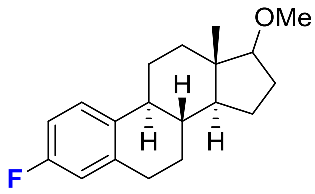

To the solution of **S10** in MeOH (8 ml) was added NaOH aqueous (3.48 mmol, 0.70 ml, 5 N). Then $NaBH_4$ (0.120 g, 3.24 mmol) in MeOH (5 ml) was dropwise added and stirred for 1 h at room temperature. After completion, the mixture was diluted by water (5 ml), extracted by EtOAc (3 × 8 ml), washed by brine (10 ml) and dried over $MgSO_4$. After filtration, the filtrate was concentrated under reduced pressure. The residue **S11** was used for the next step without further purification.

To the solution of **S11** in THF (20 ml) was added NaH (0.115 g, 2.88 mmol, 60% in mineral oil) at 0 °C. After 15 min, MeI (0.35 ml, 5.52 mmol) was added. The mixture was stirred at room temperature overnight. To the mixture was added water (20 ml) and extracted by EtOAc (3 × 8 ml). The organic phases was dried over $MgSO_4$, filtrated and concentrated under reduced pressure. The residue was purified by column chromatography on silica gel (hexane/EtOAc = 40/1) to give **6** as white solid (0.296 g, 86%).

$^1$H NMR (300 MHz, $CDCl_3$) δ 7.21 (d, *J* = 6.3 Hz, 1H), 6.90 – 6.70 (m, 2H), 3.38 (s, 3H), 3.35 – 3.27 (m, 1H), 2.93 – 2.75 (m, 2H), 2.33 – 2.15 (m, 2H), 2.12 – 1.99 (m, 2H), 1.95 – 1.83 (m, 1H), 1.76 – 1.63 (m, 1H), 1.59 – 1.18 (m, 7H), 0.79 (s, 3H). $^{19}$F NMR (282 MHz, $CDCl_3$) δ -118.62 (dd, *J* = 14.6, 8.1 Hz).

$^{19}$F NMR (282 MHz, $CDCl_3$) δ -118.62 (dd, *J* = 14.6, 8.1 Hz).

$^{13}$C NMR (126 MHz, $CDCl_3$) δ 161.02 (d, *J* = 243.6 Hz), 139.04 (d, *J* = 6.9 Hz), 136.11 (d, *J* = 2.9 Hz), 126.92 (d, *J* = 7.9 Hz), 115.18 (d, *J* = 20.0 Hz), 112.42 (d, *J* = 20.7 Hz), 90.86 , 58.06, 50.40, 44.08, 43.31, 38.48, 38.11, 29.76 (d, *J* = 1.4 Hz), 27.89, 27.14, 26.56, 23.19, 11.68.

IR (KBr) 2923, 2869, 1587, 1494, 1135, 1105, 813.

HRMS (EI) [$C_{19}H_{25}FO$] ($M^+$) *calcd*. 288.1889, *found* 288.1878.

**Triethyl((8*R*,9*S*,13*S*,14*S*)-17-methoxy-13-methyl-7,8,9,11,12,13,14,15,16,17-decahydro-6*H*-cyclopenta[a]phenanthren-3-yl)silane (7)**

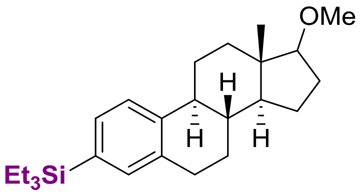

Following the general procedure **B**. Purification by PLC (hexane/EtOAc = 40/1) afforded **7** as white solid (31.5 mg, 41% yield).

Mp 109-114 °C.

$^1$H NMR (300 MHz, $CDCl_3$) δ 7.27 (s, 2H), 7.19 (s, 1H), 3.38 (s, 3H), 3.31 (dd, *J* = 14.3, 5.8 Hz, 1H), 2.95 – 2.81 (m, 2H), 2.38 – 2.20 (m, 2H), 2.11 – 2.00 (m, 2H), 1.94 – 1.85 (m, 1H), 1.75 – 1.65 (m, 1H), 1.54 – 1.28 (m, 5H), 1.27 – 1.17 (m, 1H), 0.97 (t, *J* = 7.7 Hz, 9H), 0.85 – 0.68 (m, 9H).

$^{13}$C NMR (126 MHz, $CDCl_3$) δ 141.07, 135.93, 135.19, 134.20, 131.68, 124.78, 90.93, 58.06, 50.62, 44.66, 43.36, 38.40, 38.25, 29.74, 27.90, 27.41, 26.14, 23.18, 11.68, 7.65, 3.55.

IR (KBr) 2950, 2869, 1454, 1415, 1382, 1101, 1012, 975, 717, 676.

HRMS (EI) [$C_{25}H_{40}OSi$] ($M^+$) *calcd*. 384.2848, *found* 384.2848.

## Synthesis of Pitavastatin Derivative (8)

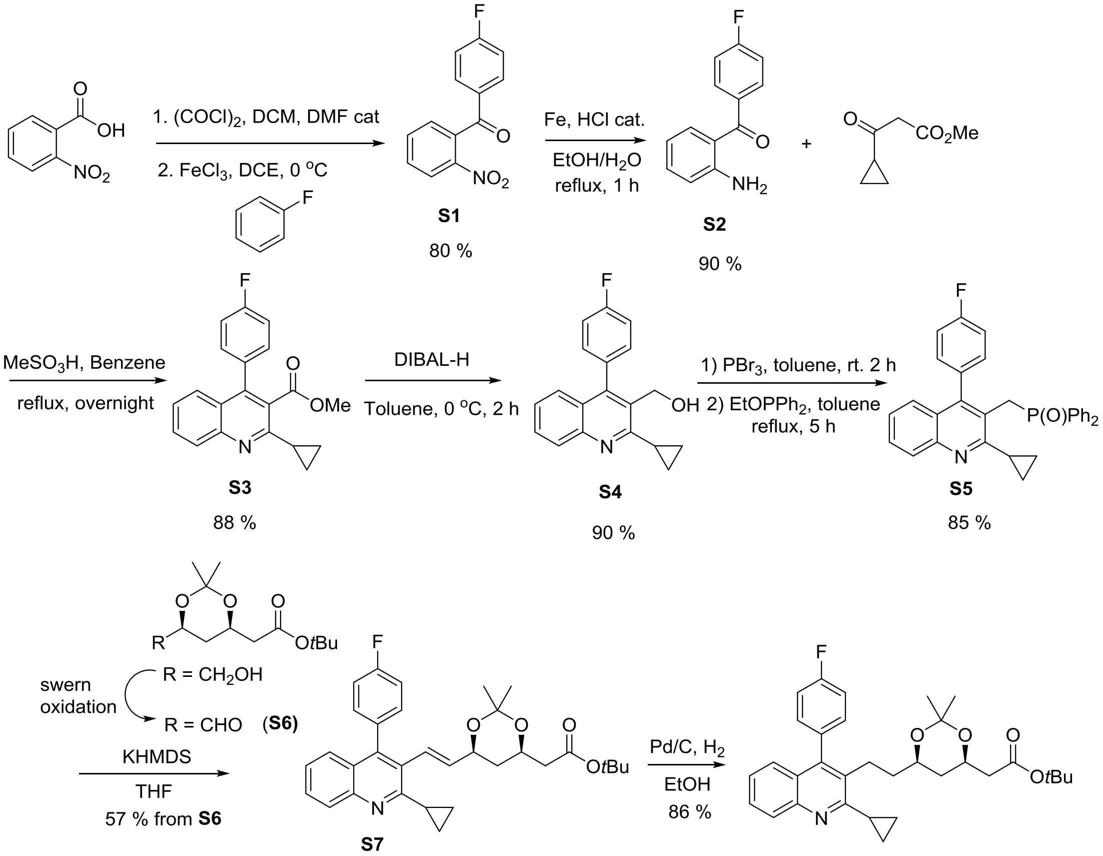


Synthetic intermediates **S1** and **S2** were prepared according to procedures described in the literatures: Zhang, J., Zhu, D., Yu, C., Wan, C. & Wang, Z. *Org. Lett.* **12**, 2841-2843 (2010); Counceller, C. M., Eichman, C. C., Wray, B. C. & Stambuli, J. P. *Org. Lett.* **10**, 1021-1023 (2008). Compounds **S3** and **S4** were obtained according to Suzuki, M., Iwasaki, H., Fujikawa, Y., Kitahara, M., Sakashita, M. & Sakoda, R. *Bioorg. Med. Chem.* **9**, 2727-2743 (2001); Makarov, I., Kuwahara, T., Jusseau, X., Ryu, I., Lindhardt, A. T. & Skrydstrup, T. *J. Am. Chem. Soc.* **137**, 14043-14046 (2015). Compounds **S5** was prepared according to Hiyama, T., Minami, T. & Takahashi, K. *Bull. Chem. Soc. Jpn.* **68**, 364-372 (1995).

### ***tert*-butyl 2-((4*R*,6*S*)-6-((*E*)-2-(2-cyclopropyl-4-(4-fluorophenyl)quinolin-3-yl)vinyl)-2,2-dimethyl-1,3-dioxan-4-yl)acetate (S7)**

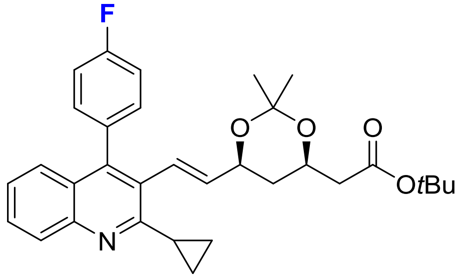

The solution of oxalyl chloride (0.27 ml, 3.12 mmol) in $CH_2Cl_2$ (15 ml) was added DMSO (0.22 ml, 3.12 mmol) at −78 °C. After 5 min, to the mixture was added a solution of *tert*-butyl (3*R*,5*S*)-

6-hydroxy-3,5-*O*-isopropylidene-3,5-dihydroxyhexanoate (0.745 g, 2.86 mmol) in $CH_2Cl_2$ (5 ml) at −78 °C. After 15 min, to this was added $Et_3N$ (1.8 ml, 13 mmol) at −78 °C and stirred for 30 min. Then, the mixture was heated to room temperature and stirred for 6 h. To this solution was added saturated aqueous sodium bicarbonate (3 ml) and extracted with EtOAc (5 ml × 3). The combined organic extract was dried over $MgSO_4$ and filtrated. The filtrate was concentrated under reduced pressure. The residue containing aldehyde **S6** was used for the next step without further purification.

To a solution of KHMDS ( 0.57 g, 2.86 mmol) in THF (5 ml) was added a solution of **S5** (1.25 g, 2.6 mmol) in THF (5 ml) at −78 °C and stirred for 5 min. A solution of crude aldyhyde **S6** in THF (10 ml) was added at −78 °C. Then the mixture was stirred for 12 h at 0 °C. To the mixture was added saturated aqueous ammonium chloride (5 ml) and extracted with EtOAc (5 ml × 3 ml). The organic phase was dried over $MgSO_4$ and filtrated. The filtrate was concentrated under reduced pressure. The residue was purified by column chromatography silica gel (hexane/EtOAc = 10/1) to give **S7** as colorless oil (0.76 g, 57%).

$^{1}$H NMR (300 MHz, $CDCl_3$) δ 7.94 (d, *J* = 8.3 Hz, 1H), 7.58 (ddd, *J* = 8.3, 6.4, 1.8 Hz, 1H), 7.39 – 7.26 (m, 2H), 7.24 – 7.08 (m, 4H), 6.55 (dd, *J* = 16.3, 1.0 Hz, 1H), 5.57 (dd, *J* = 16.3, 6.1 Hz, 1H), 4.35 (dd, *J* = 10.1, 6.1 Hz, 1H), 4.29 – 4.18 (m, 1H), 2.49 – 2.34 (m, 2H), 2.26 (dd, *J* = 15.3, 6.3 Hz, 1H), 1.53 – 1.40 (m, 12H), 1.40 – 1.32 (m, 5H), 1.32 – 1.23 (m, 1H), 1.10 – 1.01 (m, 2H), 1.01 – 0.90 (m, 1H).

$^{13}$C NMR (126 MHz, $CDCl_3$) δ 170.31, 162.36 (d, *J* = 246.9 Hz), 160.88, 146.90, 144.45, 138.14, 133.48 (d, *J* = 3.5 Hz), 132.23 (d, *J* = 8.0 Hz), 131.81 (d, *J* = 8.0 Hz), 129.30, 129.00, 128.86, 126.18, 126.07, 125.49, 115.35 (dd, *J* = 21.4, 9.2 Hz), 98.93, 80.83, 69.97, 65.92, 42.64, 36.44, 30.14, 28.22, 19.89, 16.09, 10.72, 10.30.

$^{19}$F NMR (282 MHz, $CDCl_3$) δ -114.49 – -114.70 (m).

IR (KBr) 2983, 2938, 1727, 1511, 1378, 1218, 1159, 1091, 1027, 981, 956, 917, 835, 763, 728.

HRMS (ESI) [$C_{32}H_{37}FNO_4$] $(M+H)^+$ *calcd*. 518.2707, *found* 518.2683.

***tert*-Butyl 2-((4*R*,6*R*)-6-(2-(2-cyclopropyl-4-(4-fluorophenyl)quinolin-3-yl)ethyl)-2,2-dimethyl-1,3-dioxan-4-yl)acetate (8)**

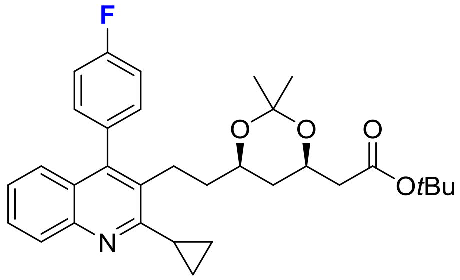


To the solution of protected pitavastatin **S7** (0.28 g, 0.537 mmol) in EtOH (17 ml) was added palladium on charcoal (5.0% Pd/C, 0.14 g) and stirred under $H_2$ at room temperature. After completion, the mixture was filtrated through a pad of Celite®, the filtrate was concentrated under reduced pressure. The residue was purified by column chromatography silica gel (hexane/EtOAc = 10/1) to give protected dihydorpitavastain **8** as a colorless oil ( 0.24 g, 86%).

$^{1}$H NMR (300 MHz, $CDCl_3$) δ 7.93 (d, *J* = 8.4 Hz, 1H), 7.61 – 7.50 (m, 1H), 7.30 – 7.25 (m, 2H), 7.24 – 7.08 (m, 5H), 4.22 – 4.13 (m, 1H), 3.80 – 3.64 (m, 1H), 2.99 – 2.78 (m, 2H), 2.70 – 2.55 (m, 1H), 2.46 – 2.33 (m, 2H), 2.30 – 2.13 (m, 2H), 1.67 – 1.54 (m, 2H), 1.44 (s, 9H), 1.38 (s, 3H), 1.30 (s, 3H), 1.29 – 1.21 (m, 2H), 1.09 – 1.03 (m, 2H).

$^{13}C$ NMR (126 MHz, $CDCl_3$) δ 170.45, 162.45 (d, *J* = 246.7 Hz), 161.90, 146.42, 144.94, 133.62 (d, *J* = 3.5 Hz), 131.89, 131.18 (t, *J* = 8.2 Hz), 128.89, 128.29, 126.73, 125.95, 125.27, 115.61 (dd, *J* = 21.4, 8.9 Hz), 98.73, 80.76, 68.59, 66.17, 42.72, 37.21, 36.14, 30.14, 28.22, 25.33, 19.85, 14.67, 10.17, 10.10.
$^{19}F$ NMR (282 MHz, $CDC_3$) δ -114.42 – -114.61 (m).
IR (KBr) 2935, 2865, 1727, 1604, 1509, 1367, 1155, 846.
HRMS (EI) [$C_{32}H_{38}FNO_4$] ($M^+$) *calcd*. 519.2785, *found* 519.2788.

***tert*-Butyl 2-((4R,6R)-6-(2-(2-cyclopropyl-4-(4-(triethylsilyl)phenyl)quinolin-3-yl)ethyl)-2,2-dimethyl-1,3-dioxan-4-yl)acetate (9)**

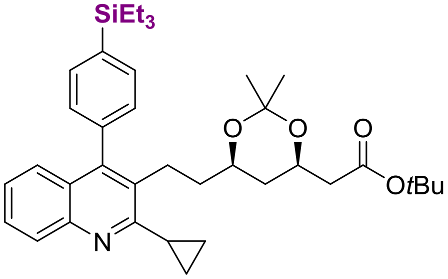


Following the general procedure **B**. Purification by PLC (hexane/EtOAc = 6/1) afforded **9** as colorless oil (31.0 mg, 50% yield, 0.1 mmol scale).
$^{1}H$ NMR (300 MHz, $CDCl_3$) δ 7.93 (d, *J* = 8.1 Hz, 1H), 7.59 – 7.51 (m, 1H), 7.25 – 7.12 (m, 6H), 3.62 – 3.53 (m, 1H), 2.84 – 2.60 (m, 3H), 2.47 – 2.30 (m, 2H), 2.13 (t, *J* = 7.2 Hz, 2H), 1.76 – 1.60 (m, 3H), 1.44 (s, 11H), 1.38 – 1.27 (m, 5H), 1.11 – 0.99 (m, 3H), 0.91 (t, *J* = 7.9 Hz, 9H), 0.52 (q, *J* = 7.8 Hz, 6H).
$^{13}C$ NMR (126 MHz, $CDCl_3$) δ 172.96, 163.46, 161.81, 161.50, 146.37, 144.83, 133.67, 132.10, 131.18, 128.86, 128.25, 126.77, 125.92, 125.26, 115.71, 115.54, 80.19, 71.92, 37.64, 35.98, 35.67, 28.24, 25.69, 21.04, 14.61, 10.23, 7.05, 5.11.
IR (KBr) 2958, 2881, 1727, 1604, 1577, 1511, 1492, 1457, 1411, 1224, 1155, 1016, 840, 742.
HRMS (ESI) [$C_{38}H_{54}NO_4Si$] $(M+H)^+$ *calcd*. 616.3822, *found* 616.3826.

**Synthetic application**

**C-H bond arylation**

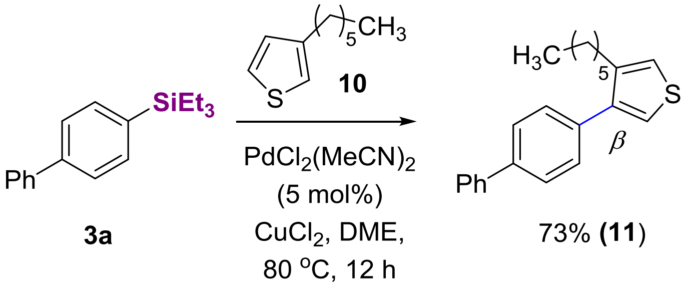


A dried Schlenk tube was charged with **3a** (107.4 mg, 0.4 mmol), $CuCl_2$ (53.8 mg, 0.4 mmol), $PdCl_2(MeCN)_2$ (7.6 mg, 0.01 mmol) and **10** (33.7 mg, 0.2 mmol). Dried 1,2-dichloroethane (1 ml) was added by syringe under $N_2$ protected. The mixture was stirred at 80 °C for 12 h. After cooling to room temperature, the mixture was diluted with DCM (2 ml) and filtered through a silica plug,

eluting with DCM (2 ml). The filtrate was concentrated and purified by column chromatography silica gel (hexane) to give the product **11** as a colorless oil (45 mg, 73%).
$^1$H NMR (300 MHz, $CDCl_3$) δ 7.70 – 7.62 (m, 4H), 7.52 – 7.44 (m, 4H), 7.42 – 7.34 (m, 1H), 7.23 (dd, $J$ = 3.3, 1.0 Hz, 1H), 7.11 – 7.05 (m, 1H), 2.72 – 2.63 (m, 2H), 1.59 (p, $J$ = 7.1 Hz, 2H), 1.36 – 1.24 (m, 6H), 0.88 (t, $J$ = 6.6 Hz, 3H).
$^{13}$C NMR (126 MHz, $CDCl_3$) δ 142.69, 141.69, 140.88, 139.85, 136.45, 129.26, 128.93, 127.43, 127.16, 127.12, 123.19, 121.19, 31.73, 30.13, 29.45, 29.25, 22.70, 14.22.
HRMS (ESI) [$C_{22}H_{25}S$] $(M+H)^+$ *calcd*. 321.1677, *found* 321.1683.

**Halogenation**

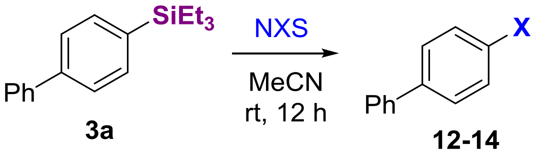


**General procedure D for halogeneration of 3a**

To the solution of 3a (56.5 mg, 0.2 mmol) in dried MeCN (1ml) was added NXS (0.5 mmol, 5 equiv) under N2 protected. The mixture was stirred at room temperature for 12 h. Then, the mixture was concentrated and purified by column chromatography silica gel (hexane) to give the aryl halide.

**4-Chloro-1,1'-biphenyl**

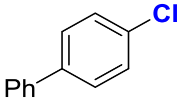


Following general procedure **D**. Purification by column chromatography on silica gel (hexane) afforded **12** as white solid (26.5 mg, 70% yield).
$^1$H NMR (300 MHz, $CDCl_3$) δ 7.59 – 7.49 (m, 4H), 7.48 – 7.34 (m, 5H).
The chemical shifts were consistent with those reported in the literature.[26]

**4-Bromo-1,1'-biphenyl**

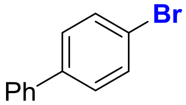


Following general procedure **D**. Purification by column chromatography on silica gel (hexane) afforded **13** as white solid (43.4 mg, 93% yield).
$^1$H NMR (300 MHz, $CDCl_3$) δ 7.60 – 7.53 (m, 4H), 7.45 (dd, $J$ = 8.6, 7.0 Hz, 4H), 7.40 – 7.33 (m, 1H).
The chemical shifts were consistent with those reported in the literature.[27]

**4-Iodo-1,1'-biphenyl**

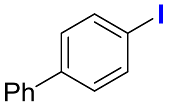


Following general procedure **D**. Purification by column chromatography on silica gel (hexane) afforded **14** as white solid (50.0 mg, 90% yield).
$^1$H NMR (300 MHz, $CDCl_3$) δ 7.81 – 7.73 (m, 2H), 7.59 – 7.51 (m, 2H), 7.49 – 7.41 (m, 2H), 7.40 – 7.30 (m, 3H).

The chemical shifts were consistent with those reported in the literature.[27]

**Desilylative acyloxylation**

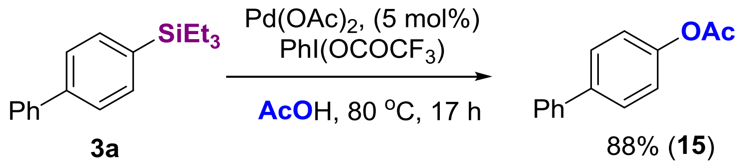


To the solution of 3a (56.6 mg, 0.2 mmol) in AcOH (0.5 ml) was added PhI(OCOCF3)2 (129.0 mg, 0.3 mmol) and Pd(OAc)2 (2.2 mg, 0.01 mmol). The mixture was stirred at 80 °C for 17 h, and poured into aquous NaHCO3 solution and extract with DCM (3× 5 ml). The combined organic extract was dried over anhydrous MgSO4 and concentrated. The resident was purified by column chromatography on silica gel (hexane/EtOAc = 6/1) to give **15** as white solid (37.1 mg, yield 88%).
$^{1}$H NMR (300 MHz, $CDCl_3$) δ 7.63 – 7.52 (m, 4H), 7.48 – 7.41 (m, 2H), 7.39 – 7.32 (m, 1H), 7.20 – 7.13 (m, 2H), 2.34 (s, 3H).
The chemical shifts were consistent with those reported in the literature.[28]

**Sequential protocol for C(sp3)-F carboxylation with $CO_2$.**

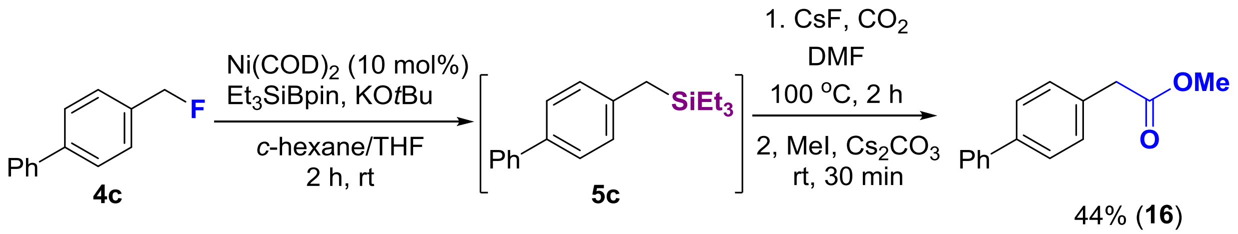


First, following general procedure **C** to prepare **5c**, **4c** (37.2 mg, 0.2 mmol), $Et_3SiBpin$ (72.6 mg, 0.3 mmol), KO*t*Bu (56.1 mg, 0.5 mmol). After defluorosilylation, the mixture of reaction was concentrated under a reduced pressure. Then, the resident was dissolved in DMF (1 ml), evacuated and backfilled with $CO_2$ (×3). CsF (90 mg, 0.6 mmol) was added. The mixture was stirred at 100 °C for 1 h under 1 atm of $CO_2$ (balloon). Cooling to room temperature, Cs2CO3 (130 mg, 0.4 mmol) and MeI (25 μl, 0.4 mmol) were added. After 30 min, water (2 ml) was added and the mixture was extracted with EtOAc (3× 5 ml). The combined organic layer was concentrated and purified by column chromatography on silica gel (hexane/EtOAc = 6/1) to give ester 16 as colorless liquid (20.0 mg, 44%).
$^{1}$H NMR (300 MHz, $CDCl_3$) δ 7.57 (t, $J$ = 7.2 Hz, 5H), 7.44 (t, $J$ = 7.4 Hz, 2H), 7.36 (d, $J$ = 7.8 Hz, 3H), 3.72 (s, 3H), 3.68 (s, 2H).
The chemical shifts were consistent with those reported in the literature.[29]

## Supplementary Table

**Supplementary Table 1. Optimization of ligand.**

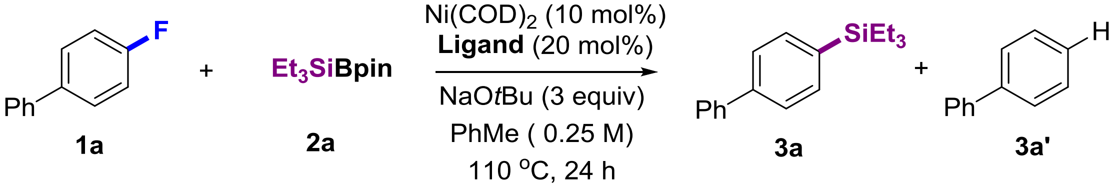


| Entry | Ligand | Conversion 1a (%) | 3a (%) | 3a′(%) |
|---|---|---|---|---|
| 1 | IPr·HCl | 93 | 50 | 33 |
| 2 | IMes·HCl | 96 | 30 | 58 |
| 3 | SIPr·HCl | 86 | 31 | 33 |
| 4 | IPr | 75 | 46 | 25 |
| 5* | IPr·HCl | 87 | 55 | 31 |
| 6 | $PCy_3$ | 65 | 37 | 17 |
| 7 | $Ph_3P$ | 40 | 19 | 16 |
| 8 | *t*$Bu_3P$ | 60 | 35 | 19 |
| 9 | dppe | 9 | 1 | 0 |
| 10 | dcpe | 28 | 15 | 0 |
| 11 | Xantphos | 35 | 43 | 0 |
| 12 | bpy | 60 | 11 | 15 |
| 13 | dtbpy | 69 | 16 | 10 |
| 14† | IPr·HCl | 75 | 47 | 20 |
| 15†† | No Ligand | 98 | 56 | 36 |

Reaction condition: **1a** (0.2 mmol), **2a** (1.5 equiv), $Ni(COD)_2$ (10 mol%), ligand (20 mol%), NaO*t*Bu (3 equiv) in toluene (0.8 mL), 110 °C, 24 h. GC conversion and yields using decane as internal standard. *160 °C. †K*t*OBu (3.0 equiv), ††K*t*OBu (3.0 equiv), 80 °C.

**Supplementary Table 2. Optimization of additive.**

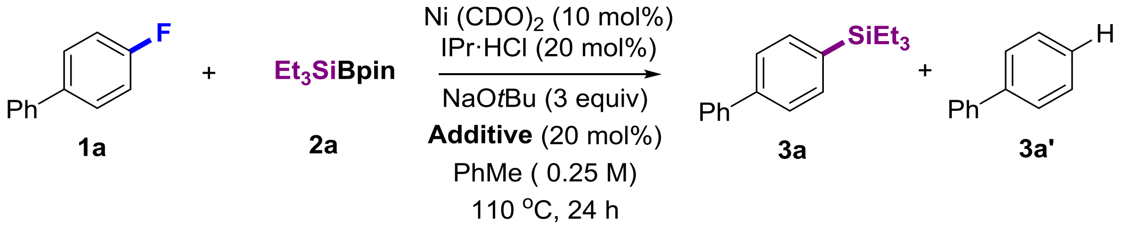


| Entry | Additive | Conversion 1a (%) | 3a (%) | 3a′(%) |
|---|---|---|---|---|
| 1 | $CuF_2$ | 57 | 15 | 41 |
| 2 | CuI | 16 | 6 | 4 |
| 3 | CuCl | 25 | 17 | 4 |
| 4 | LiCl | 0 | 0 | 0 |
| 5 | NaCl | 0 | 0 | 0 |
| 6 | TMAF | 0 | 0 | 0 |
| 7 | $Ag_2CO_3$ | 88 | 46 | 40 |

Reaction condition: **1a** (0.2 mmol), **2a** (1.5 equiv), $Ni(COD)_2$ (10 mol%), IPr·HCl (20 mol%), NaO*t*Bu (3 equiv), additive (20 mol%) in toluene (0.8 mL), 110 °C, 24 h. GC conversion and yields using decane as internal standard.

**Supplementary Table 3. Optimization of solvent.**

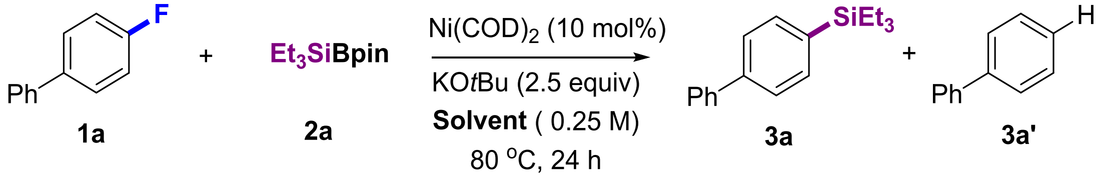


| Entry | Solvent | Conversion 1a (%) | 3a (%) | 3a′(%) |
|---|---|---|---|---|
| 1* | toluene | 98 | 56 | 36 |
| 2 | benzene | 95 | 55 | 32 |
| 3 | 1,4-dioxane | 0 | 0 | 0 |
| 4 | DMF | 0 | 0 | 0 |
| 5 | *c*-hexane | 100 | 74 | 18 |
| 6† | *c*-hexane | 98 | 56 | 38 |
| 7†† | *c*-hexane | 97 | 70 | 19 |
| 8 | THF | 100 | 55 | 14 |
| 9 | toluene/*c*-hexane (1/1) | 72 | 20 | 28 |
| 10 | *c*-hexane/THF 15/1 | 100 | 66 | 24 |
| 11 | *c*-hexane/THF 9/1 | 100 | 74 | 15 |
| 12 | *c*-hexane/THF 4/1 | 100 | 74 | 19 |
| 13 | *c*-hexane/THF 1/1 | 100 | 85 | 14 |
| 14 | *c*-hexane/THF 1/2 | 100 | 89 | 8 |
| 15 | *c*-hexane/THF 1/4 | 97 | 59 | 18 |

Reaction condition: **1a** (0.2 mmol), **2a** (1.5 equiv), $Ni(COD)_2$ (10 mol%), KO*t*Bu (2.5 equiv) in solvent (0.8 mL), 80 °C, 24 h. GC conversion and yields using decane as internal standard. *KO*t*Bu (3.0 equiv), 110 °C. †**2a** (1.3 equiv). ††LiI (1 equiv) as additive.

**Supplementary Table 4. Optimization of base.**

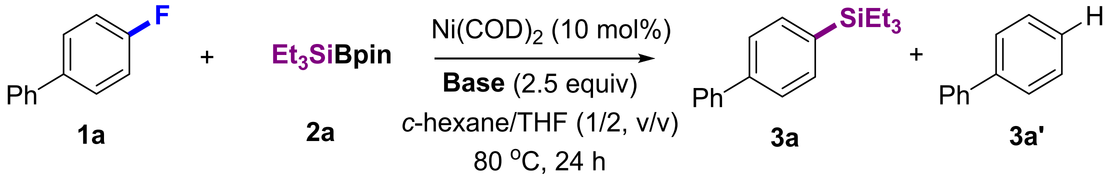


| Entry | Base | Conversion 1a (%) | 3a (%) | 3a′(%) |
|---|---|---|---|---|
| 1 | KO*t*Bu | 100 | 89 | 8 |
| 2* | KO*t*Bu | 86 | 59 | 24 |
| 3† | KO*t*Bu | 100 | 88 | 8 |
| 4 | NaO*t*Bu | 100 | 71 | 23 |
| 5 | $K_2CO_3$ | 0 | 0 | 0 |
| 6 | KOMe | 76 | 60 | 10 |
| 7 | $K_3PO_4$ | 0 | 0 | 0 |
| 8 | KF | 0 | 0 | 0 |
| 9 | KHMDS | 98 | 56 | 24 |

Reaction condition: **1a** (0.2 mmol), **2a** (1.5 equiv), Ni(COD)$_2$ (10 mol%), KO*t*Bu (2.5 equiv) in solvent (0.8 mL), 80 °C, 24 h. GC conversion and yields using decane as internal standard. *KO*t*Bu (2 equiv). †KO*t*Bu (3 equiv).

**Supplementary Table 5. Optimization of metal.**

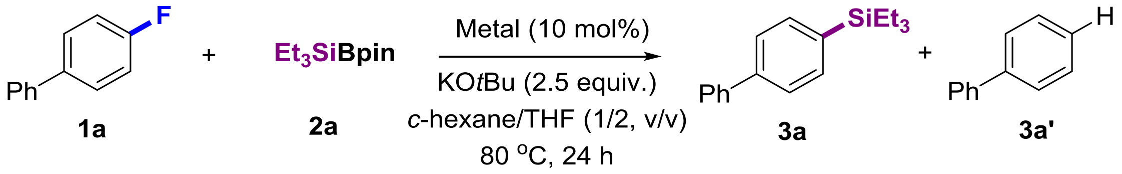


| Entry | Metal | Conversion 1a (%) | 3a (%) | 3a′(%) |
|---|---|---|---|---|
| 1 | Ni(COD)$_2$ | 100 | 89 | 8 |
| 2 | Ni(PPh$_3$)$_2$Cl$_2$ | 36 | 20 | 11 |
| 3 | Ni(acac)$_2$ | 48 | 22 | 25 |
| 4 | NiCl$_2$ | 59 | 23 | 26 |
| 5 | CuCl | 56 | 26 | 17 |
| 6 | Cu(OAc) | 43 | 21 | 16 |
| 7 | Cu(OAc)$_2$ | 61 | 24 | 20 |
| 8* | Ni(COD)$_2$ | 98 | 68 | 22 |

Reaction condition: **1a** (0.2 mmol), **2a** (1.5 equiv), Ni(COD)$_2$ (10 mol%), KO*t*Bu (2.5 equiv) in solvent (0.8 mL), 80 °C, 24 h. GC conversion and yields using decane as internal standard. *Ni(COD)$_2$ (5 mol%).

**Supplementary Table 6. Optimization of temperature.**

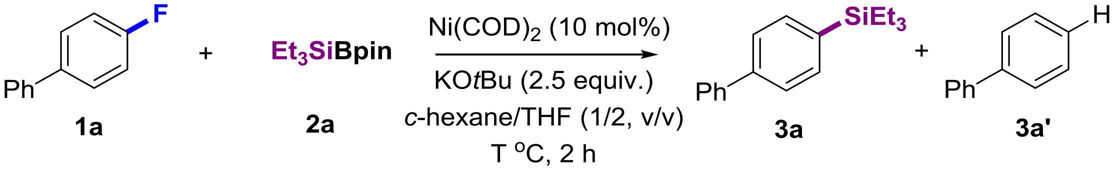


| Entry | T ( °C) | Conversion 1a (%) | 3a (%) | 3a′(%) |
|---|---|---|---|---|
| 1 | 25 | 100 | 89 | 5 |
| 2 | 50 | 100 | 85 | 13 |
| 3 | 80 | 100 | 85 | 12 |

Reaction condition: **1a** (0.2 mmol), **2a** (1.5 equiv), Ni(COD)$_2$ (10 mol%), KO*t*Bu (2.5 equiv) in solvent (0.8 mL). GC conversion and yields using decane as internal standard.

**Supplementary Table 7. Blank experiments.**

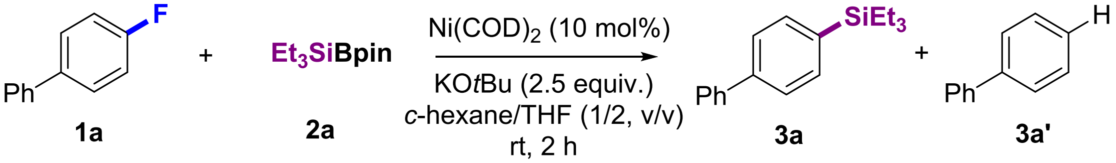


| Entry | 2a | Ni(COD)$_2$ | KO*t*Bu | Conversion 1a (%) | 3a (%) | 3a′(%) |
|---|---|---|---|---|---|---|
| 1 | √ | √ | √ | 100 | 89 | 5 |
| 2 | × | √ | √ | 0 | 0 | 0 |
| 3 | √ | × | √ | 5 | 0 | 2 |
| 4 | √ | √ | × | 0 | 0 | 0 |

Reaction condition: **1a** (0.2 mmol), **2a** (1.5 equiv), Ni(COD)$_2$ (10 mol%), KO*t*Bu (2.5 equiv) in solvent (0.8 mL). GC conversion and yields using decane as internal standard.

## Supplementary Figure

## Unsuccessful substrates for the Ni-catalyzed defluorosilylation

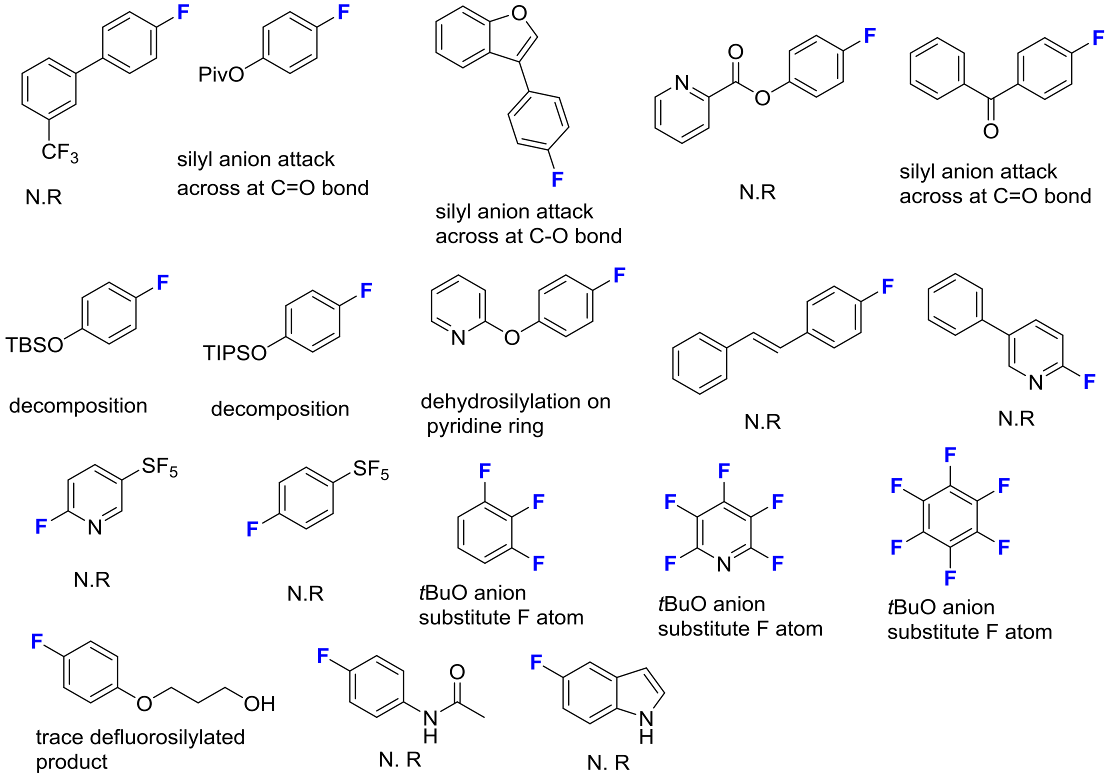


**Supplementary Figure 1**. Unsuccessful substrates

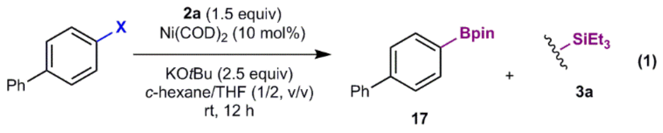


| X | Yield **3a** (%)* | **17/3a**† |
|---|---|---|
| F | 89 | none borylation |
| Cl | 10 | 70:30 |
| Br | 21 | 67:33 |
| I | 26 | 67/33 |

*Yield by GC. †The ratio was determined by GCMS

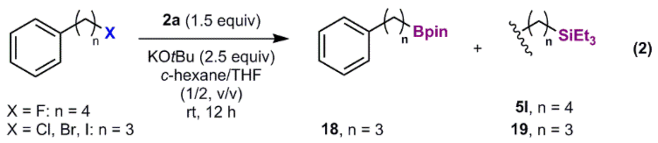


| X | Yield **5l, 18** or **19** (%)* | **18/19**† |
|---|---|---|
| F | **5l**, 70% | none borylation |
| Cl | **19**, 60% | 4:96 |
| Br | **18**, 53% | 91:9 |
| I | **18**, 18% | 81:19 |

*Isolated yield. †The ratio was determined by GCMS

**Supplementary Figure 2.** Competition between silylation and borylation of halogen-containing compounds

**Equation 1.**

Following the general procedure **B**, using aryl halide (0.2 mmol) and **2a** (72.6 mg, 0.3 mmol) for 12 h; and the mixture tube was added $Et_2O$ (20 ml), saturated aqueous ammonium chloride (1 ml), followed by *n*-decane (10 μl, 0.05 mmol) as an internal standard. After stirring the mixture vigorously, GC and GCMS analysis were conducted using a portion of the resulting organic phase.

**Equation 2.**
Following the general procedure **C**, using alkyl halide (0.2 mmol) and **2a** (72.6 mg, 0.3 mmol) for 12 h. The mixture was analyzed by GCMS to determine the ratio of silylation and borylation products. Then, to the mixture was added saturated aqueous ammonium chloride (1 ml) and extracted by $Et_2O$ (3 × 5 ml). The organic phase was concentrated under reduced pressure. The residue was purified by PLC (hexane) to give corresponding silylated or borylated product.

**4,4,5,5-Tetramethyl-2-(3-phenylpropyl)-1,3,2-dioxaborolane (18)**

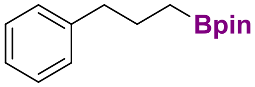


Purification by PLC (hexane) afforded **18** as colorless liquid (26.0 mg, 53% yield).
$^1$H NMR (300 MHz, $CDCl_3$) δ 7.30 – 7.23 (m, 2H), 7.22 – 7.12 (m, 3H), 2.68 – 2.54 (m, 2H), 1.73 (dt, *J* = 15.8, 7.9 Hz, 2H), 1.24 (s, 12H), 0.88 – 0.76 (m, 2H).
$^{13}$C NMR (126 MHz, $CDCl_3$) δ 142.84, 128.70, 128.31, 125.70, 83.08, 77.41, 77.16, 76.91, 38.74, 26.27, 24.97.
IR (KBr) 2952, 2875, 1594, 1502, 1461, 1276, 1247, 1182, 1110, 1016, 809, 734.
HRMS (EI) [$C_{15}H_{23}BO_2$] ($M^+$) *calcd*. 246.1791, *found* 246.1805.

**Triethyl(3-phenylpropyl)silane (19)**

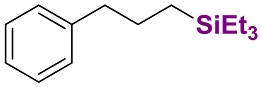


Purification by PLC (hexane) afforded **19** as colorless liquid (28.0 mg, 60% yield).
$^1$H NMR (300 MHz, $CDCl_3$) δ 7.32 – 7.25 (m, 2H), 7.22 – 7.15 (m, 3H), 2.62 (t, *J* = 7.7 Hz, 2H), 1.70 – 1.57 (m, 2H), 0.91 (t, *J* = 7.9 Hz, 10H), 0.62 – 0.42 (m, 9H).
The chemical shifts were consistent with those reported in the literature.[30]

**Studies of the reactivity of C-X bonds**

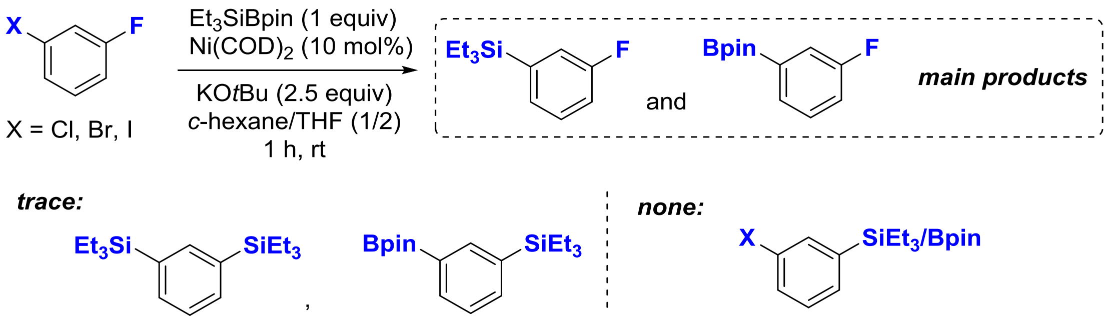


**Supplementary Figure 3.** The reactivity of the C-F bond compared the C-Cl, C-Br, and C-I bond, in 1,3-disubstituted benzene derivatives.

Following the general procedure B, $Et_3SiBpin$ (1 equiv), reaction time 1 h. After 1 h, the mixture was detected by GCMS. The results were as shown in Fig. S2, C-Cl, C-Br and C-I bond is prior to C-F bond.

## Preliminary mechanistic investigations

## Radical clock experiments with 20

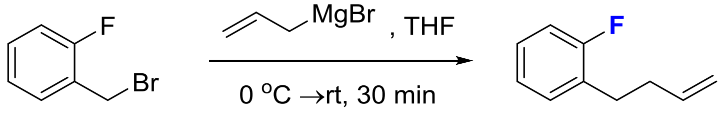


To the solution of 1-(bromomethyl)-2-fluorobenzene (0.61 ml, 5.0 mmol) in THF (7 ml) was dropwise added allymagnesium bromide (10.7 ml, 7.5 mmol, 0.7 M) at 0 °C. The reaction mixture was stirred for 30 min after warming to room temperature. To this was added saturated aqueous ammonium chloride at 0 °C. The mixture was extracted with $Et_2O$ (3 × 5 ml), washed with brine (5 ml) and dried over $MgSO_4$. After filtration, the filtrate was concentrated under reduced pressure. The residue was purified by column chromatography on silica gel (hexane) to give **20** as colorless liquid (0.63 g, 84%).

$^1$H NMR (300 MHz, $CDCl_3$) δ 7.17 (dd, *J* = 13.6, 7.1 Hz, 2H), 7.10 – 6.95 (m, 2H), 5.86 (ddt, *J* = 16.8, 10.0, 6.6 Hz, 1H), 5.11 – 4.92 (m, 2H), 2.74 (t, *J* = 7.8 Hz, 2H), 2.37 (dd, *J* = 15.0, 7.2 Hz, 2H).

$^{19}$F NMR (282 MHz, $CDCl_3$) δ -118.79 – -119.02 (m).

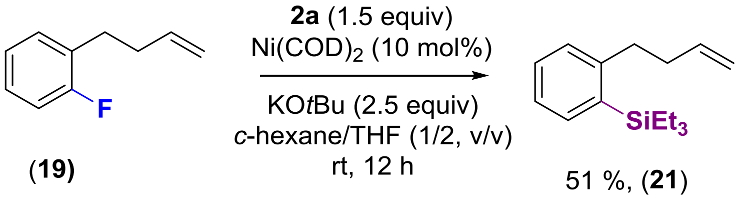


Following the general procedure **B**, using 12 (30.0 mg, 0.2 mmol) and 2a (72.6 mg, 0.3 mmol) for 12 h. The mixture was analyzed by GCMS to determine the products. Then, the mixture was added saturated aqueous ammonium chloride (1 ml) and extracted with $Et_2O$ (3 × 5 ml). The combined organic phase was dried over $MgSO_4$ and filtrated. The filtration was concentrated under reduced pressure. The residue was purified by PLC (hexane) to give **21** as colorless liquid (25.0 mg, 51%).

$^1$H NMR (300 MHz, $CDCl_3$) δ 7.43 (d, *J* = 7.2 Hz, 1H), 7.36 – 7.27 (m, 1H), 7.25 – 7.12 (m, 2H), 6.05 – 5.81 (m, 1H), 5.05 (dd, *J* = 22.6, 13.7 Hz, 2H), 2.90 – 2.70 (m, 2H), 2.35 (dd, *J* = 15.7, 6.9 Hz, 2H), 0.95 (t, *J* = 6.9 Hz, 9H), 0.90 – 0.76 (m, 6H).

$^{13}$C NMR (126 MHz, $CDCl_3$) δ 148.23, 138.38, 135.79, 135.18, 129.13, 128.61, 125.15, 114.88, 77.41, 77.16, 76.91, 36.43, 35.56, 7.79, 4.52.

IR (KBr) 3058, 2954, 2875, 1459, 1002, 912, 727.

HRMS (EI) (EI) [$C_{16}H_{26}Si$] ($M^+$) *calcd.* 246.1804, *found* 246.1809.

## Reaction with silicon radical scavengers

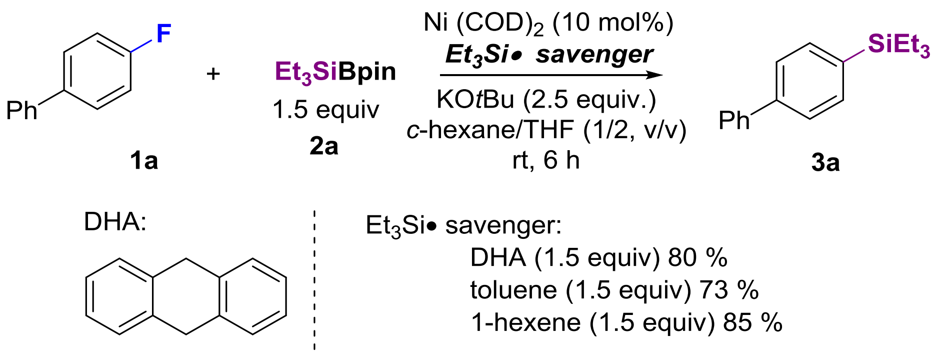


**Supplementary Figure 4.** Reaction with silicon radical scavengers.

Following the general procedure **B**, adding DHA, toluene or 1-hexane (0.3 mmol) in the glovebox and stirring the resulting mixture at room temperature for 6 h. The reaction was diluted with $Et_2O$ (20 ml) and *n*-decane (10 µl, 0.05 mmol) as an internal standard was added. The corresponding yield was calculated by GC. The yield of **2a** was not decrease almost in all the experiments, indicating the involvement of triethylsilyl radical in this reaction is unlikely.

### $^{19}$F and $^{11}$B spectroscopic studies

To a NMR tube was sequentially added a silylborane **2a** (36.3 mg, 0.15 mmol), KO*t*Bu (28.0 mg, 0.25 mmol) and *c*-hexane/THF-$d_8$ (1/2, v/v, 0.6 ml) in a glovebox filled with argon gas. The NMR tube with the mixture was sealed and removed from the glovebox, and stirred at room temperature. After 30 min, the $^{11}$B NMR analysis of the crude mixture revealed that the adduct **A** was present in the reaction mixture as shown in the following chart[31].

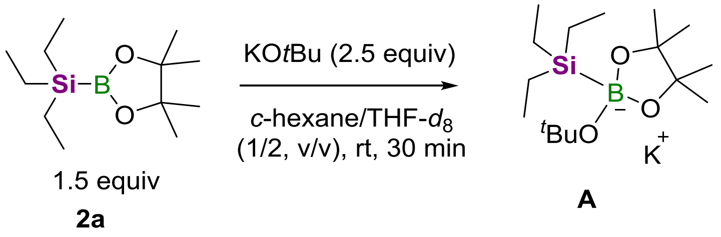


$^{11}$B{$^1$H} NMR (THF-$d_8$, 225 MHz): 3.91.

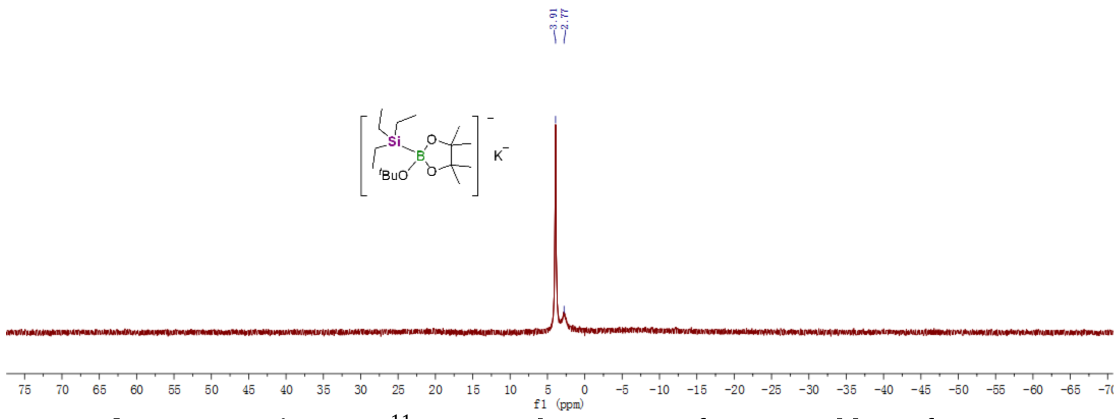


**Supplementary Figure 5.** $^{11}$B NMR observation of KO*t*Bu adduct of $Et_3SiBpin$.

**Experimental procedure for monitoring the catalytic reaction:**

To a NMR tube was sequentially added a **1** (17.2 mg, 0.10 mmol), silylborane **2a** (36.3 mg, 0.1.5 mmol), $Ni(COD)_2$ (2.7 mg, 0.01 mmol), KO*t*Bu (28.0 mg, 0.25 mmol) and *c*-hexane/THF-$d_8$ (1/2, v/v, 0.6 ml) in a glovebox filled with argon gas. The NMR tube with the mixture was sealed and removed from the glovebox, and stirred at room temperature. The reaction progress was monitored by $^{19}F$ NMR and $^{11}B$ NMR as shown in the following chart.

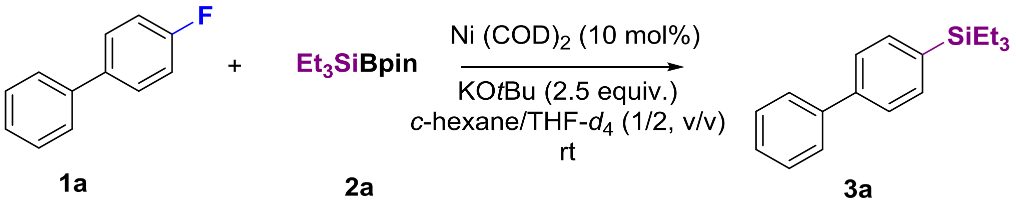


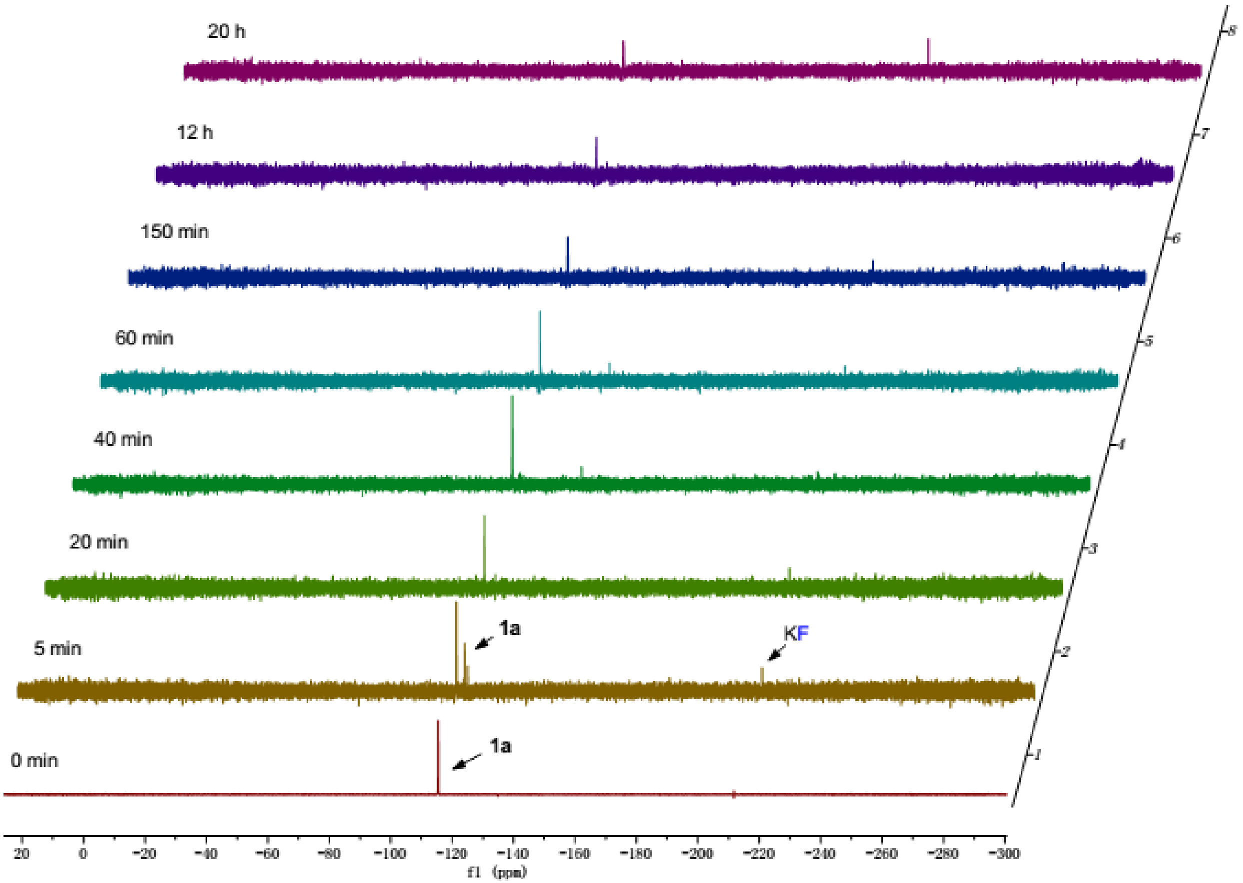

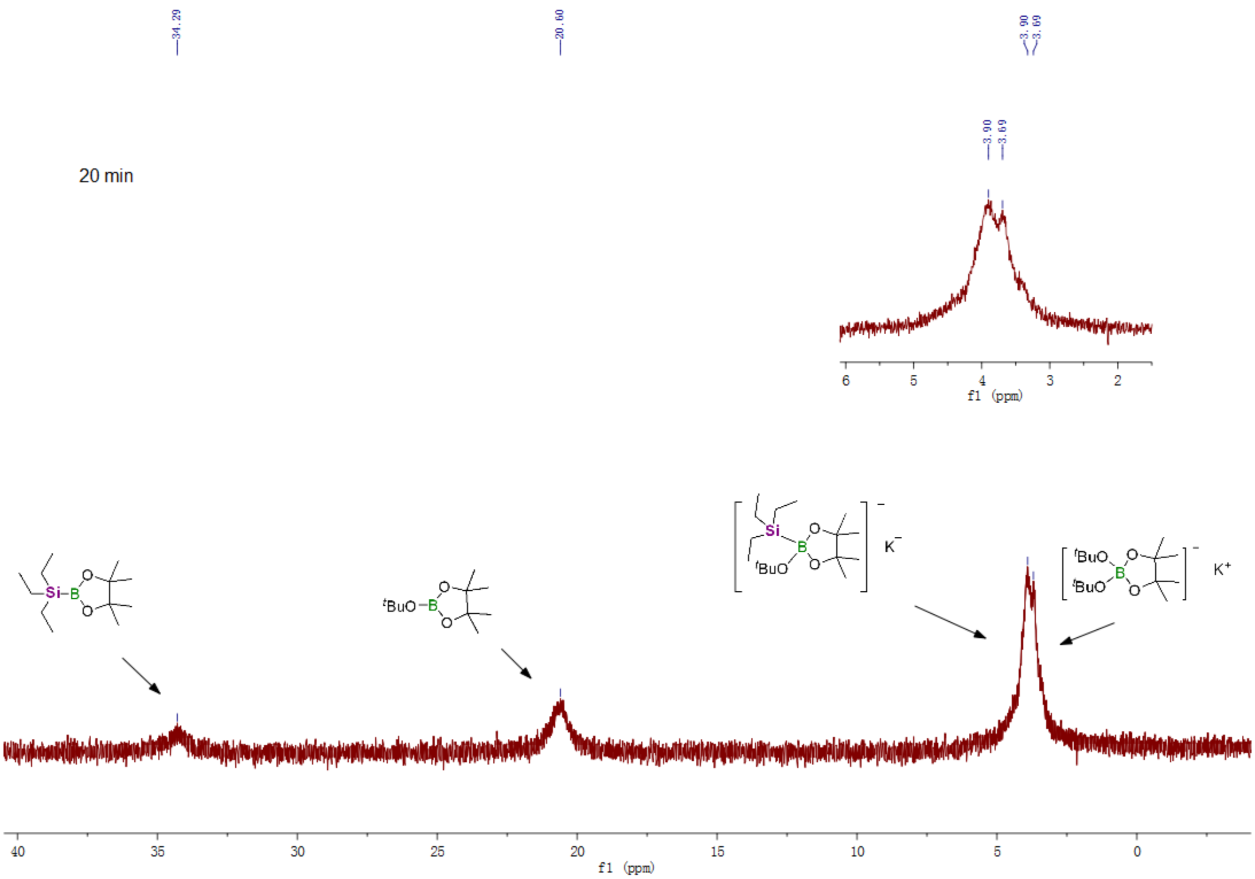


**Supplementary Figure 6.** Monitoring of the reaction of **1a** with $Et_3SiBpin$ in *c*-hexane/THF-$d_8$ by means of $^{19}F$ NMR and $^{11}B$ NMR.

It is evident from the $^{19}F$ NMR and $^{11}B$ NMR data that the reaction of **1a** with $Et_3SiBpin$ yields K[(*t*BuO)$_2$Bpin] ($^{11}B$ NMR signal at 3.69) [32, 33] and KF ($^{19}F$ NMR signal at -213.87) , aside from a small amount of uncharacterized products ($^{19}F$ NMR signal at -114.42 and -135.02).

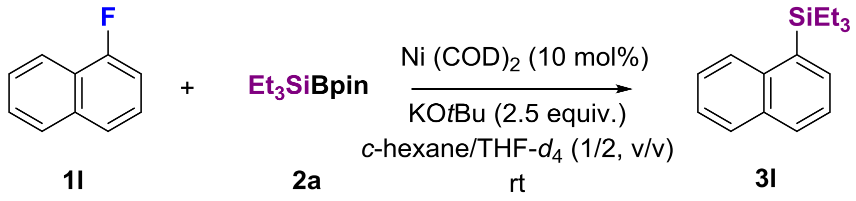


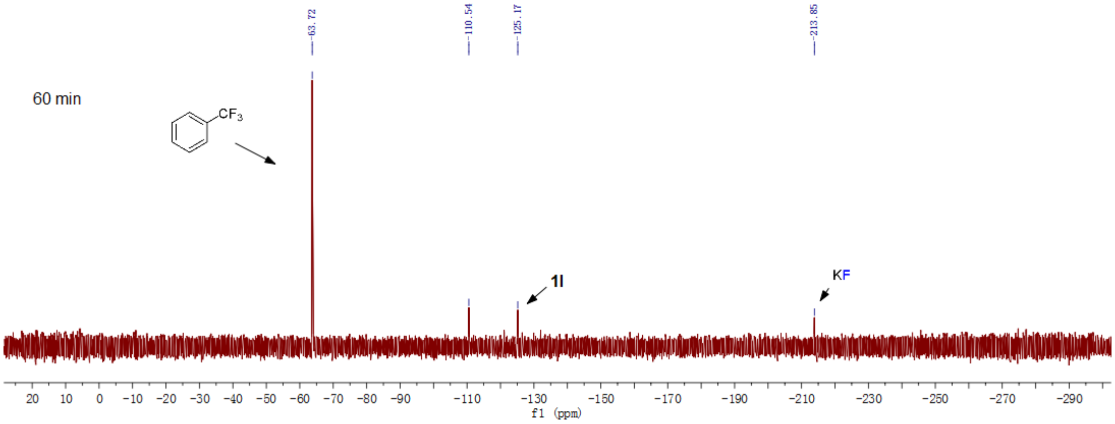


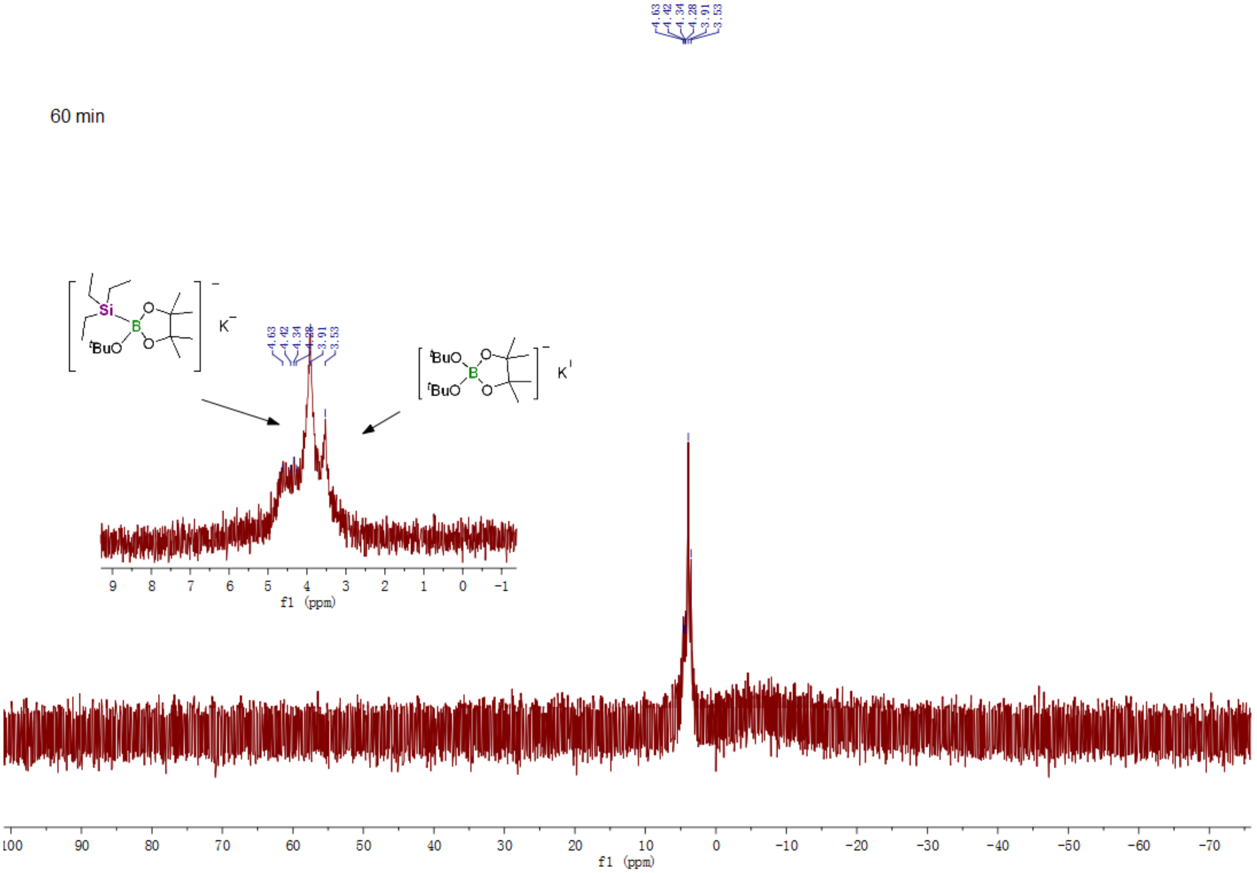


**Supplementary Figure 7.** Monitoring of the reaction of **1l** with $Et_3SiBpin$ in *c*-hexane/THF-*d*8 by means of $^{19}F$ NMR and $^{11}B$ NMR.

The reaction of π-extended **1l** with $Et_3SiBpin$ is similar as **1a** and yields K[(*t*BuO)$_2$Bpin] and KF detected in the $^{11}B$ NMR and $^{19}F$ NMR, respectively.

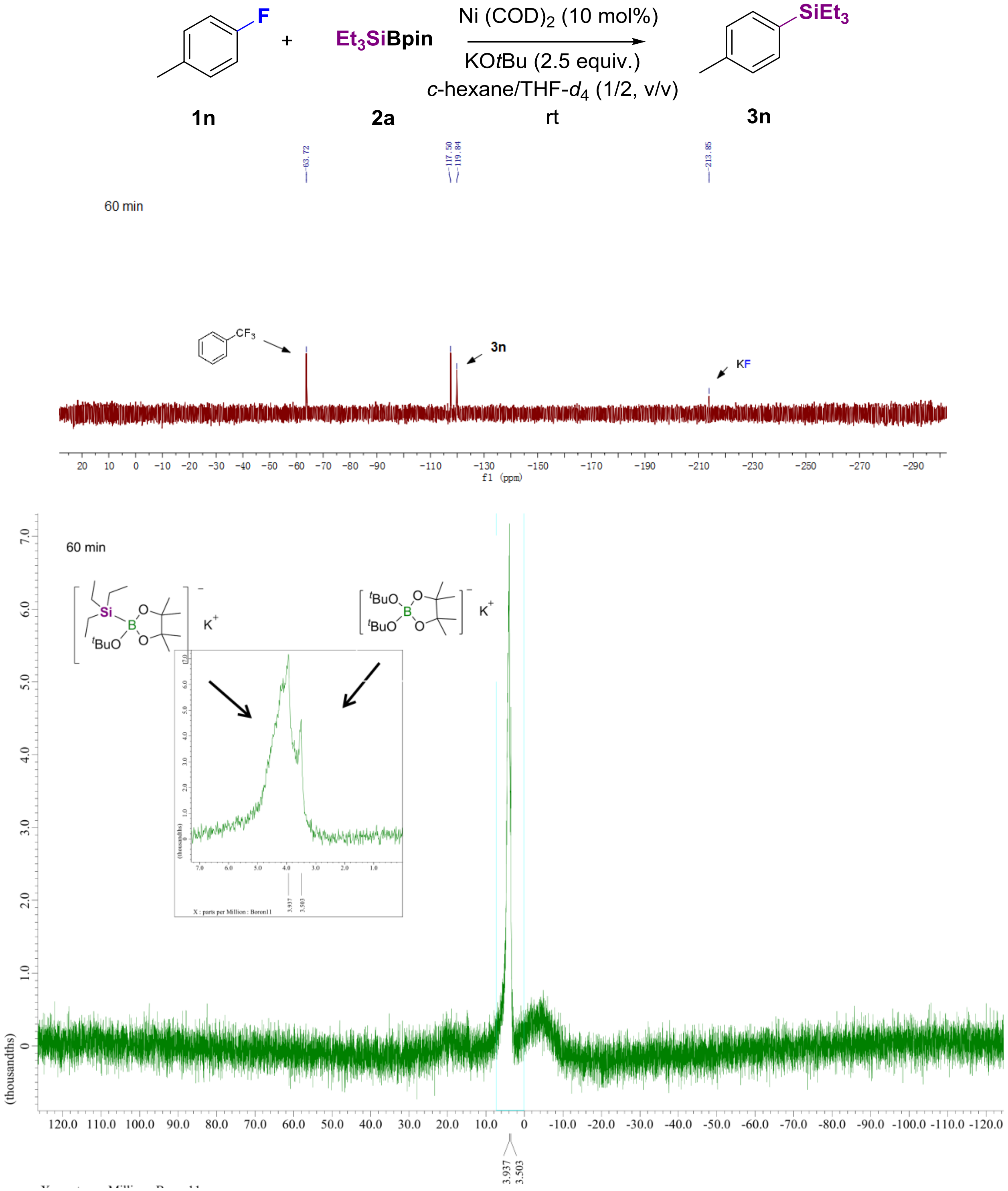


**Supplementary Figure 8.** Monitoring of the reaction of **1n** with $Et_3SiBpin$ in *c*-hexane/THF-$d_8$ by means of $^{19}F$ NMR and $^{11}B$ NMR.

While the reaction of non-π-extended **1n** with $Et_3SiBpin$ also yields K[(*t*BuO)$_2$Bpin] from $^{11}B$ NMR data, and aside from a small amount of KF as capturing F ion product from $^{19}F$ NMR.

**Map of the conventional cross-coupling mechanism**

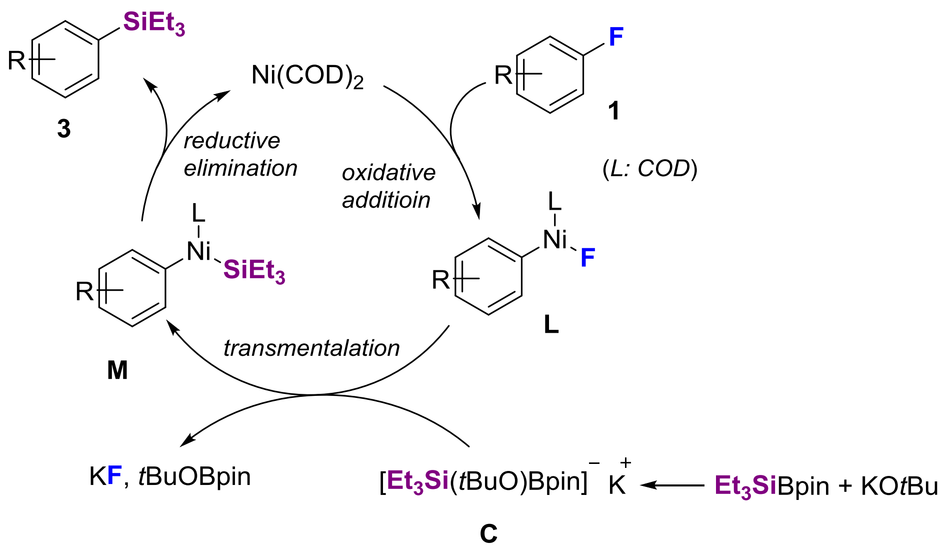


**Supplementary Figure 9.** Proposed a conventional cross-coupling mechanism in defluorosilylation reaction.

**Mechanism investigation for the reaction of 4k.**

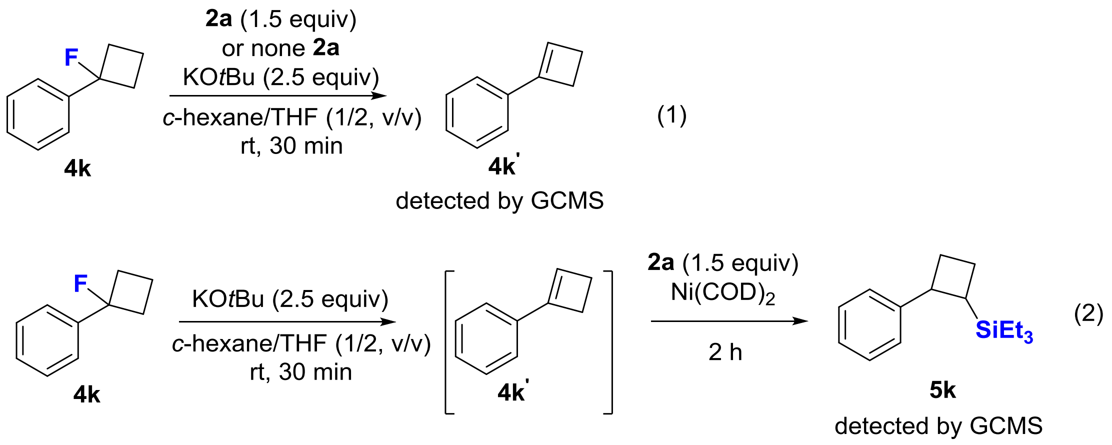


**Supplementary Figure 10**. Studies of defluorosilylation in the reaction of **4k.**

**Equation 1**

To a flame-dried screw-capped test tube were sequentially added **4k** (30.0 mg, 0.20 mmol), silylborane **2a** (72.6 mg, 0.30 mmol), KO*t*Bu (56.1 mg, 0.5 mmol) and *c*-hexane/THF (1/2, v/v, 0.8 ml) in a glovebox filled with argon gas. The tube with the mixture was sealed and removed from the glovebox, and stirred at room temperature for 30 min. The reaction progress was monitored by GCMS. After 30 min, **4k** was completely consumed, the elimination products **4k**' was detected by GCMS, no silylated products. It was the same results at the absence of **2a**.

**Equation 2**

To a flame-dried screw-capped test tube were sequentially added **4k** (30.0 mg, 0.20 mmol), KO*t*Bu (56.1 mg, 0.5 mmol) and *c*-hexane/THF (1/2, v/v, 0.8 ml) in a glovebox filled with argon gas. The sealed tube was stirred at room temperature. After 30 min, **4k** was completely converted into **4k**' (determined by GCMS). Then, **2a** (72.6 mg, 0.30 mmol) and $Ni(COD)_2$ (5.5 mg, 0.02 mmol) were added in the glovebox. The sealed tube was removed from the glovebox, and stirred for 2 h. The silylated products **5k** was detected by GCMS.

**$^{1}H$, $^{19}F$ and $^{13}C$ NMR spectra of products**

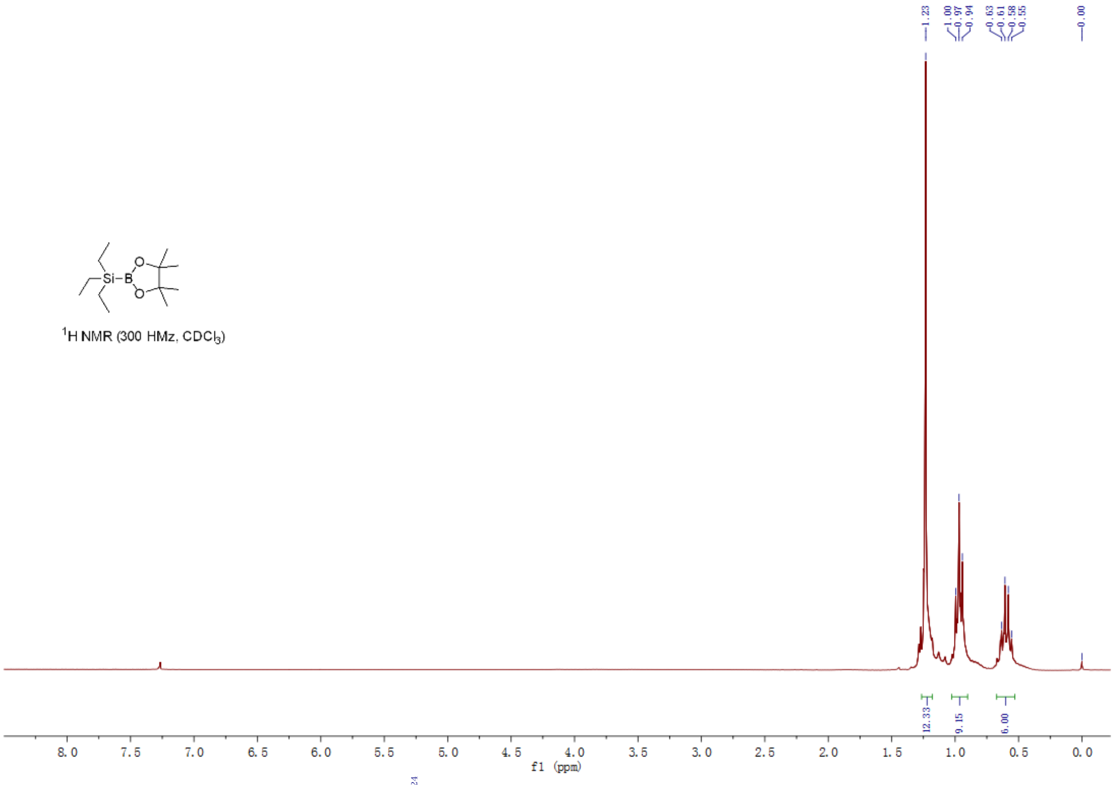


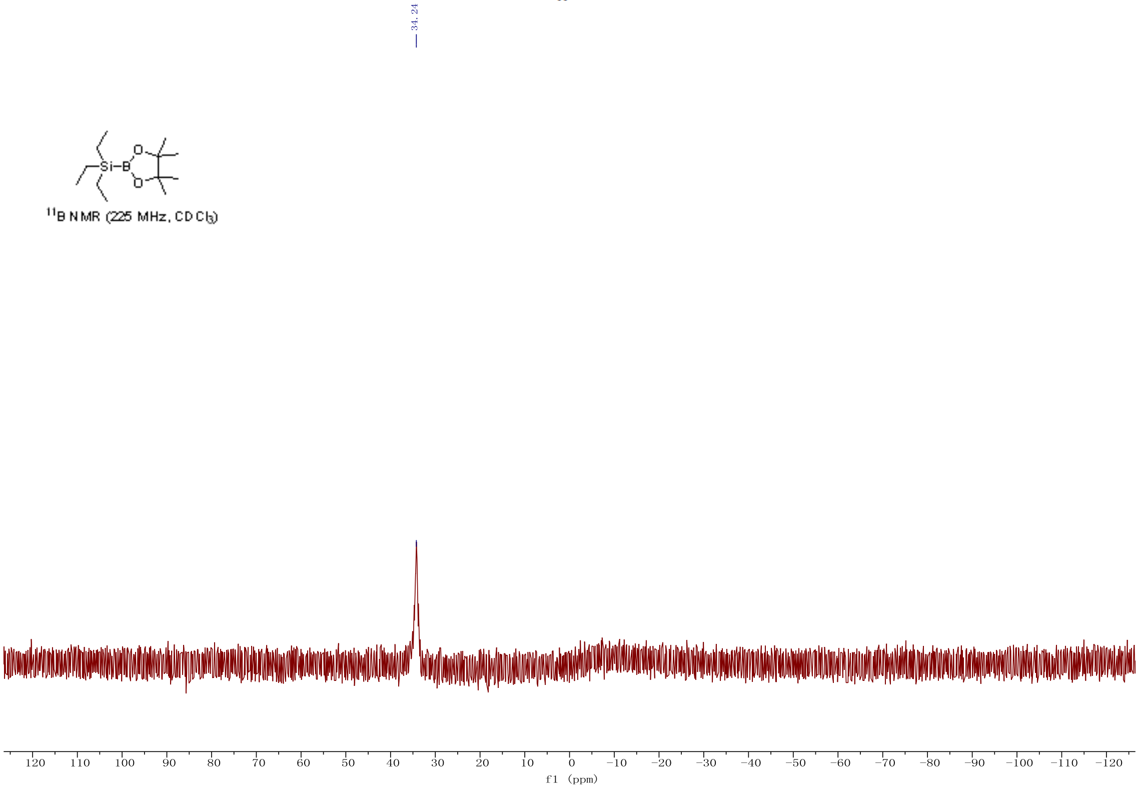


**Supplementary Figure 11**. NMR Spectra of triethyl(4,4,5,5-tetramethyl-1,3,2-dioxaborolan-2-yl)silane.

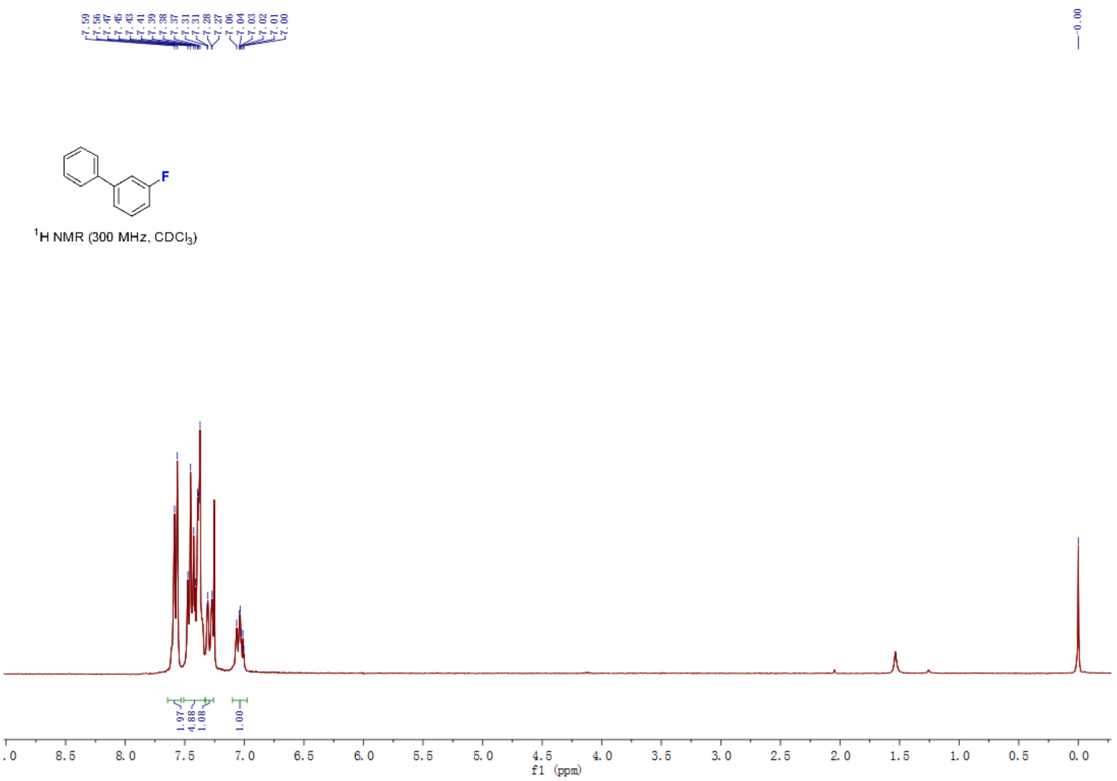
F
$^1$H NMR (300 MHz, $CDCl_3$)
f1 (ppm)

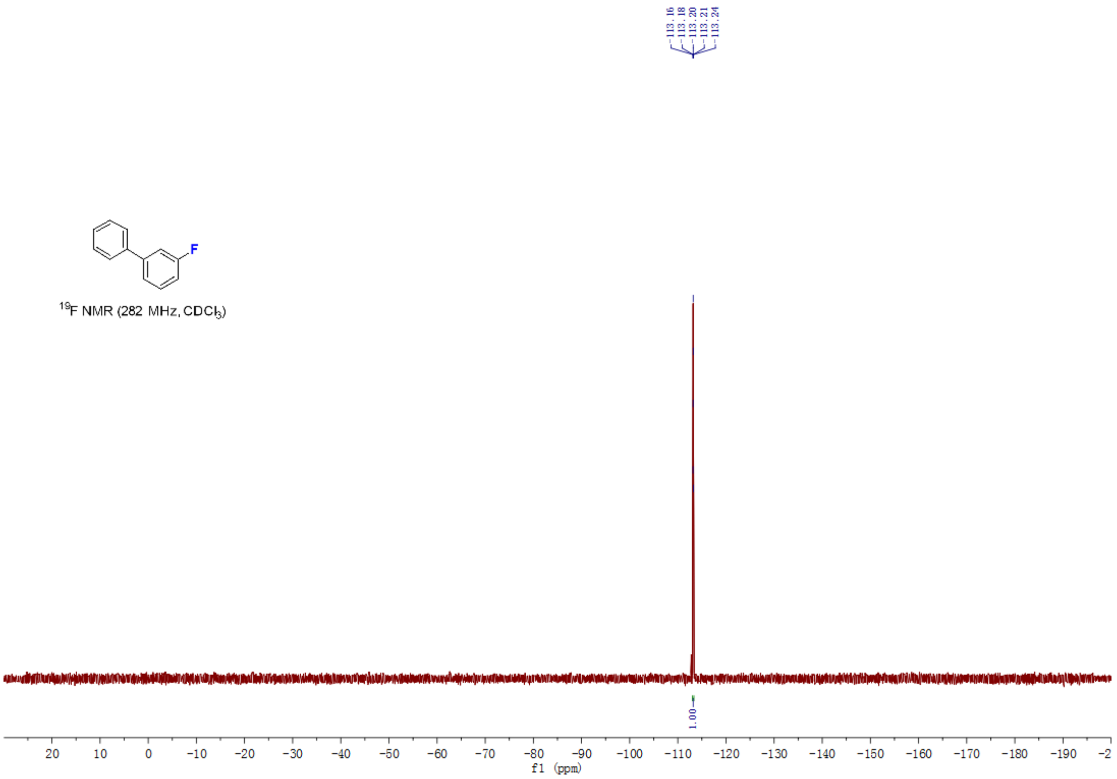
F
$^{19}$F NMR (282 MHz, $CDCl_3$)
f1 (ppm)

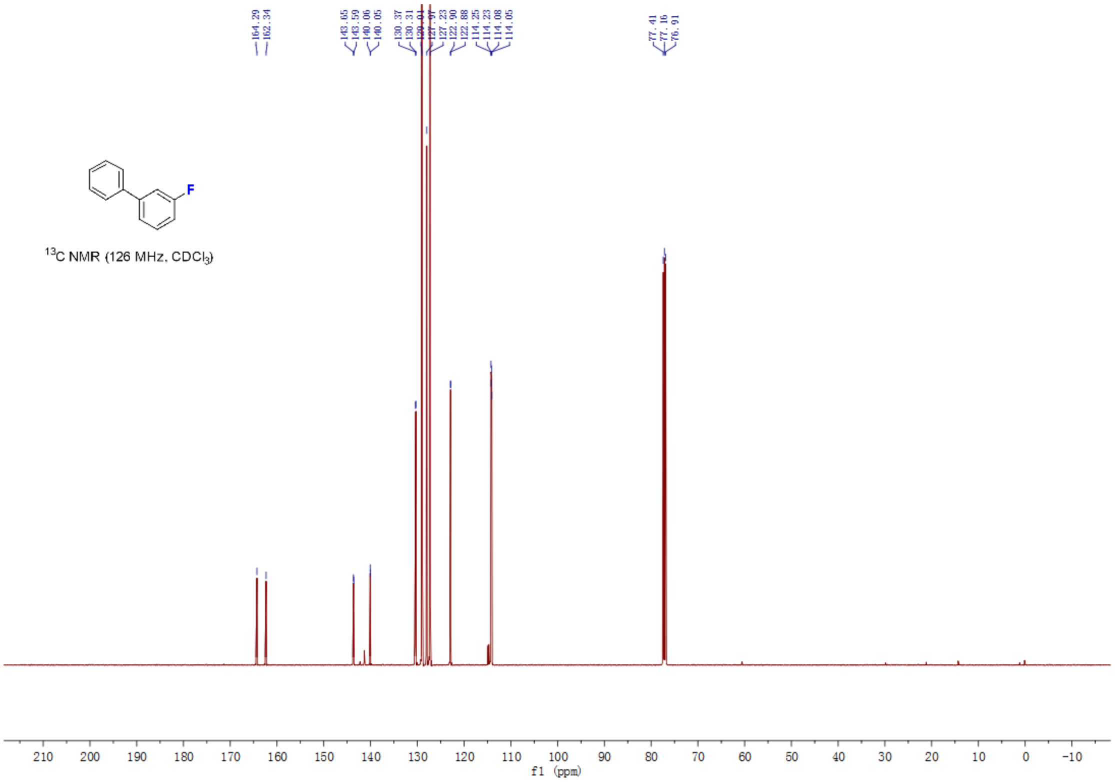


**Supplementary Figure 12**. NMR Spectra of 3-fluoro-1,1’-biphenyl

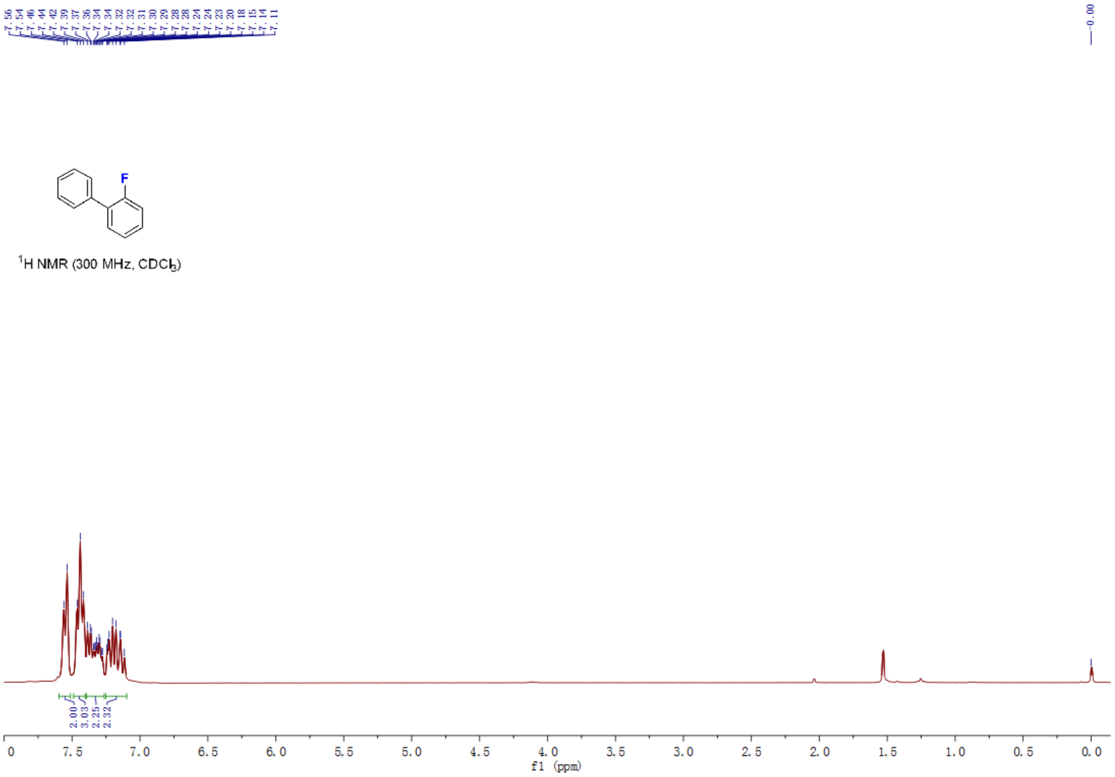


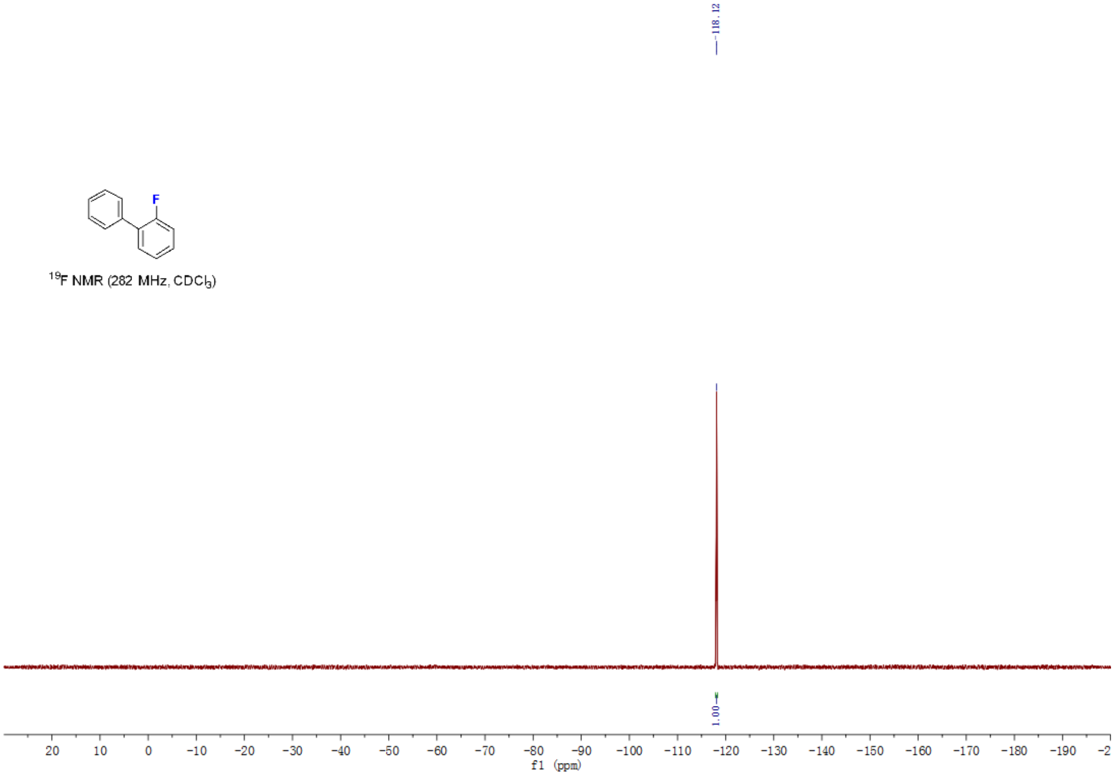


**Supplementary Figure 13**. NMR Spectra of 2-fluoro-1,1’-biphenyl

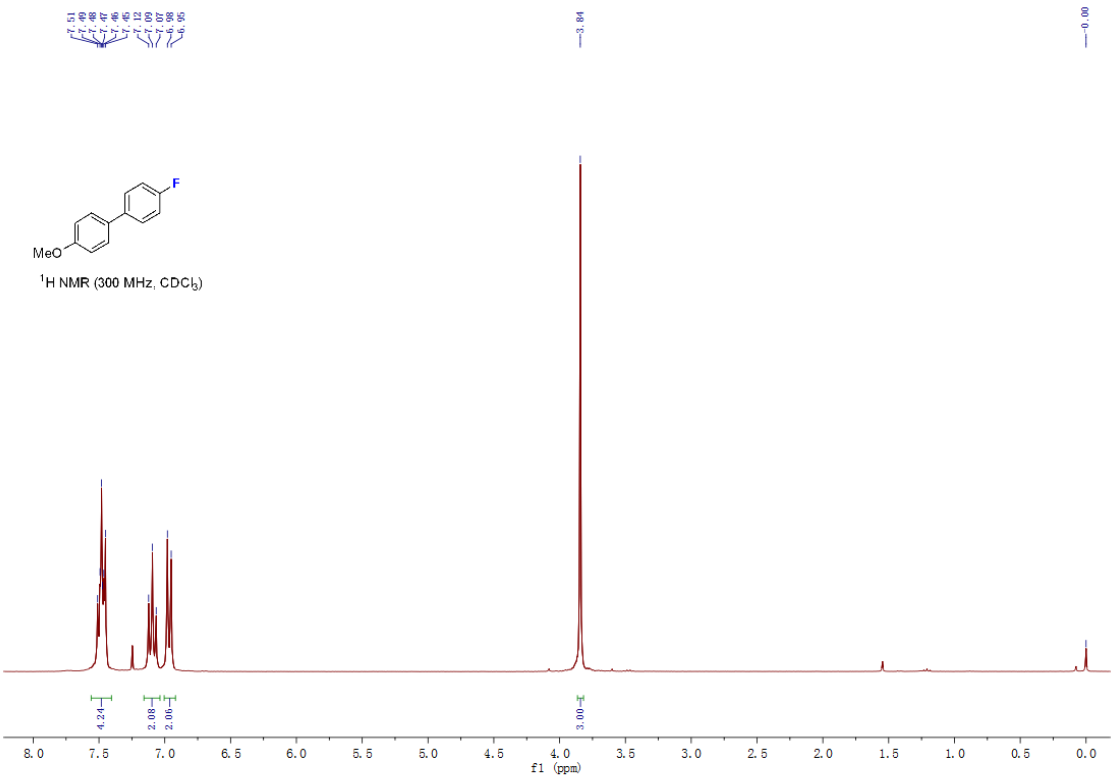


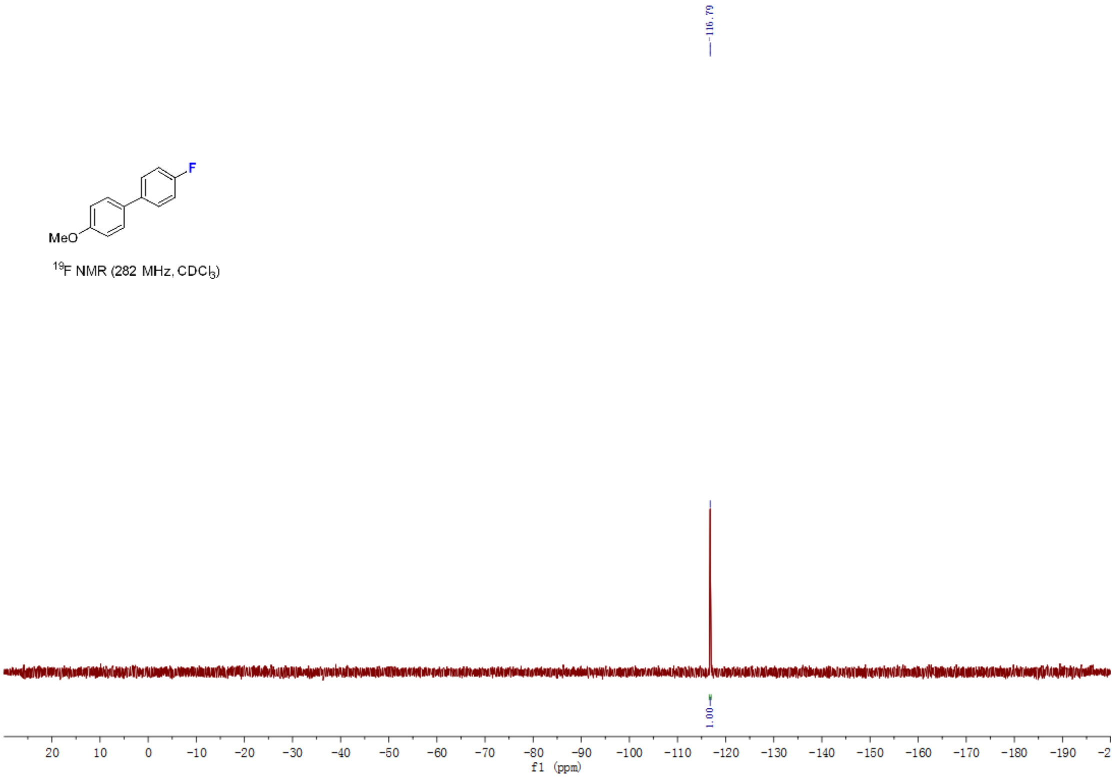


**Supplementary Figure 14**. NMR Spectra of 4-fluoro-4'-methoxy-1,1'-biphenyl.

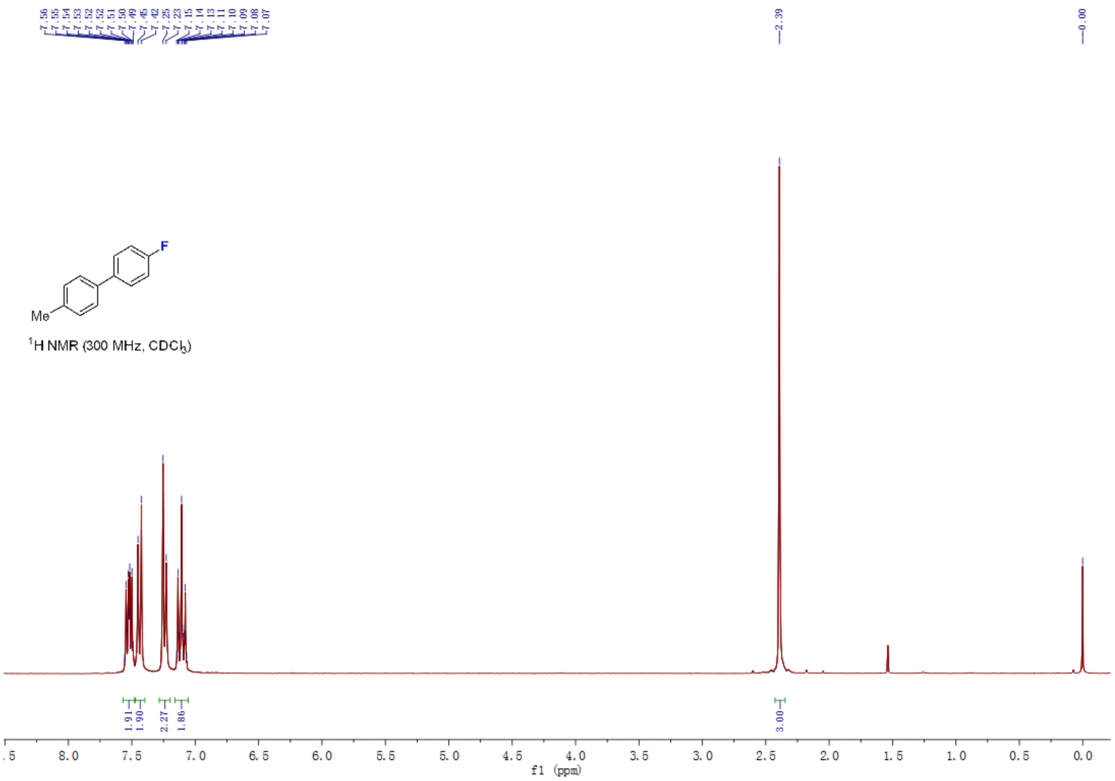


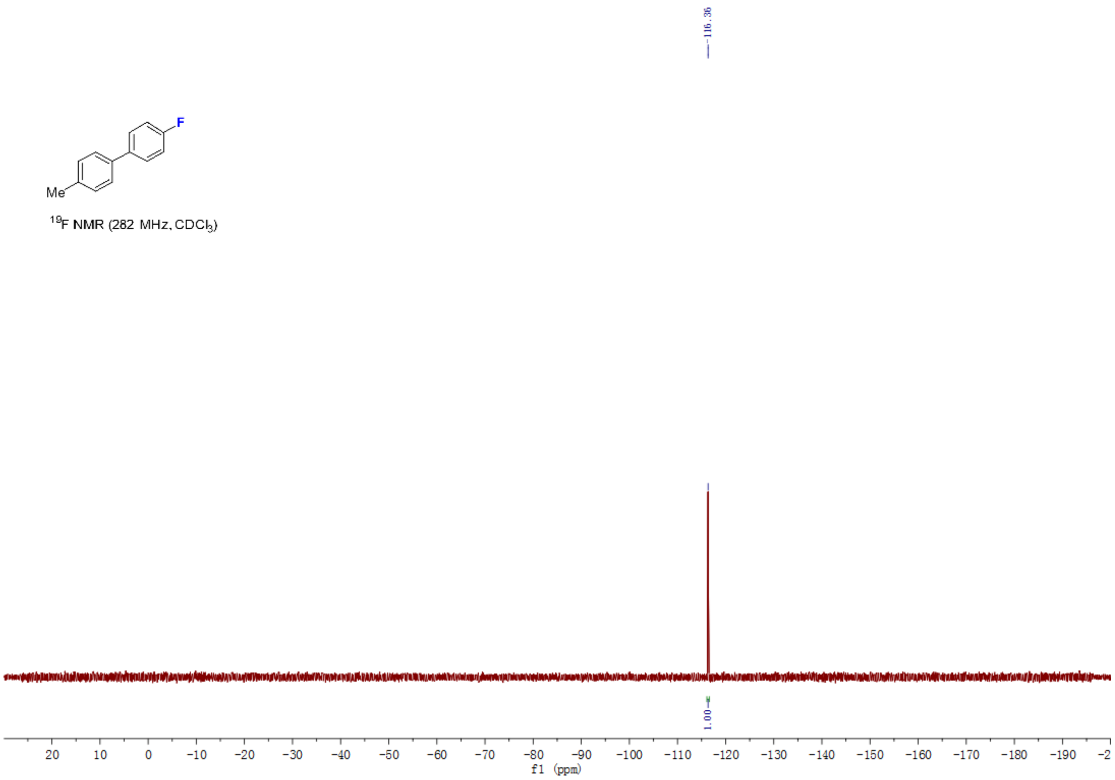


**Supplementary Figure 15**. NMR Spectra of 4-fluoro-4'-methyl-1,1'-biphenyl.

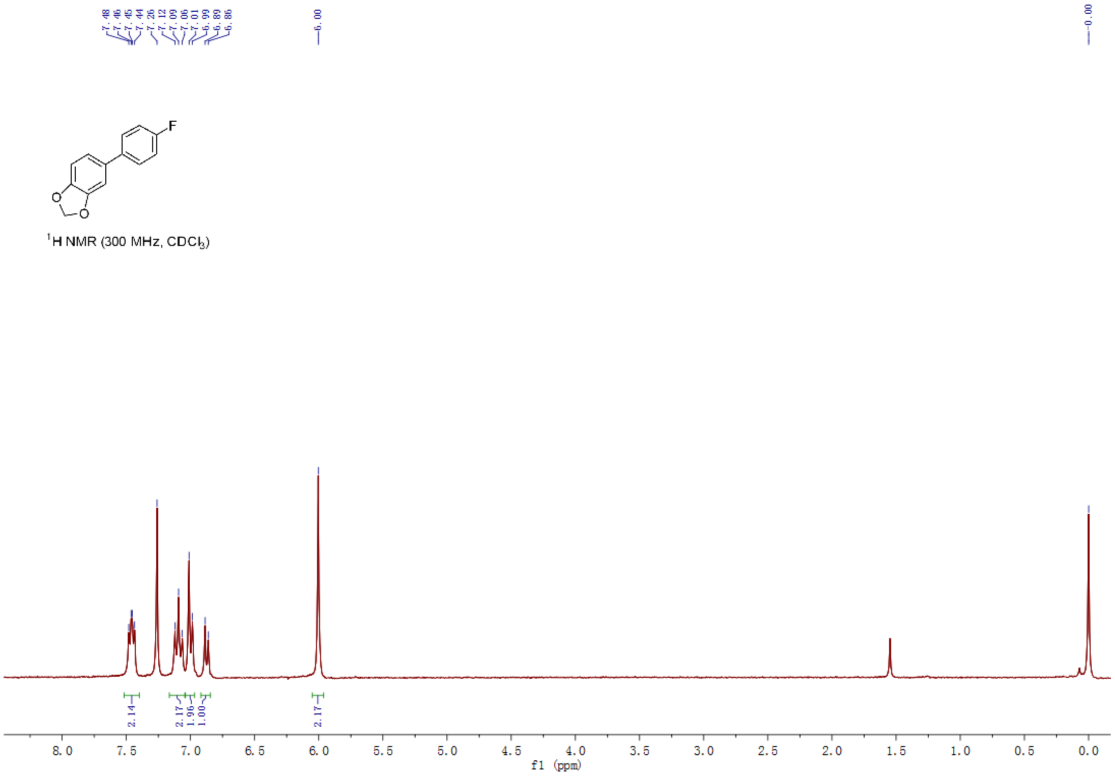


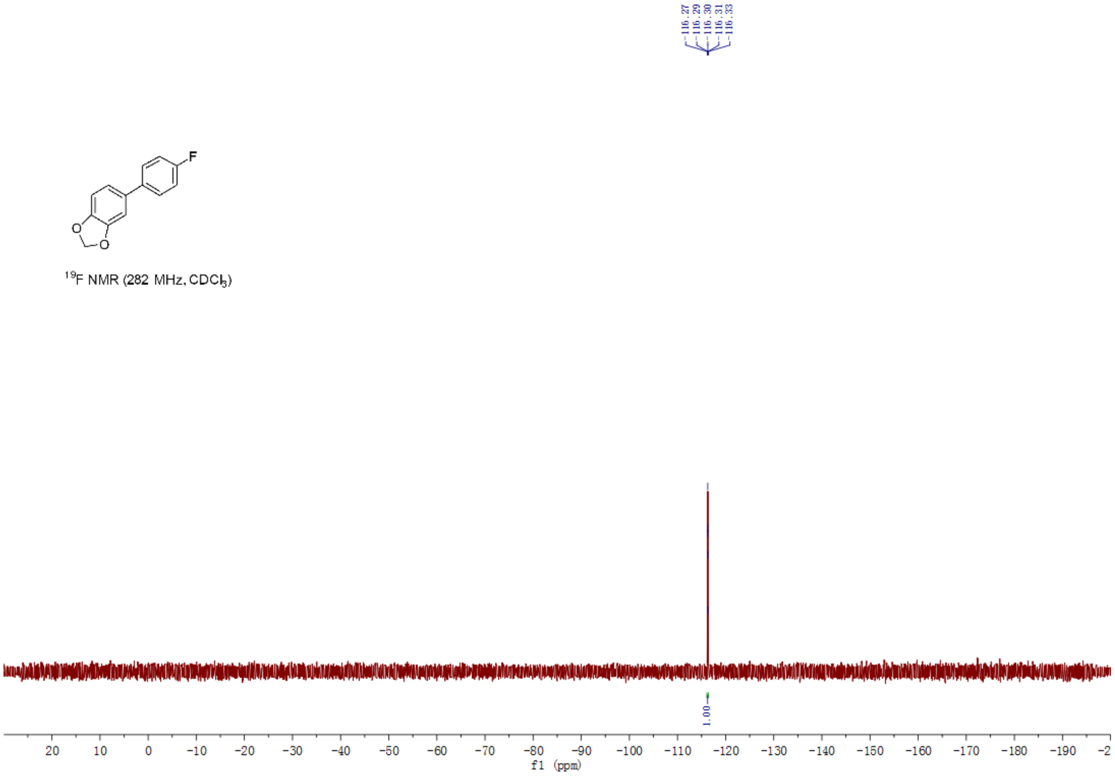


**Supplementary Figure 16**. NMR Spectra of 5-(4-fluorophenyl)benzo[*d*][1,3]dioxole.

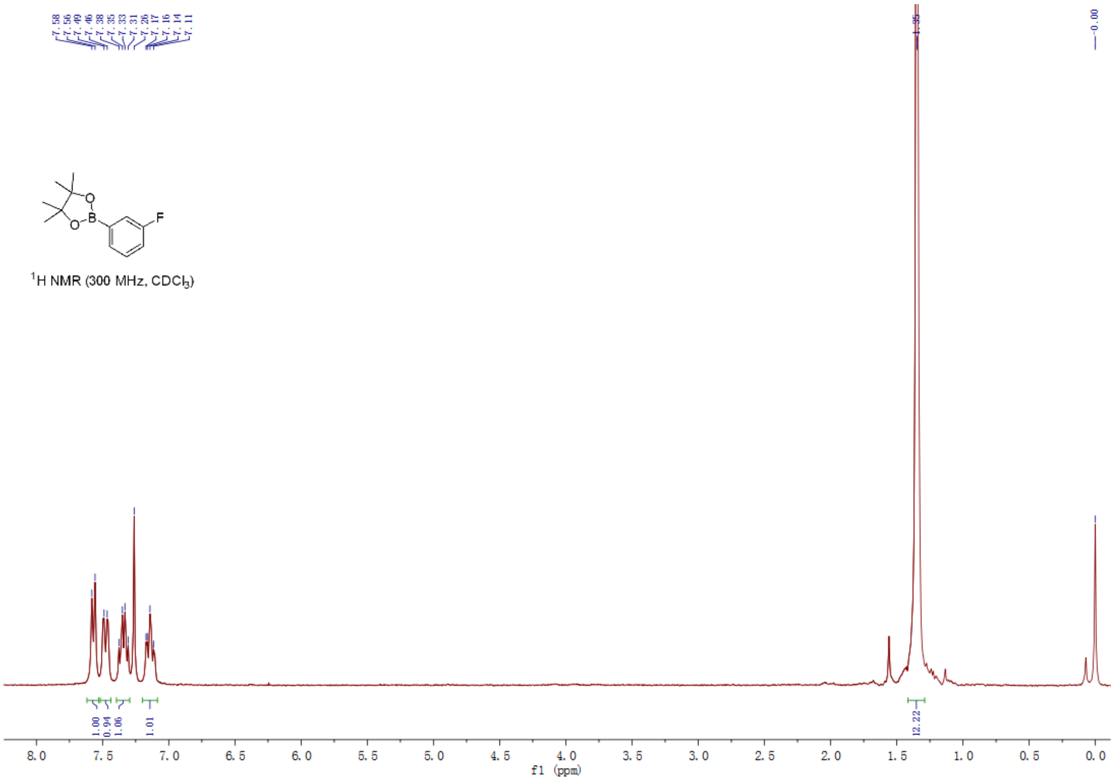

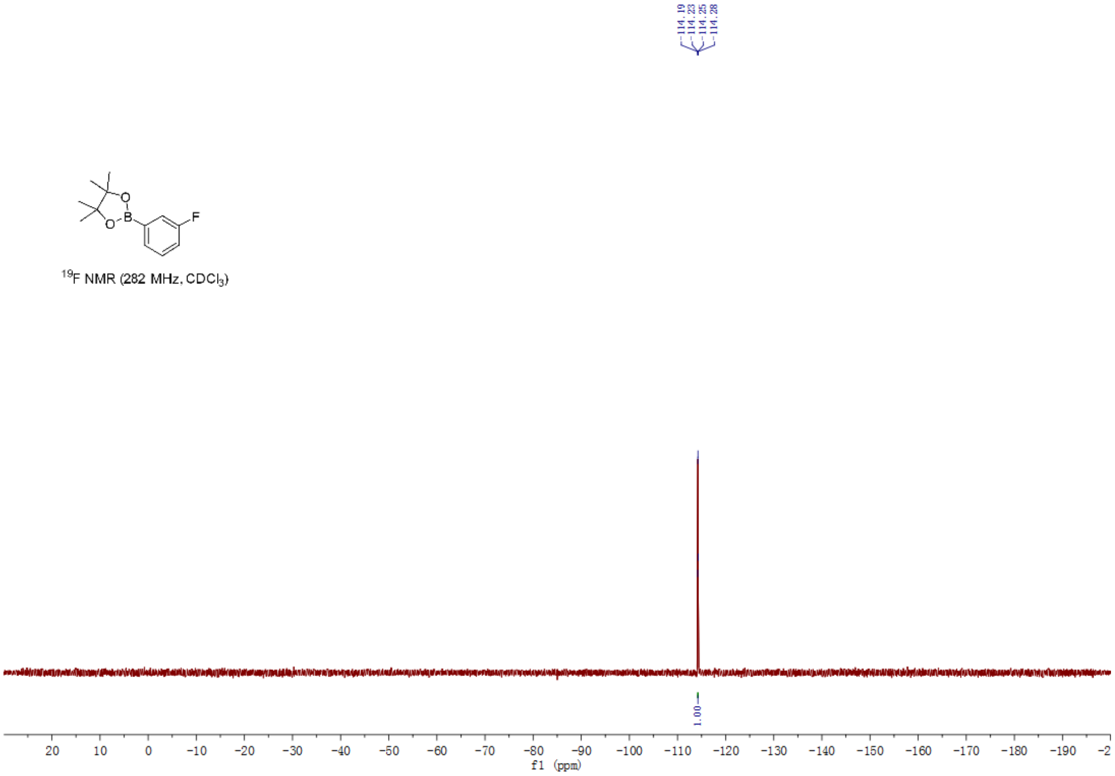

**Supplementary Figure 17**. NMR Spectra of 2-(3-fluorophenyl)-4,4,5,5-tetramethyl-1,3,2-dioxaborolane.

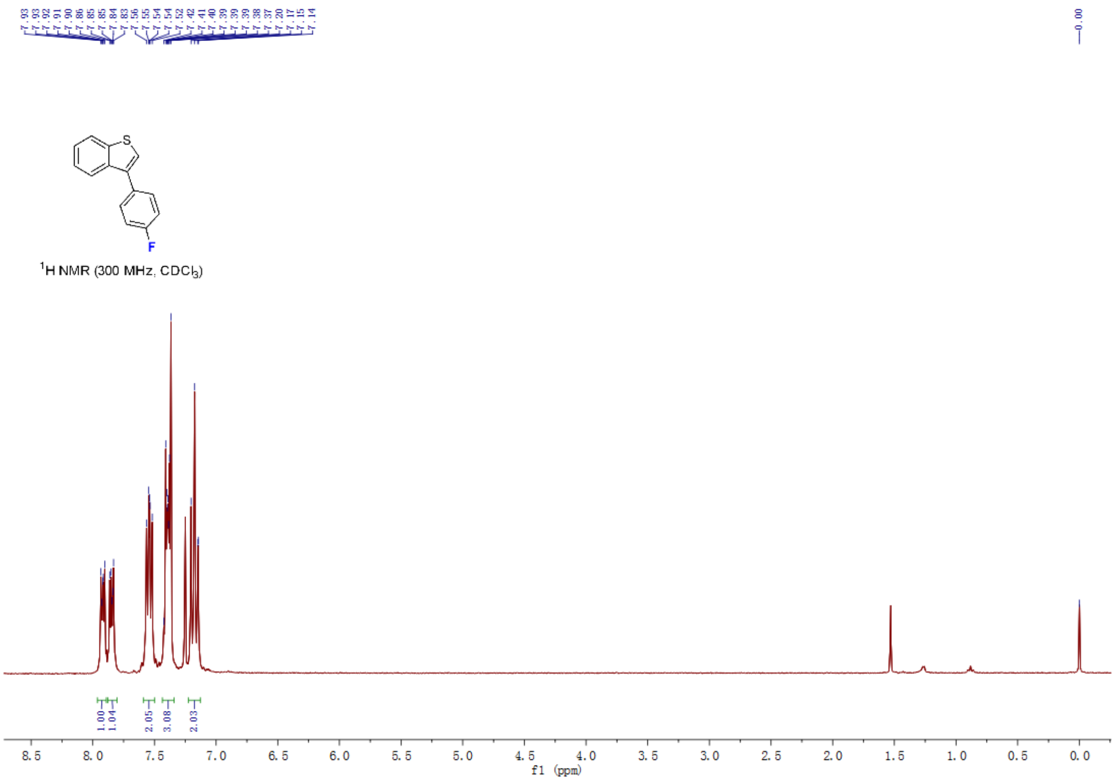


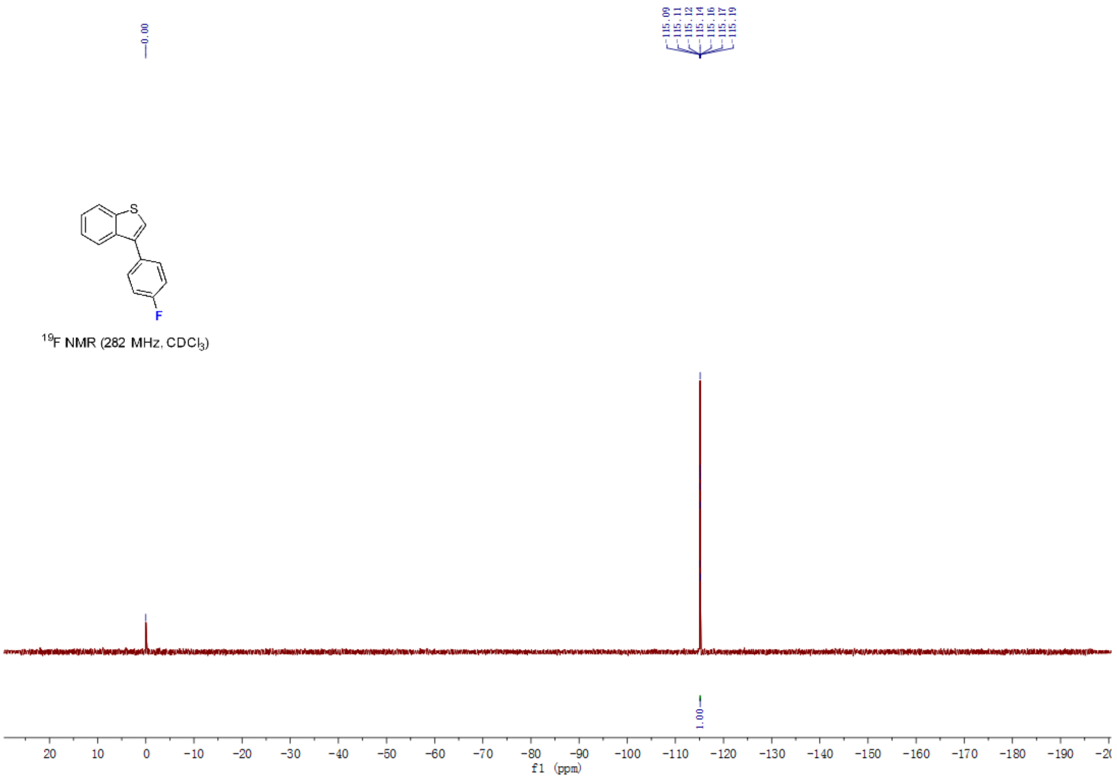


**Supplementary Figure 18**. NMR Spectra of 3-(4-fluorophenyl)benzo[*b*]thiophene.

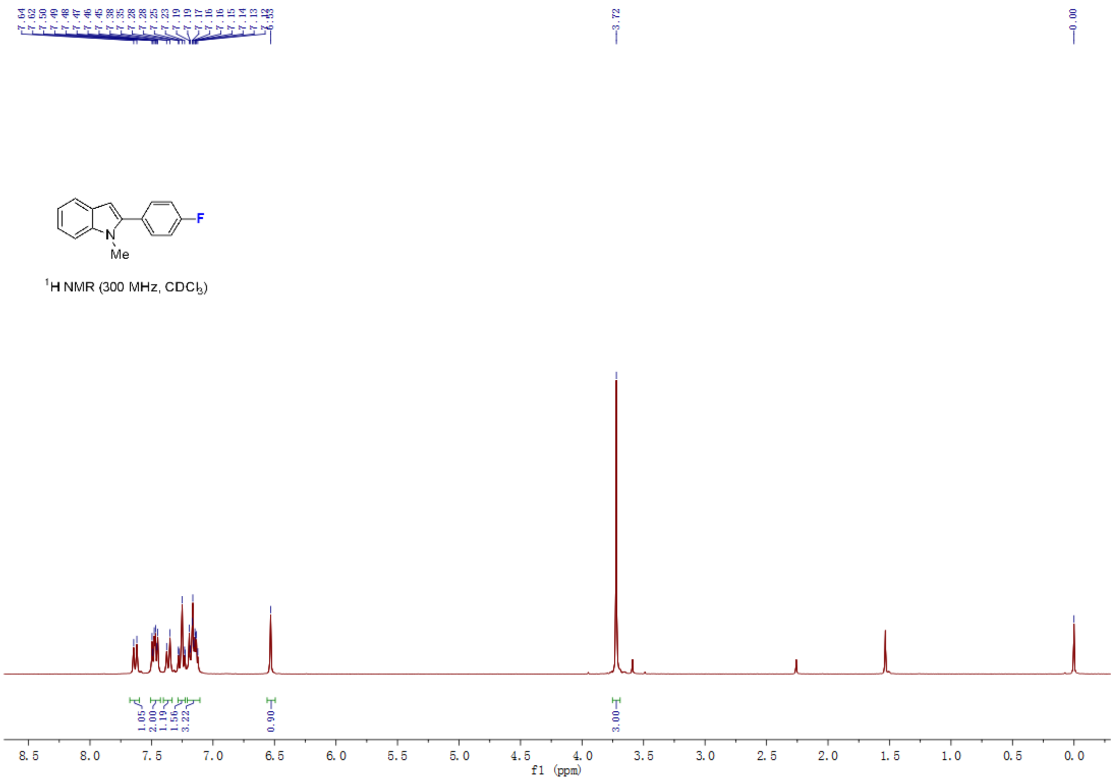


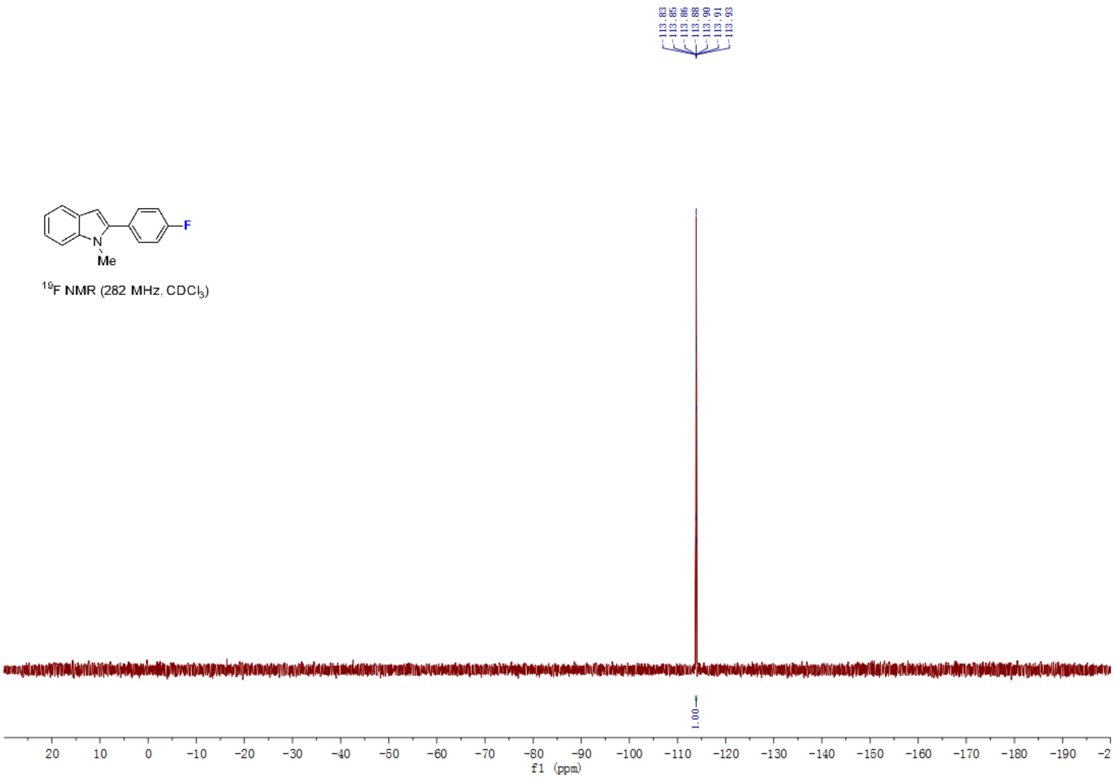


**Supplementary Figure 19**. NMR Spectra of 2-(4-fluorophenyl)-1-methyl-1*H*-indole

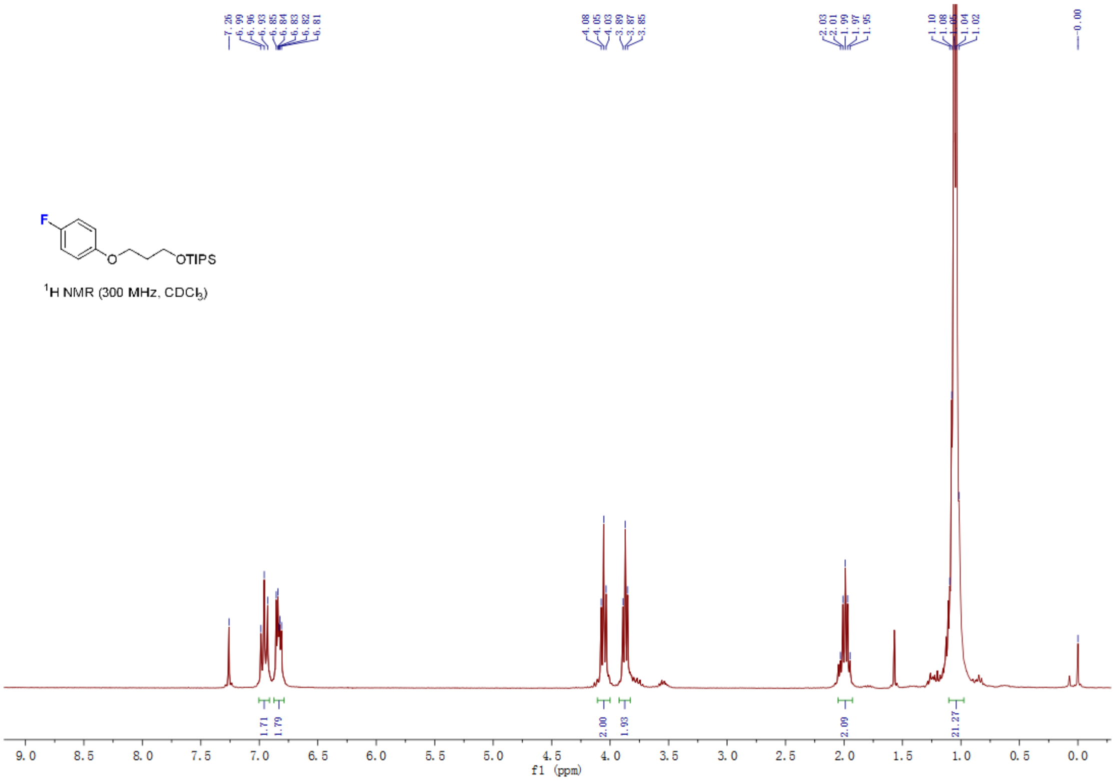
F
O
OTIPS
1H NMR (300 MHz, CDCl3)
f1 (ppm)

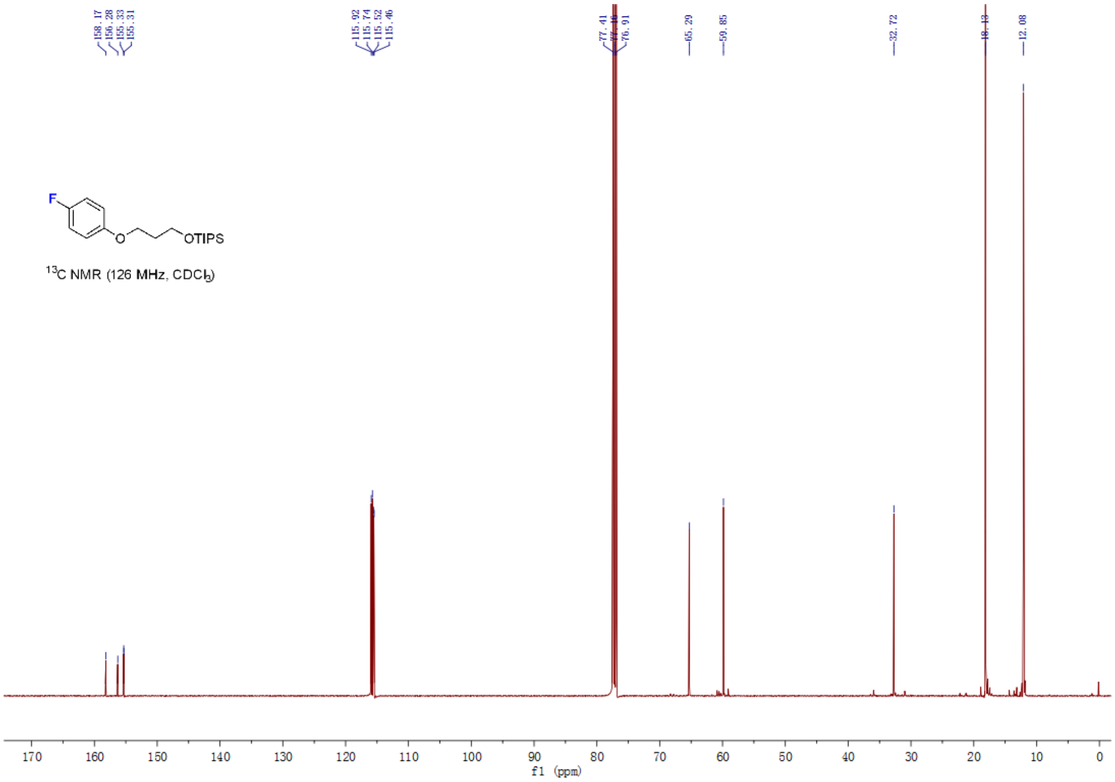
F
O
OTIPS
13C NMR (126 MHz, CDCl3)
f1 (ppm)

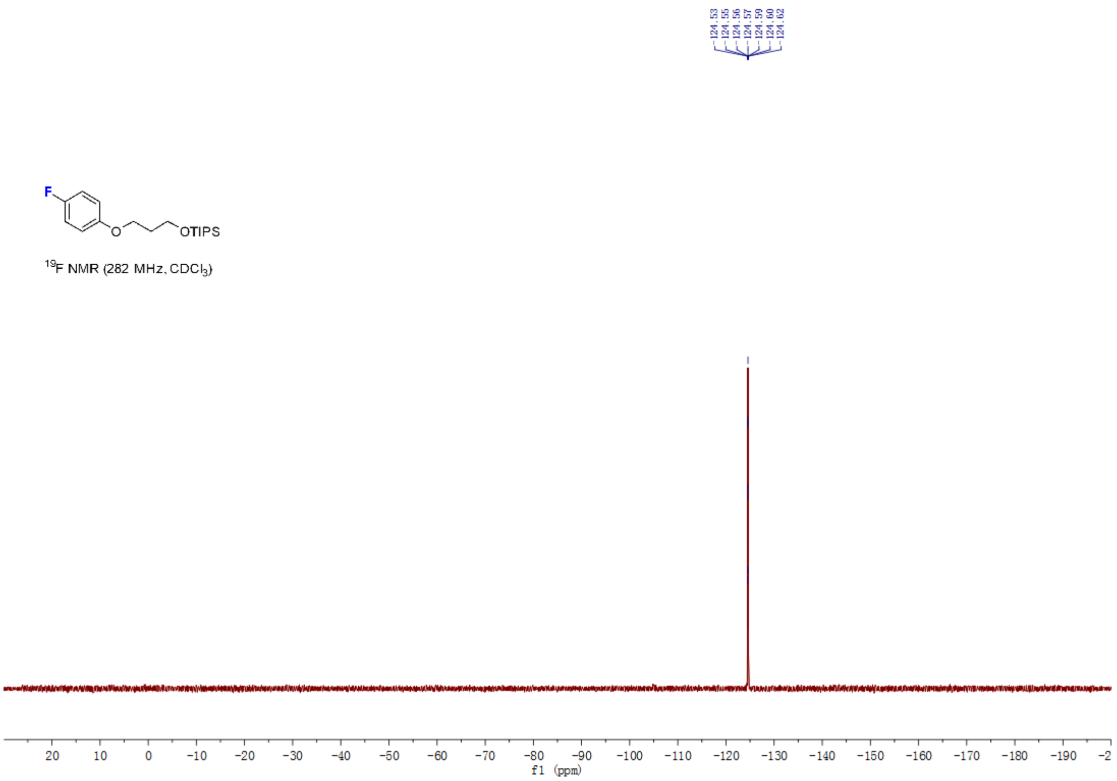


**Supplementary Figure 20**. NMR Spectra of (3-(4-fluorophenoxy)propoxy)triisopropylsilane.

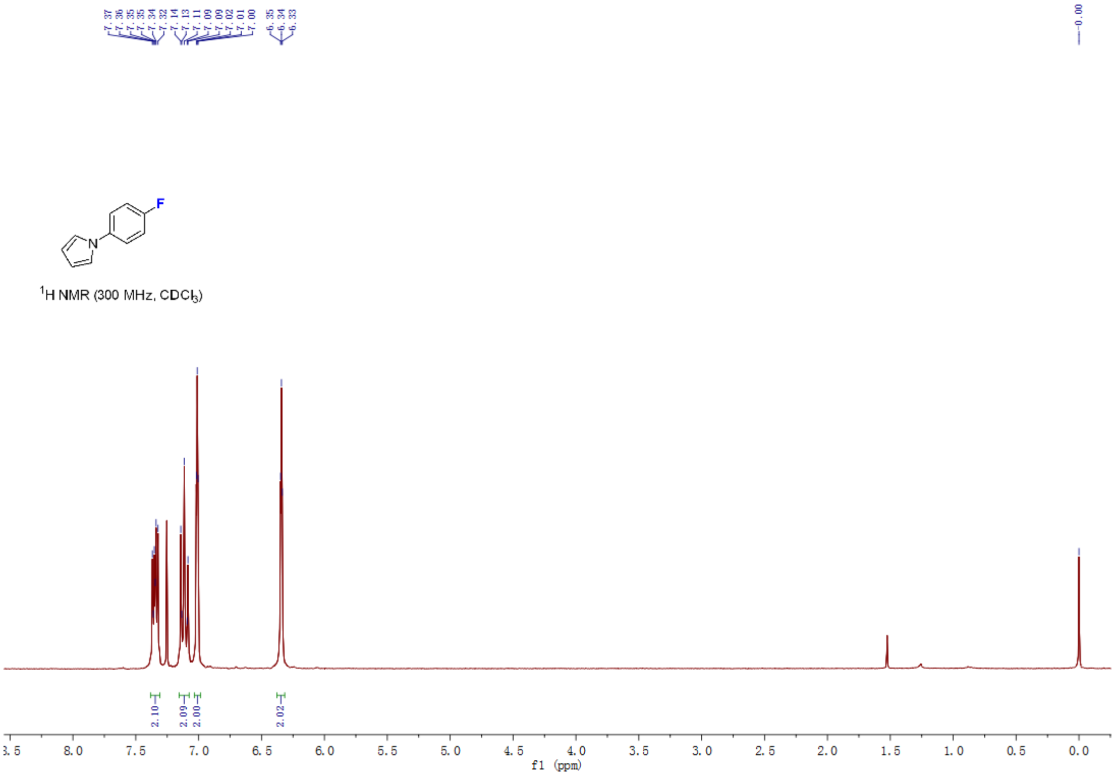


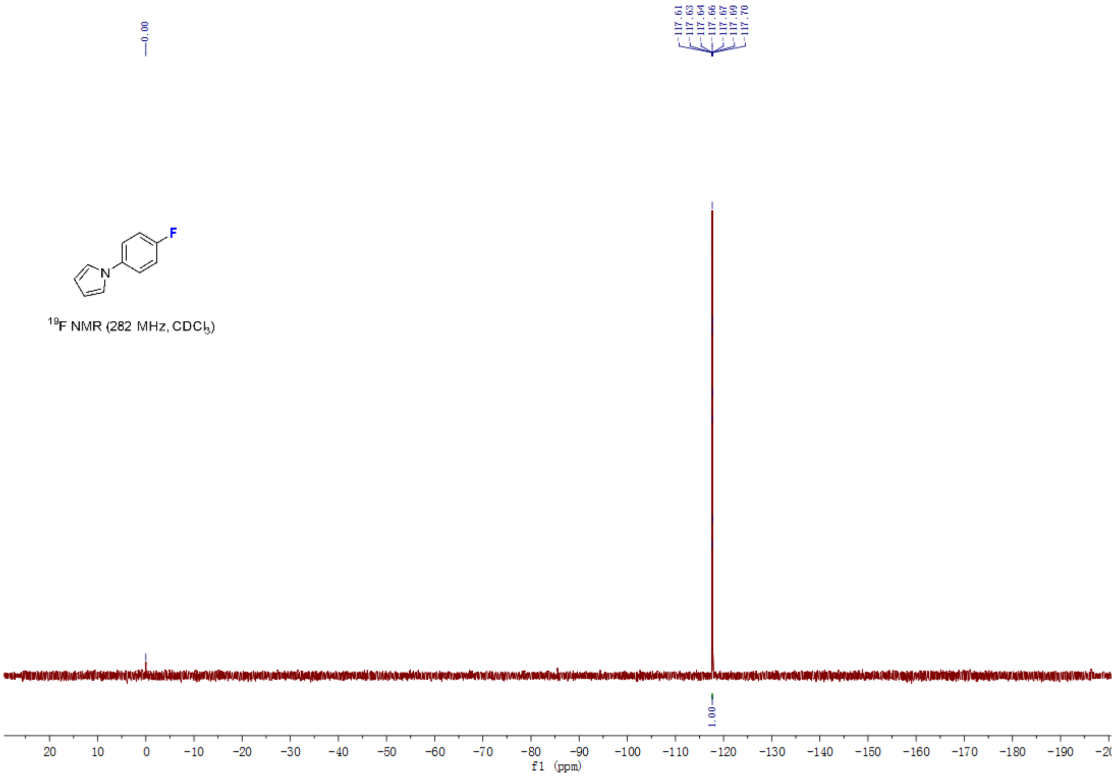


**Supplementary Figure 21**. NMR Spectra of 1-(4-fluorophenyl)-1*H*-pyrrole

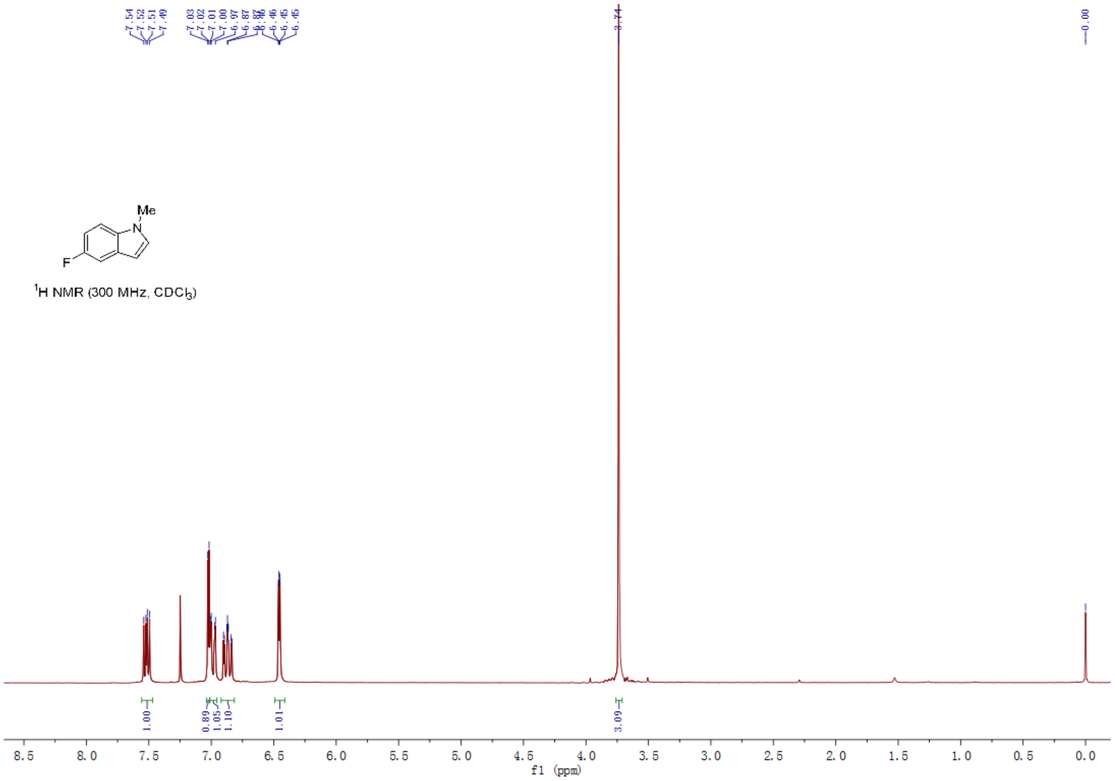

7.54
7.52
7.51
7.49
7.03
7.02
7.01
7.00
6.97
6.87
6.46
6.45
3.74
0.00
Me
N
F
$^{1}$H NMR (300 MHz, CDCl3)
1.00
0.89
1.05
1.10
1.01
3.09
f1 (ppm)


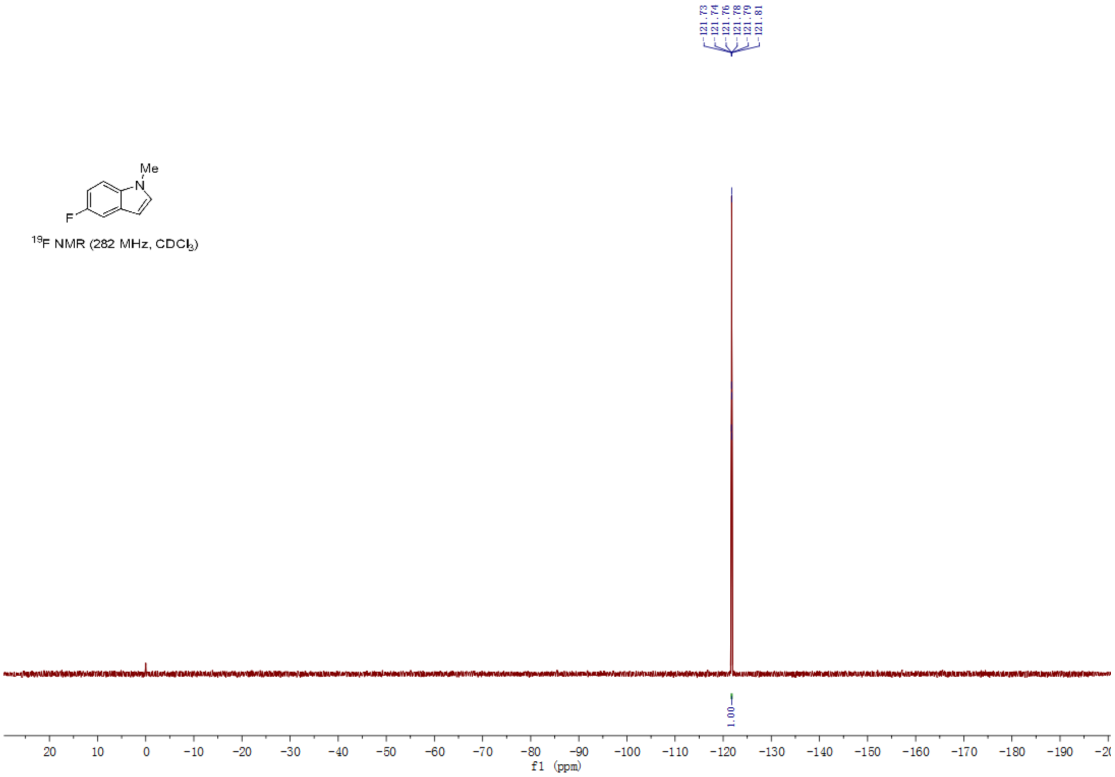

-121.73
-121.74
-121.76
-121.78
-121.79
-121.81
Me
N
F
$^{19}$F NMR (282 MHz, CDCl3)
1.00
f1 (ppm)

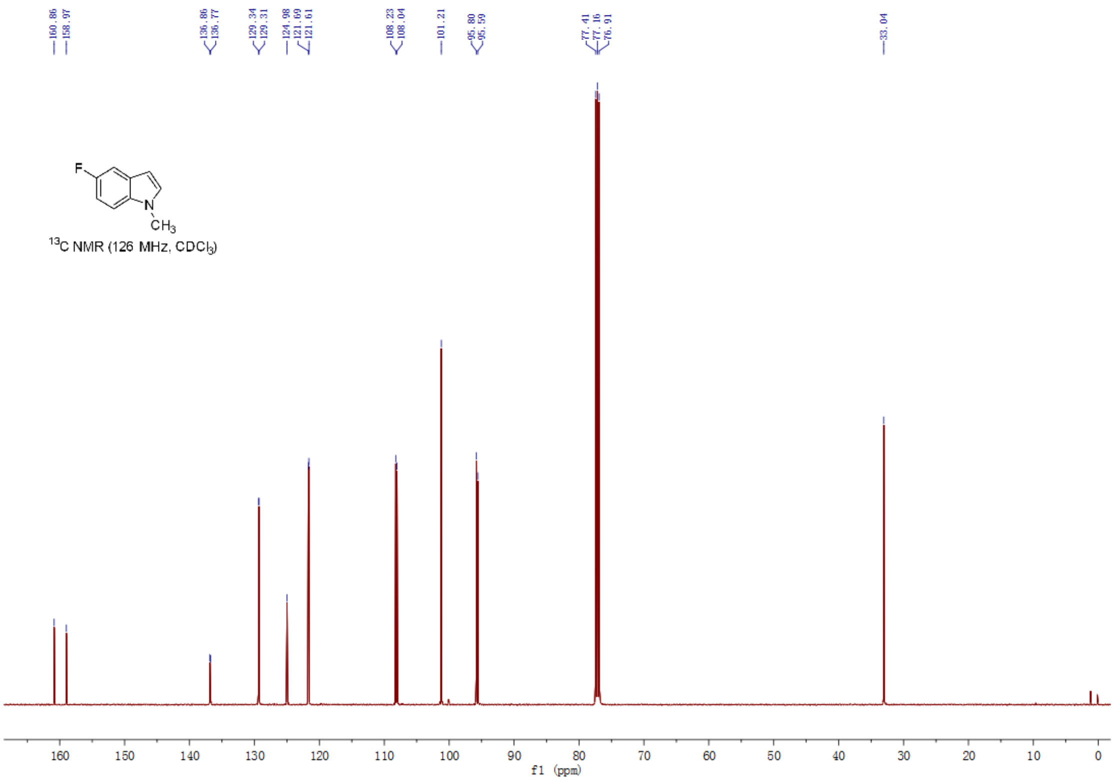


**Supplementary Figure 22**. NMR Spectra of 5-fluoro-1-methyl-1*H*-indole

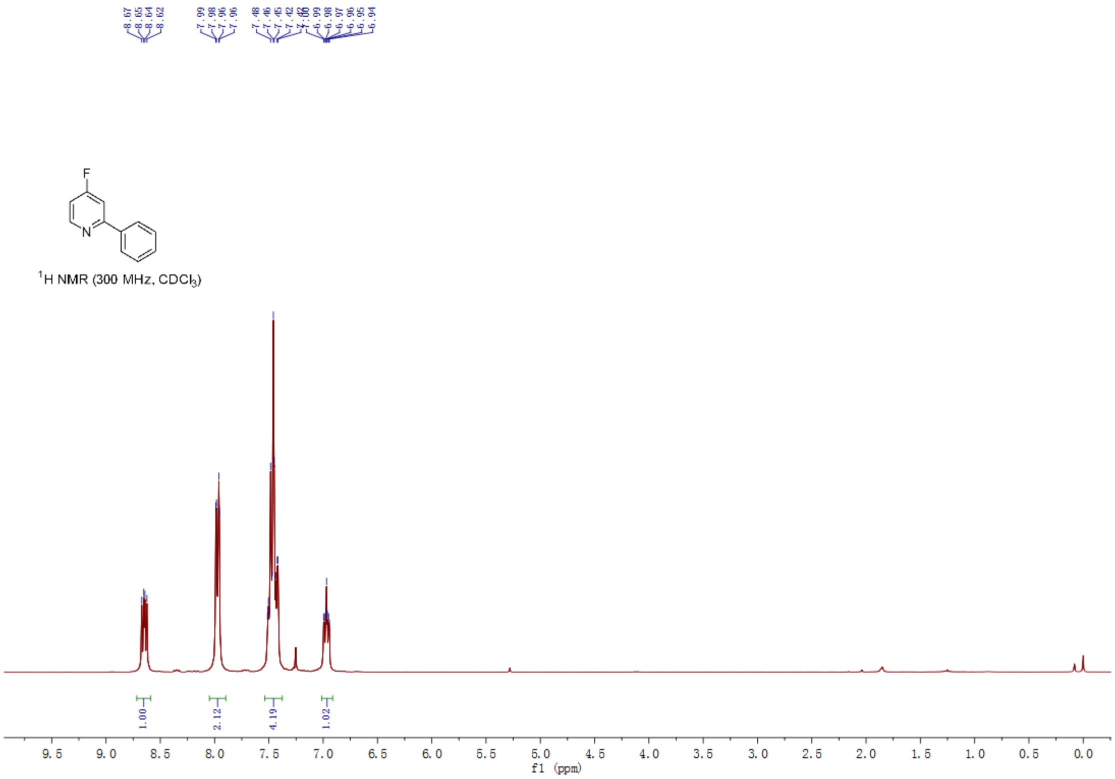


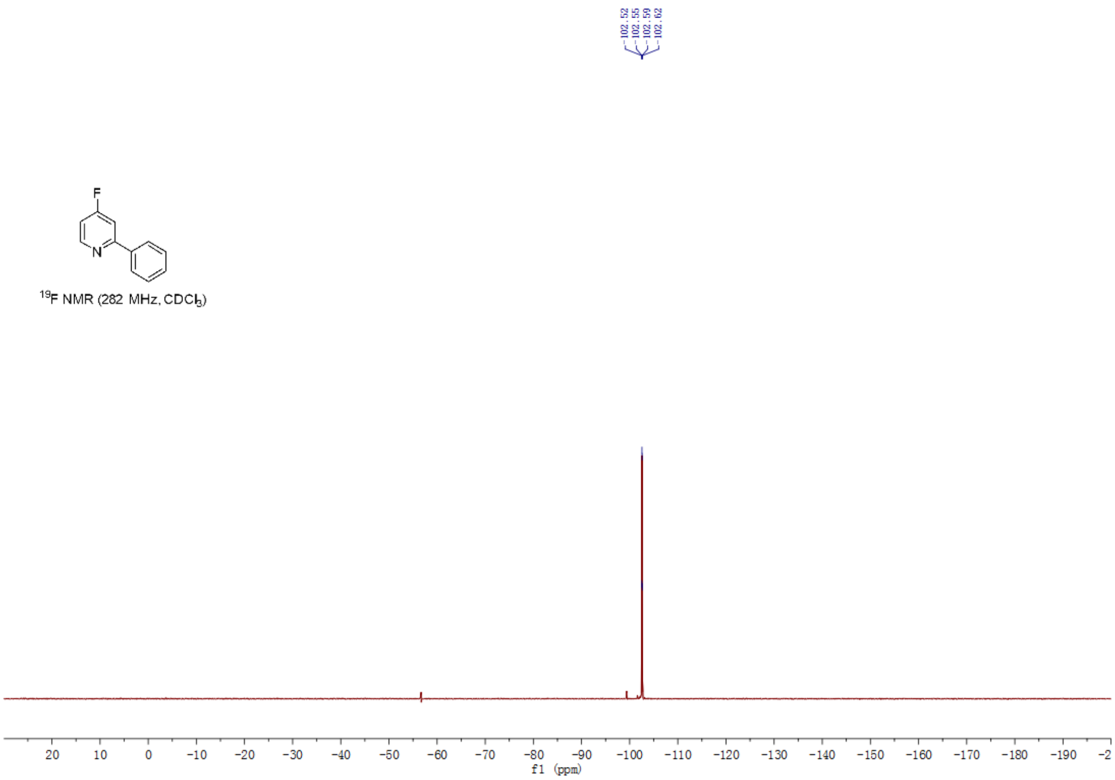


**Supplementary Figure 23**. NMR Spectra of 4-fluoro-2-phenylpyridine.

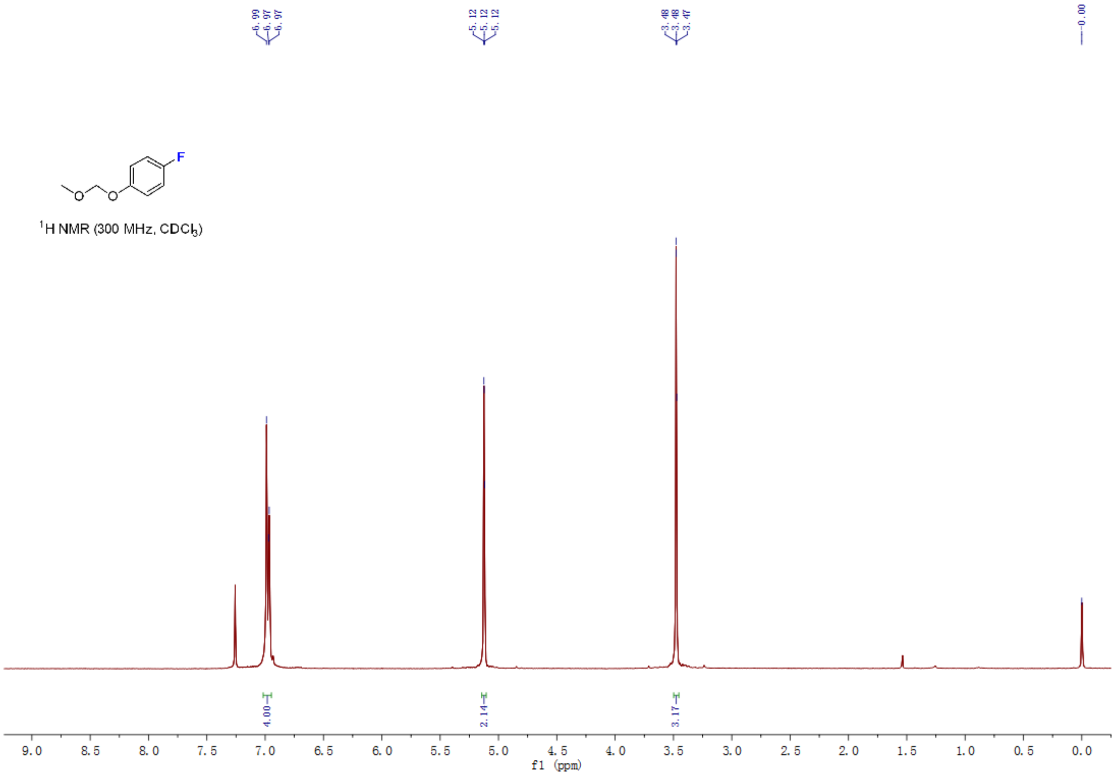


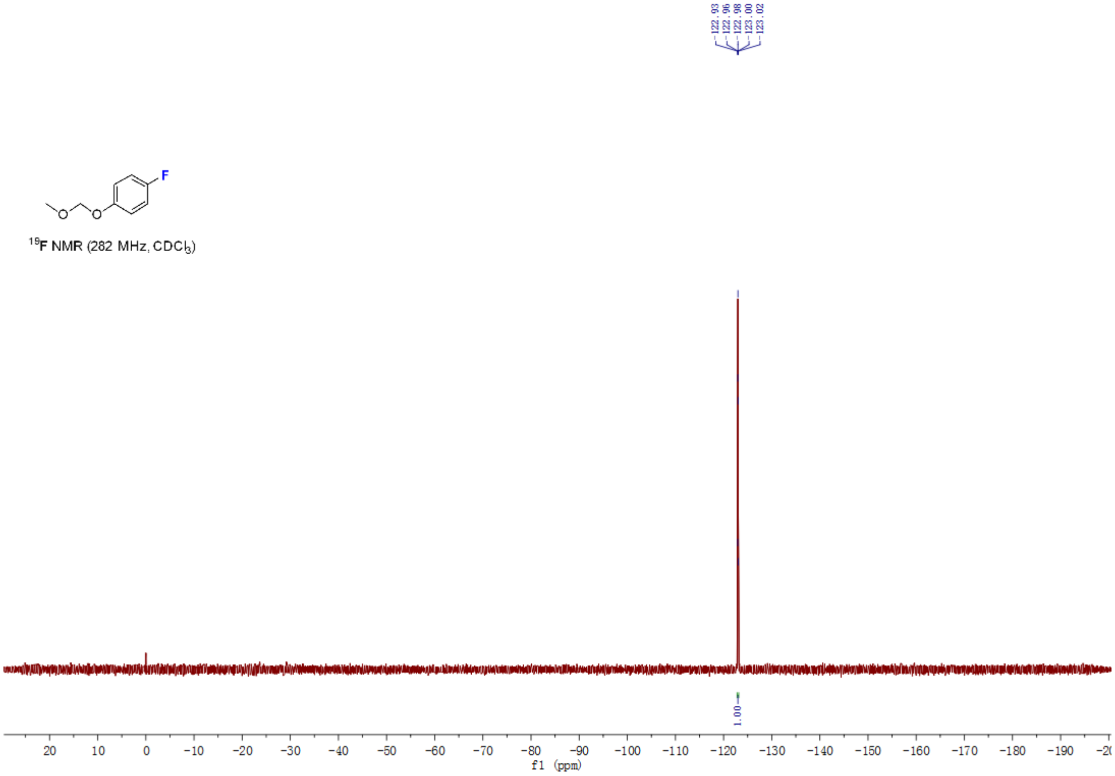


**Supplementary Figure 24**. NMR Spectra of 1-fluoro-4-(methoxymethoxy)benzene.

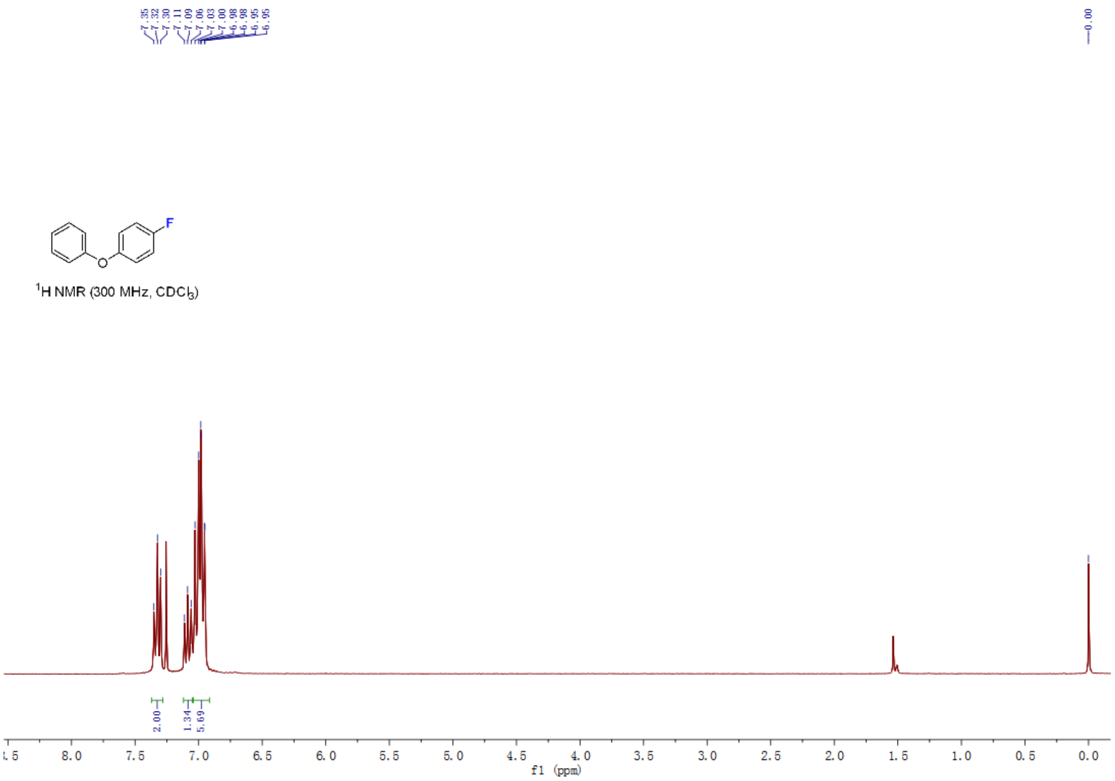


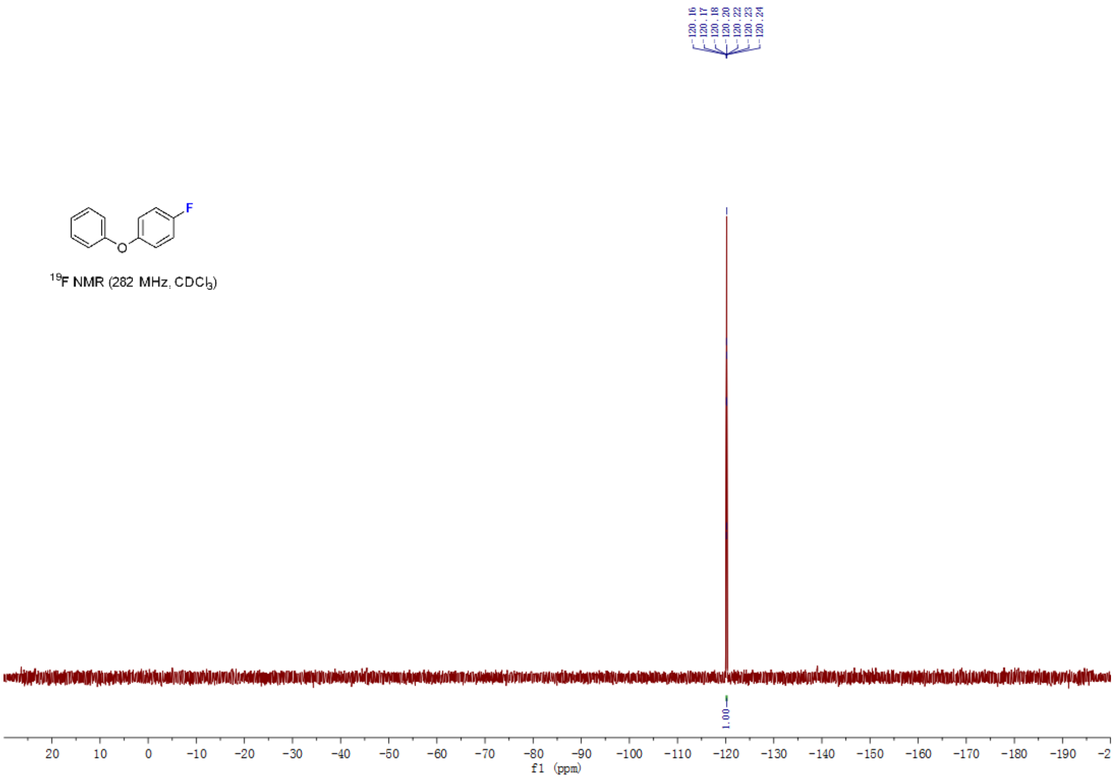


**Supplementary Figure 25**. NMR Spectra of 1-fluoro-4-phenoxybenzene.

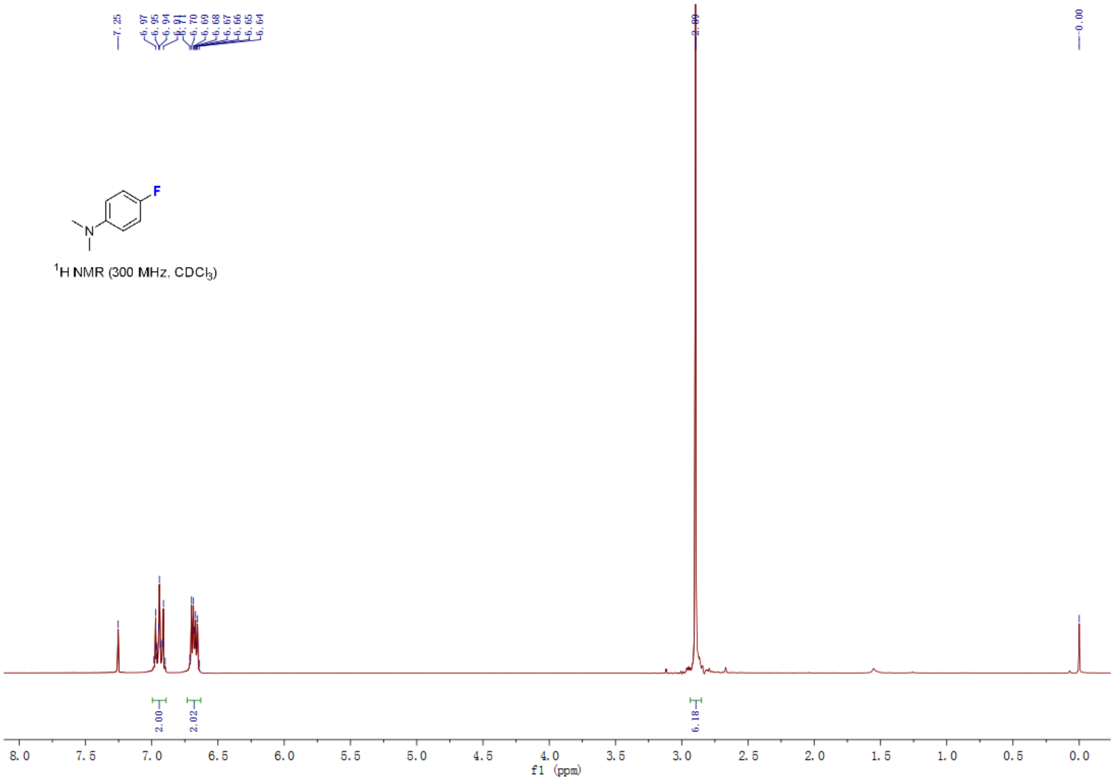


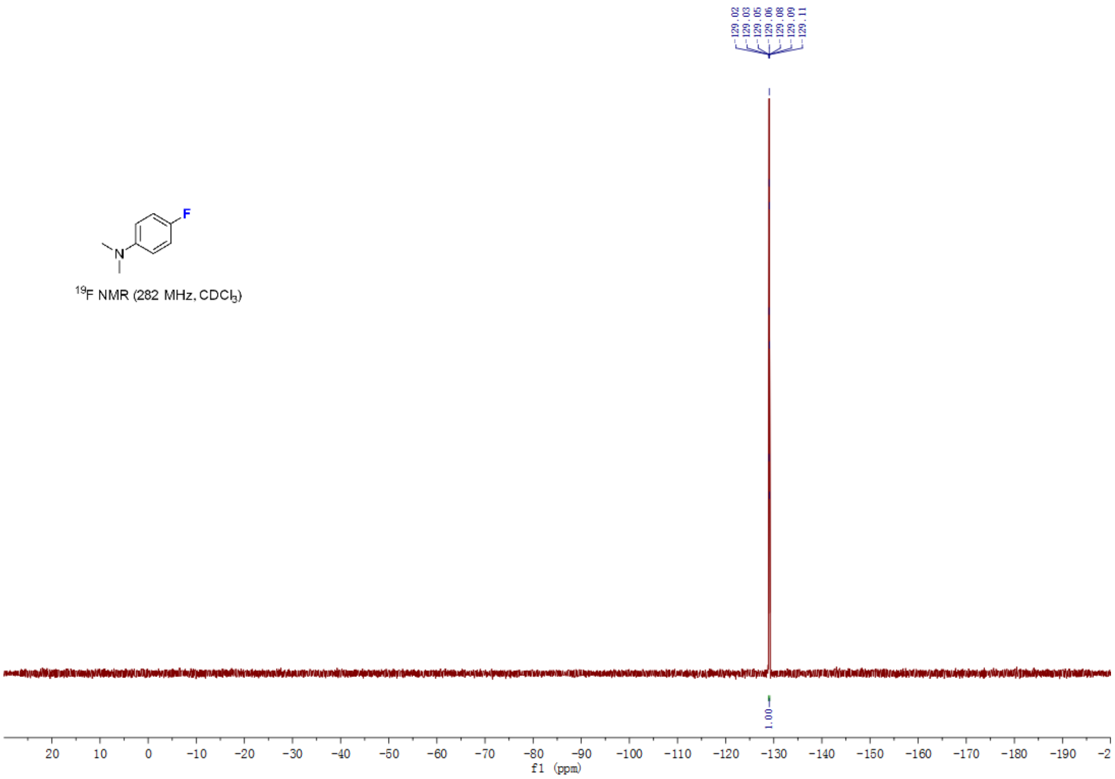


**Supplementary Figure 26**. NMR Spectra of 4-fluoro-*N*,*N*-dimethylaniline.

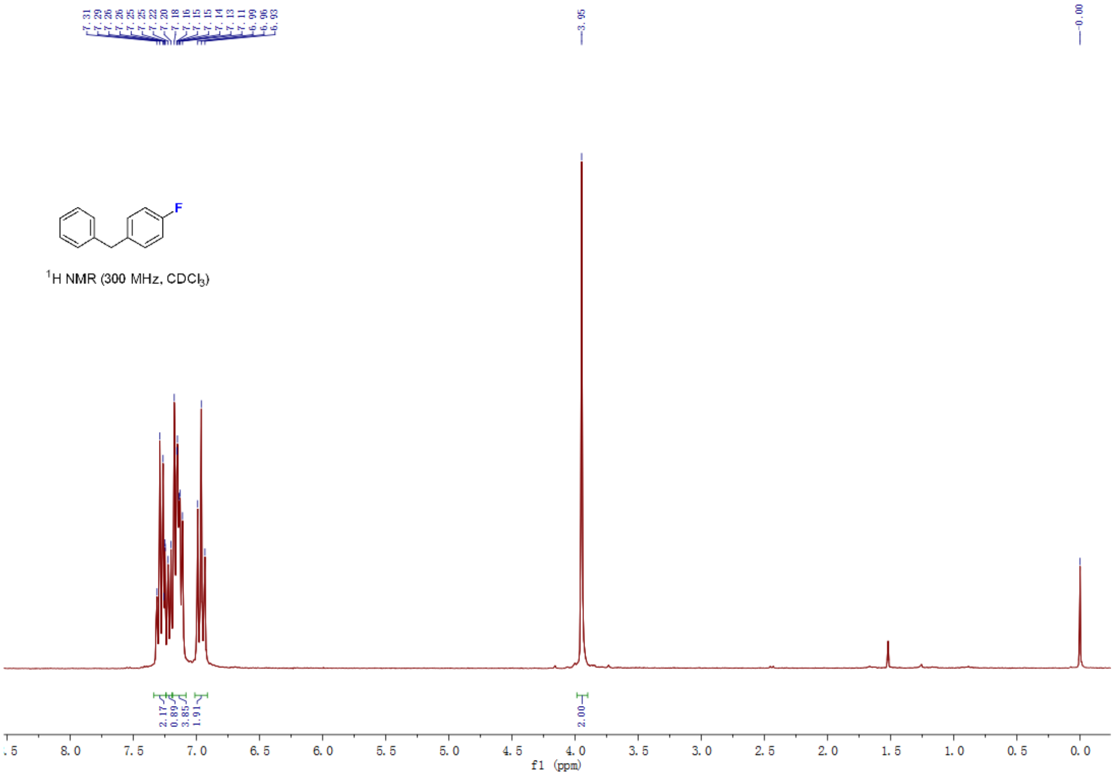


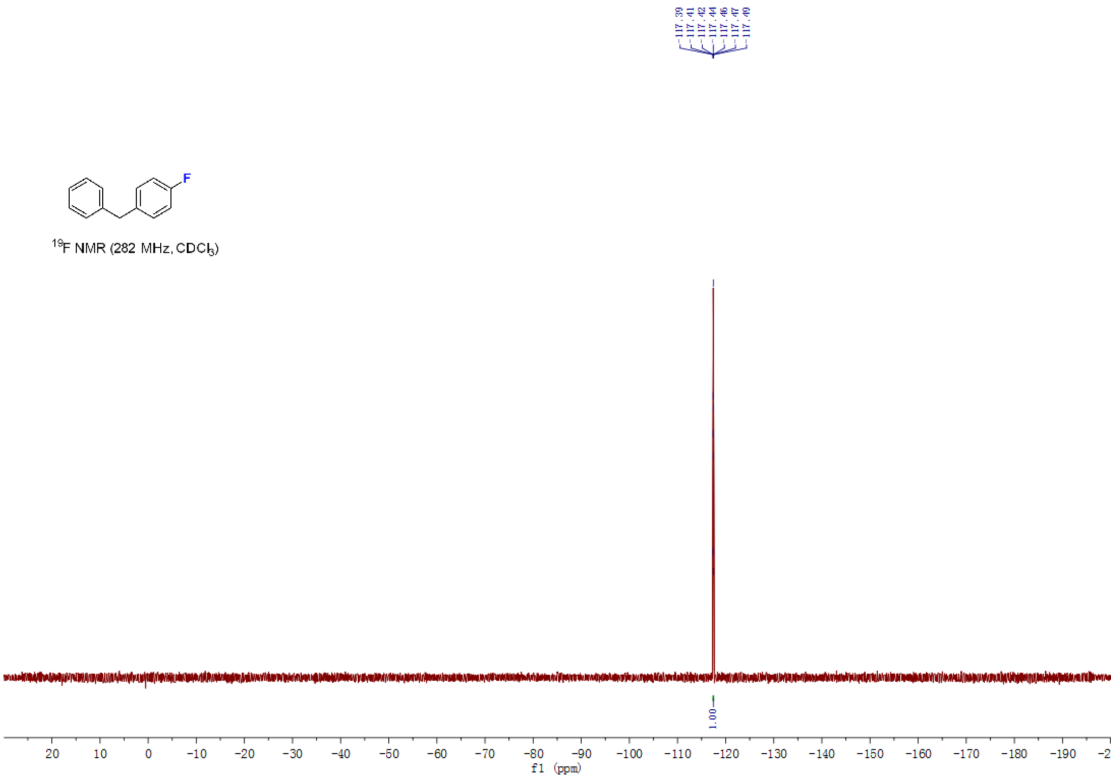


**Supplementary Figure 27**. NMR Spectra of 1-benzyl-4-fluorobenzene.

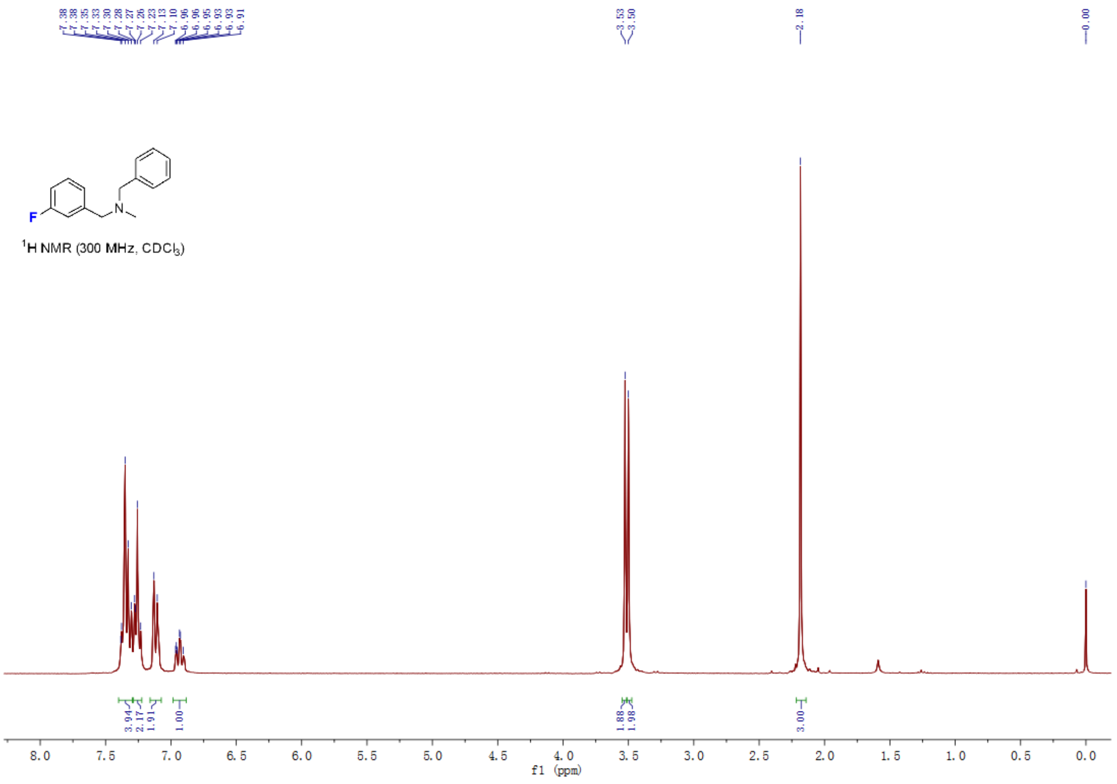

F
N
$^{1}$H NMR (300 MHz, CDCl$_3$)
7.38
7.38
7.35
7.33
7.30
7.28
7.27
7.26
7.23
7.13
7.10
6.96
6.96
6.95
6.93
6.93
6.91
3.53
3.50
2.18
0.00
3.94
2.17
1.91
1.00
1.88
1.98
3.00
f1 (ppm)


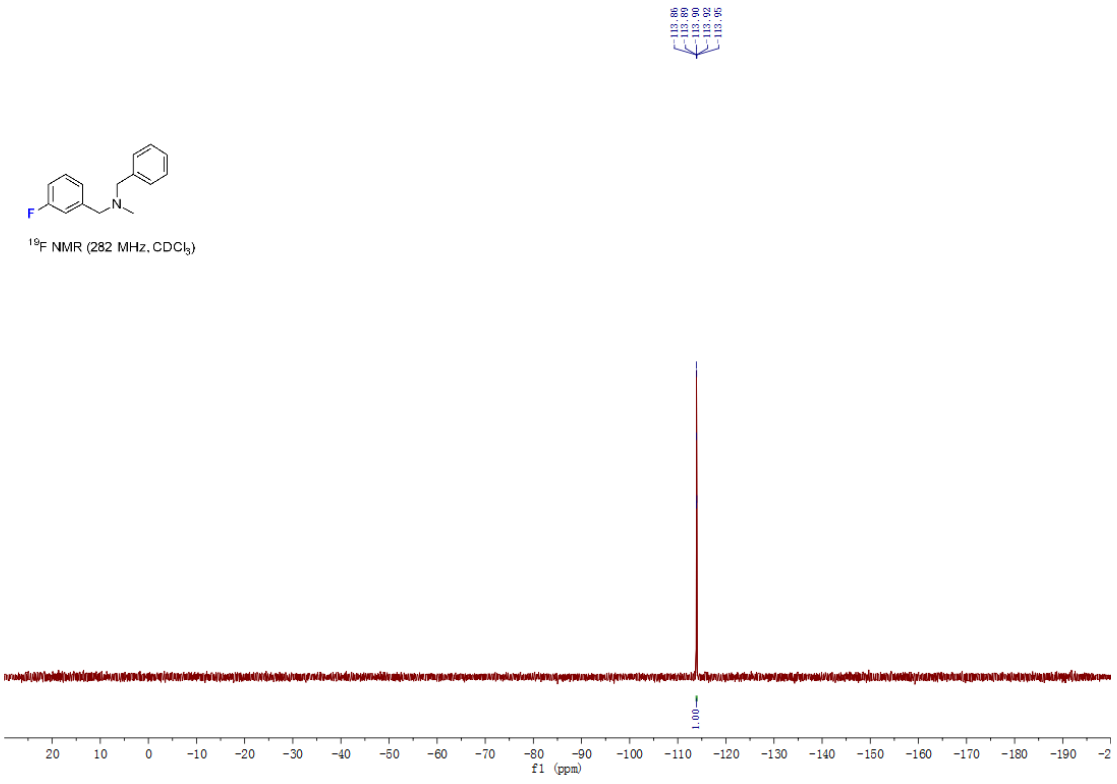

F
N
$^{19}$F NMR (282 MHz, CDCl$_3$)
-113.86
-113.89
-113.90
-113.92
-113.95
1.00
f1 (ppm)

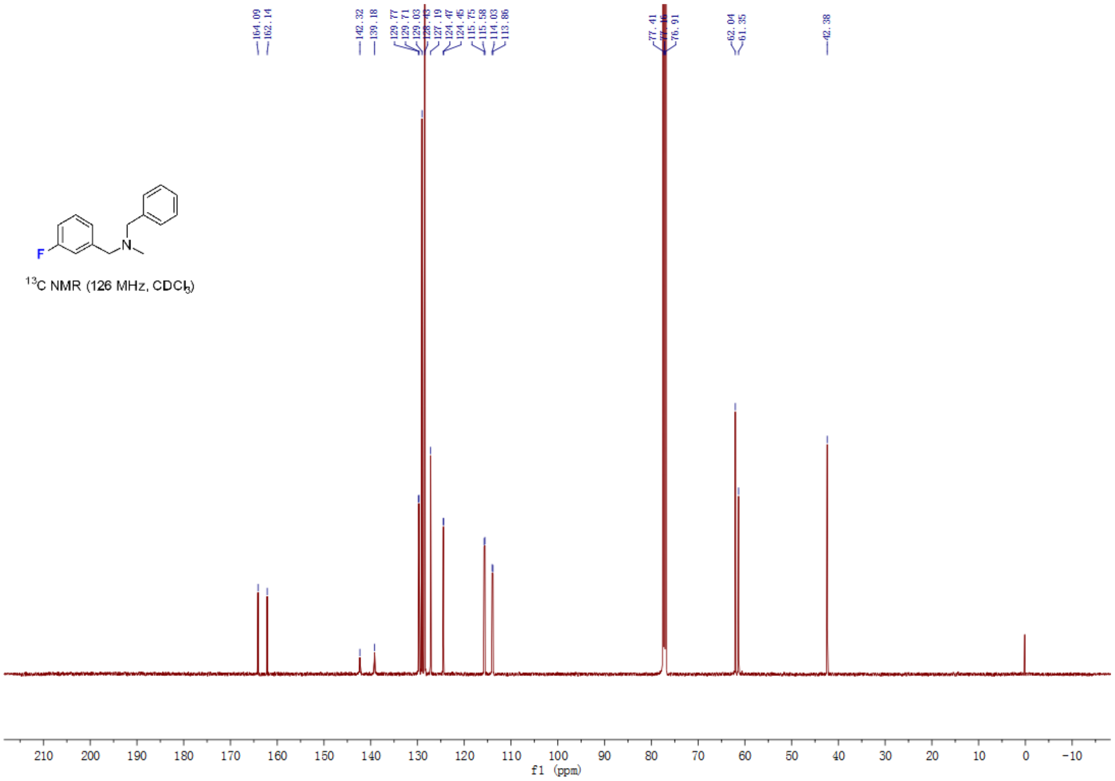


**Supplementary Figure 28**. NMR Spectra of *N*-benzyl-1-(3-fluorophenyl)-N-methylmethanamine.

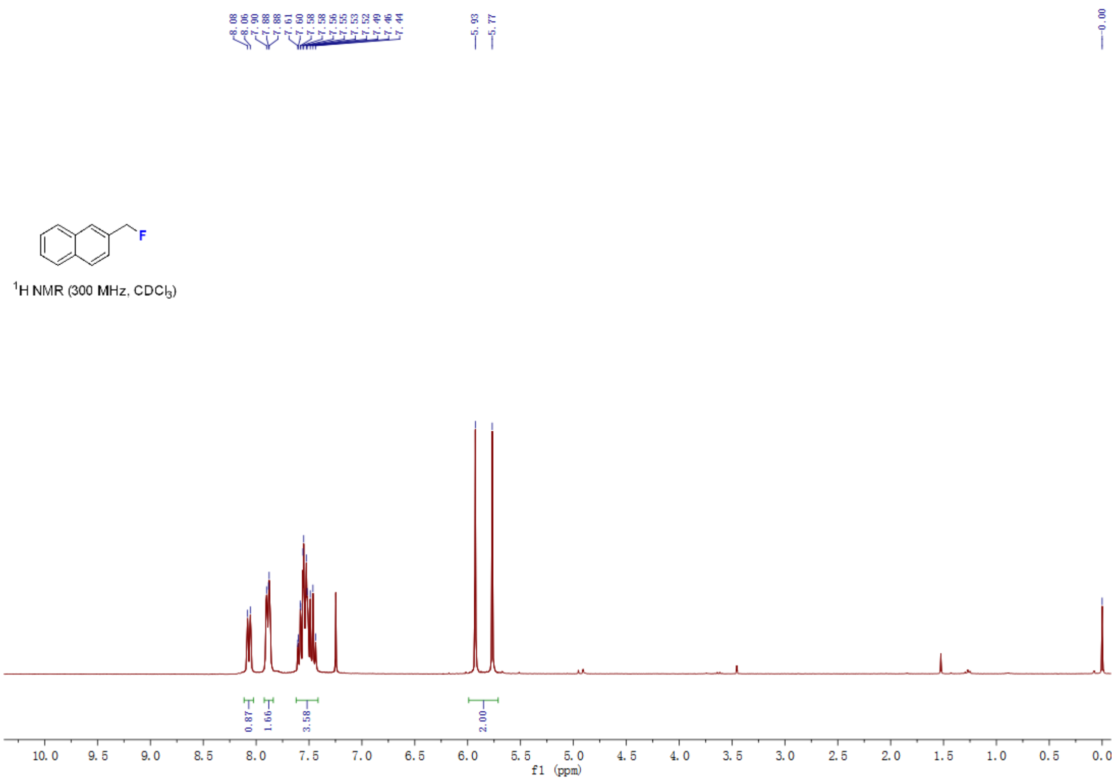


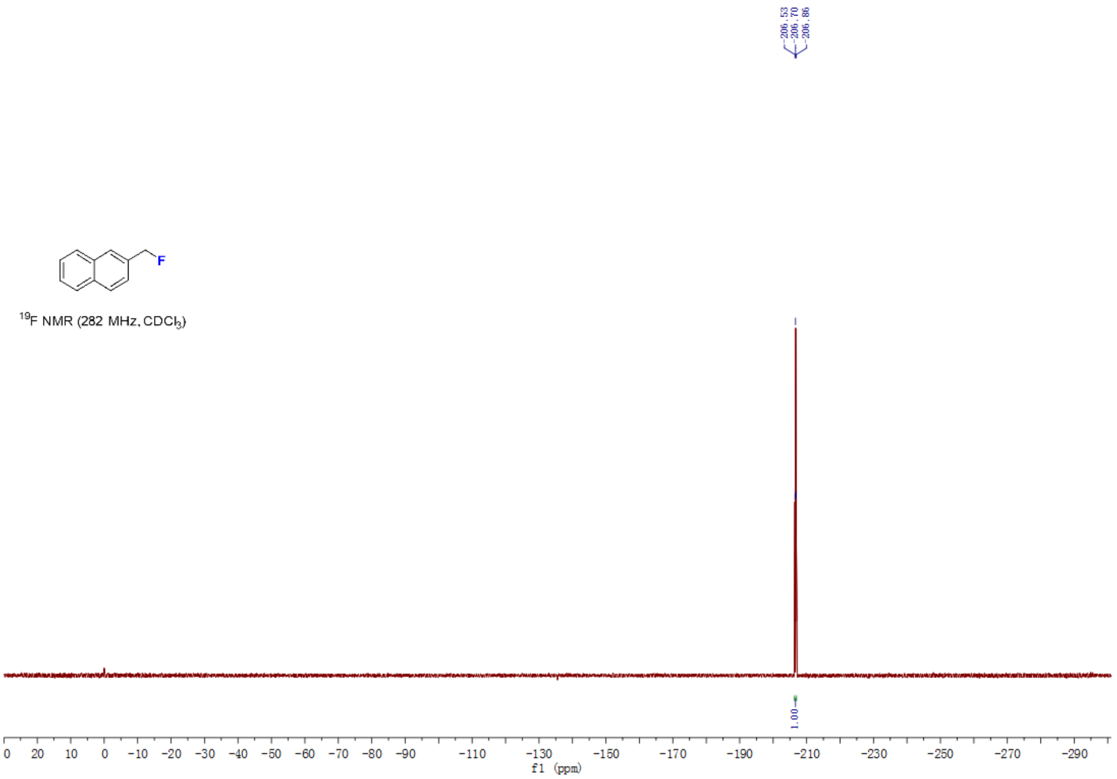


**Supplementary Figure 29**. NMR Spectra of 2-(fluoromethyl)naphthalene.

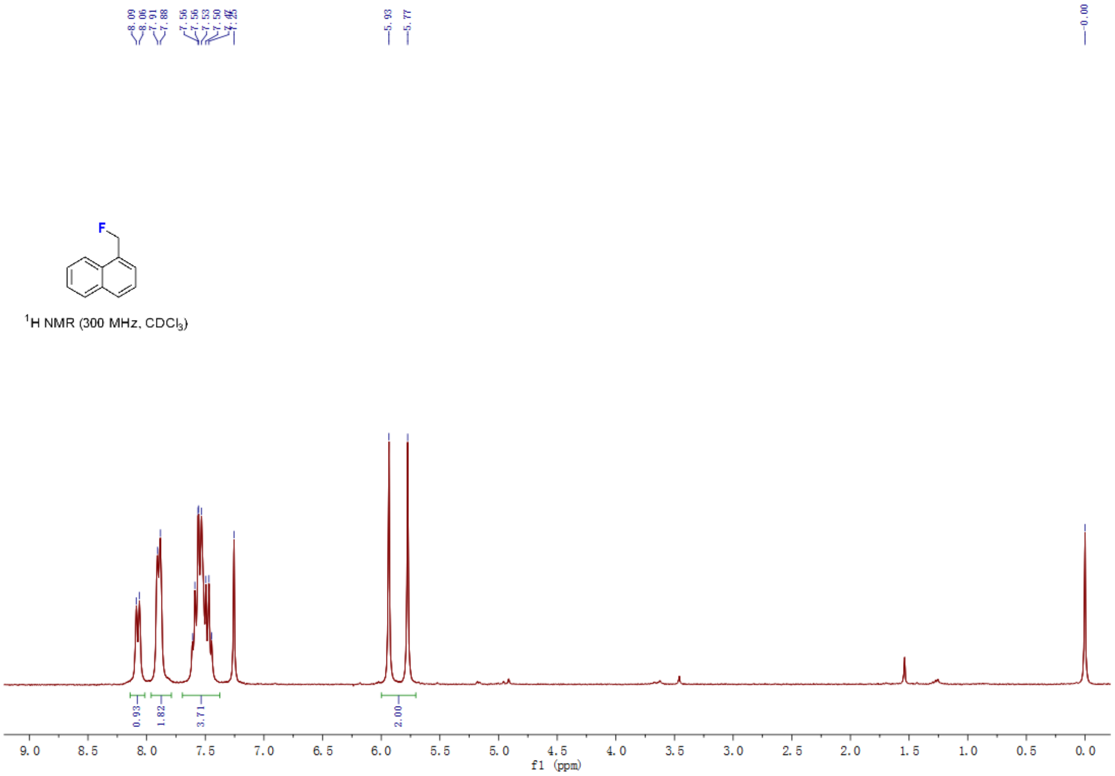


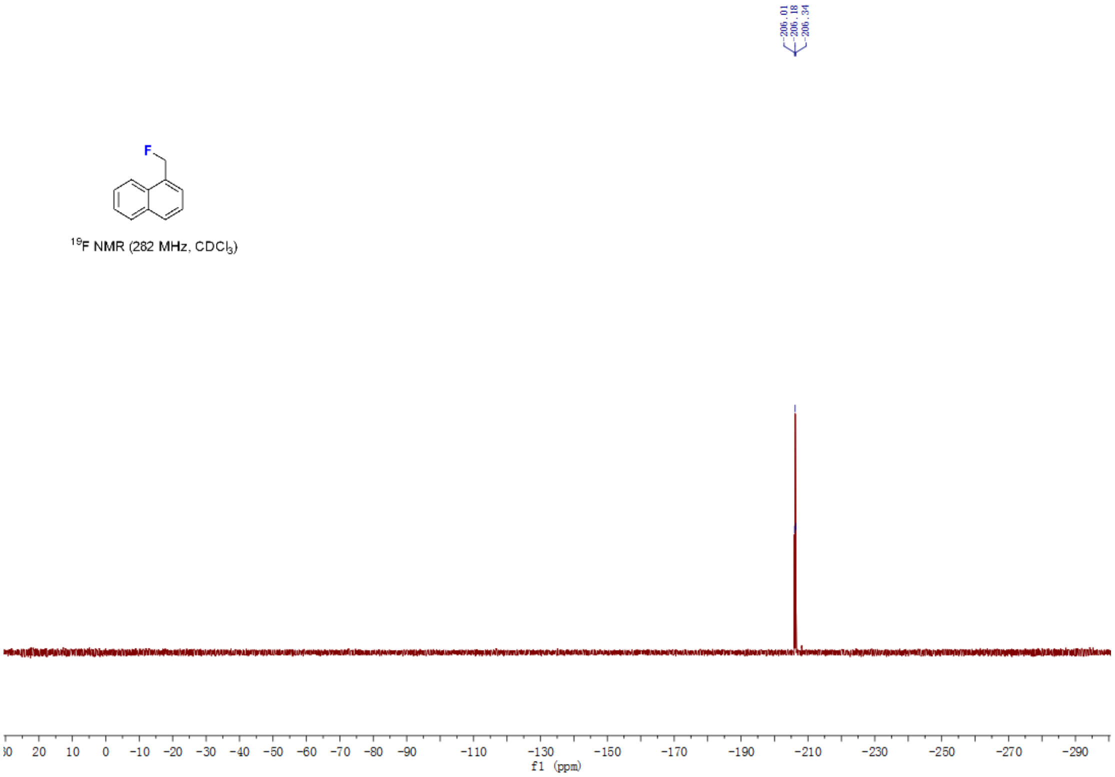


**Supplementary Figure 30**. NMR Spectra of 1-(fluoromethyl)naphthalene.

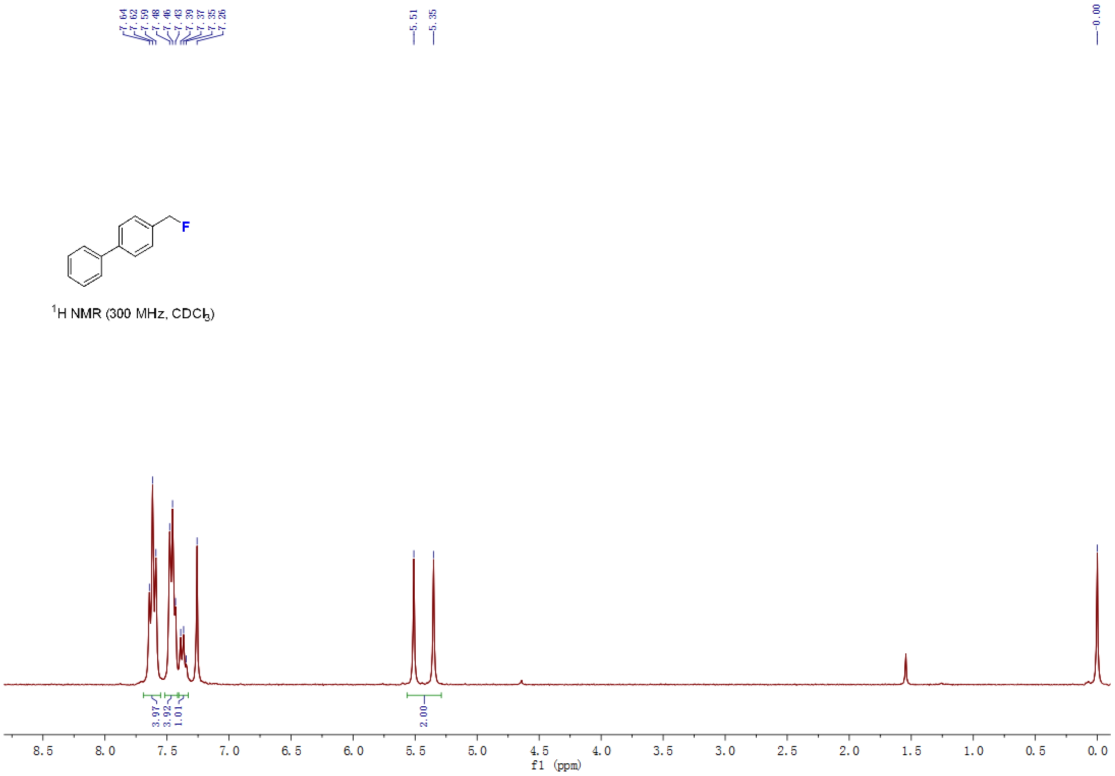


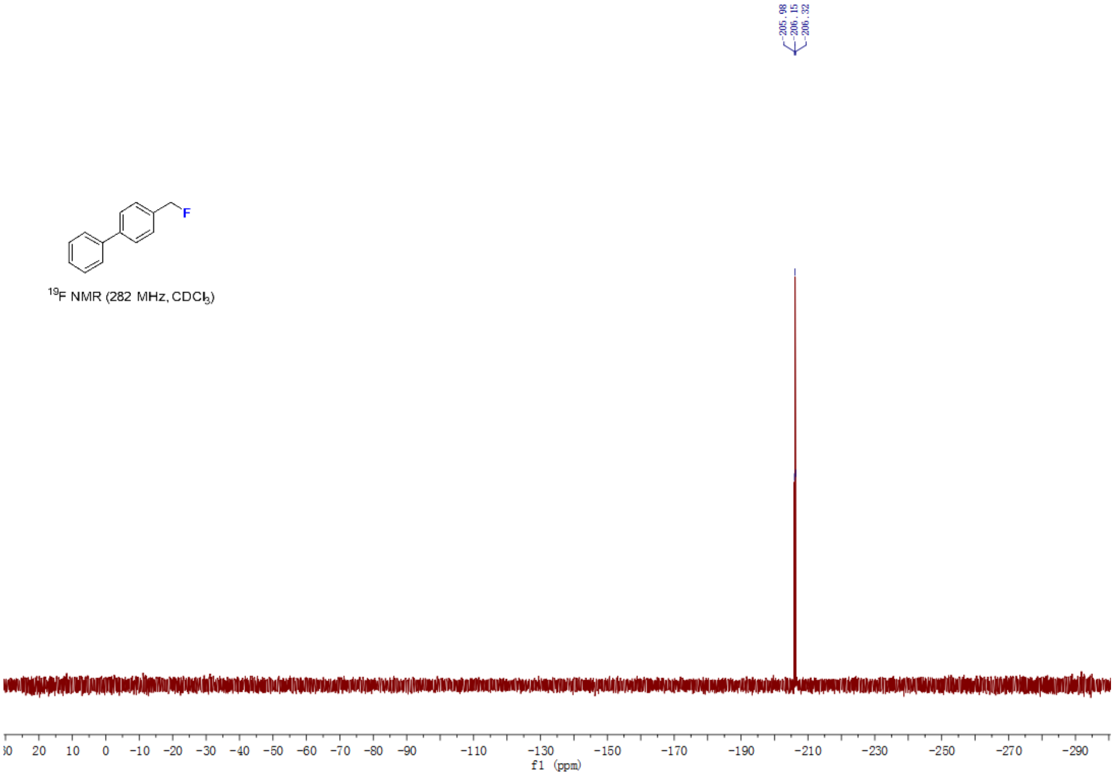


**Supplementary Figure 31**. NMR Spectra of 4-(fluoromethyl)-1,1'-biphenyl.

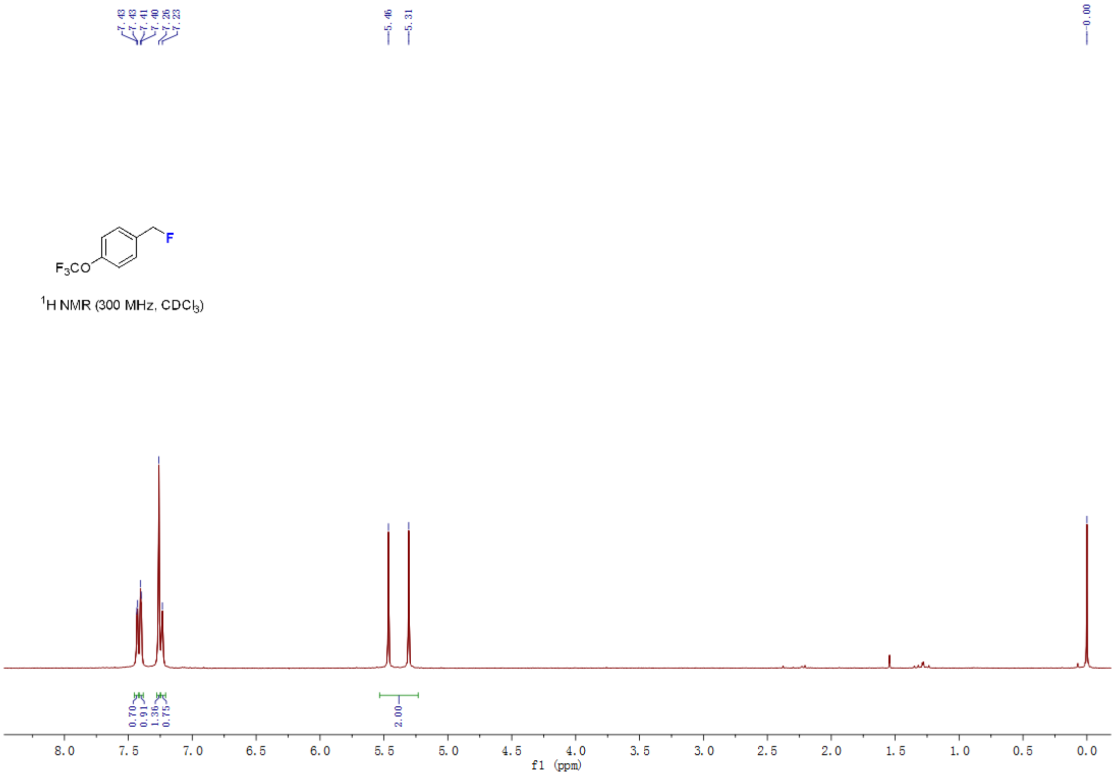

F
F3CO
1H NMR (300 MHz, CDCl3)
7.43
7.43
7.41
7.40
7.26
7.23
5.46
5.31
0.00
0.70
0.91
1.36
0.75
2.00
f1 (ppm)


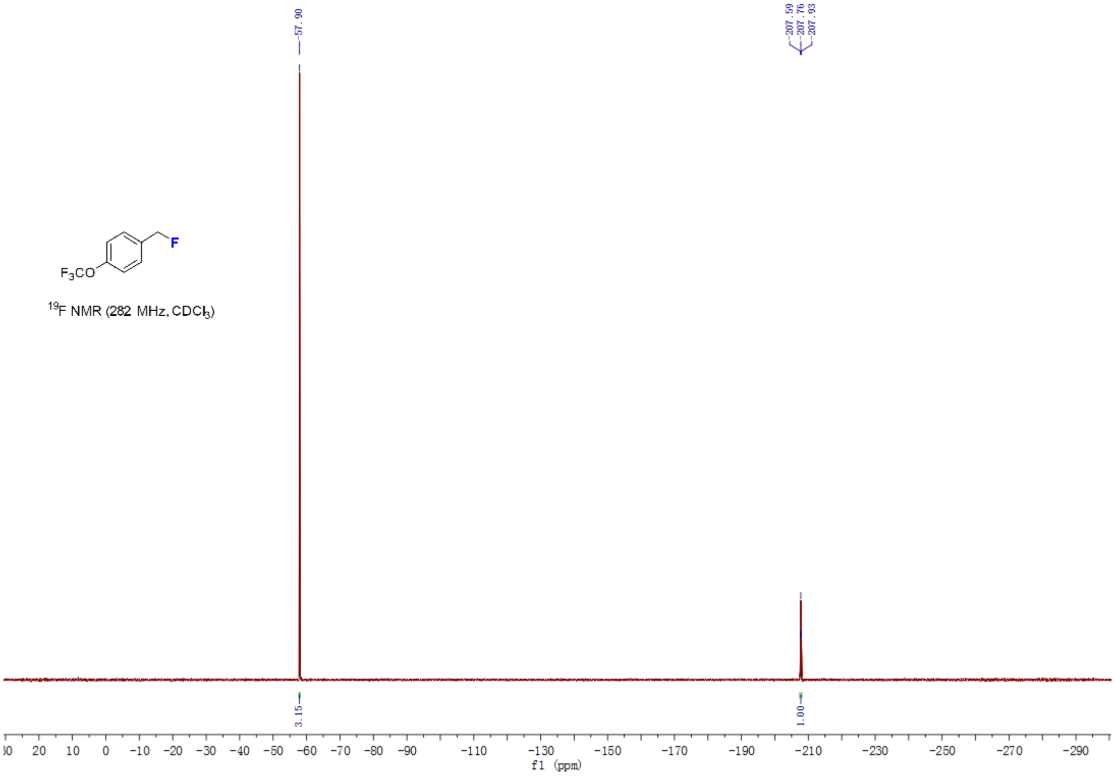

-57.90
-207.59
-207.76
-207.93
F
F3CO
19F NMR (282 MHz, CDCl3)
3.15
1.00
f1 (ppm)

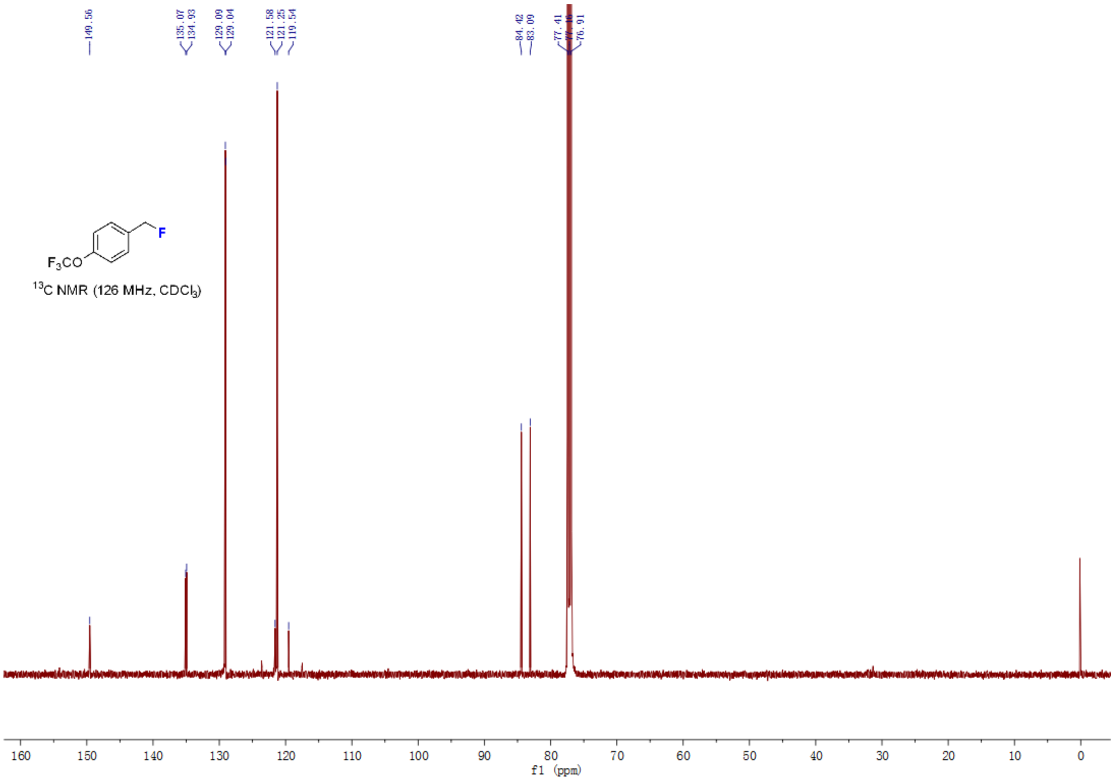


**Supplementary Figure 32**. NMR Spectra of 1-(fluoromethyl)-4-(trifluoromethoxy)benzene

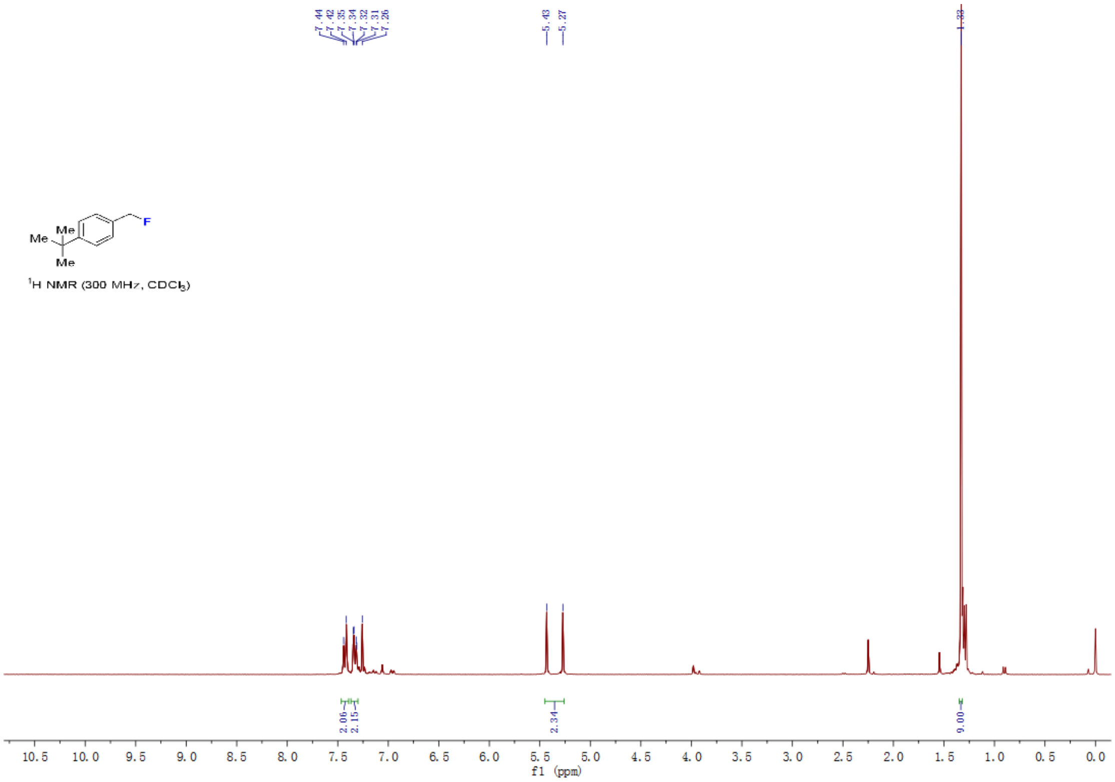


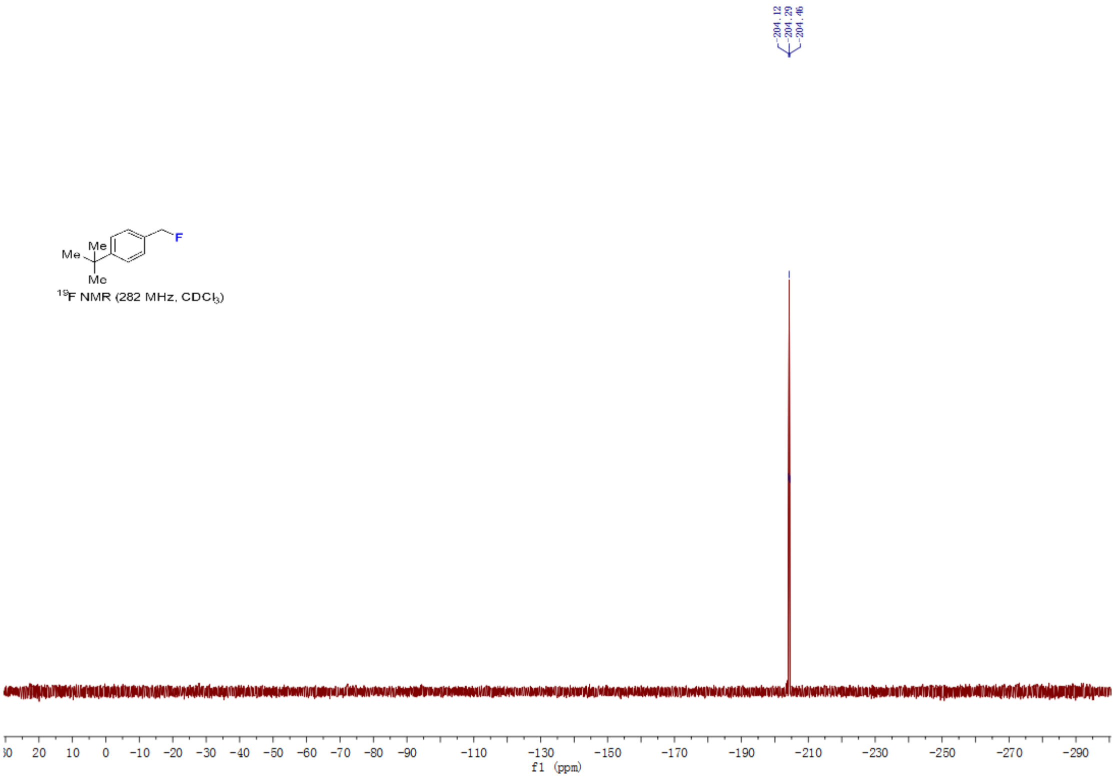


**Supplementary Figure 32**. NMR Spectra of 1-(*tert*-butyl)-4-(fluoromethyl)benzene.

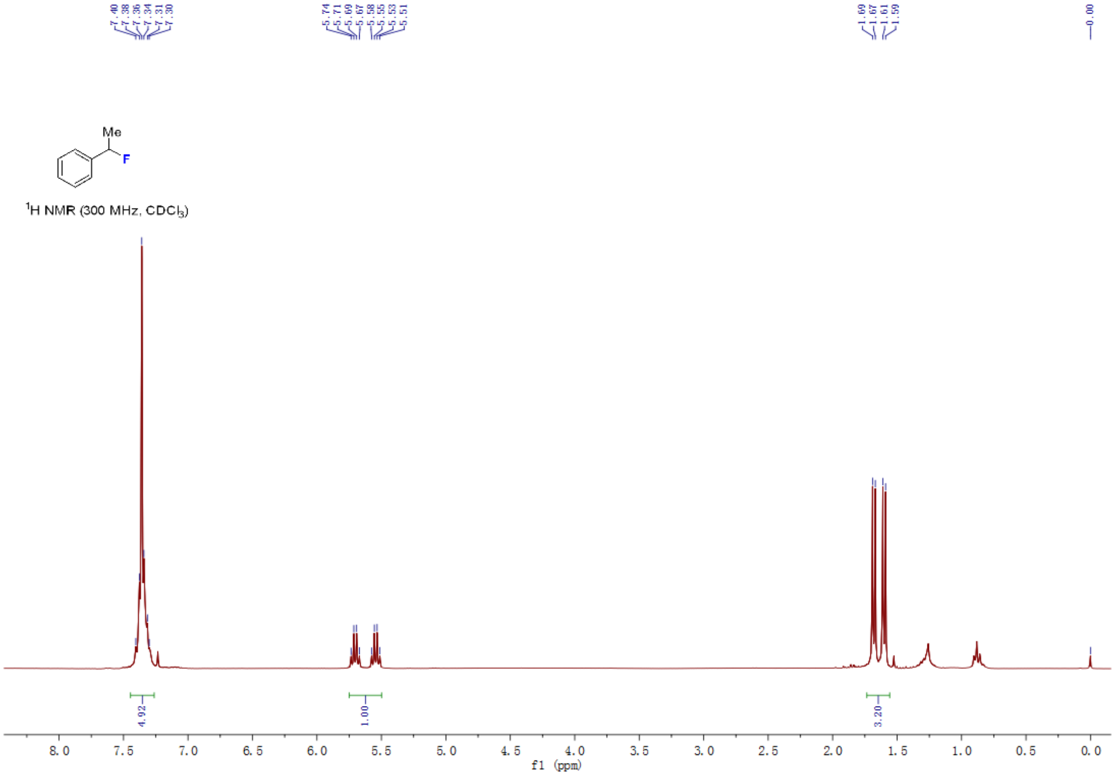


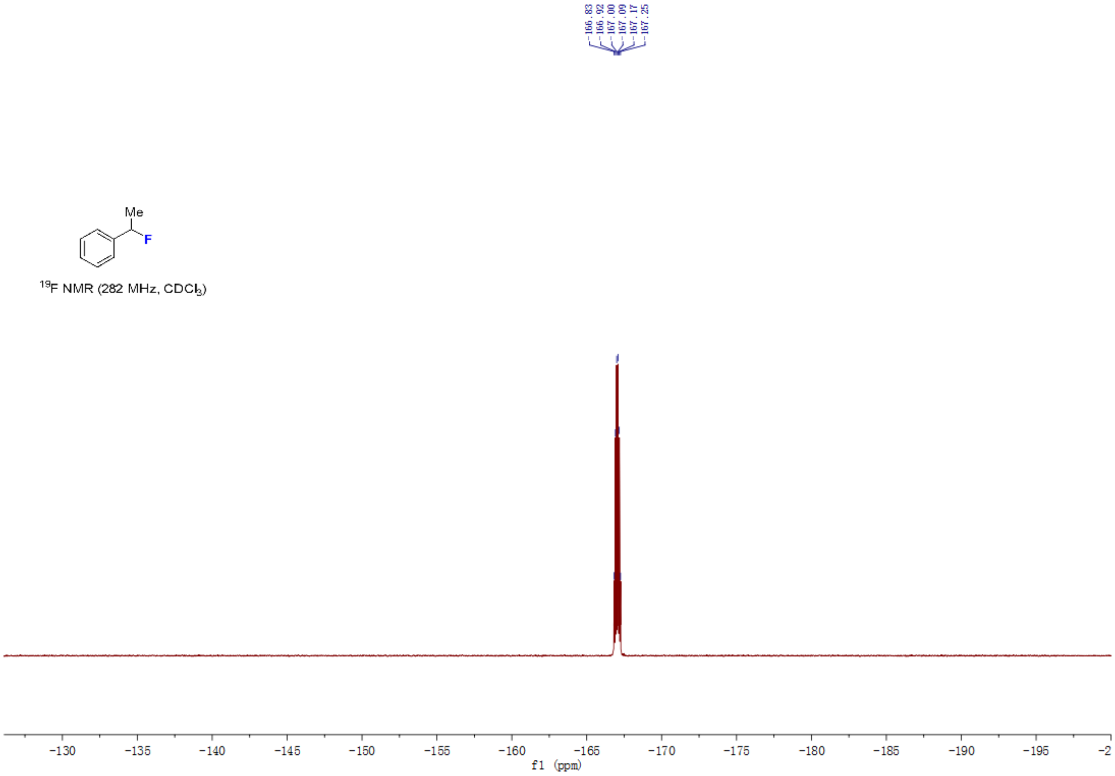


**Supplementary Figure 33**. NMR Spectra of (1-fluoroethyl)benzene.

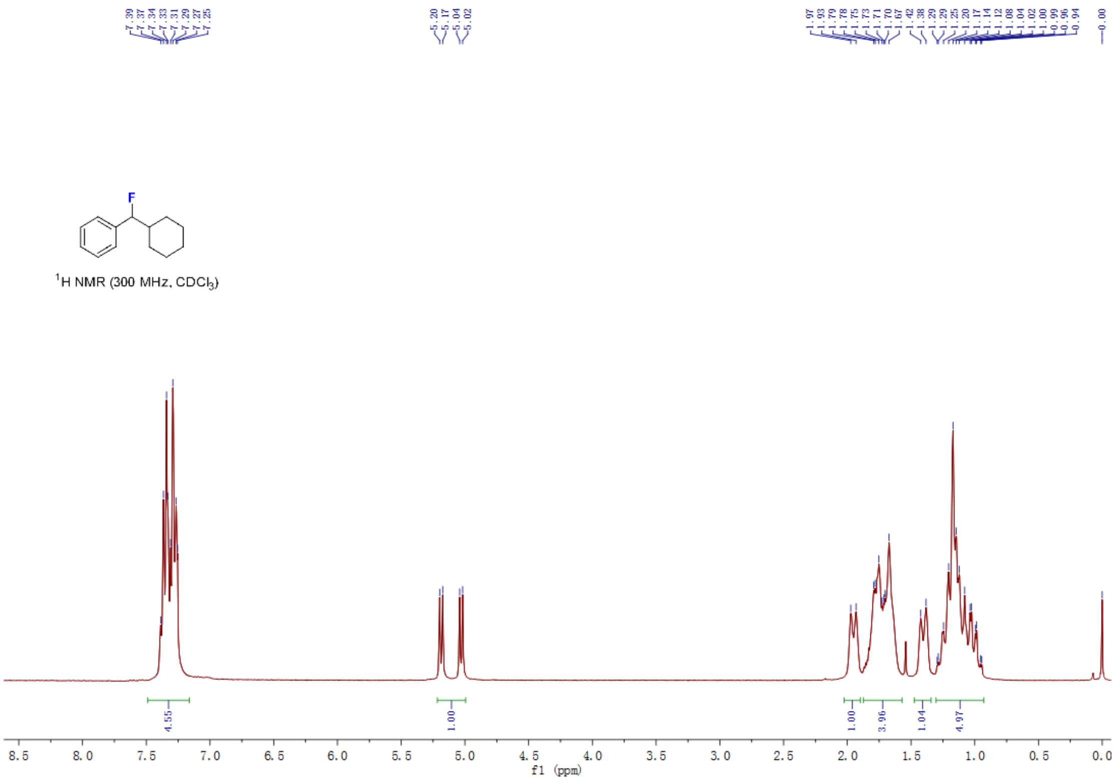

F
1H NMR (300 MHz, CDCl3)
f1 (ppm)


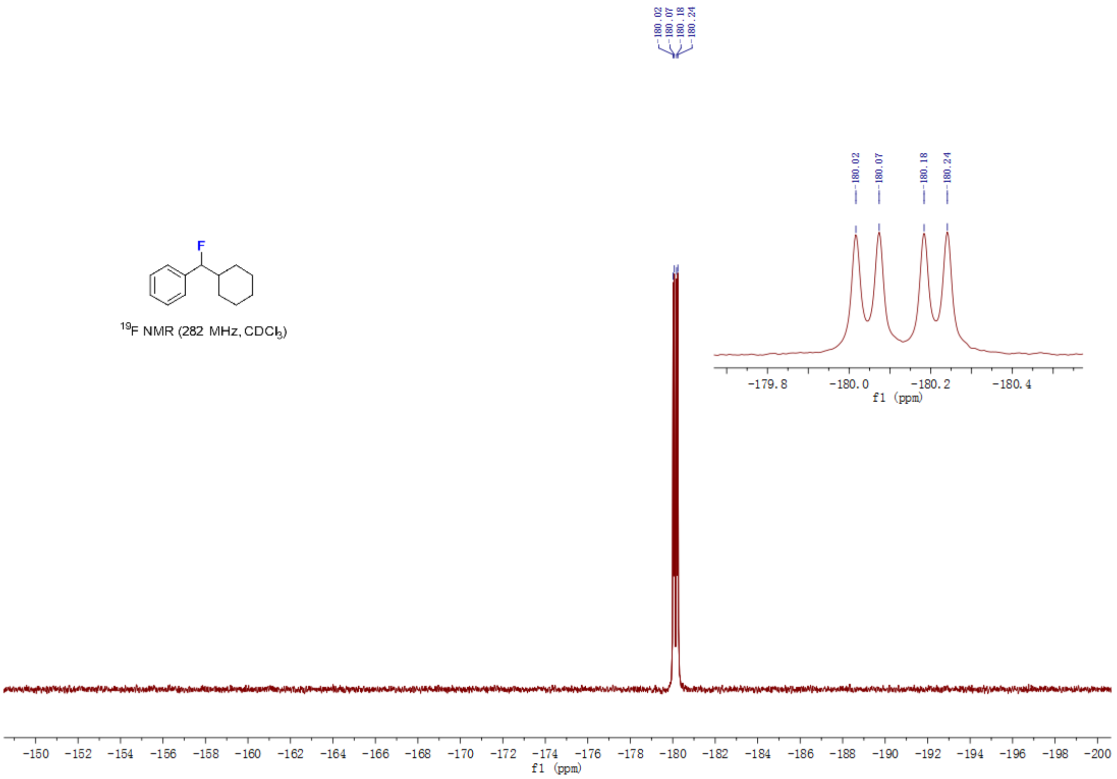

F
19F NMR (282 MHz, CDCl3)
-180.02
-180.07
-180.18
-180.24
f1 (ppm)

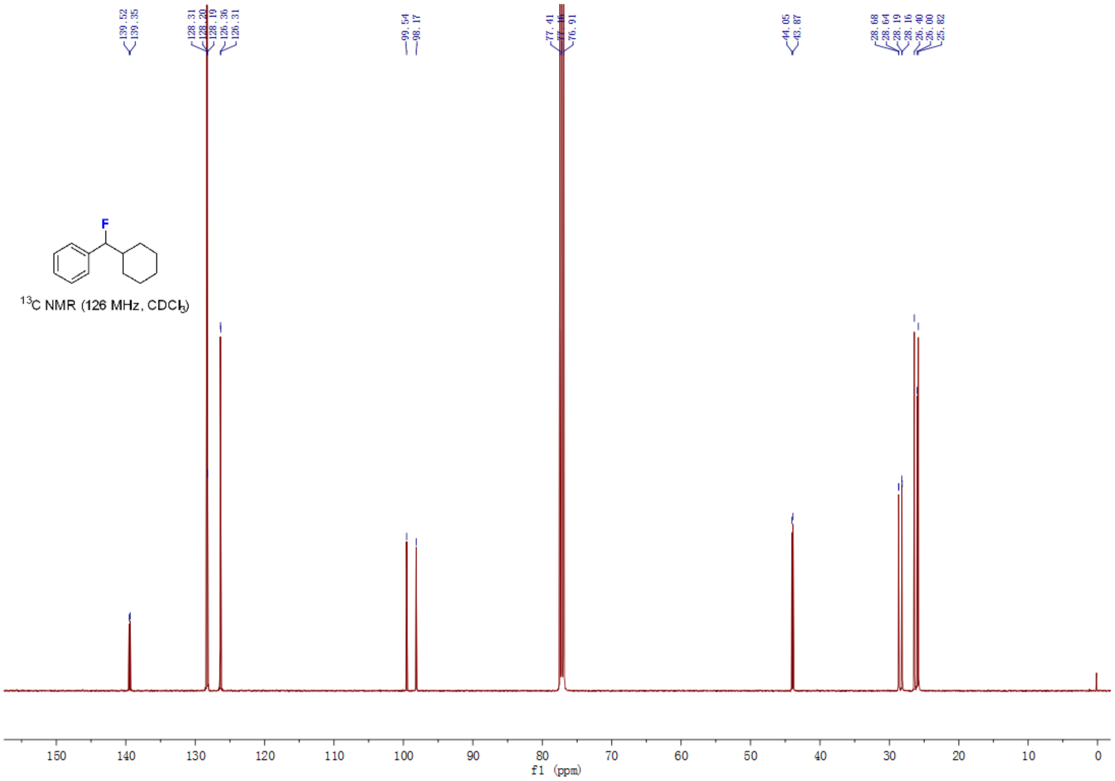


**Supplementary Figure 34**. NMR Spectra of (cyclohexylfluoromethyl)benzene.

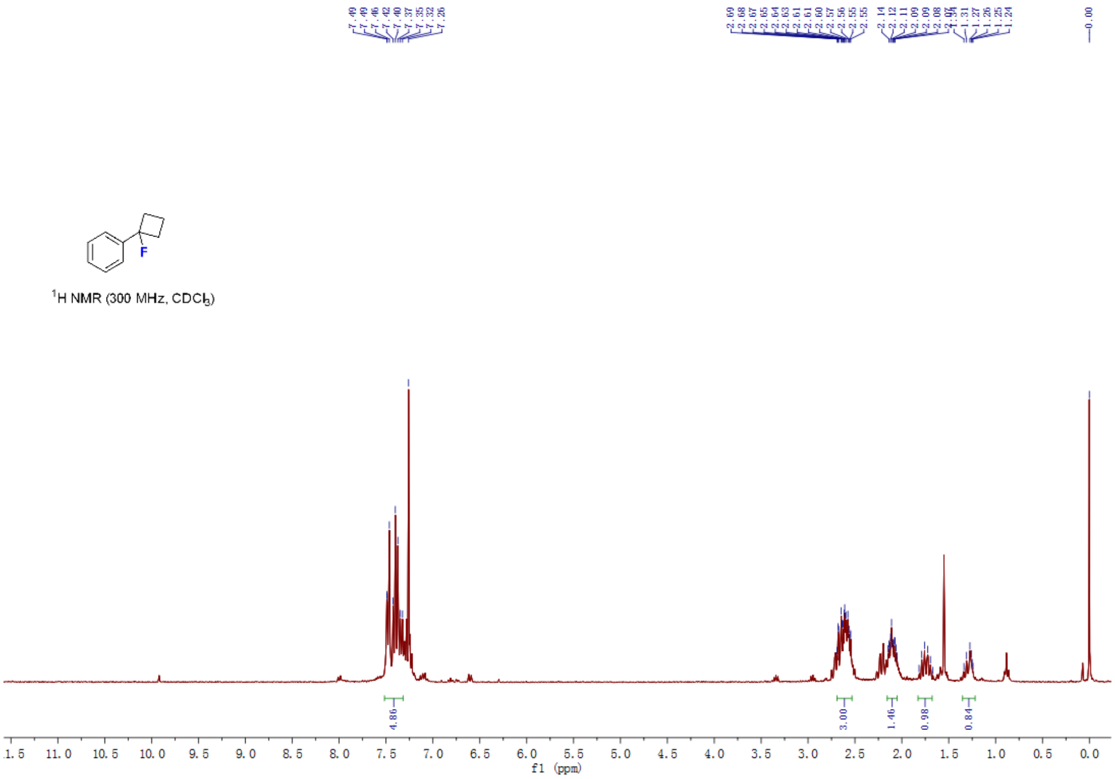


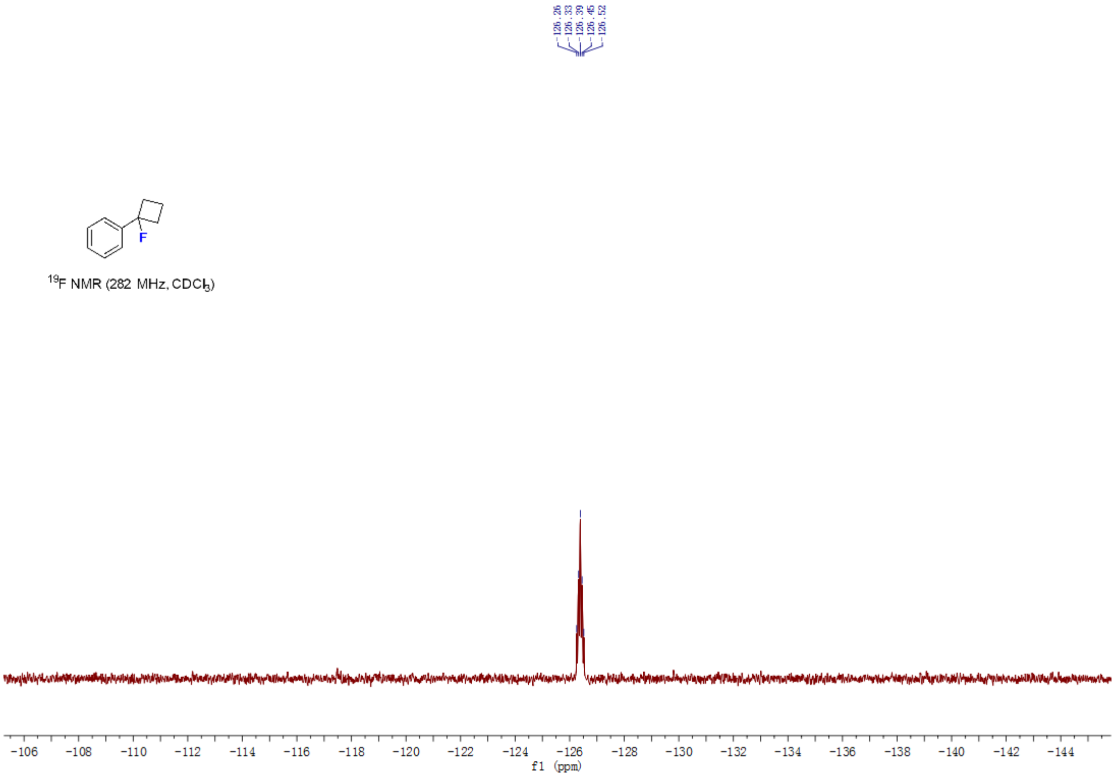


**Supplementary Figure 35**. NMR Spectra of (1-fluorocyclobutyl)benzene.

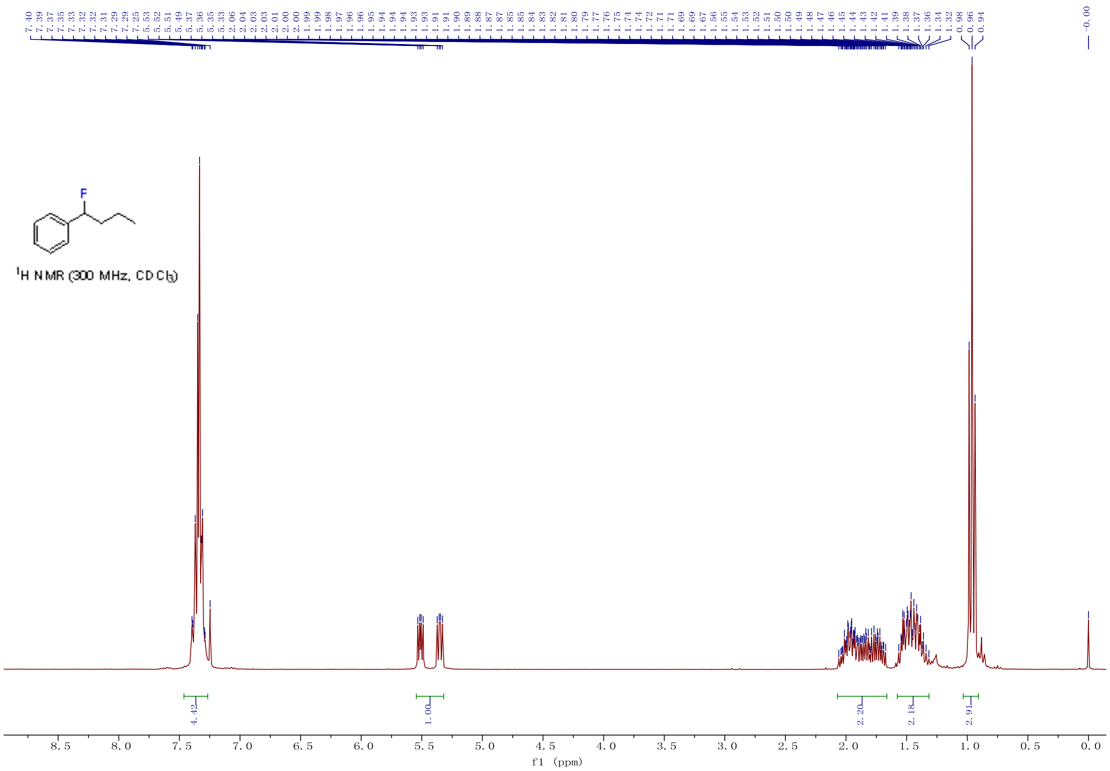
F
¹H NMR (300 MHz, CDCl3)
f1 (ppm)

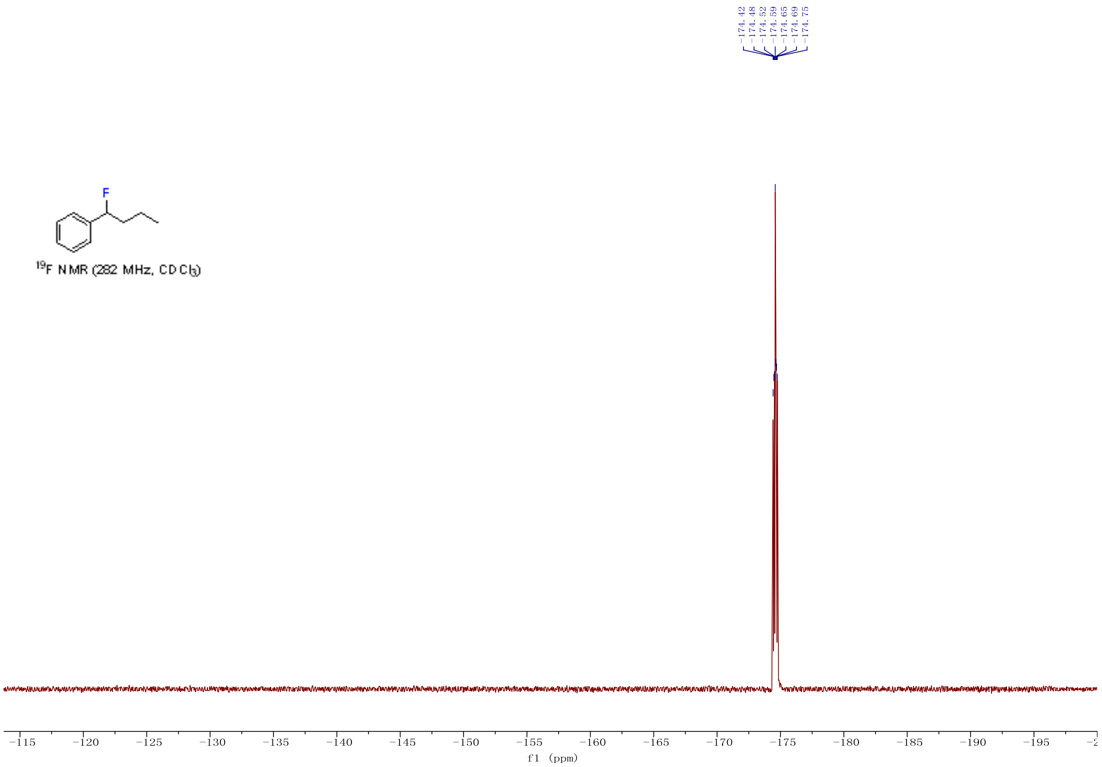
F
¹⁹F NMR (282 MHz, CDCl3)
f1 (ppm)

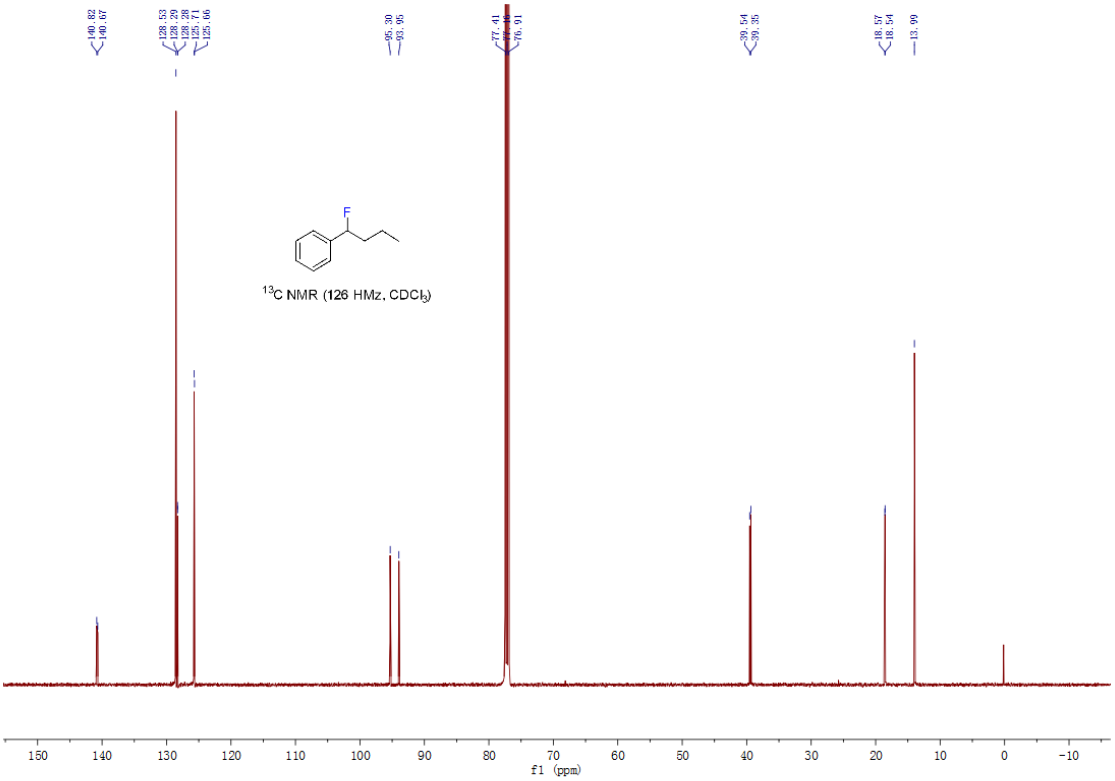

**Supplementary Figure 36**. NMR Spectra of (1-fluorobutyl)benzene.

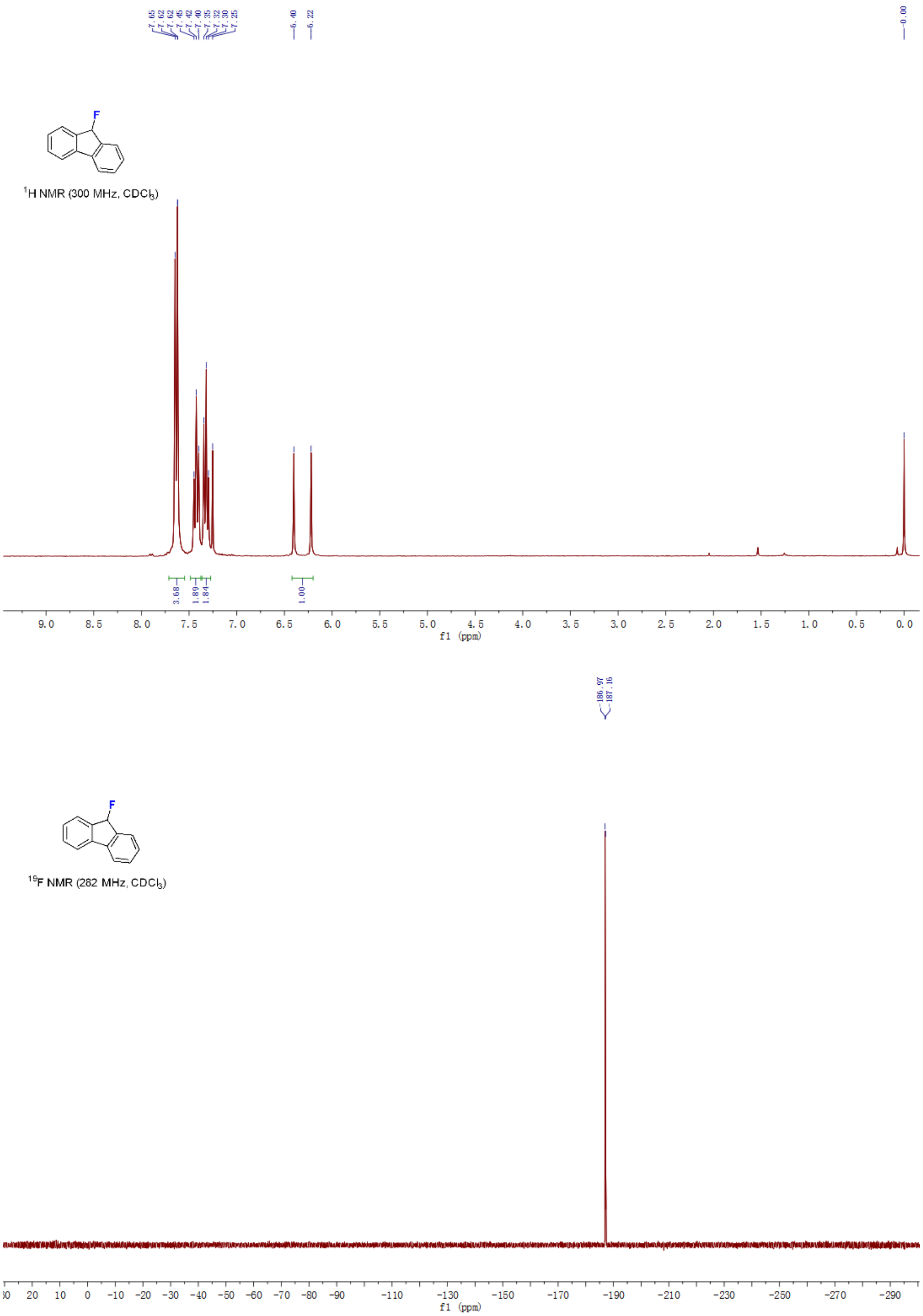


**Supplementary Figure 37**. NMR Spectra of 9-fluoro-9*H*-fluorene.

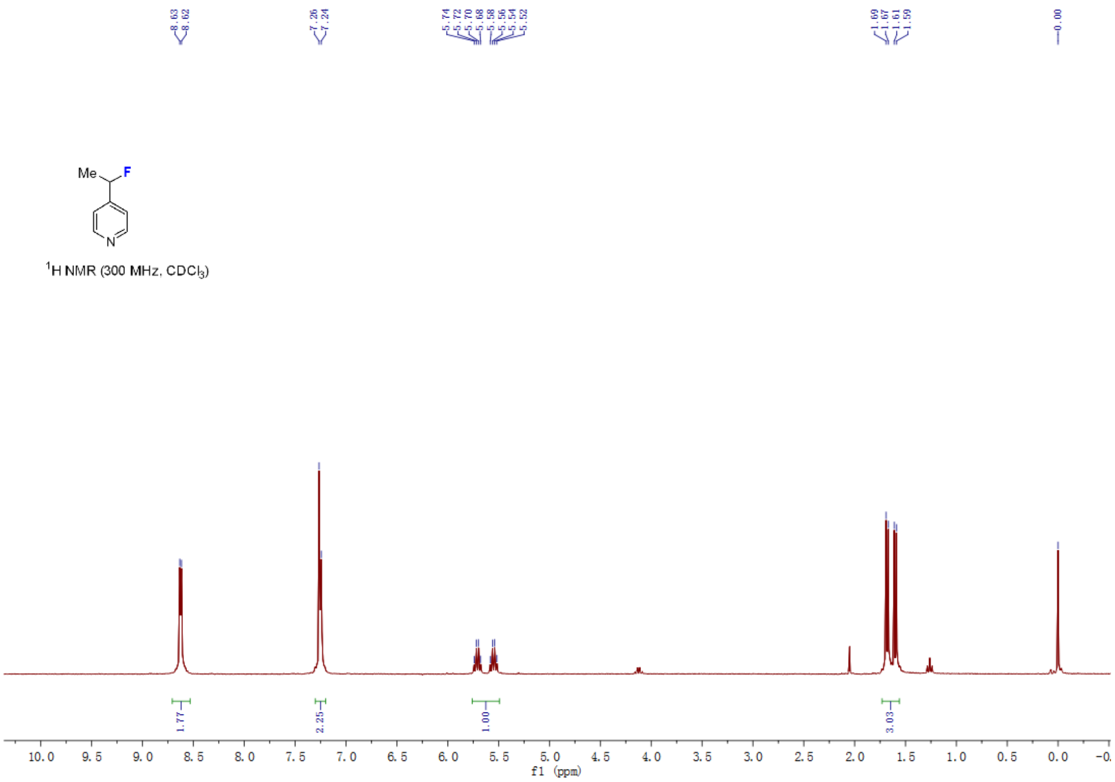


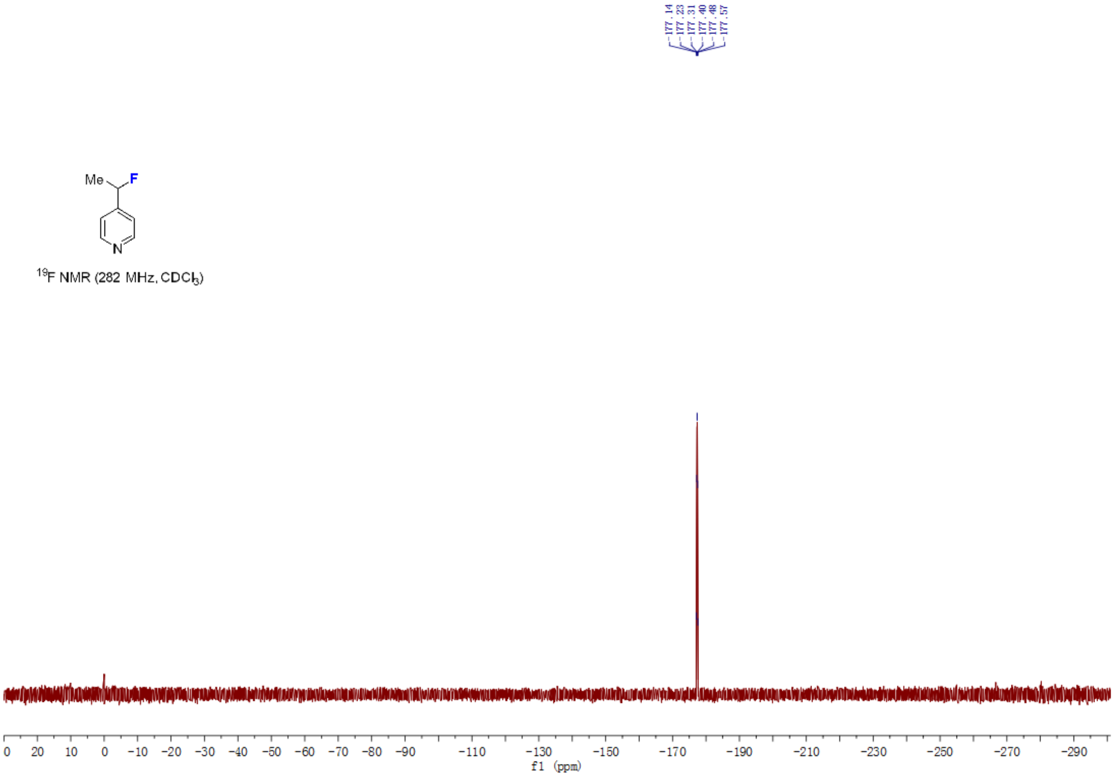


**Supplementary Figure 38**. NMR Spectra of 4-(1-fluoroethyl)pyridine.

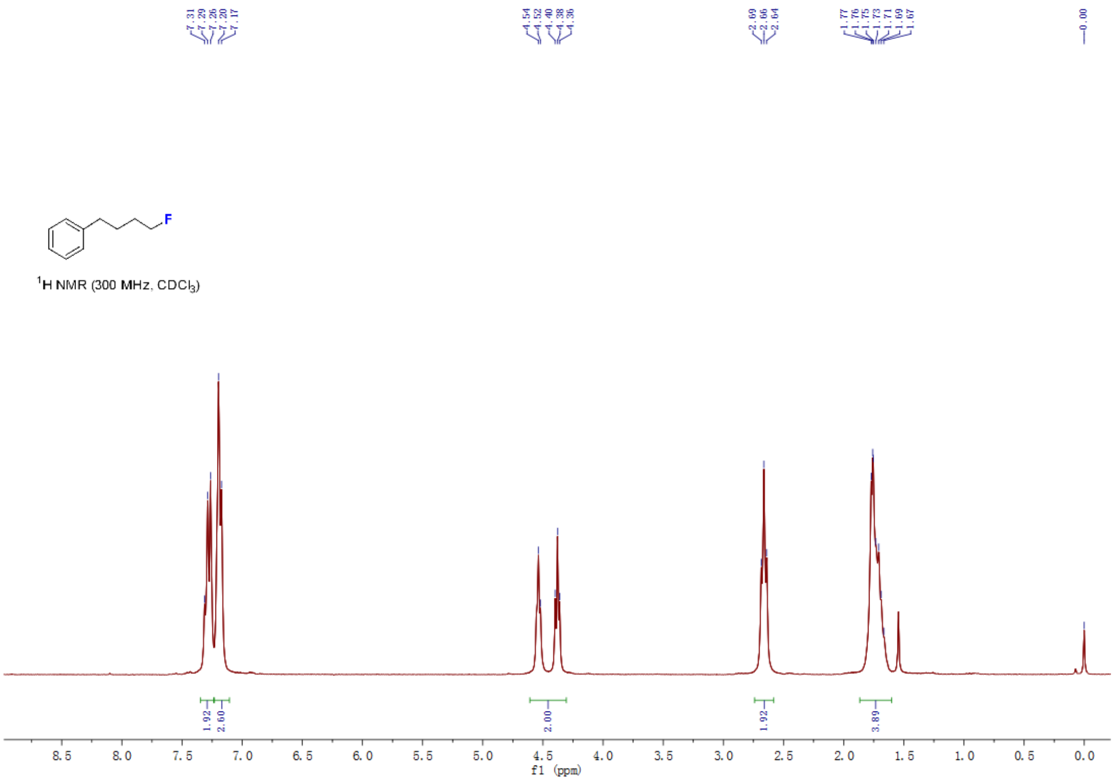


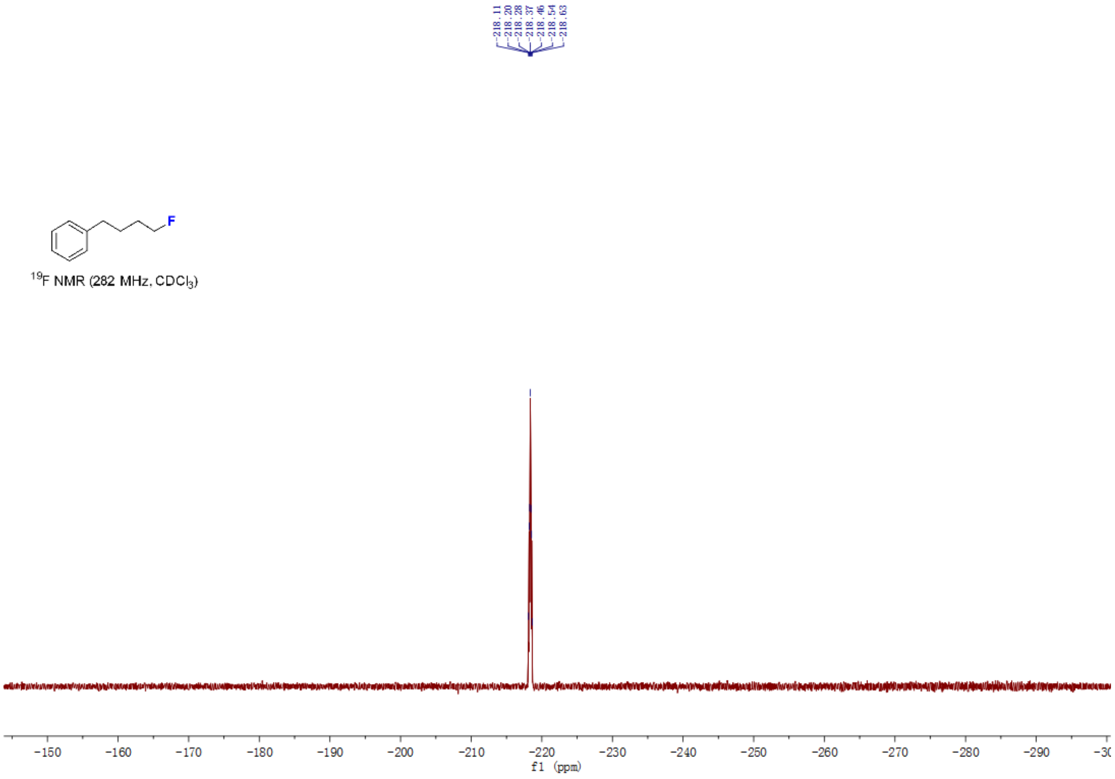


**Supplementary Figure 39**. NMR Spectra of (4-fluorobutyl)benzene.

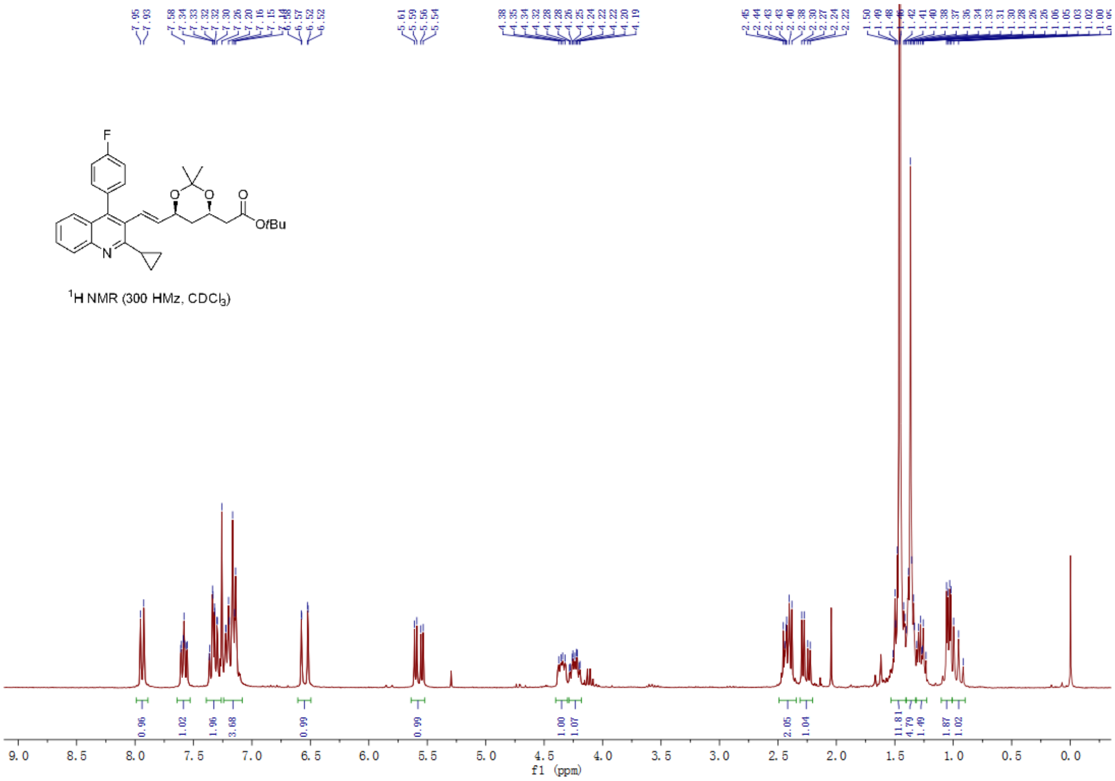
F
N
OtBu
1H NMR (300 HMz, CDCl3)
f1 (ppm)

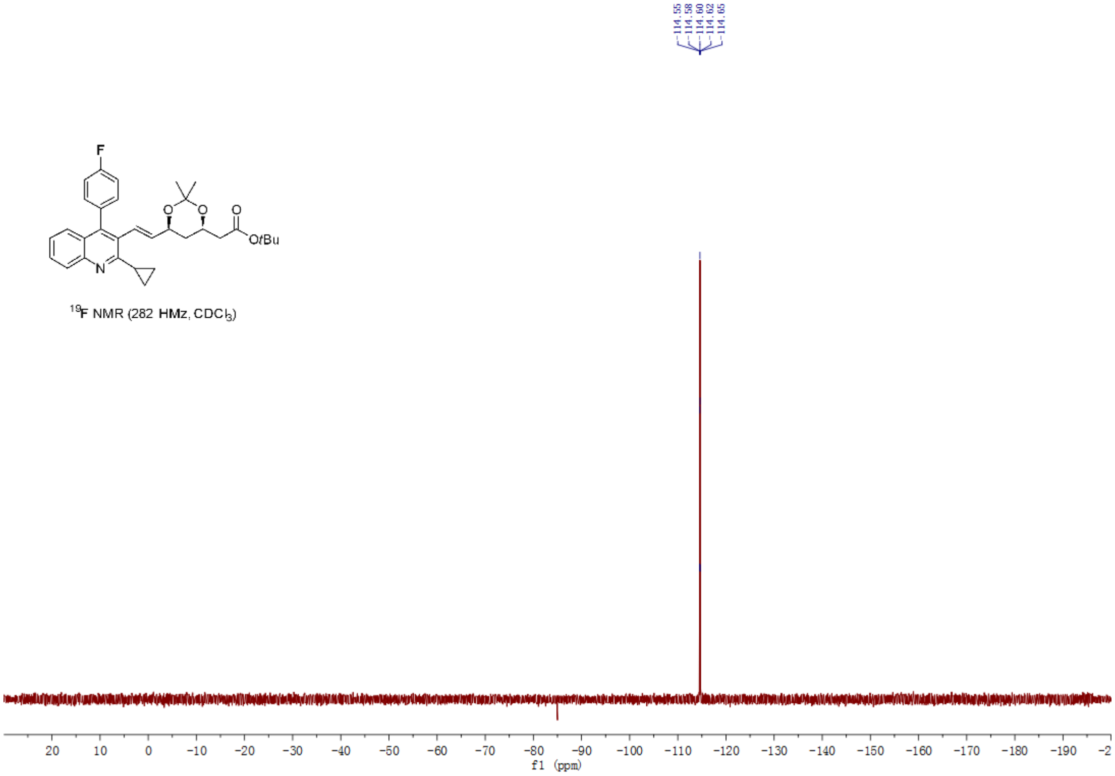
F
N
OtBu
19F NMR (282 HMz, CDCl3)
f1 (ppm)

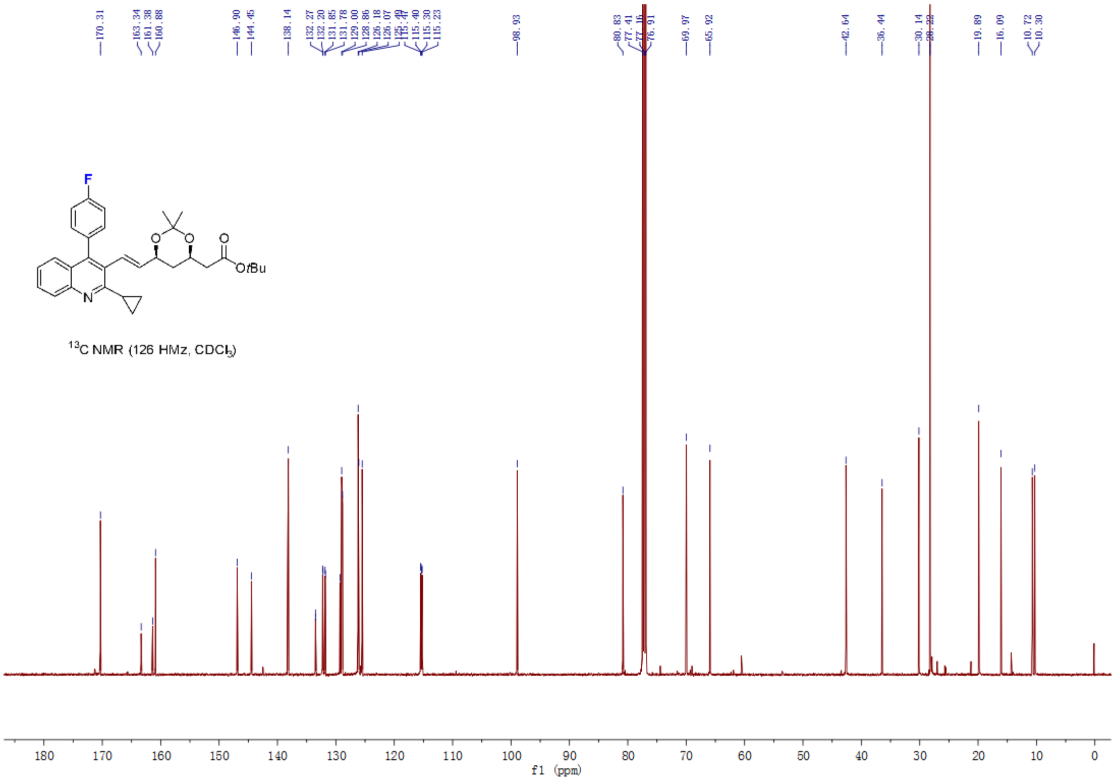


**Supplementary Figure 40**. NMR Spectra of *tert*-butyl 2-((4*R*,6*S*)-6-((*E*)-2-(2-cyclopropyl-4-(4-fluorophenyl)quinolin-3-yl)vinyl)-2,2-dimethyl-1,3-dioxan-4-yl)acetate.

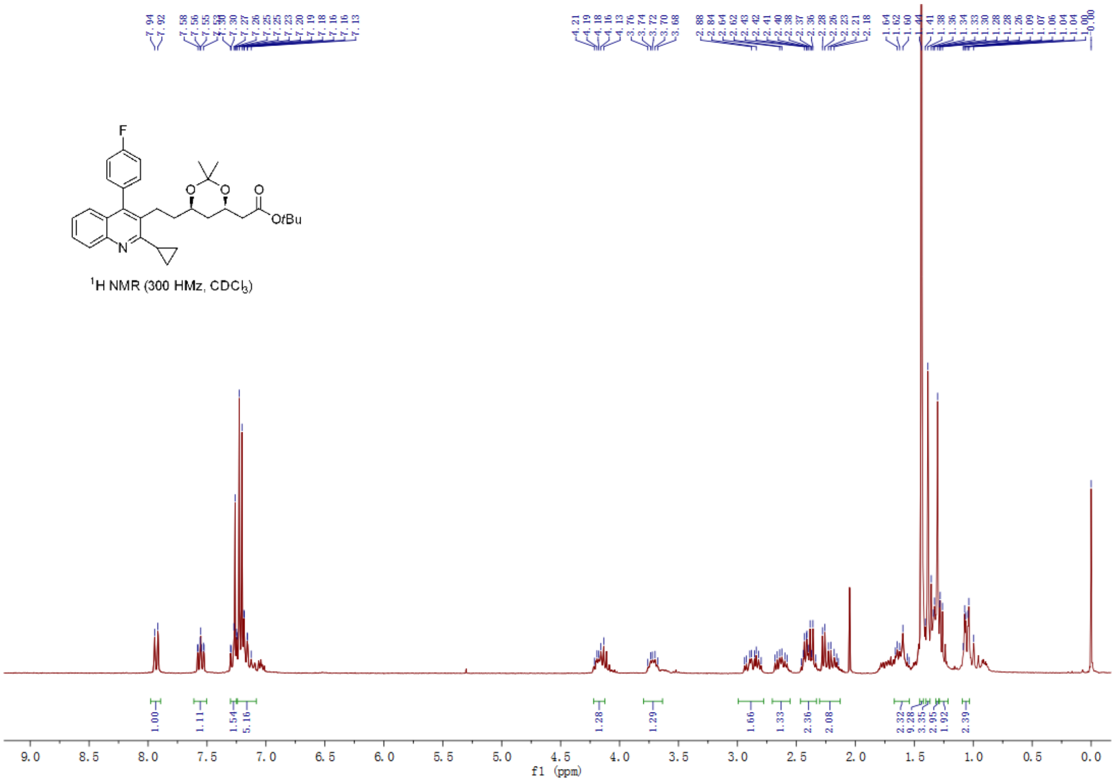

F
O
O
O
OtBu
N
¹H NMR (300 HMz, CDCl₃)
f1 (ppm)


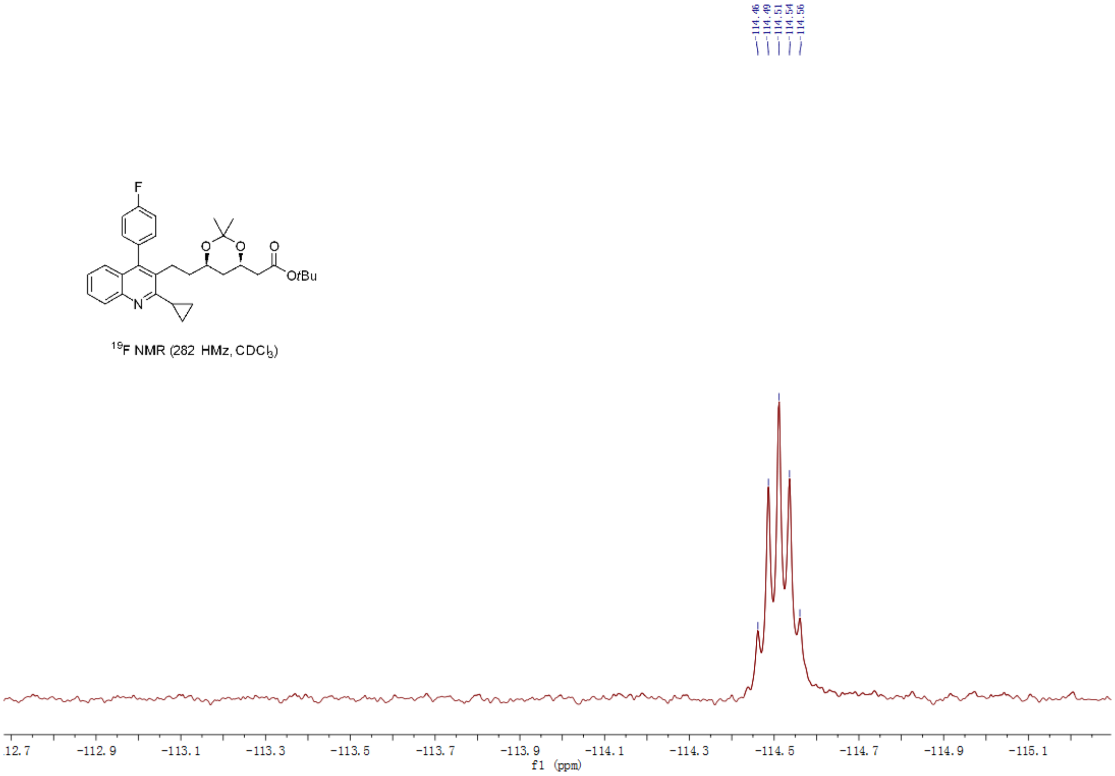

F
O
O
O
OtBu
N
¹⁹F NMR (282 HMz, CDCl₃)
f1 (ppm)

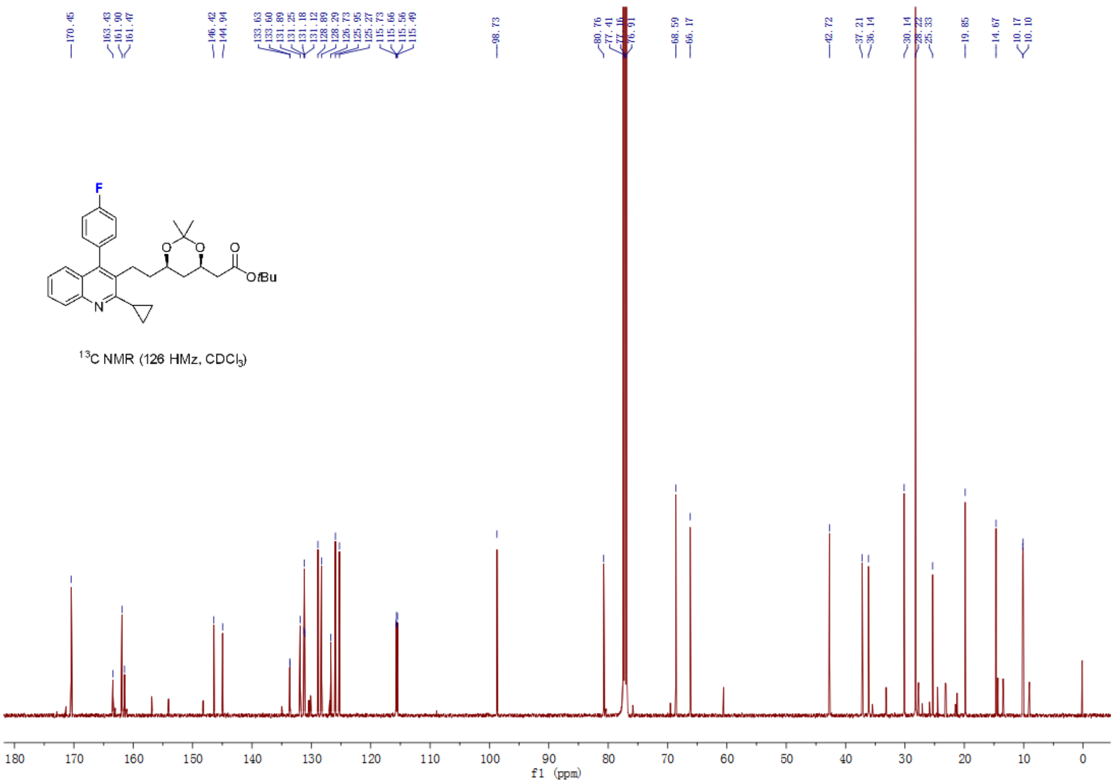

**Supplementary Figure 41**. NMR Spectra of *tert*-butyl 2-((4*R*,6*R*)-6-(2-(2-cyclopropyl-4-(4-fluorophenyl)quinolin-3-yl)ethyl)-2,2-dimethyl-1,3-dioxan-4-yl)acetate.

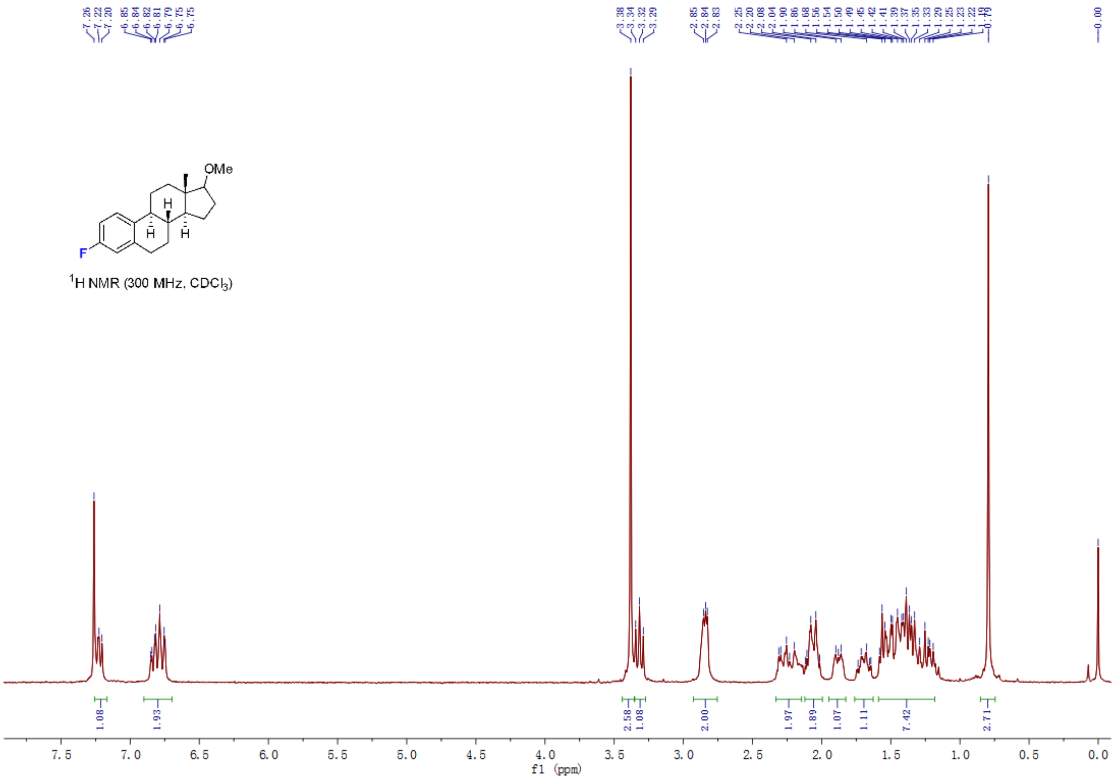
OMe
H
H
H
F
1H NMR (300 MHz, CDCl3)
f1 (ppm)

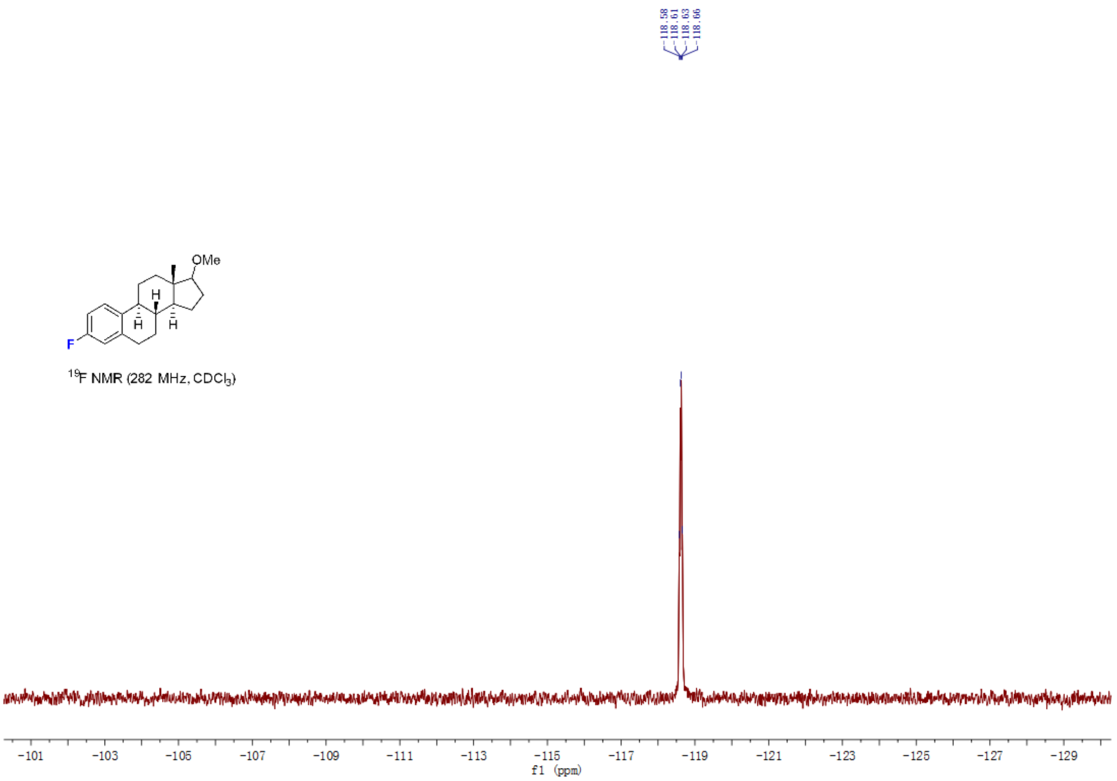
OMe
H
H
H
F
19F NMR (282 MHz, CDCl3)
f1 (ppm)

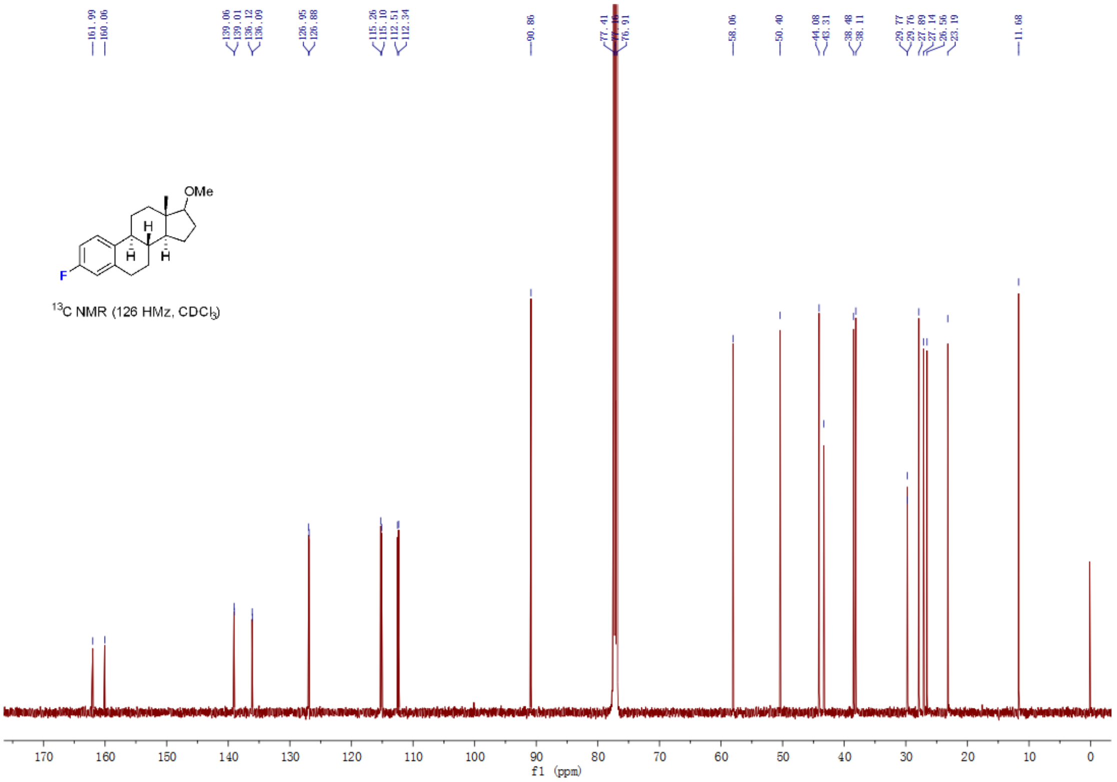


**Supplementary Figure 42**. NMR Spectra of 3-Fluoro-17-methoxyl-estra-1,3,5(10)-triene.

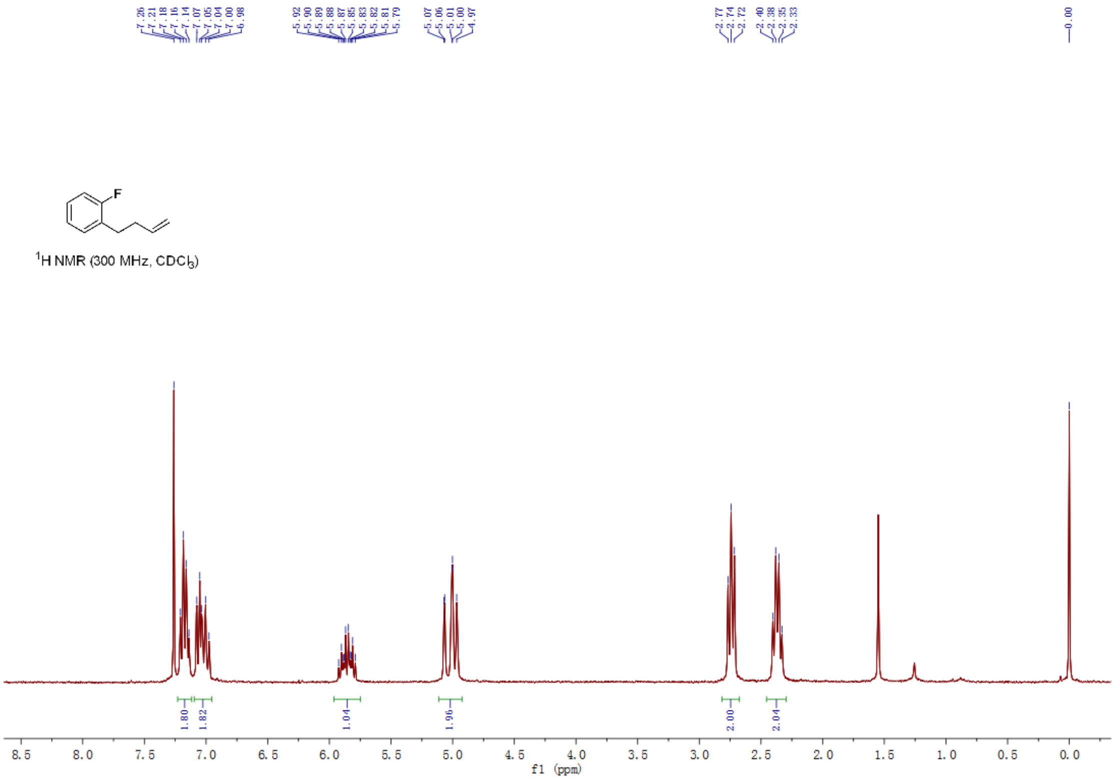


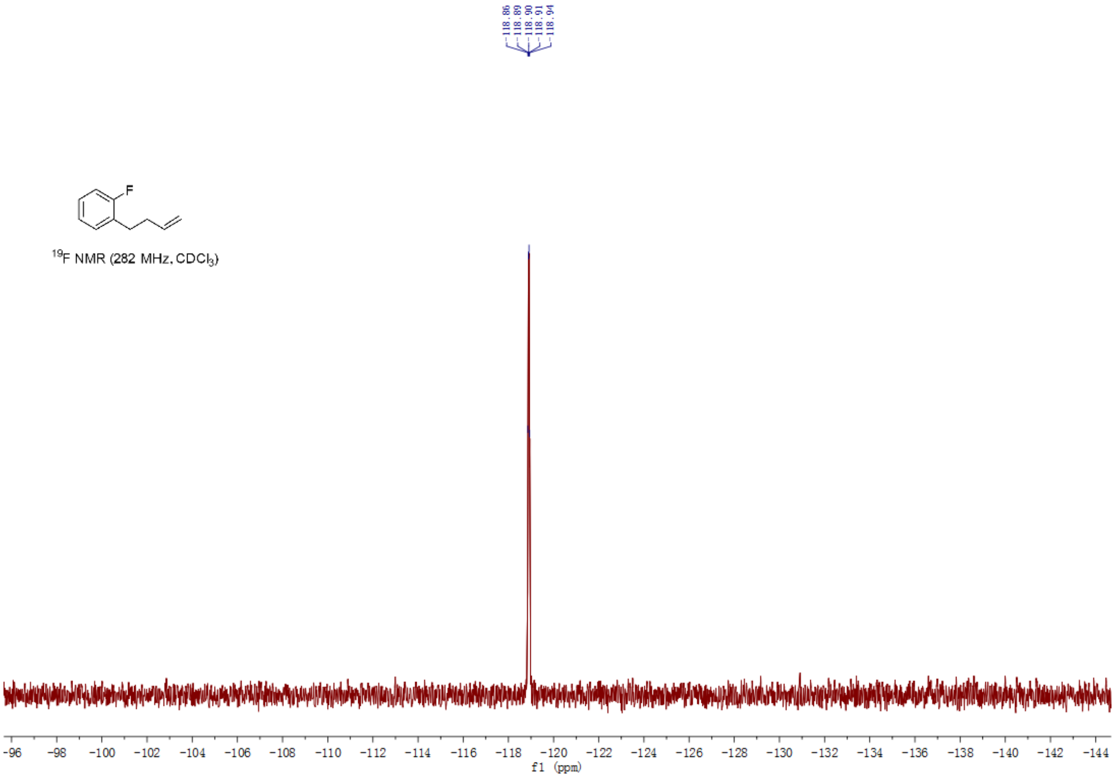


**Supplementary Figure 43**. NMR Spectra of 1-(but-3-en-1-yl)-2-fluorobenzene

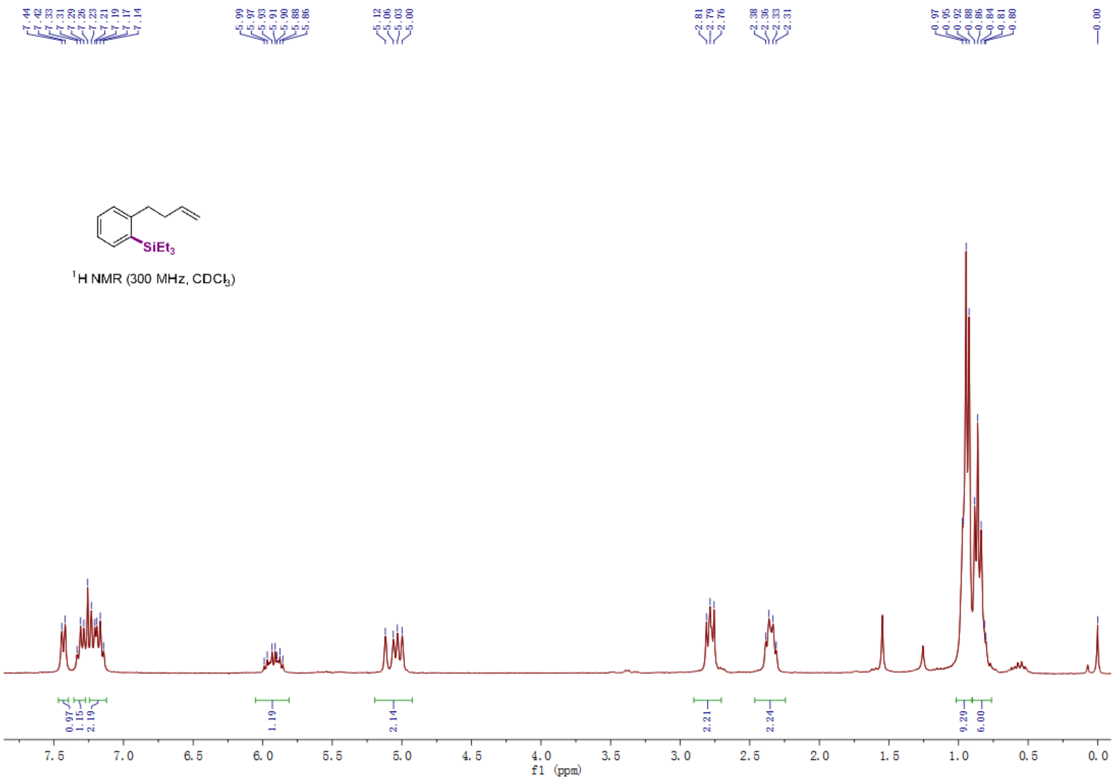


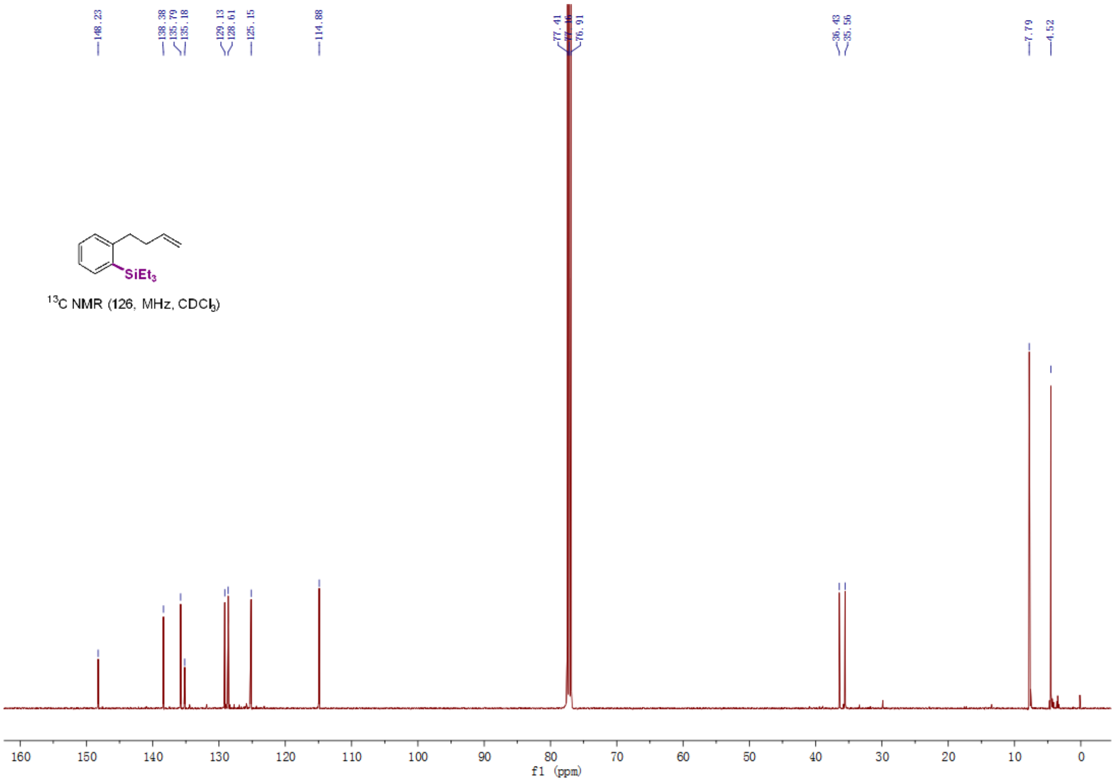


**Supplementary Figure 44**. NMR Spectra of (2-(but-3-en-1-yl)phenyl)triethylsilane.

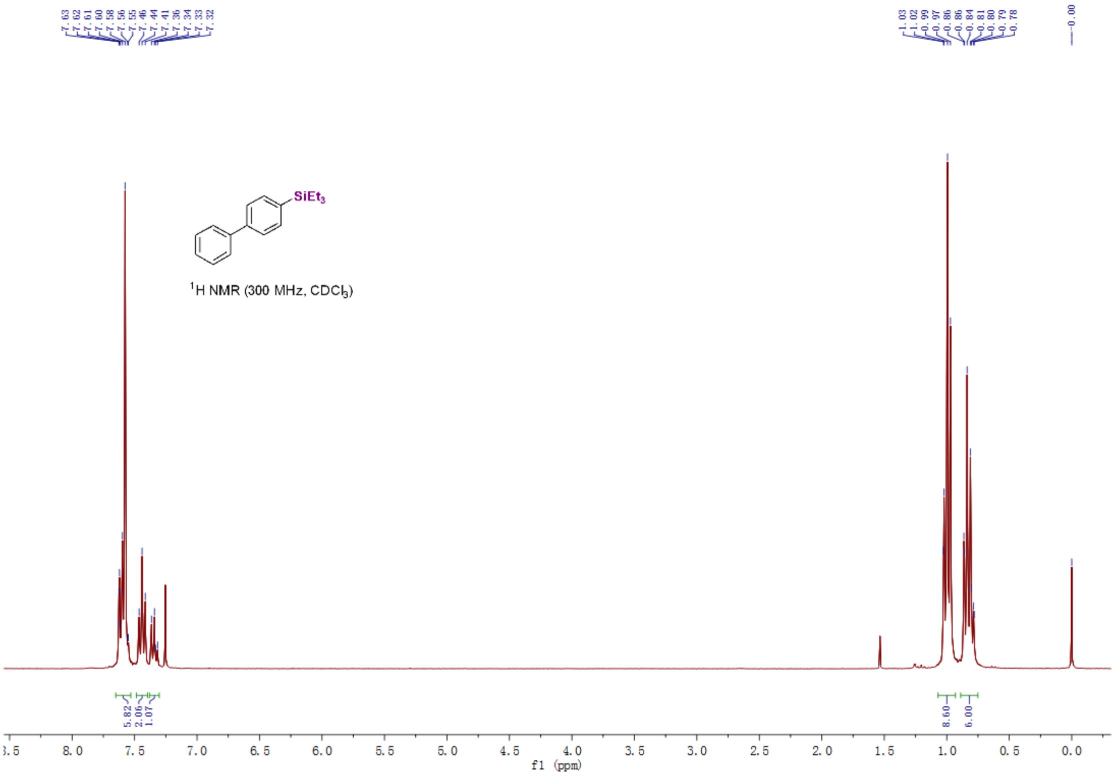


**Supplementary Figure 45**. NMR Spectra of [1,1'-Biphenyl]-4-yltriethylsilane.

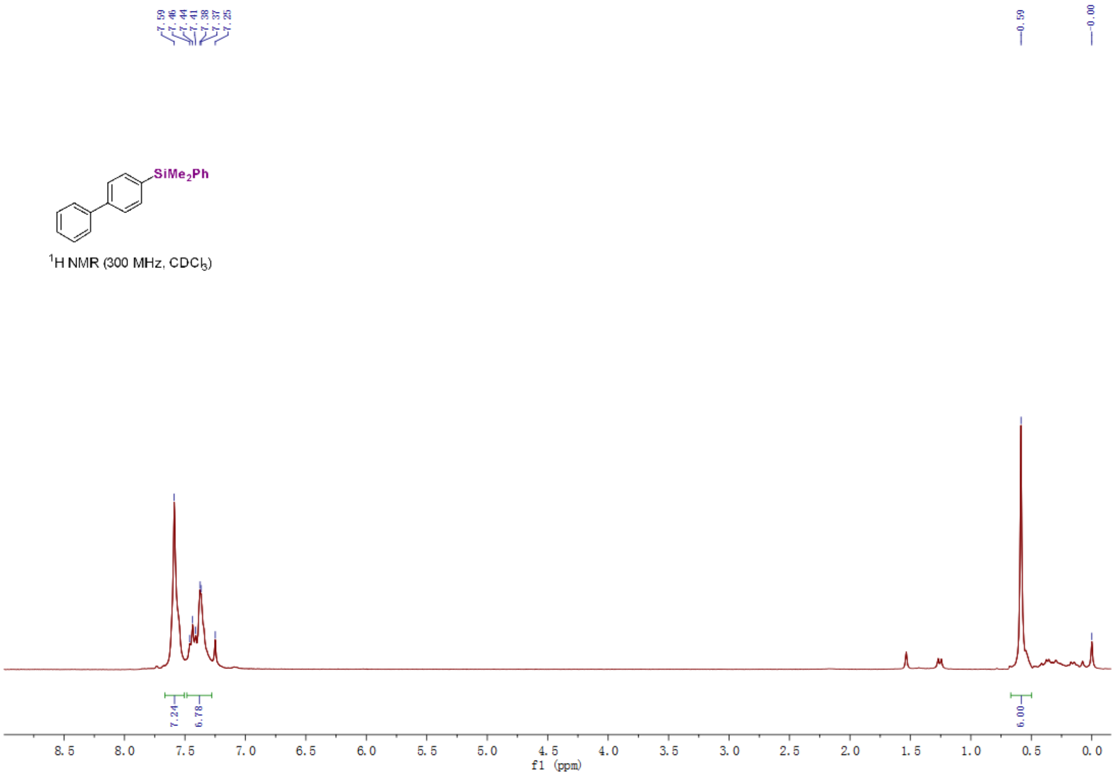


**Supplementary Figure 46**. NMR Spectra of [1,1'-biphenyl]-4-yldimethyl(phenyl)silane.

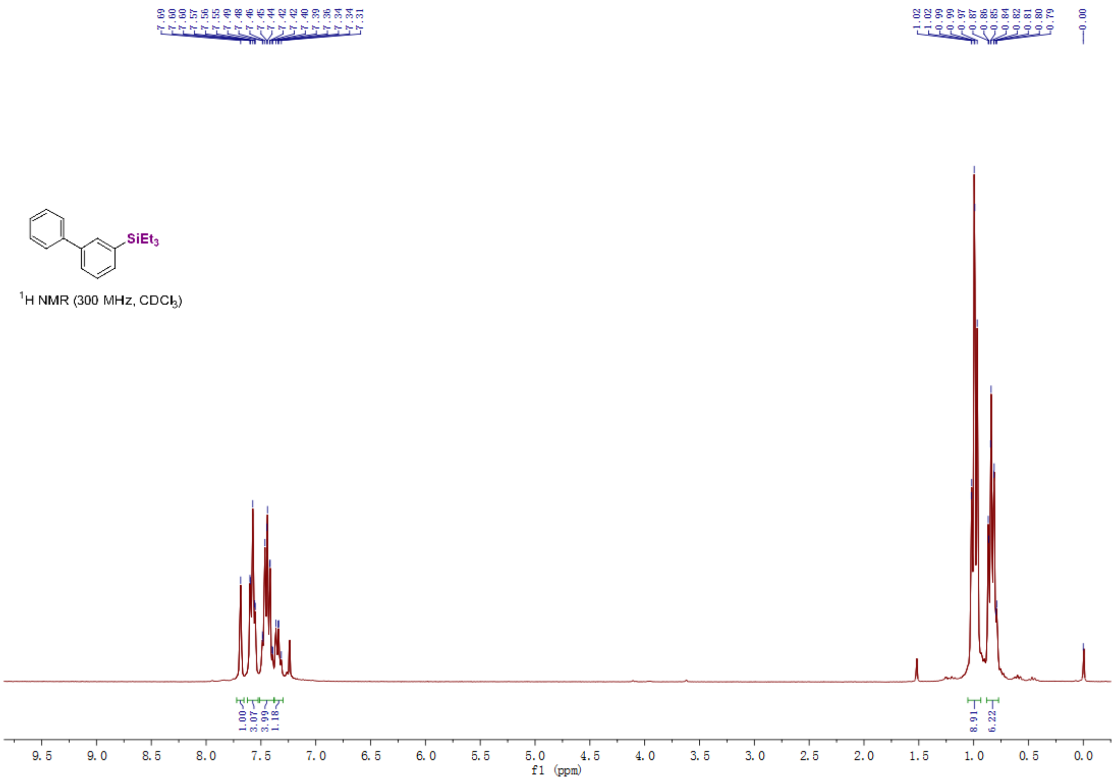


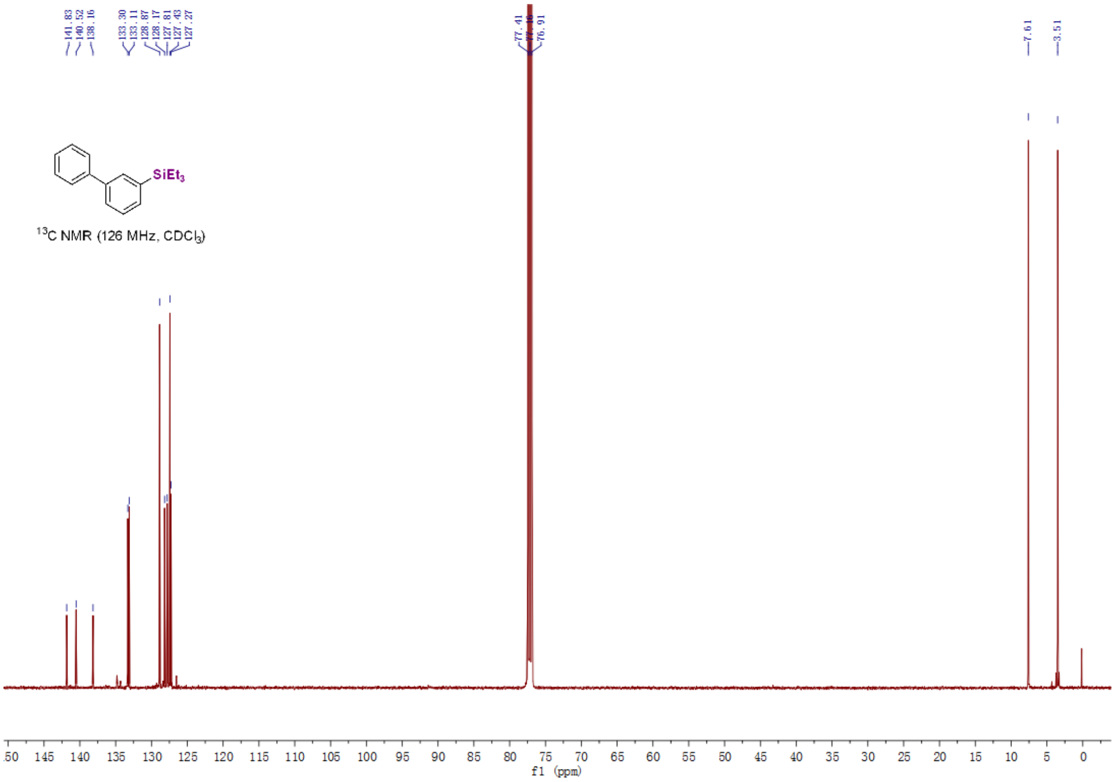


**Supplementary Figure 47**. NMR Spectra of [1,1'-biphenyl]-3-yltriethylsilane.

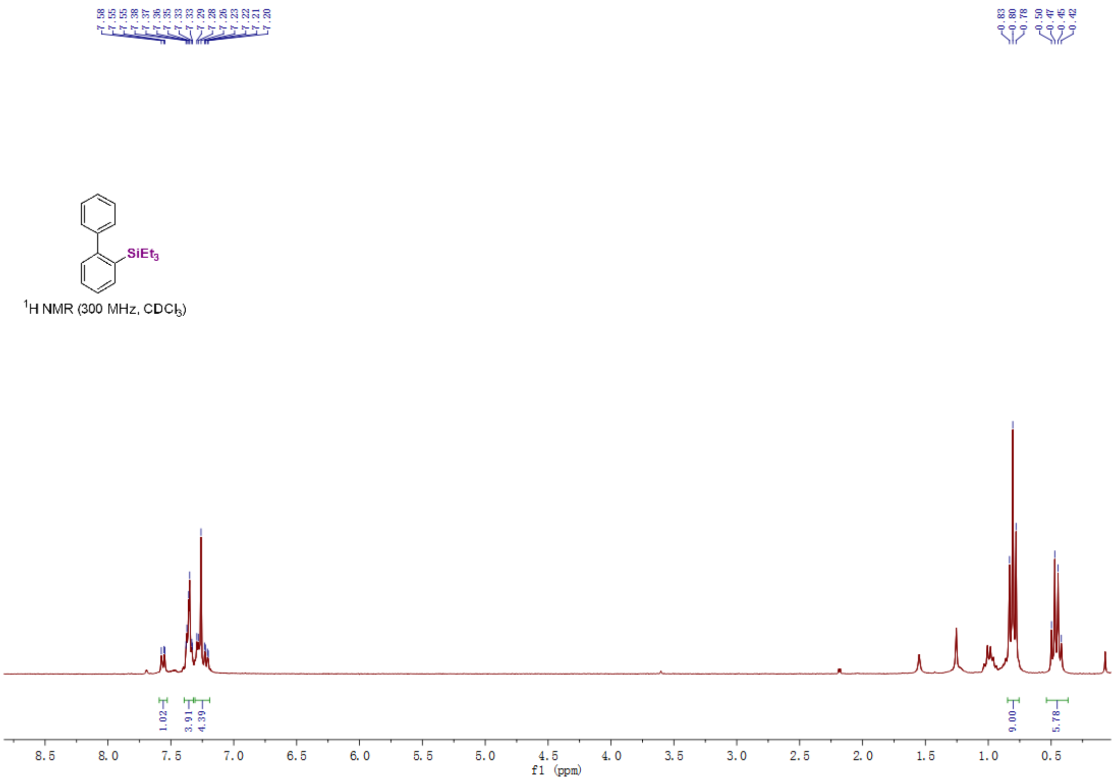


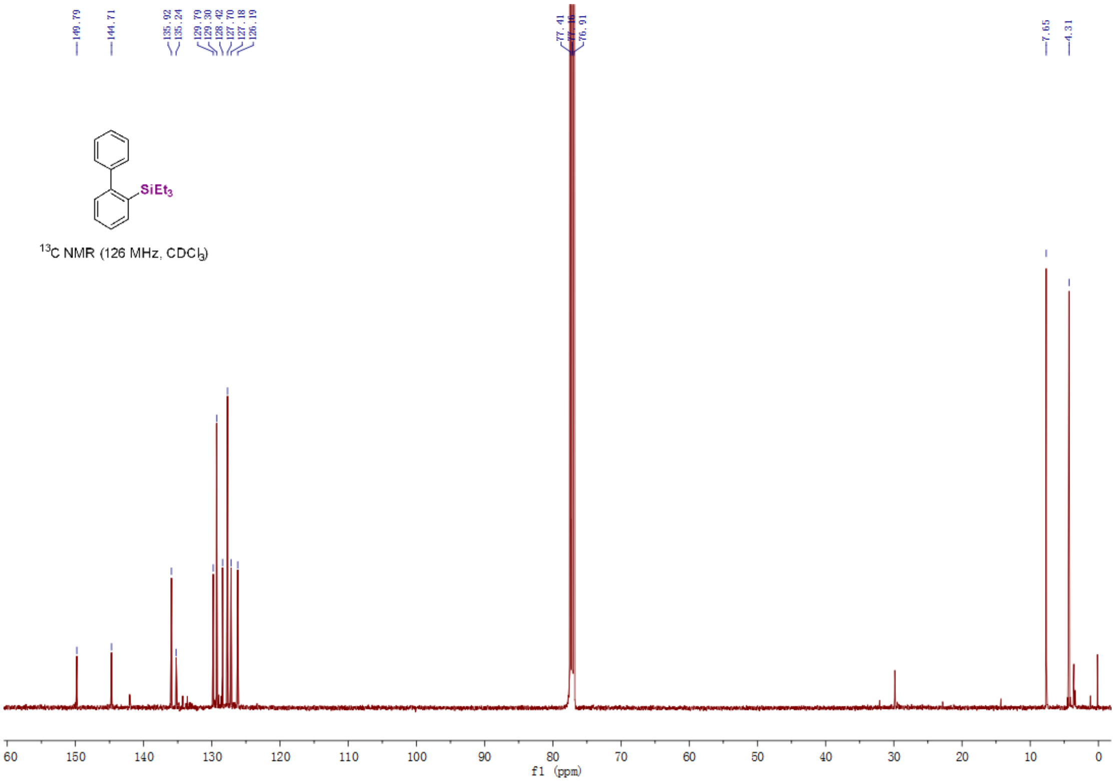


**Supplementary Figure 48**. NMR Spectra of [1,1'-biphenyl]-2-yltriethylsilane.

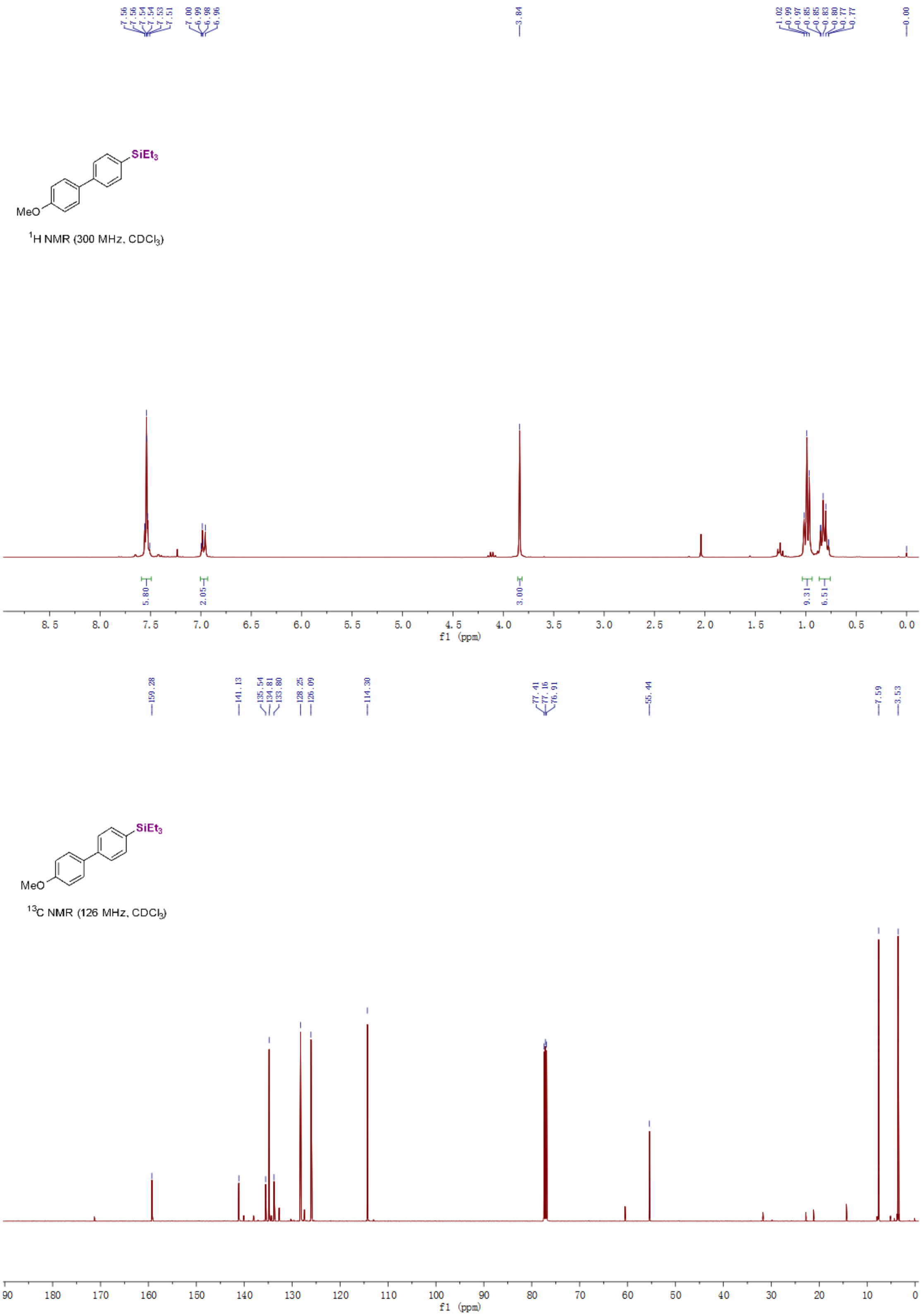


**Supplementary Figure 49**. NMR Spectra of triethyl(4'-methoxy-[1,1'-biphenyl]-4-yl)silane.

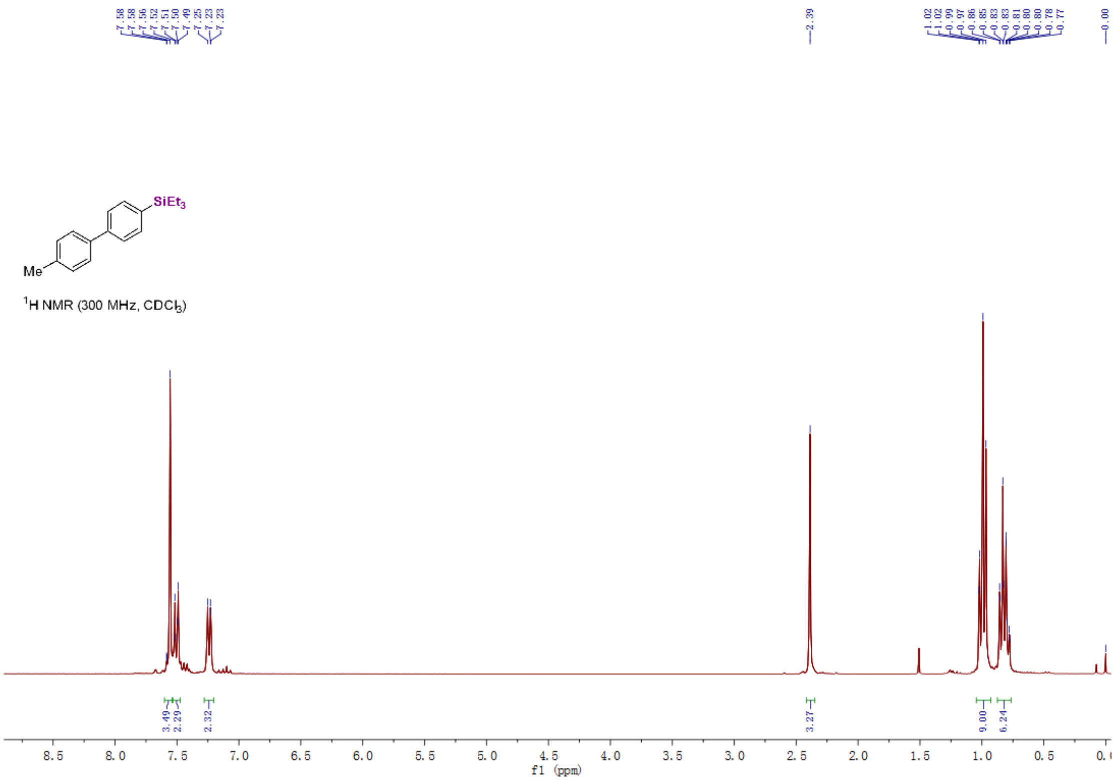


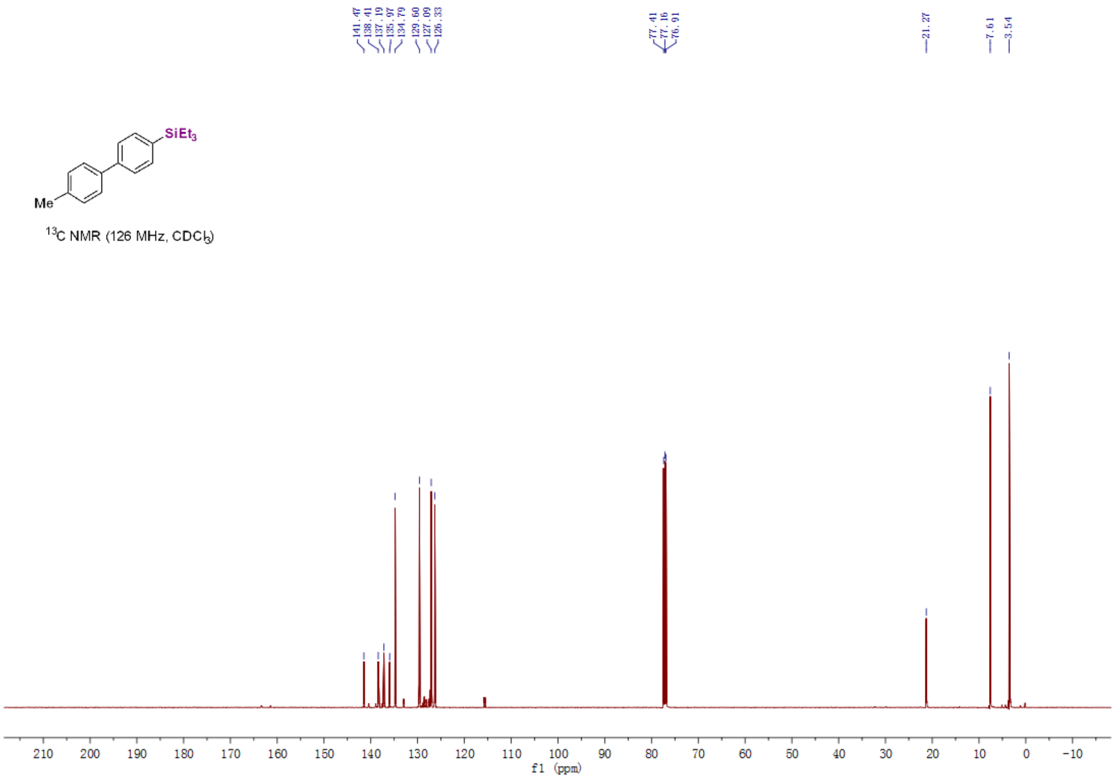


**Supplementary Figure 50**. NMR Spectra of triethyl(4'-methyl-[1,1'-biphenyl]-4-yl)silane.

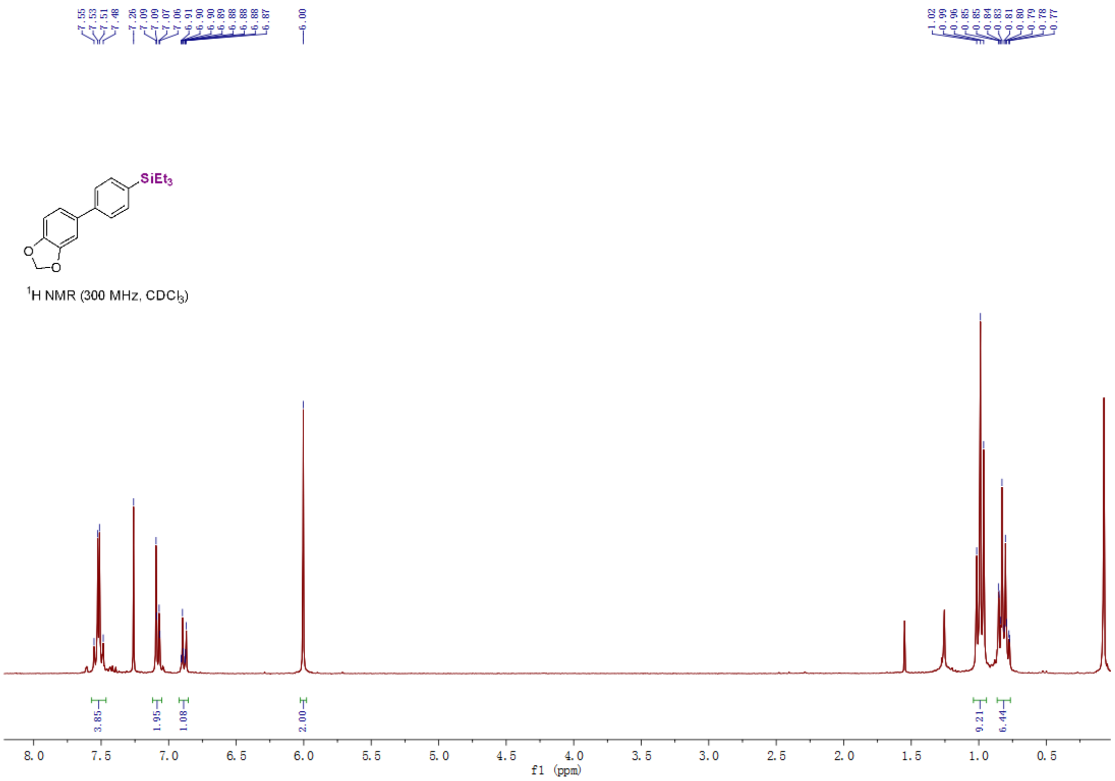


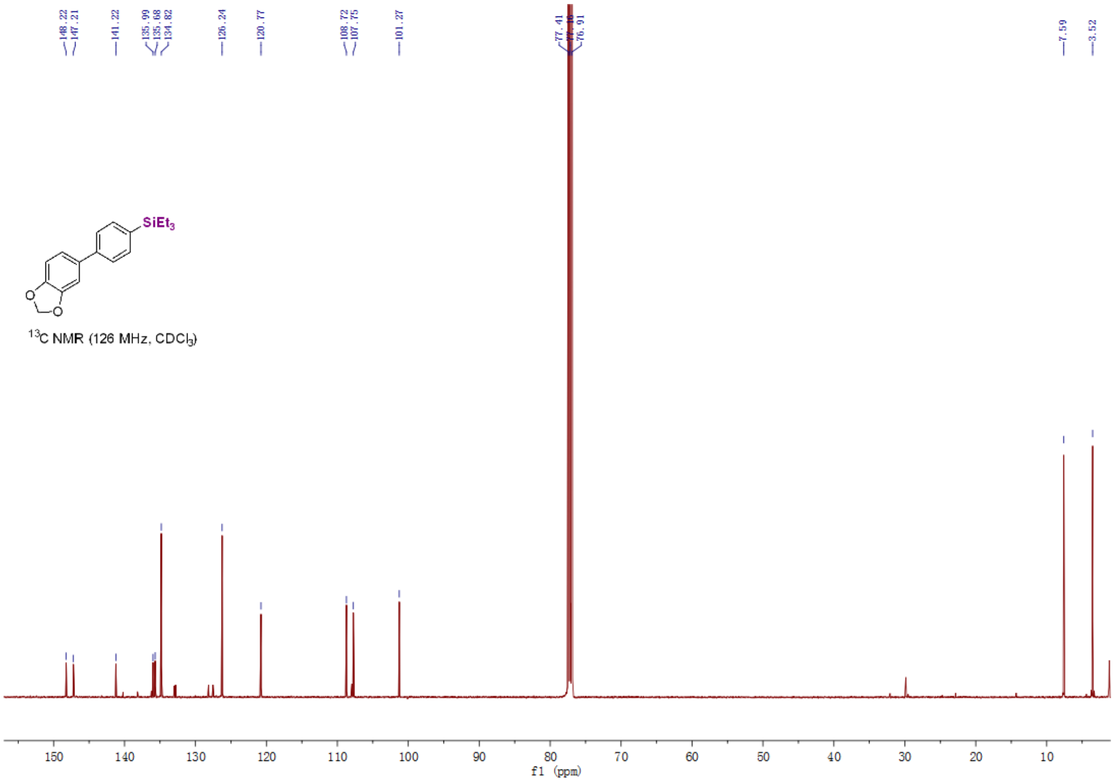


**Supplementary Figure 51**. NMR Spectra of (4-(benzo[*d*][1,3]dioxol-5-yl)phenyl)triethylsilane.

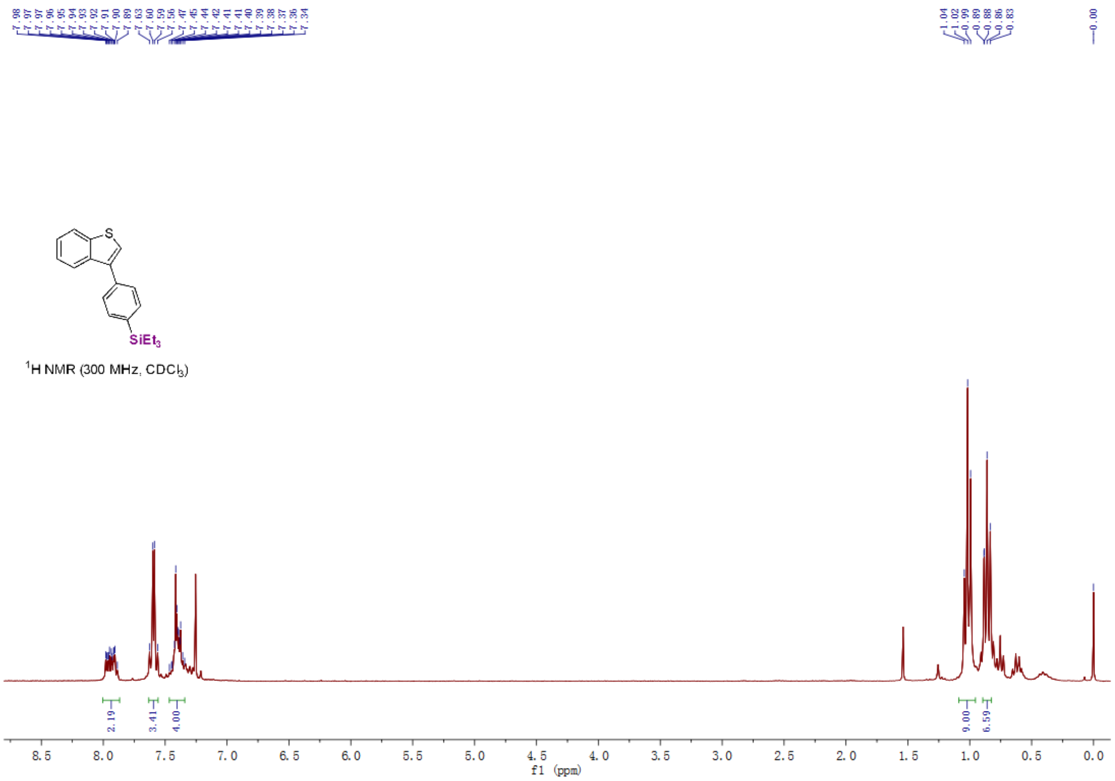


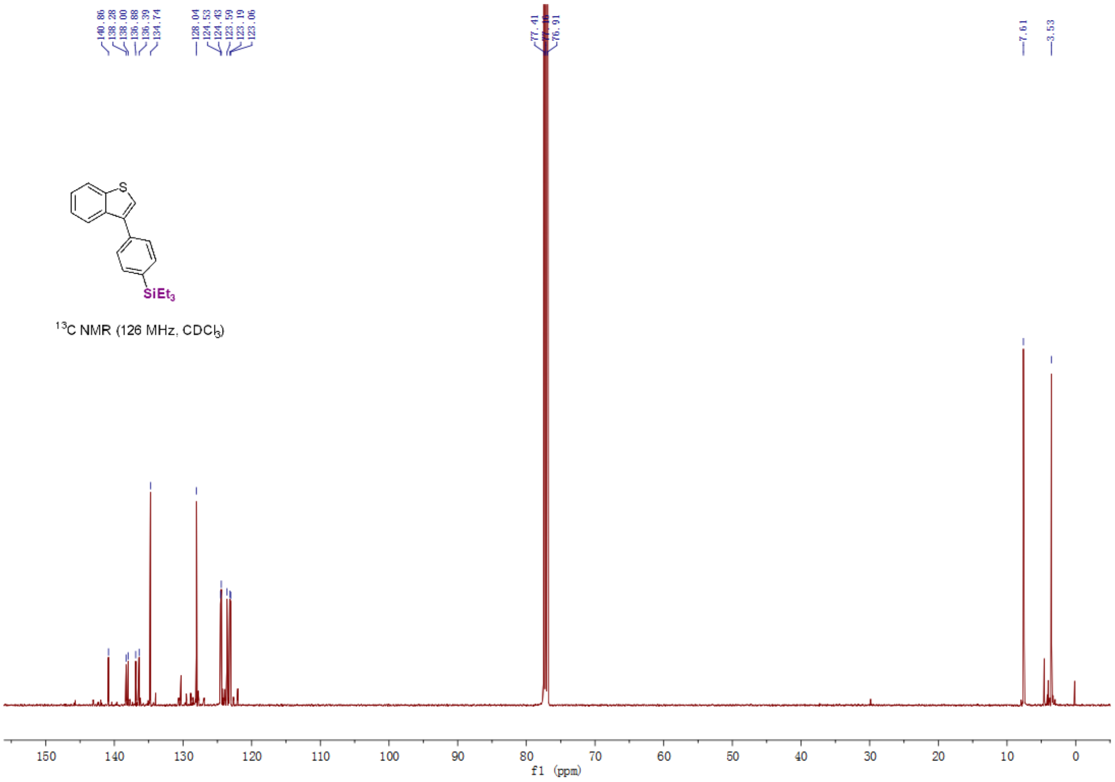


**Supplementary Figure 52**. NMR Spectra of (4-(benzo[b]thiophen-3-yl)phenyl)triethylsilane.

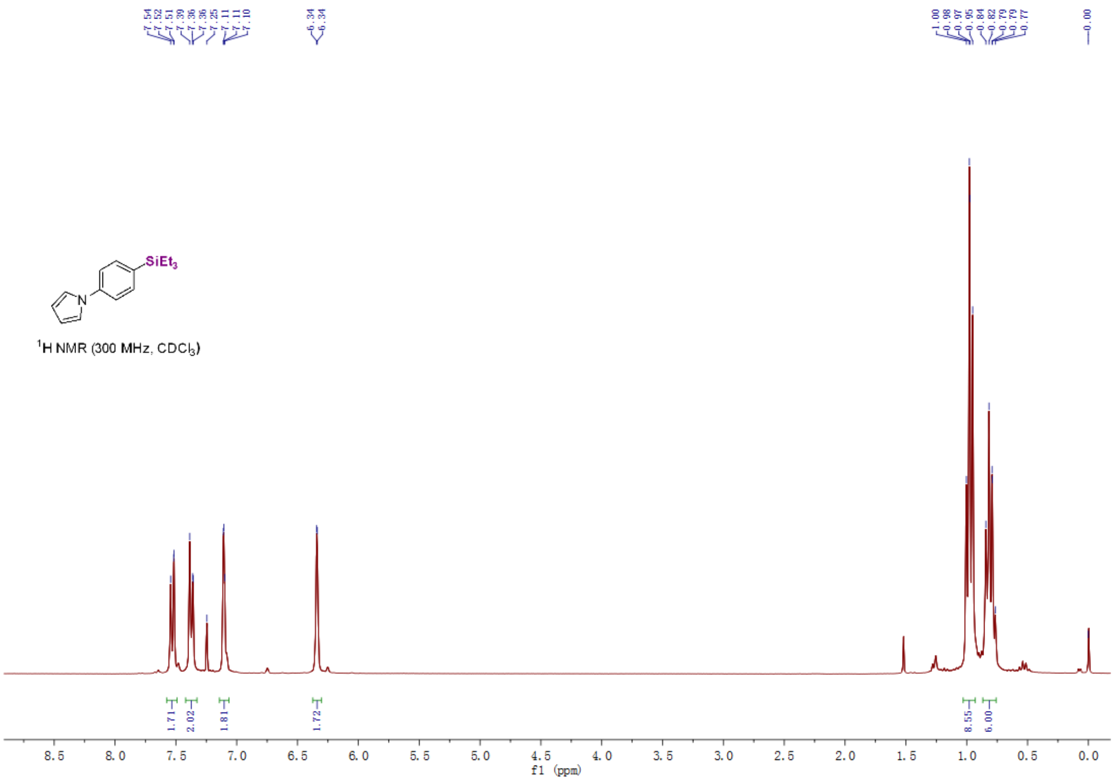


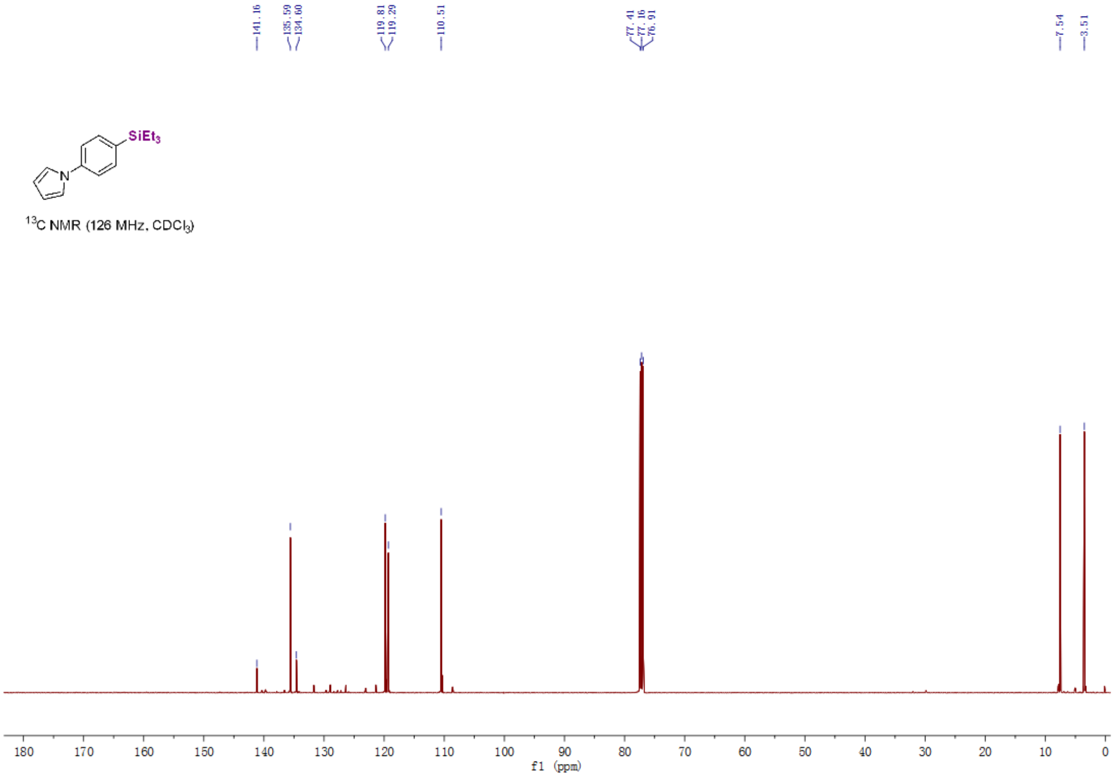


**Supplementary Figure 53**. NMR Spectra of *N*-(4-(triethylsilyl)phenyl)pyrrole.

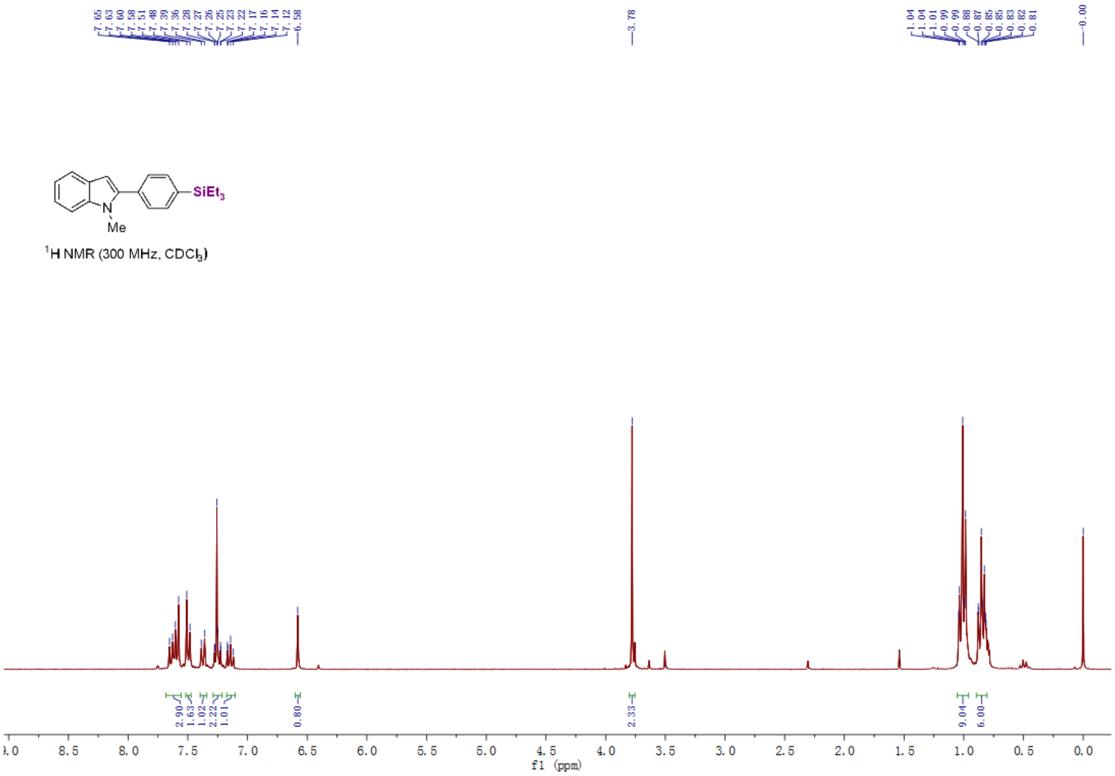


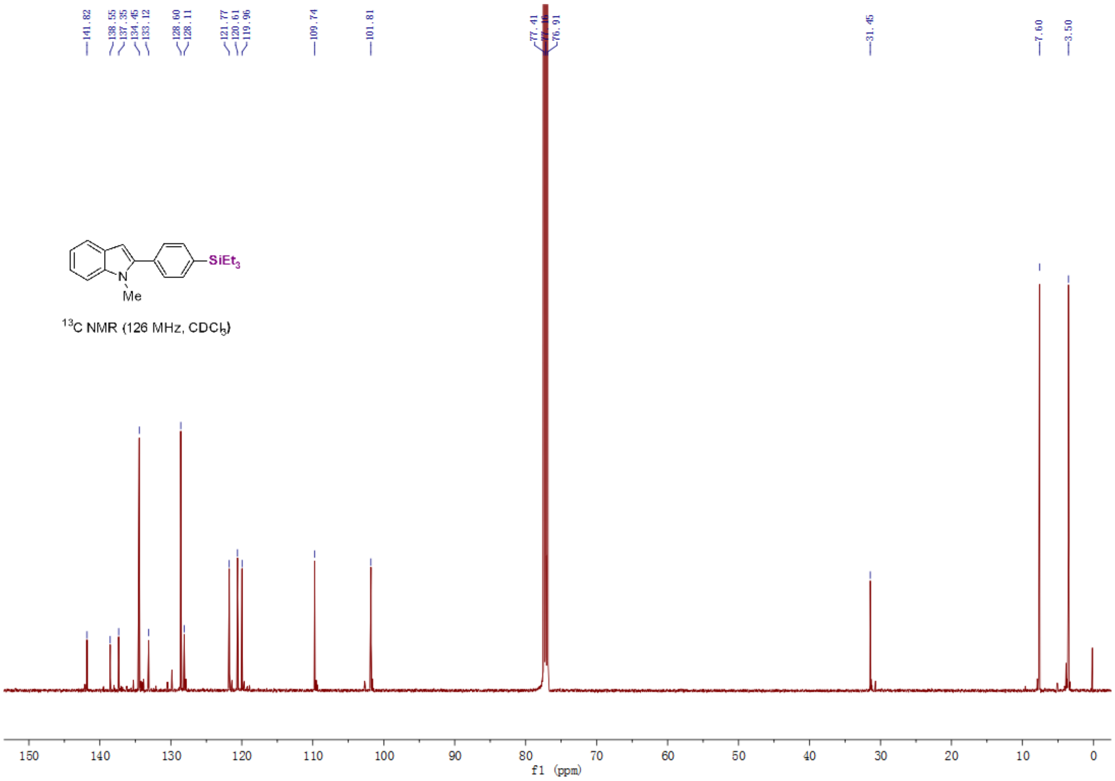


**Supplementary Figure 54**. NMR Spectra of 1-methyl-2-(4-(triethylsilyl)phenyl)-1*H*-indole.

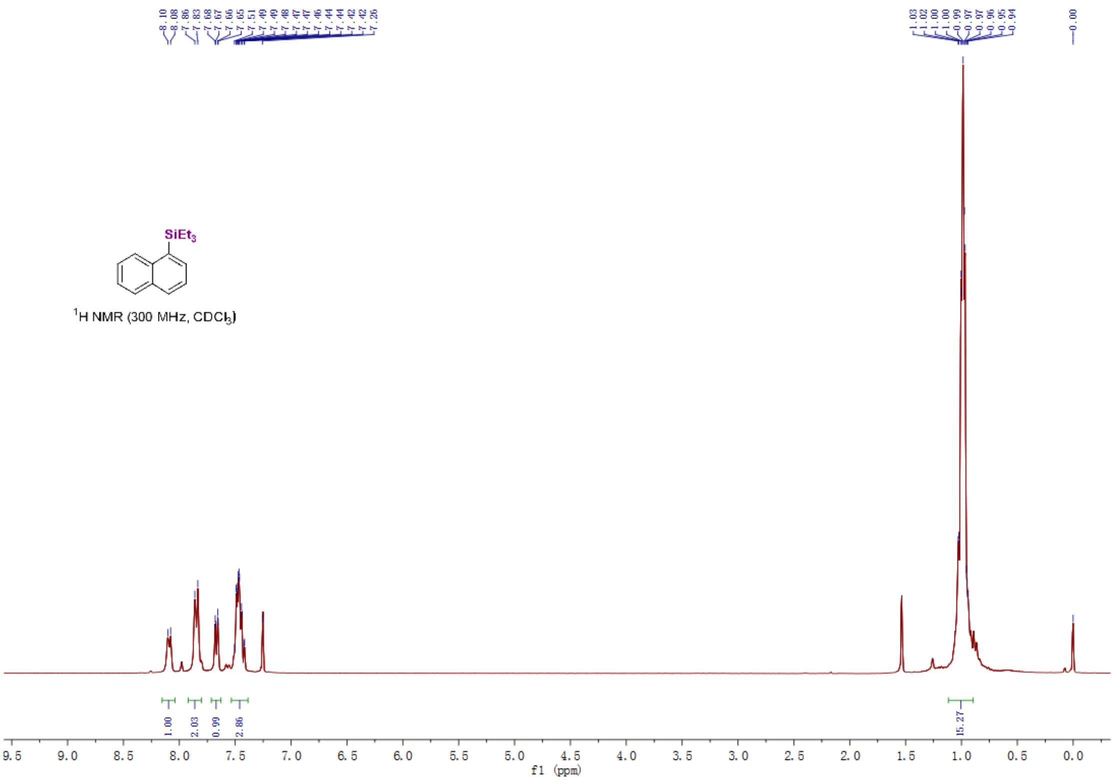


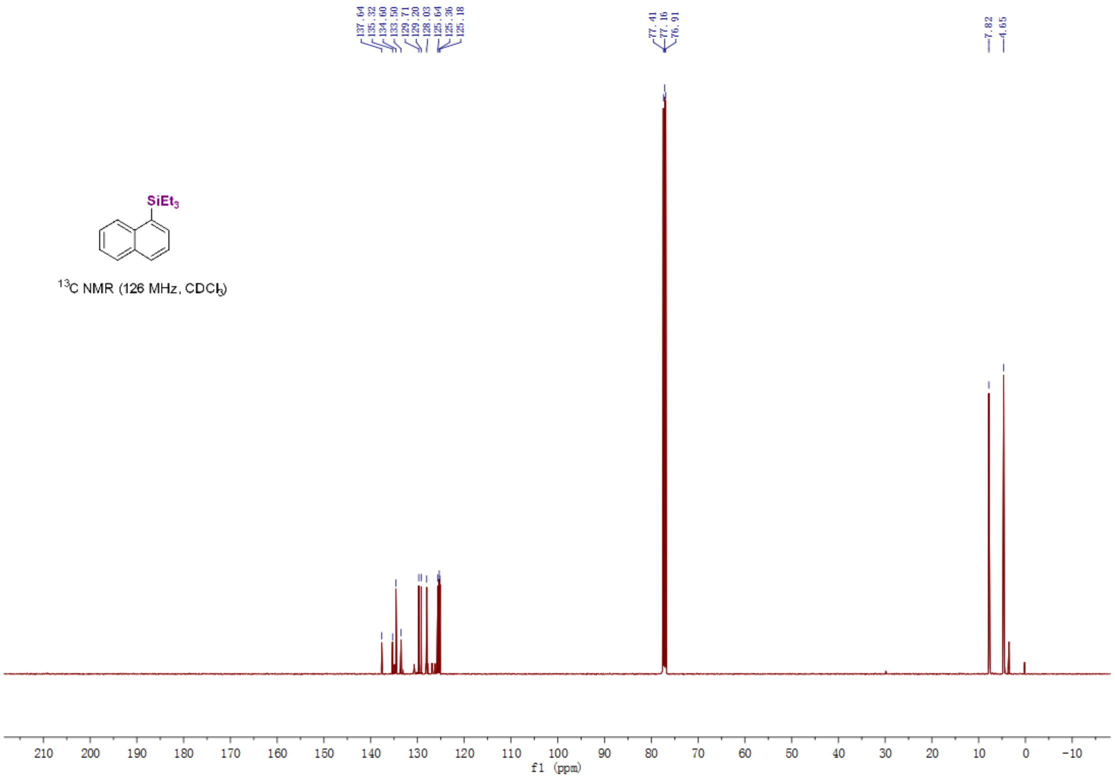


**Supplementary Figure 55**. NMR Spectra of triethyl(naphthalene-1yl)silane.

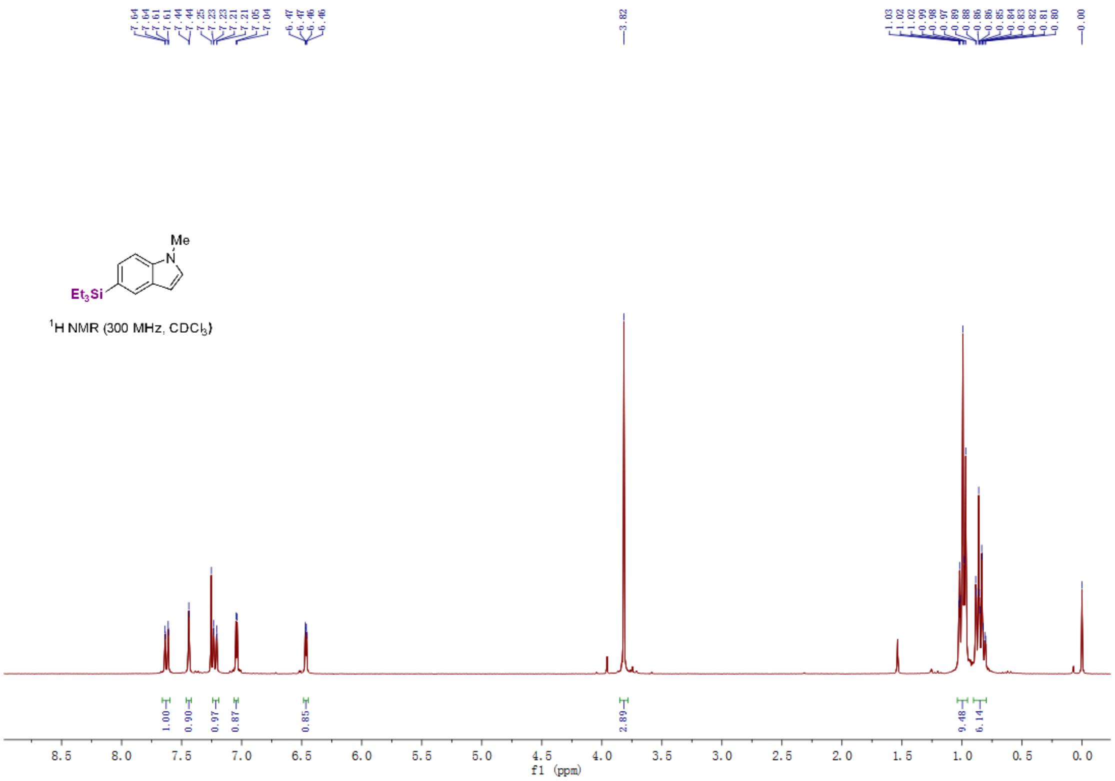


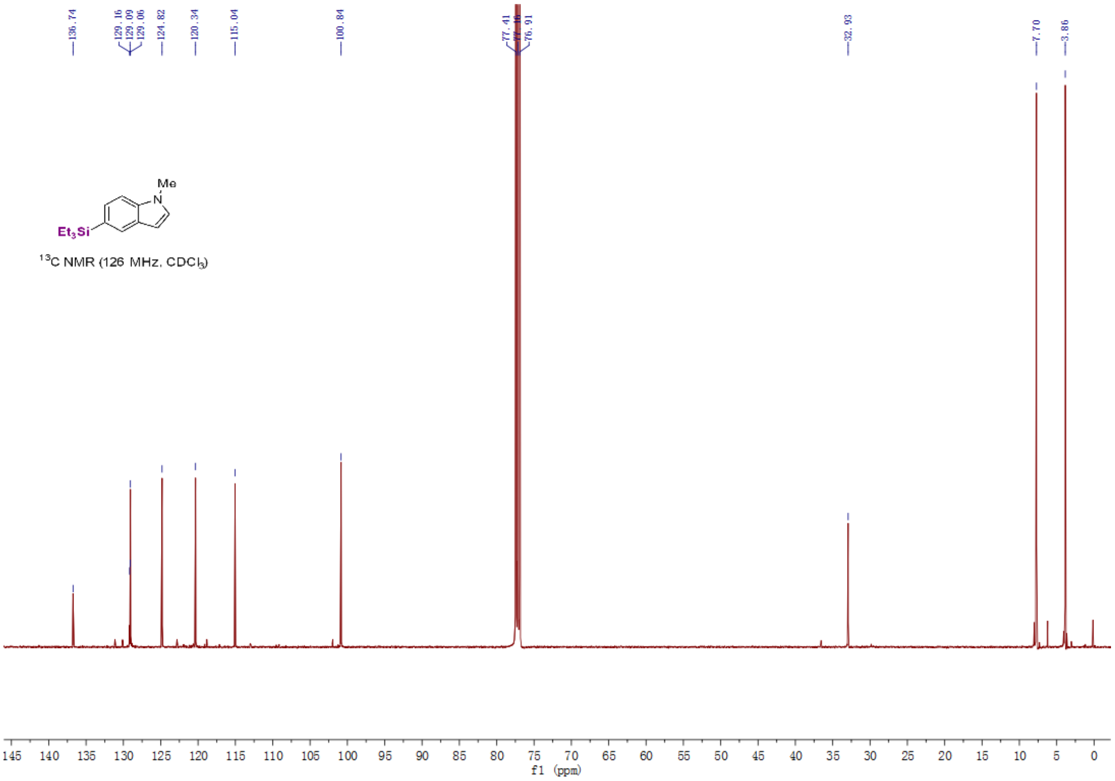


**Supplementary Figure 56**. NMR Spectra of 1-methyl-5-(triethylsilyl)-1*H*-indole.

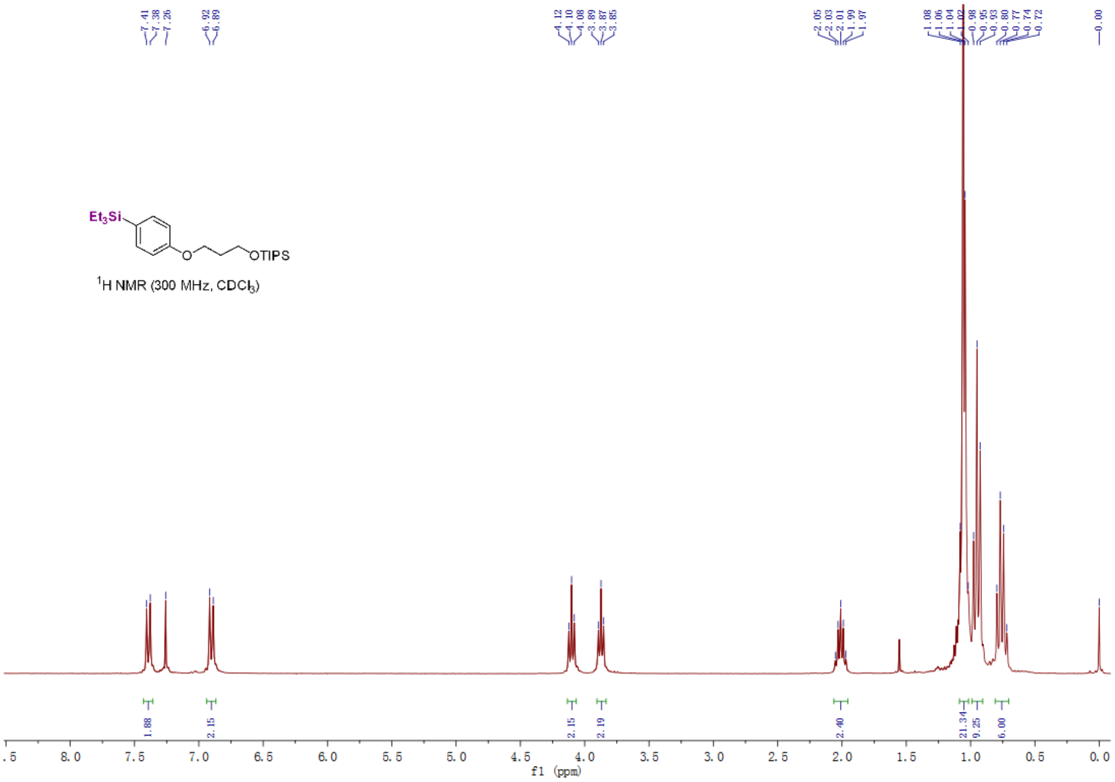


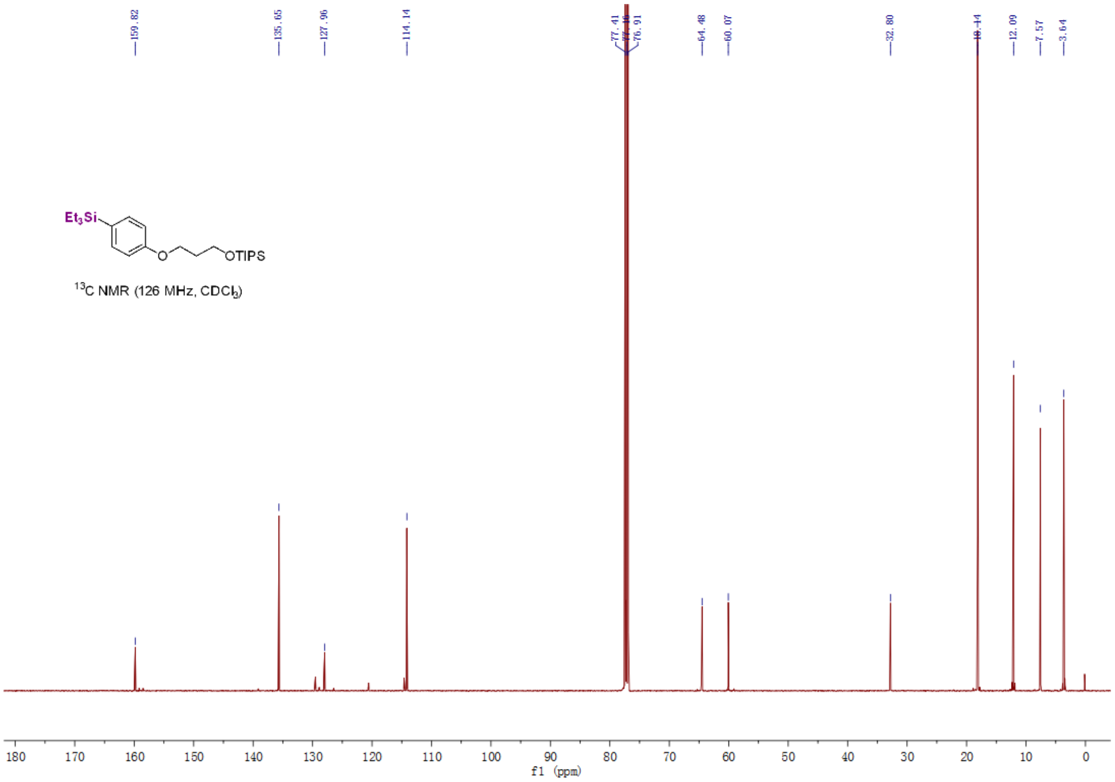


**Supplementary Figure 57**. NMR Spectra of triethyl(4-(3-((triisopropylsilyl)oxy)propoxy)phenyl)silane.

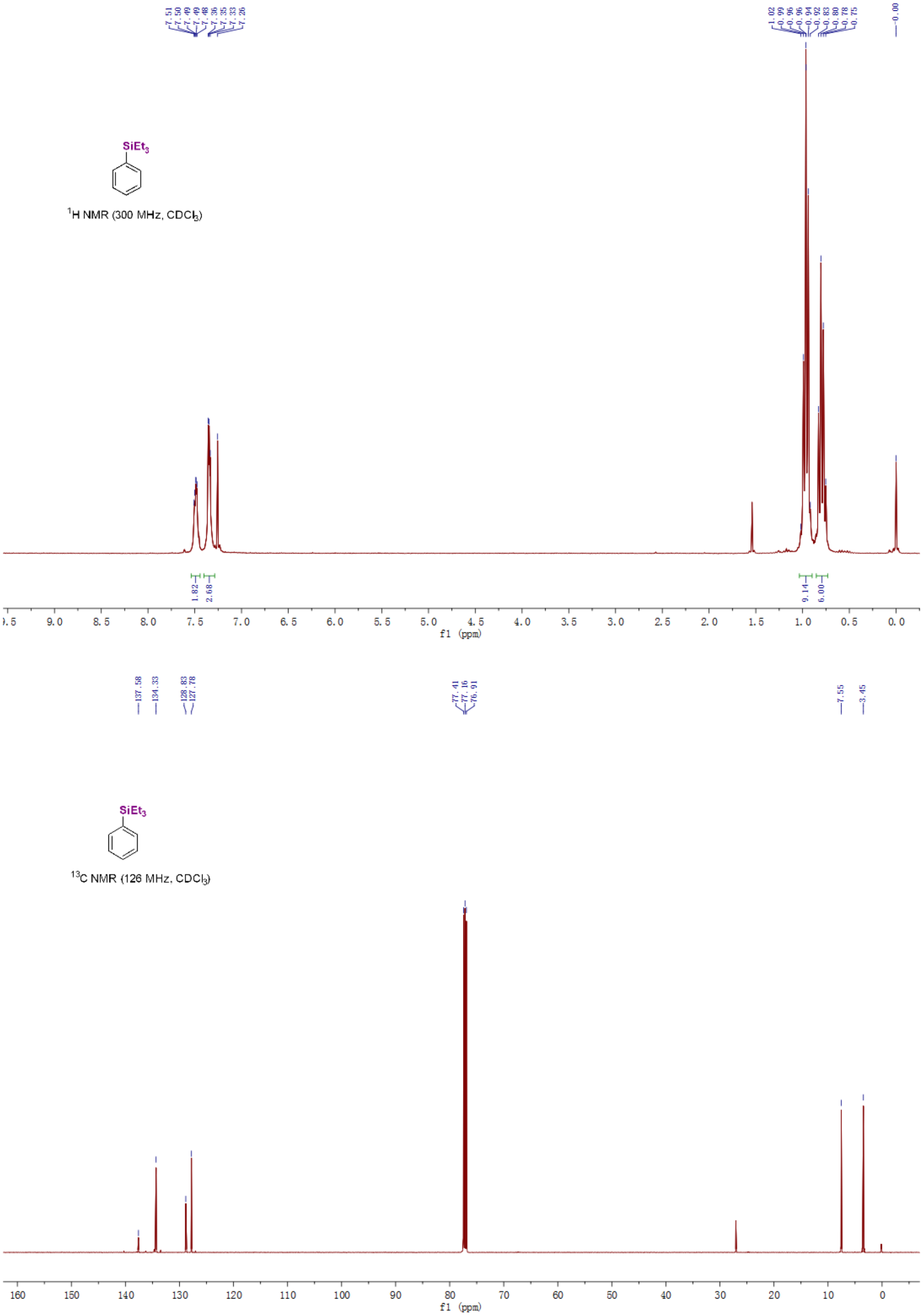


**Supplementary Figure 58**. NMR Spectra of triethyl(phenyl)silane.

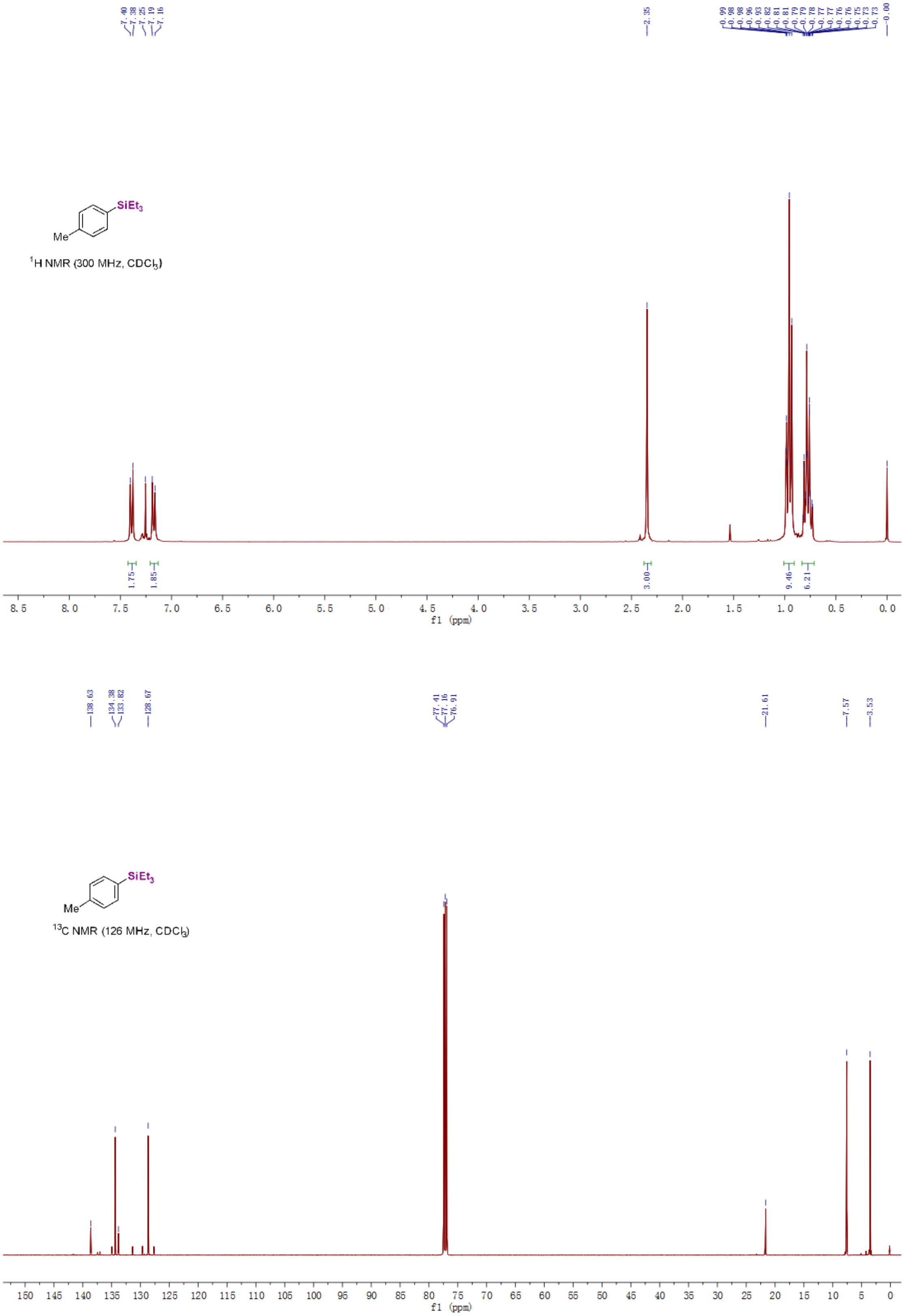


**Supplementary Figure 59**. NMR Spectra of triethyl(*p*-tolyl)silane.

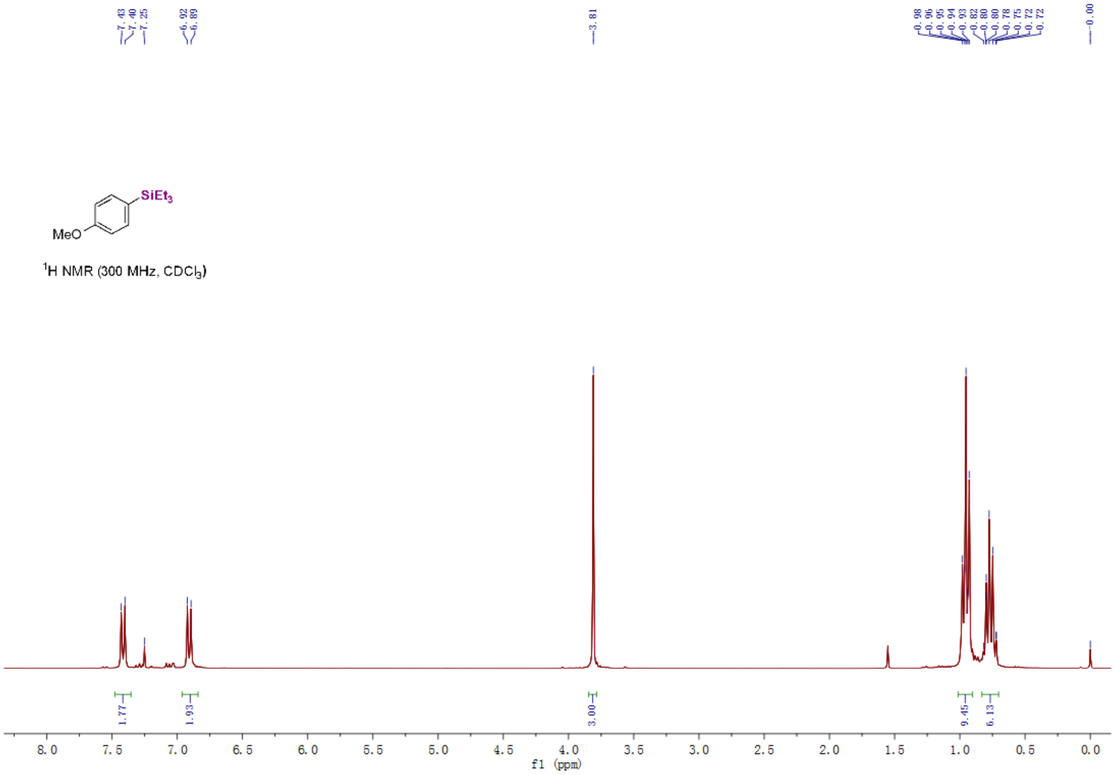


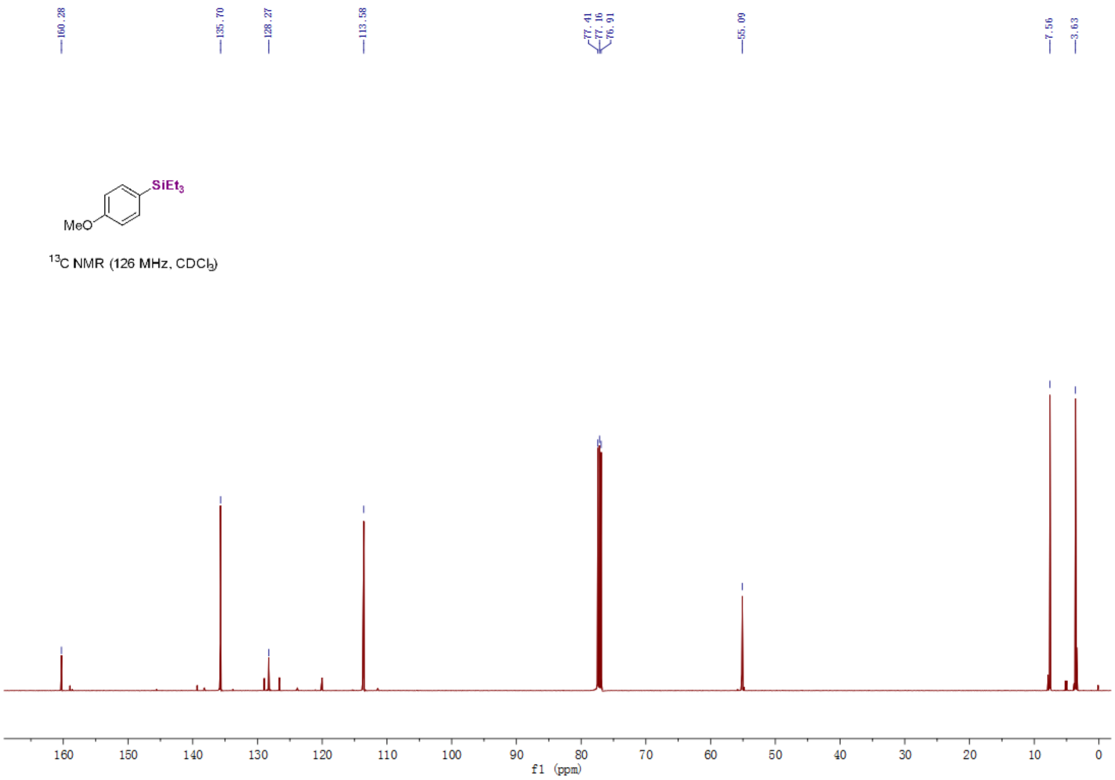


**Supplementary Figure 60**. NMR Spectra of (4-methoxy)phenyltriethylsilane.

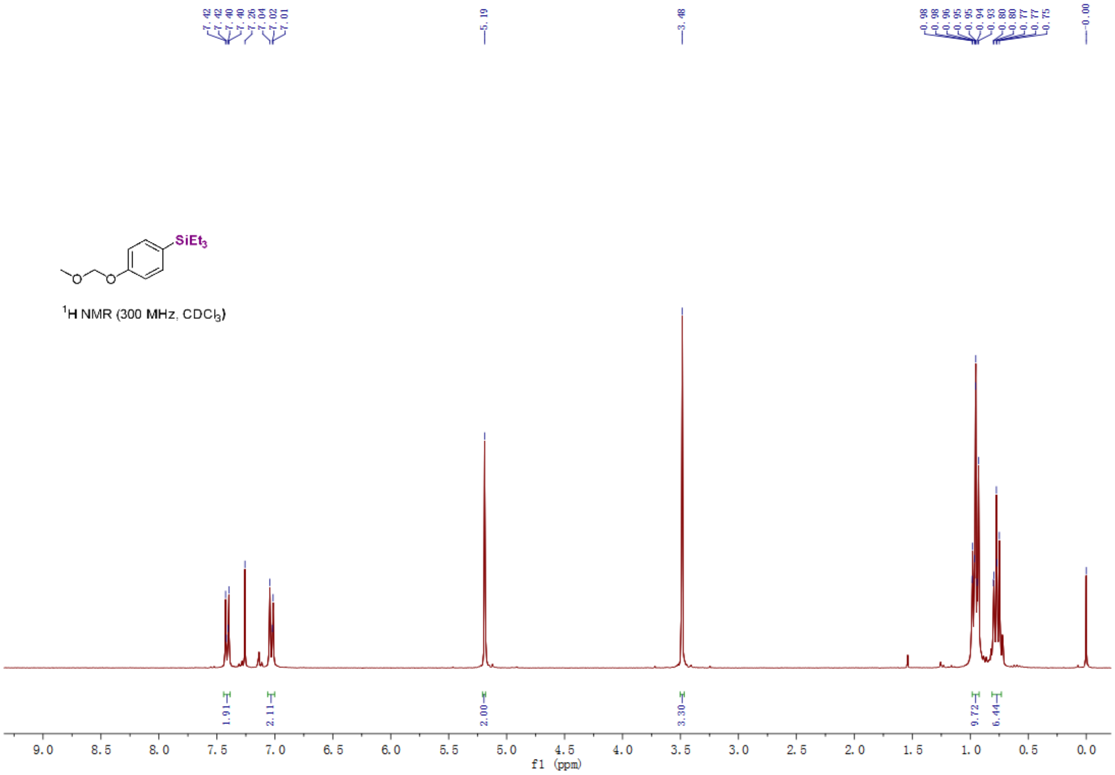


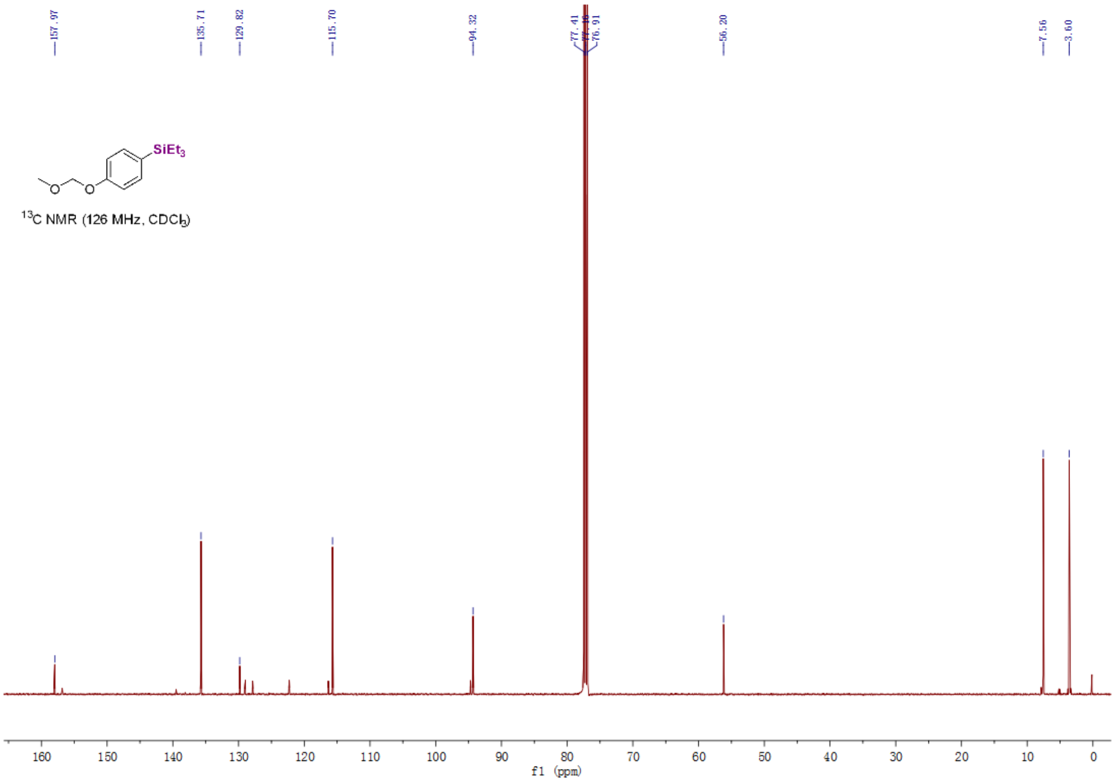


**Supplementary Figure 61**. NMR Spectra of triethyl(4-(methoxymethoxy)-phenyl)silane.

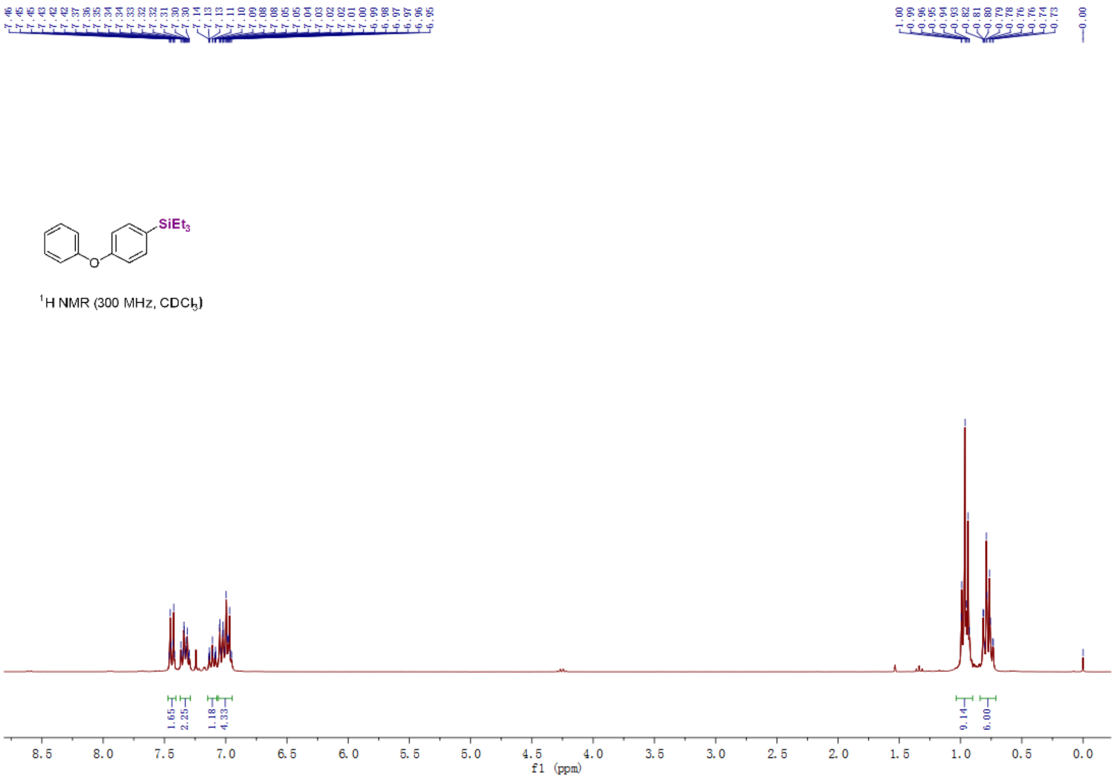


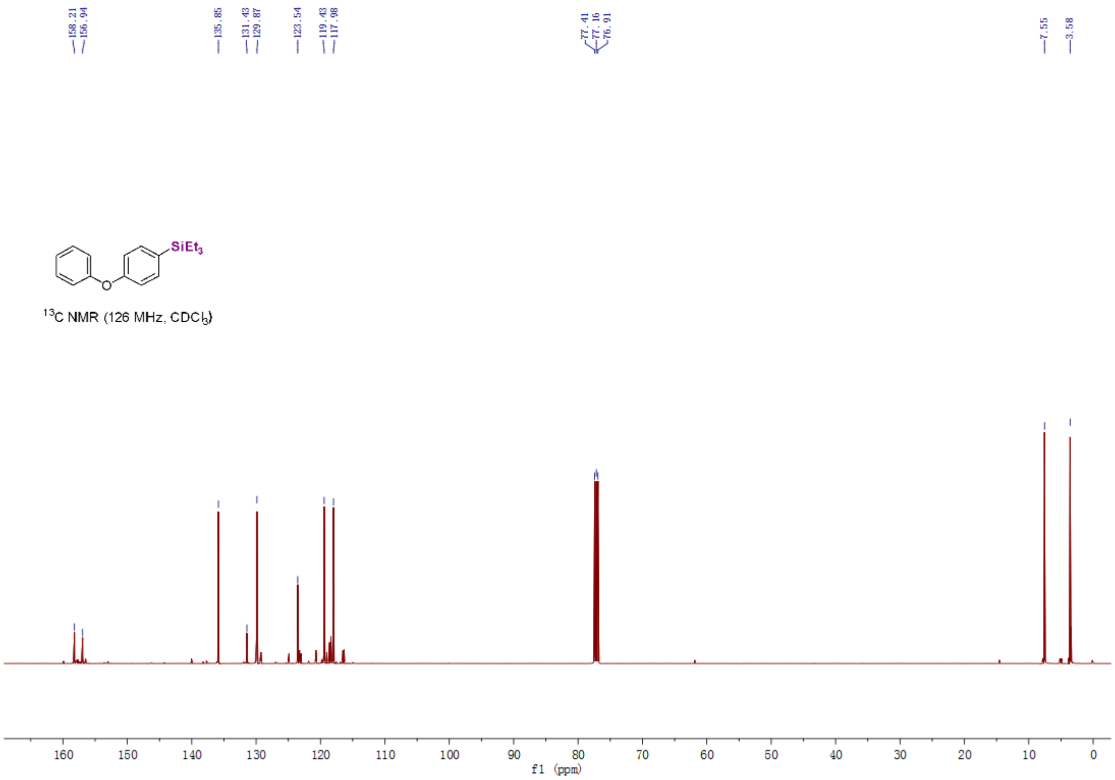


**Supplementary Figure 62**. NMR Spectra of triethyl(4-phenoxyphenyl)silane.

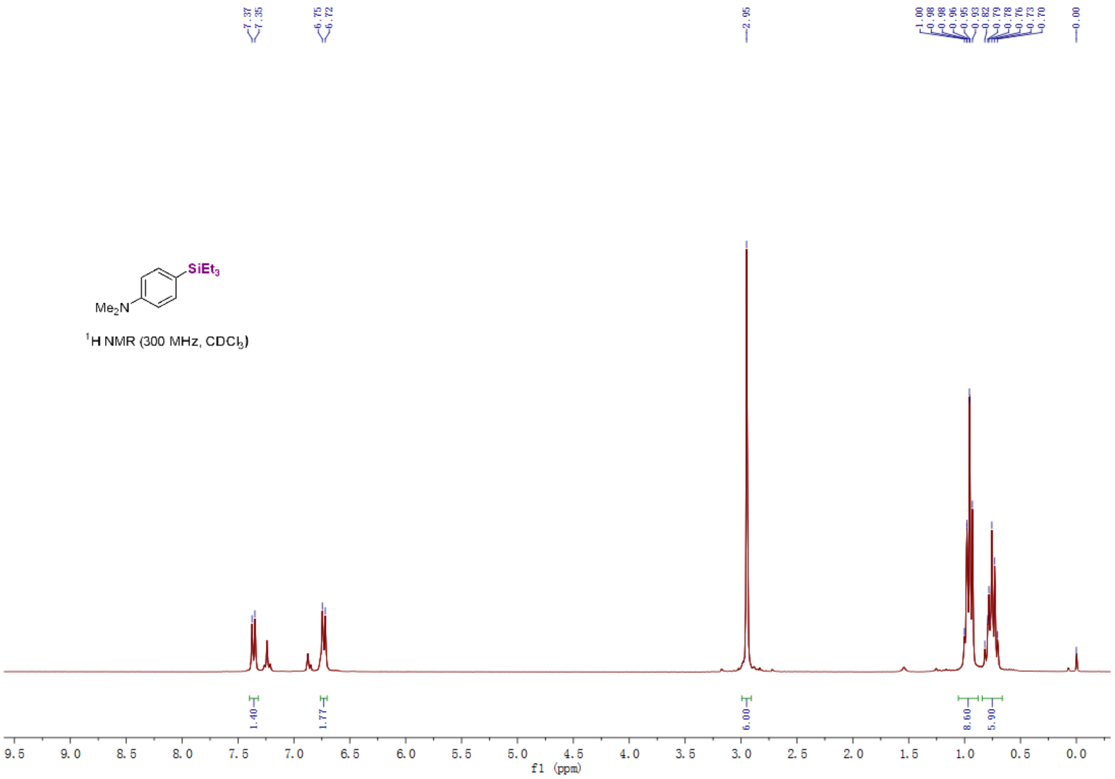


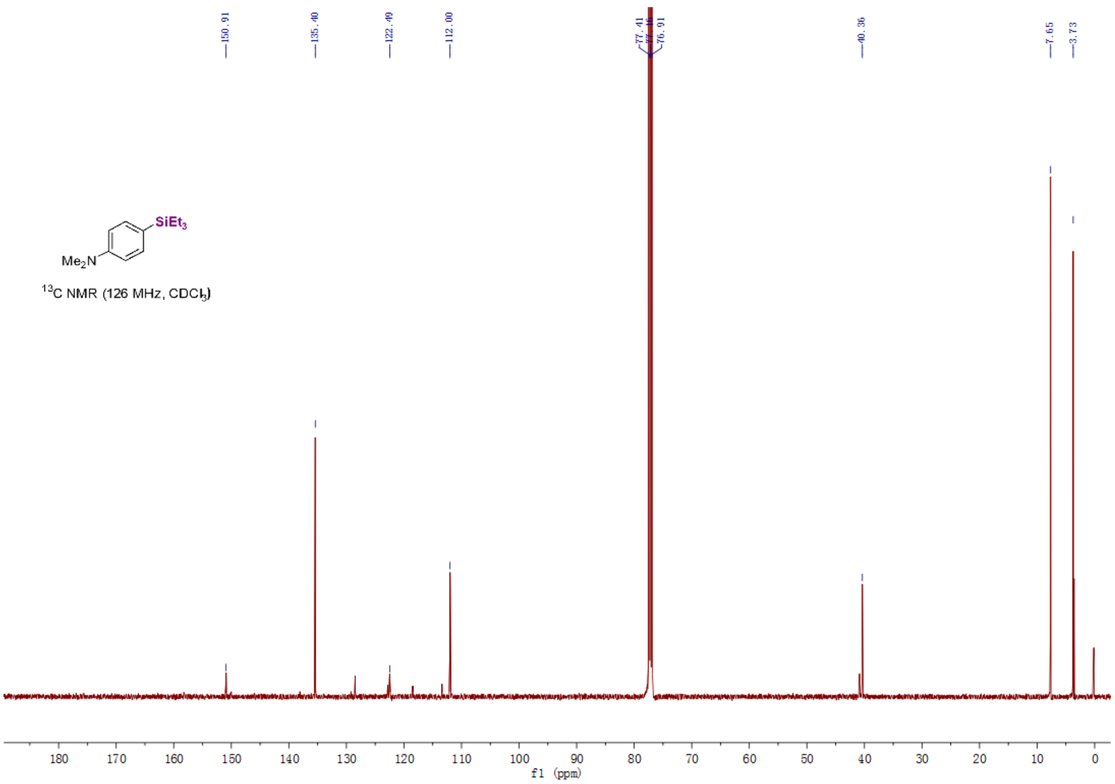


**Supplementary Figure 63**. NMR Spectra of *N*,*N*-dimethyl-4-(triethylsilyl)aniline.

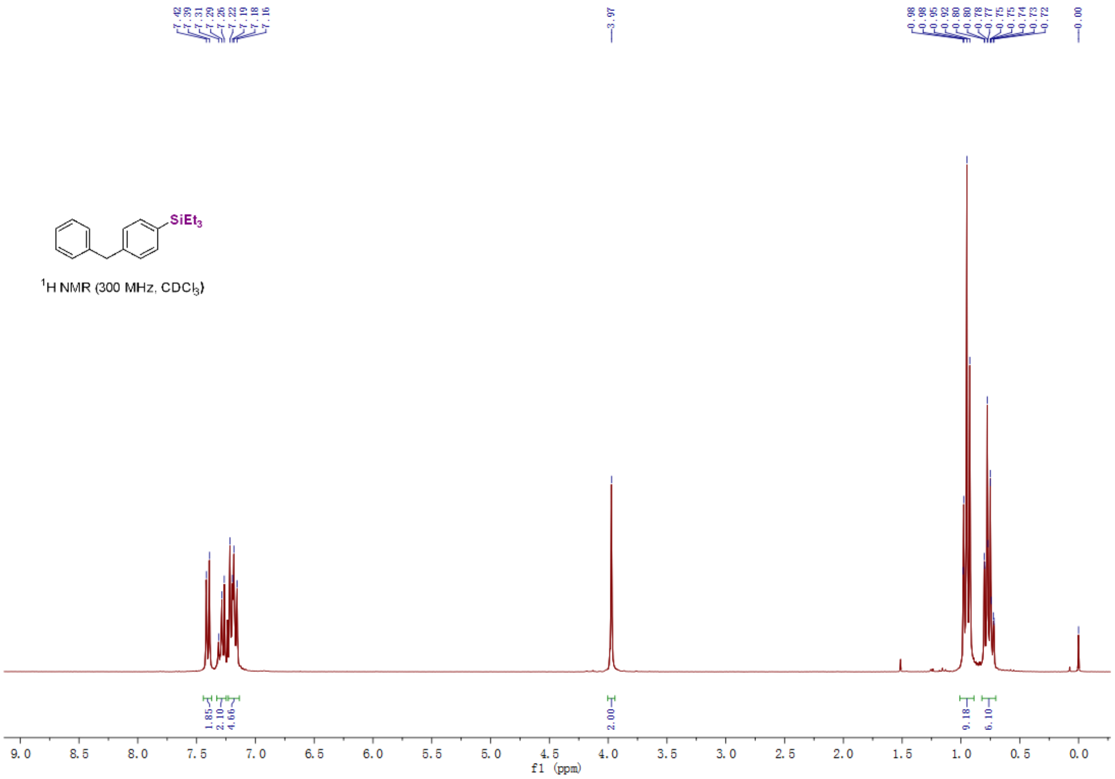


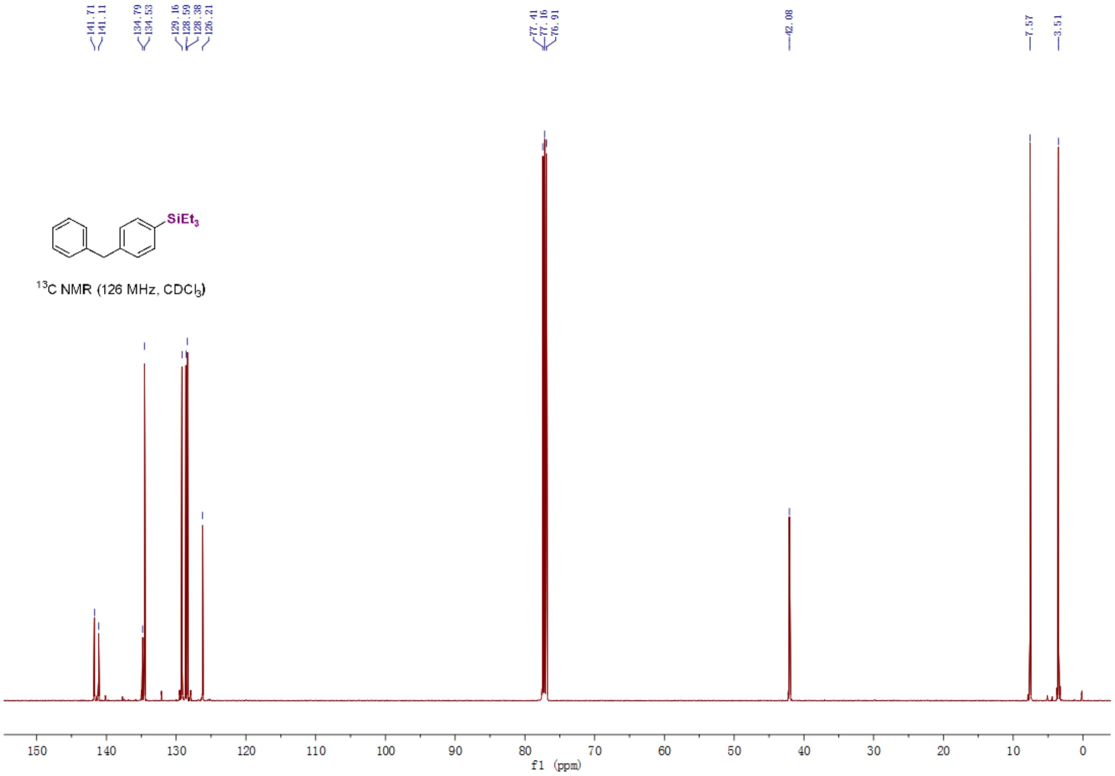


**Supplementary Figure 64**. NMR Spectra of (4-benzylphenyl)triethylsilane.

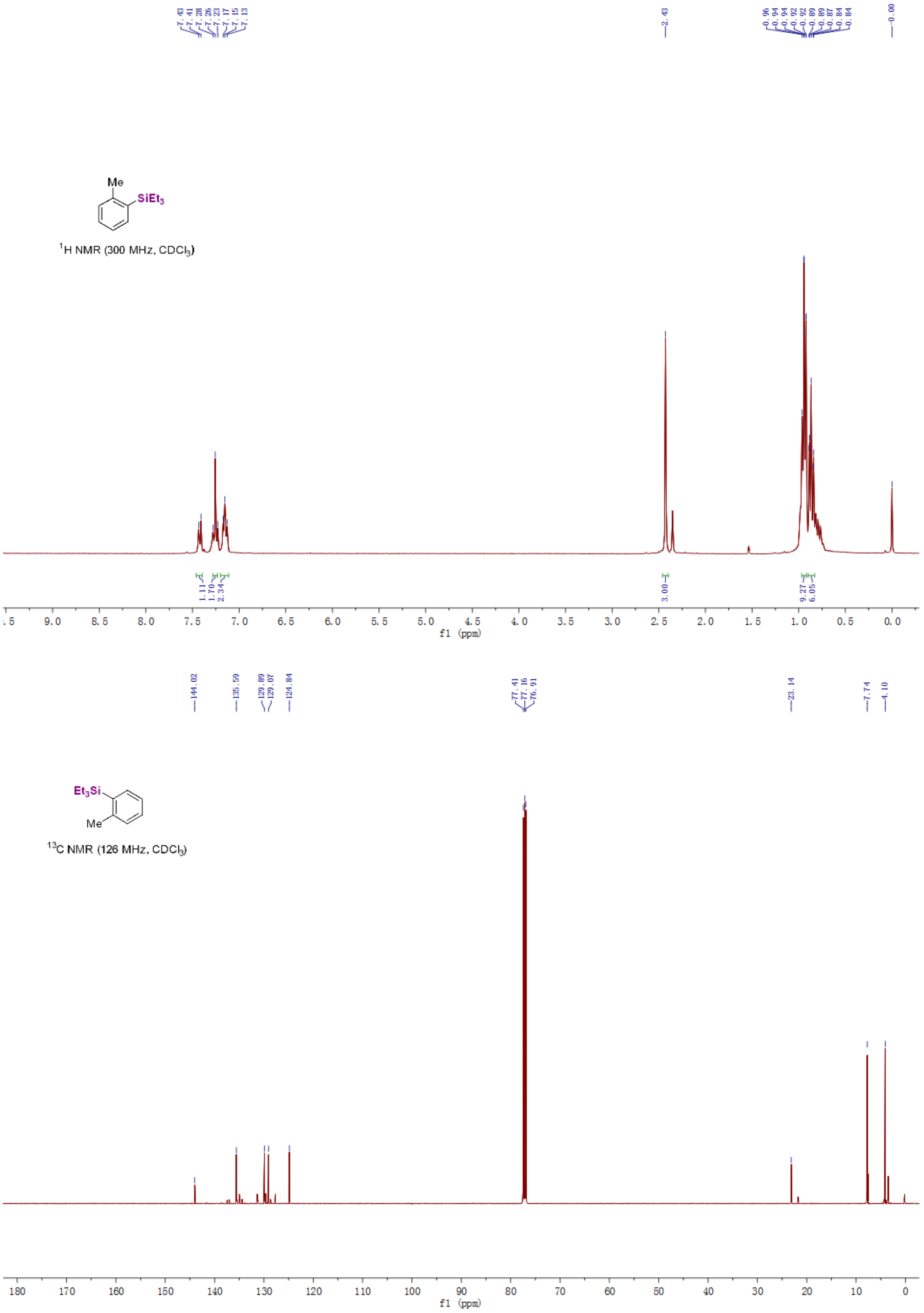


**Supplementary Figure 65**. NMR Spectra of (2-methylphenyl)triethylsilane.

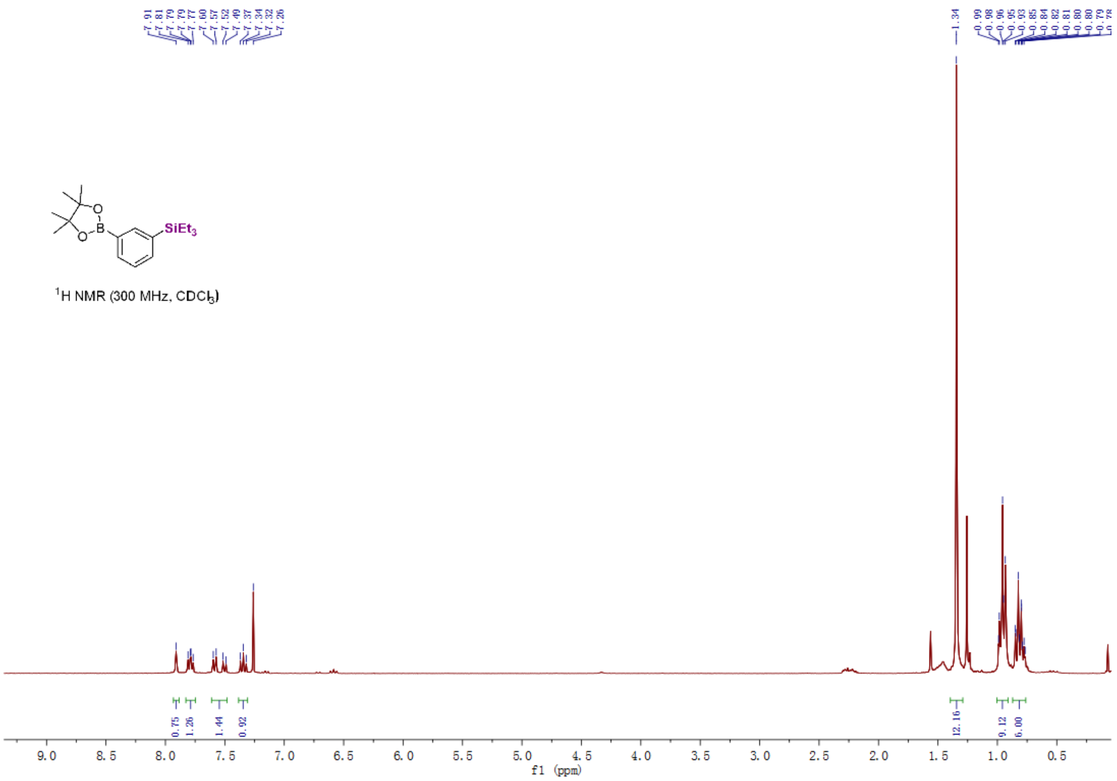


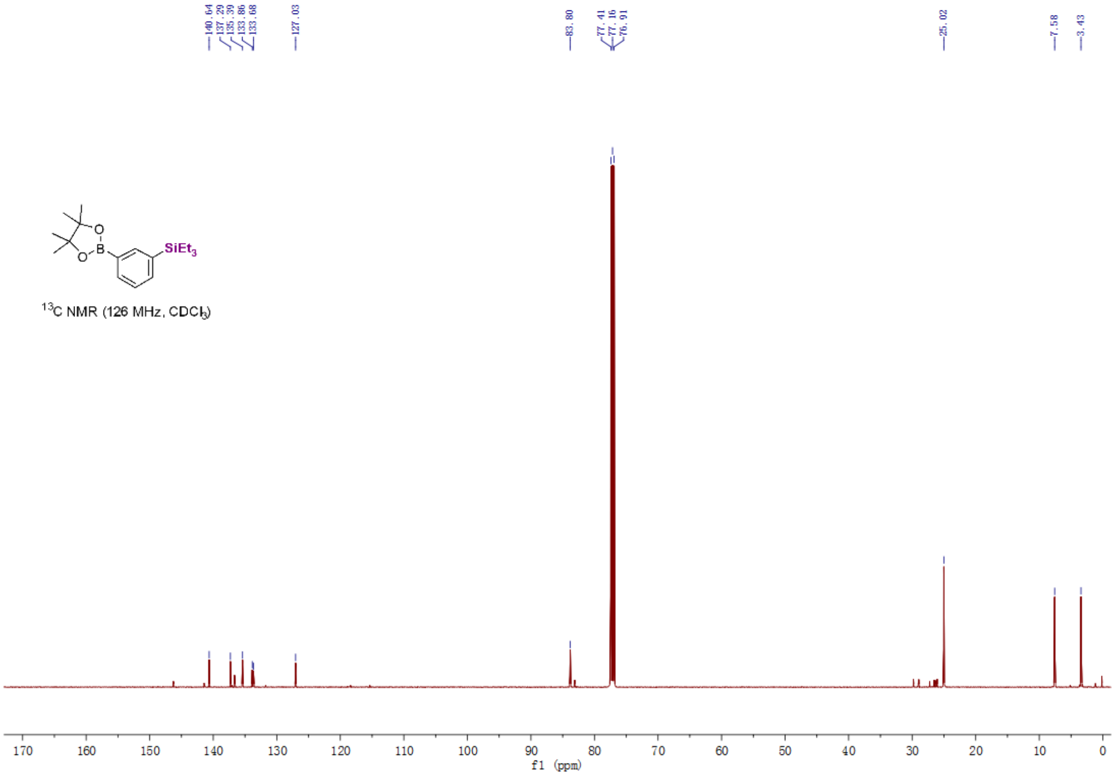


**Supplementary Figure 66**. NMR Spectra of triethyl(3-(4,4,5,5-tetramethyl-1,3,2-dioxaborolan-2-yl)phenyl)silane.

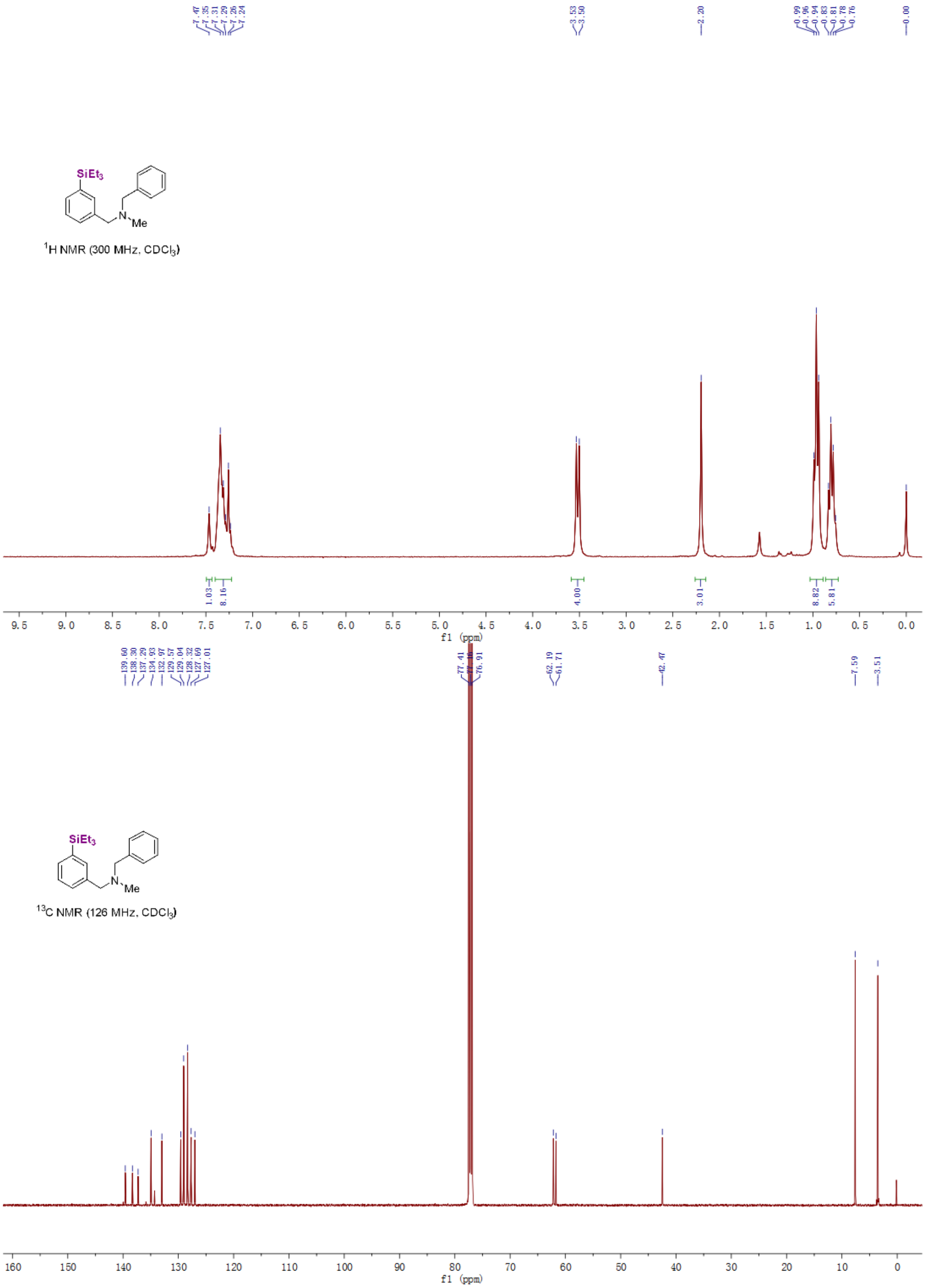


**Supplementary Figure 67**. NMR Spectra of *N*-benzyl-*N*-methyl-1-(3-(triethylsilyl)phenyl)methanamine.

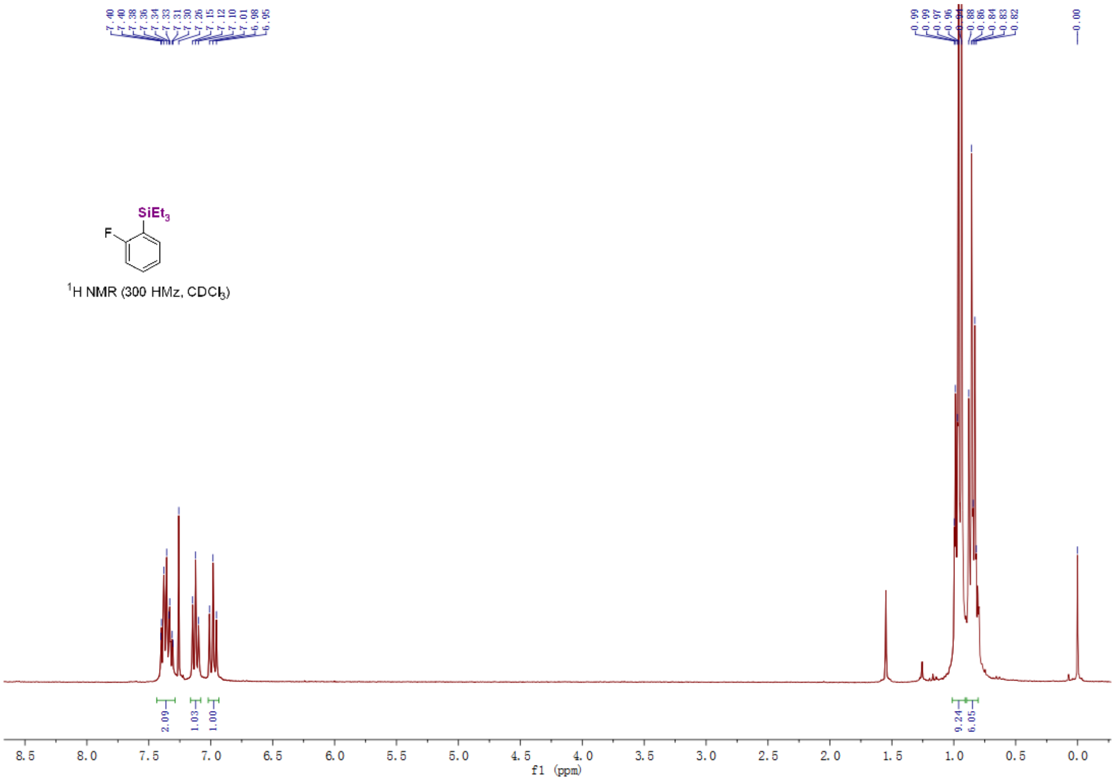
7.40
7.40
7.38
7.36
7.34
7.33
7.31
7.30
7.26
7.15
7.12
7.10
7.01
6.98
6.95
0.99
0.99
0.97
0.96
0.88
0.86
0.84
0.83
0.82
0.00
SiEt3
F
1H NMR (300 HMz, CDCl3)
2.09
1.03
1.00
9.24
6.05
8.5 8.0 7.5 7.0 6.5 6.0 5.5 5.0 4.5 4.0 3.5 3.0 2.5 2.0 1.5 1.0 0.5 0.0
f1 (ppm)

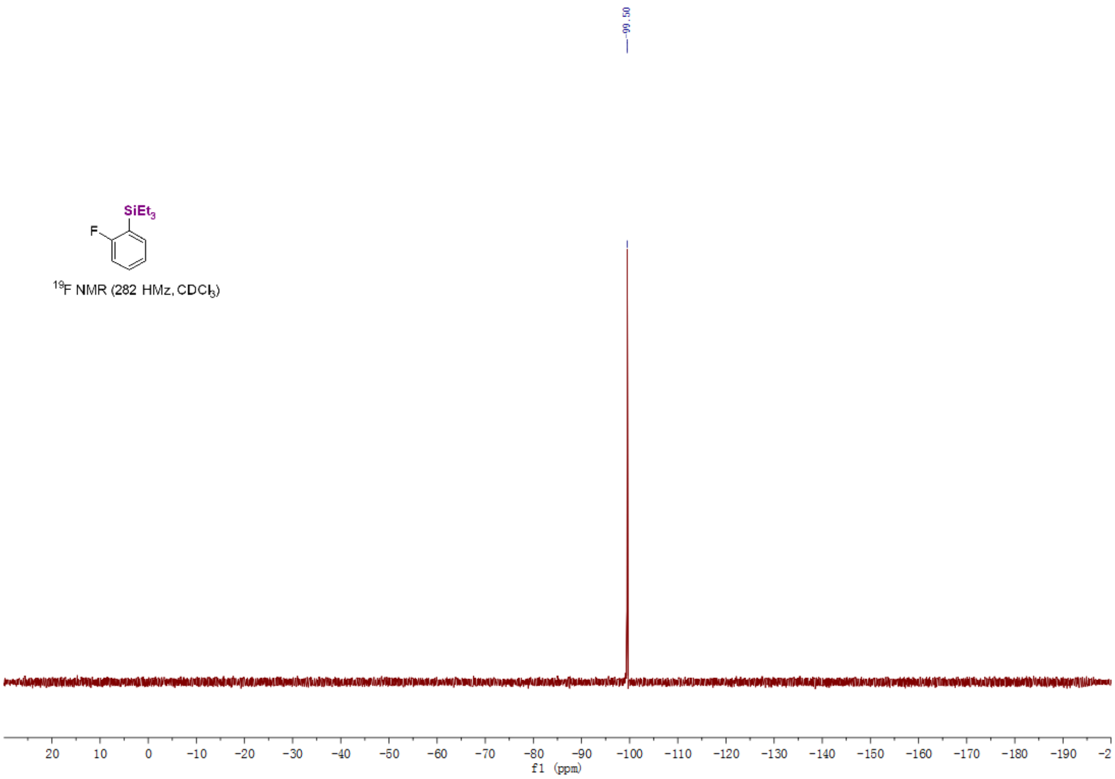
-99.50
SiEt3
F
19F NMR (282 HMz, CDCl3)
20 10 0 -10 -20 -30 -40 -50 -60 -70 -80 -90 -100 -110 -120 -130 -140 -150 -160 -170 -180 -190 -2
f1 (ppm)

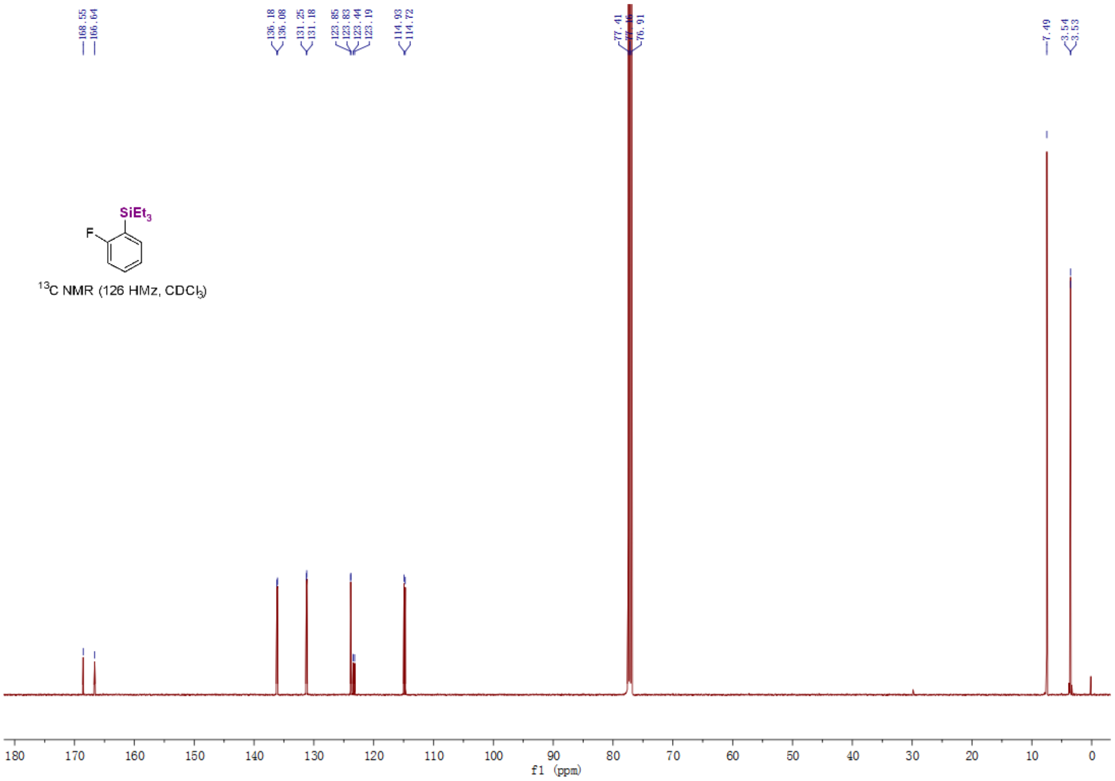


**Supplementary Figure 68**. NMR Spectra of triethyl(2-fluorophenyl)silane.

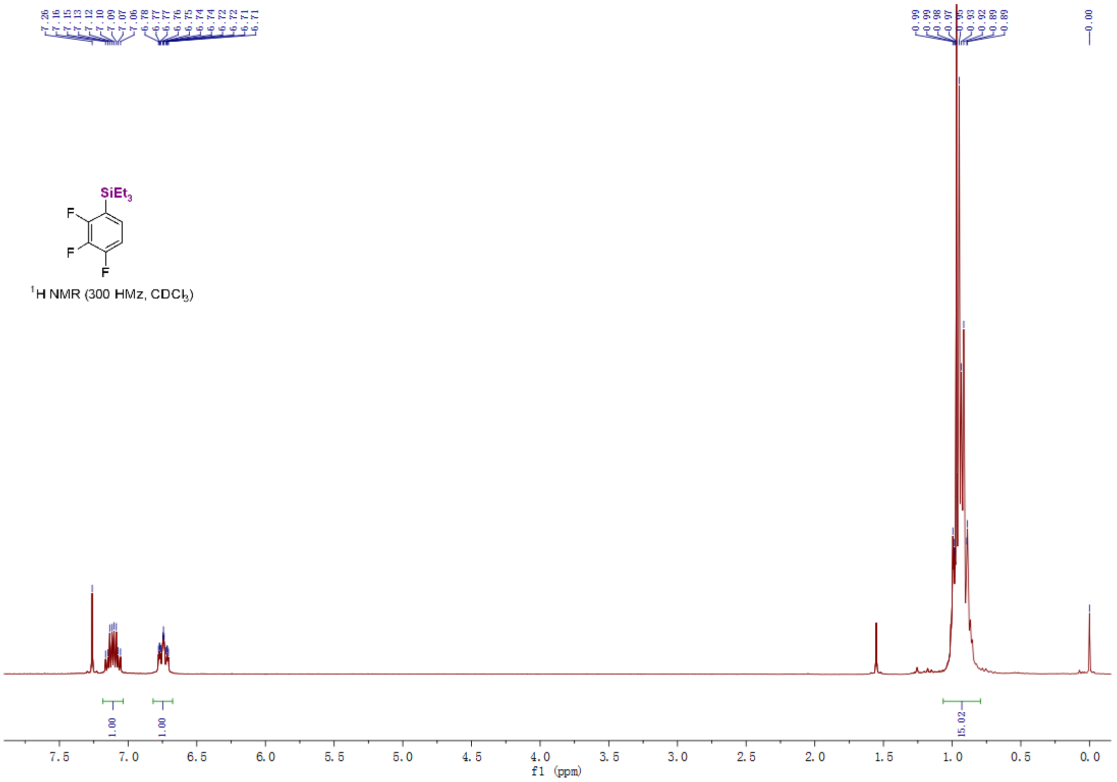
SiEt3
F
F
F
1H NMR (300 HMz, CDCl3)
f1 (ppm)

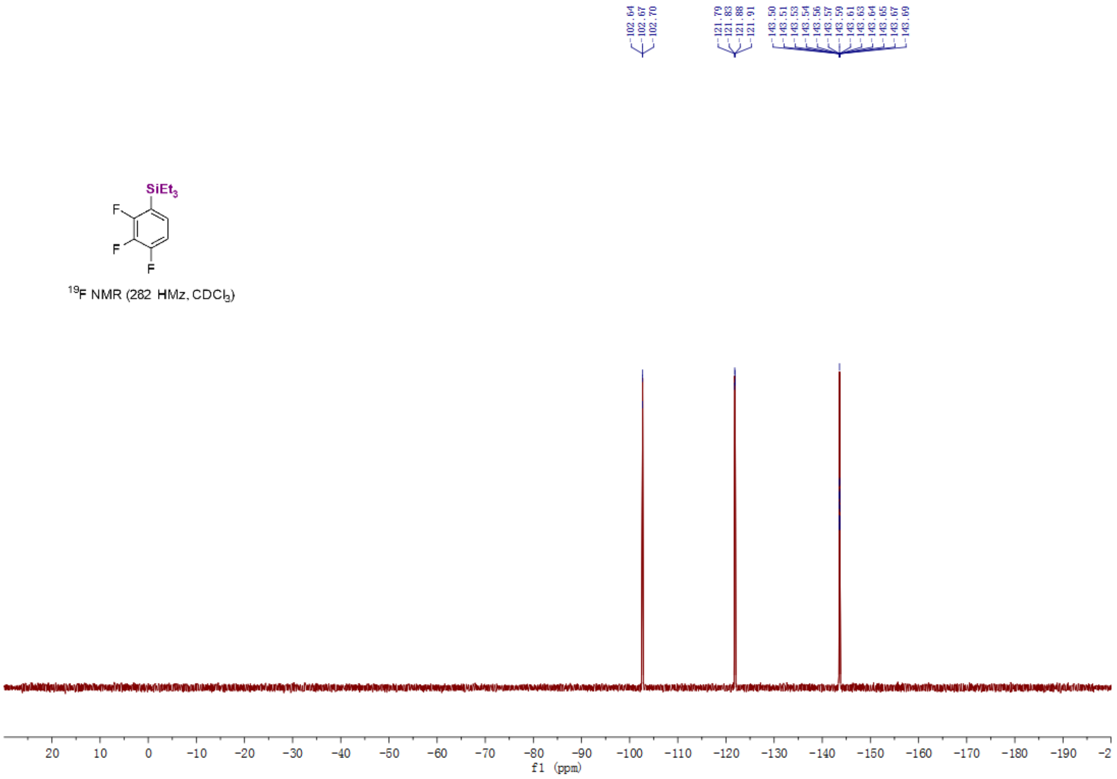
SiEt3
F
F
F
19F NMR (282 HMz, CDCl3)
f1 (ppm)

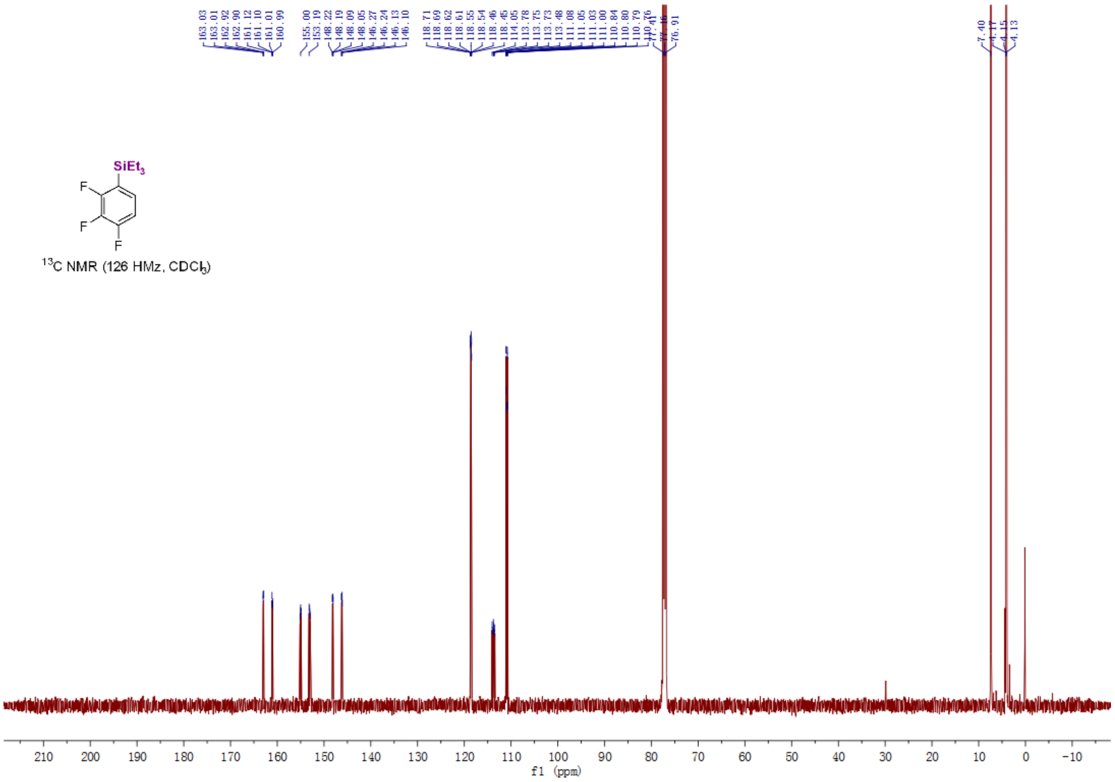


**Supplementary Figure 69**. NMR Spectra of triethyl(2,3,4-trifluorophenyl)silane.

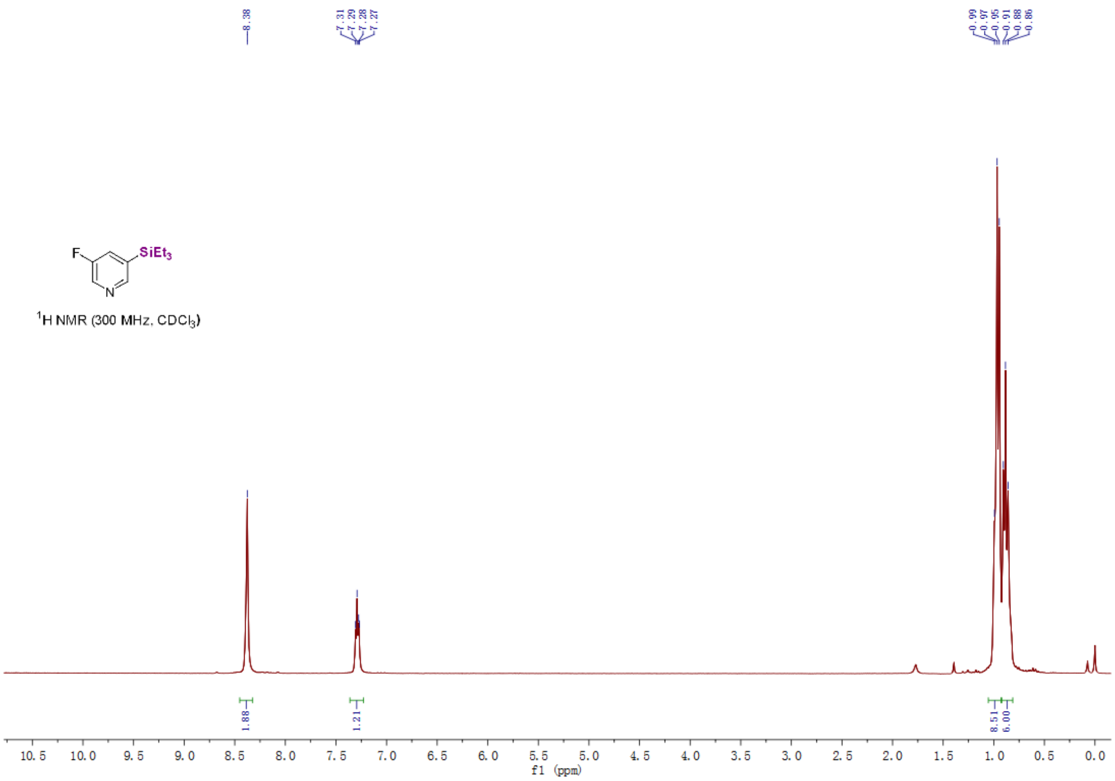
8.38
7.31
7.29
7.28
7.27
0.99
0.97
0.95
0.91
0.88
0.86
F
SiEt3
N
1H NMR (300 MHz, CDCl3)
1.88
1.21
8.51
6.00
10.5 10.0 9.5 9.0 8.5 8.0 7.5 7.0 6.5 6.0 5.5 5.0 4.5 4.0 3.5 3.0 2.5 2.0 1.5 1.0 0.5 0.0
f1 (ppm)

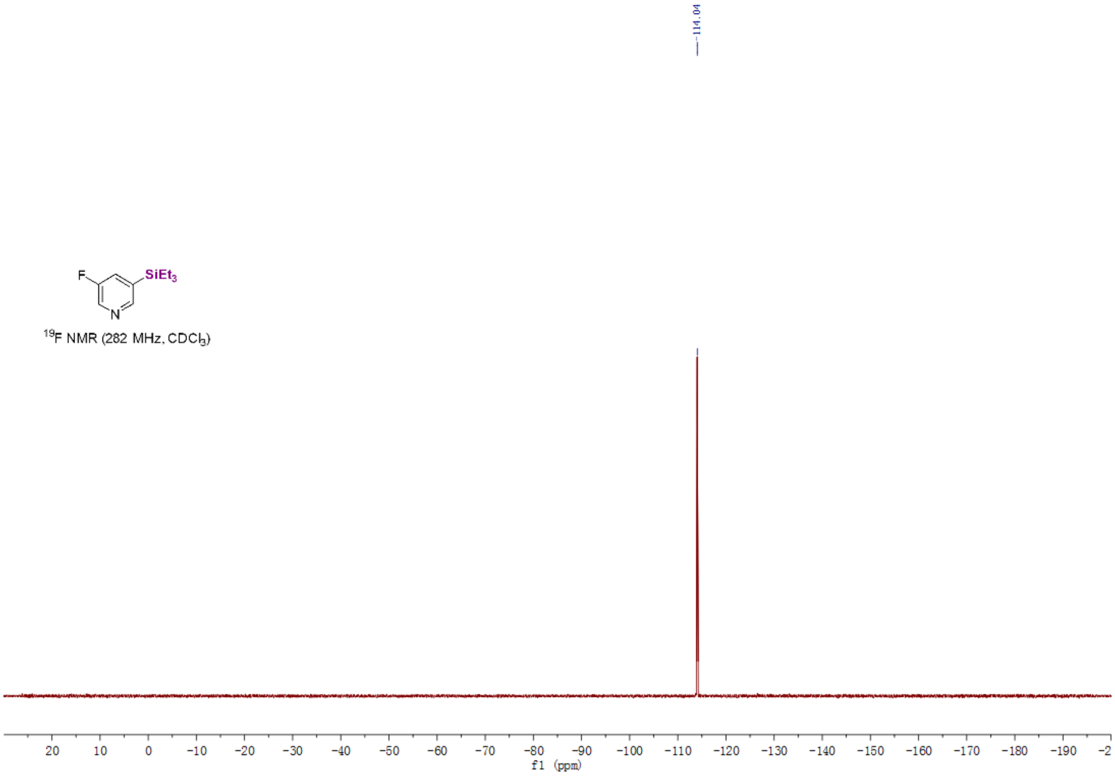
-114.04
F
SiEt3
N
19F NMR (282 MHz, CDCl3)
20 10 0 -10 -20 -30 -40 -50 -60 -70 -80 -90 -100 -110 -120 -130 -140 -150 -160 -170 -180 -190 -2
f1 (ppm)

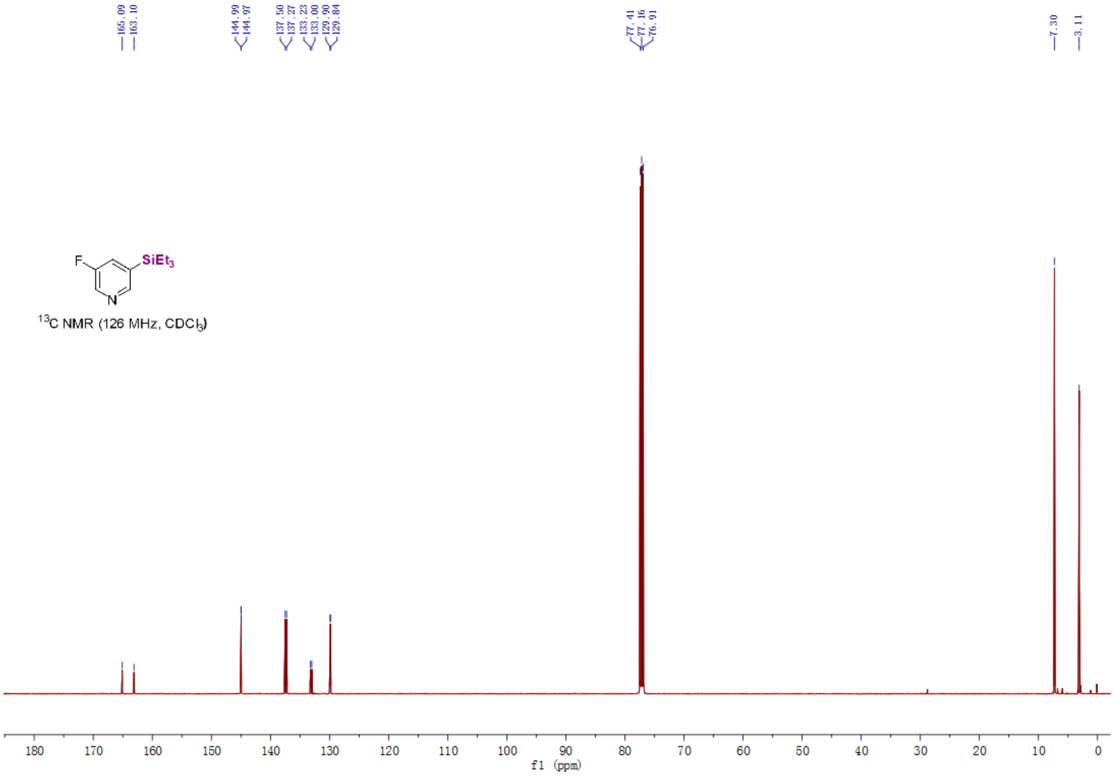


**Supplementary Figure 70**. NMR Spectra of 3-fluoro-5-(triethylsilyl)pyridine.

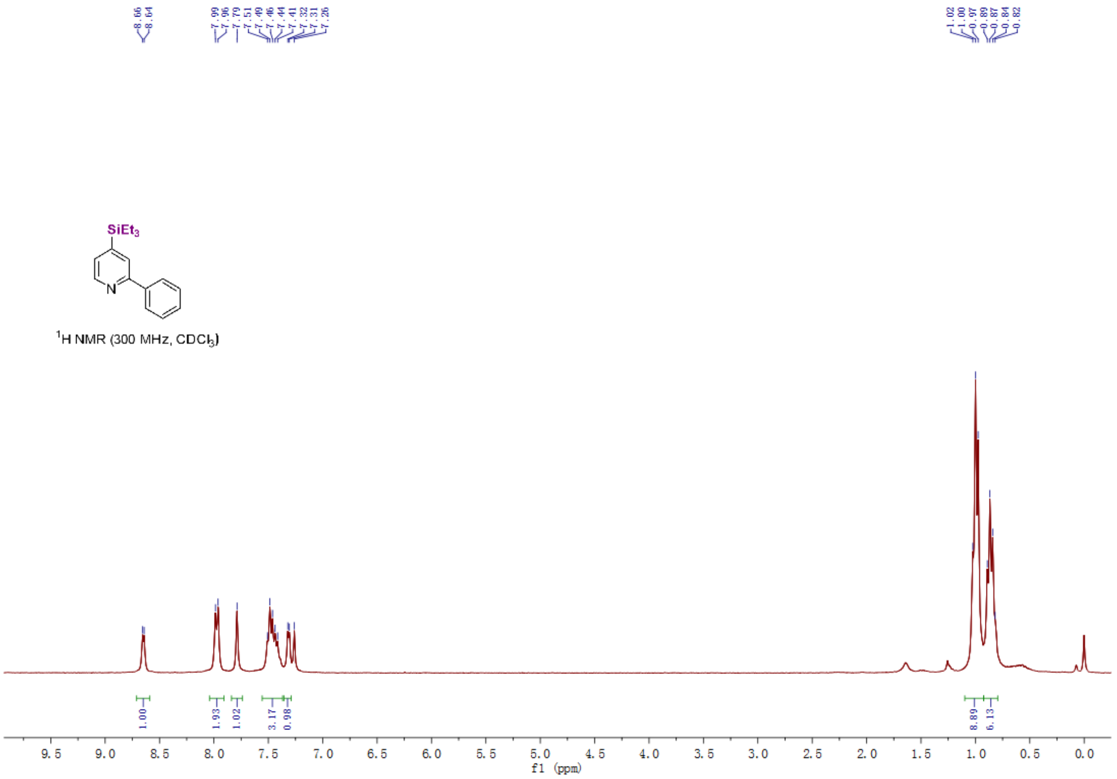


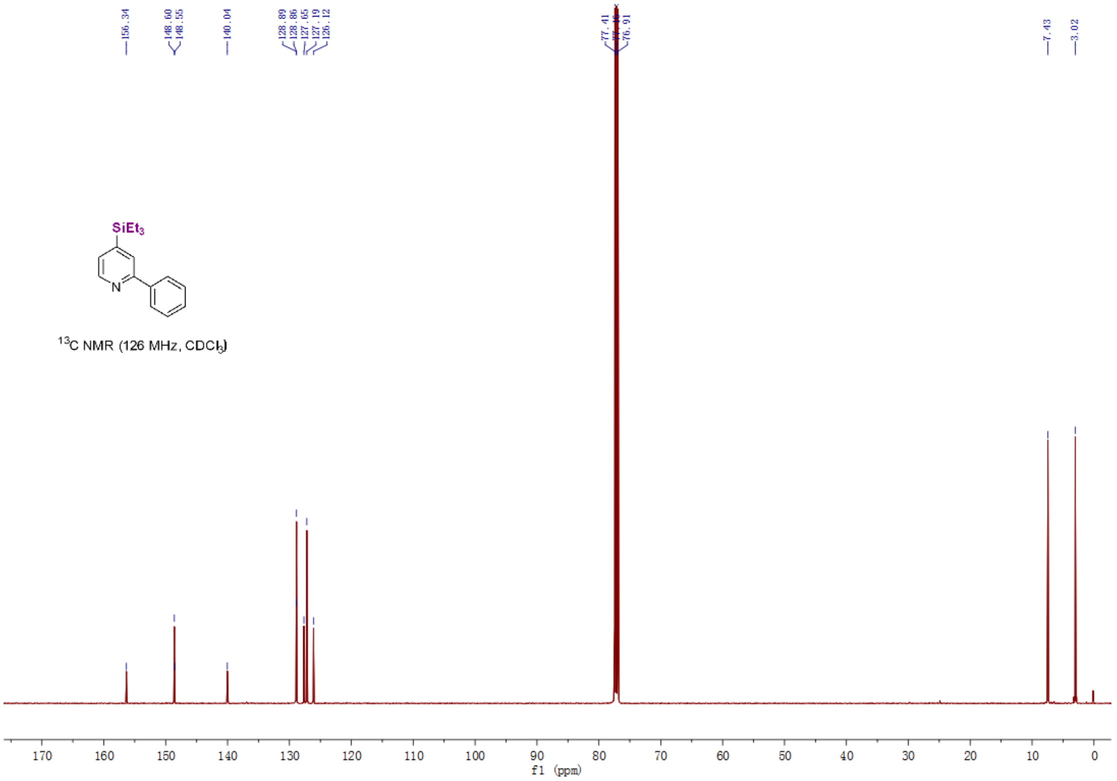


**Supplementary Figure 71**. NMR Spectra of 2-phenyl-4-(triethylsilyl)pyridine.

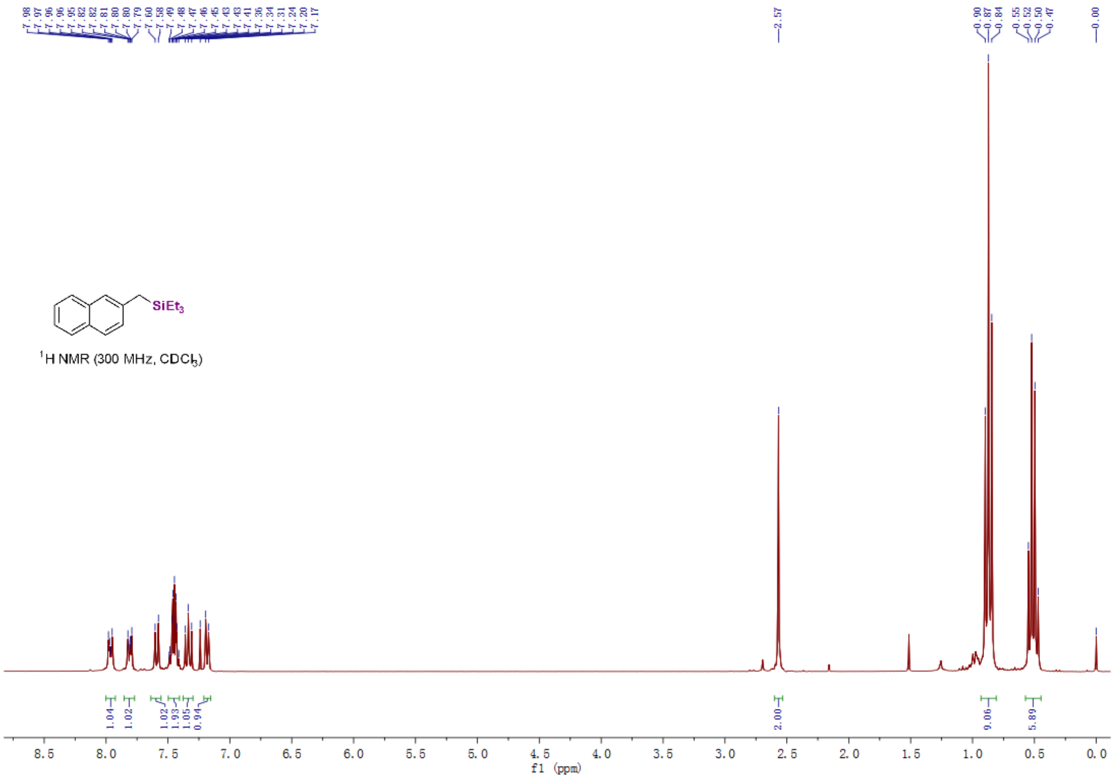


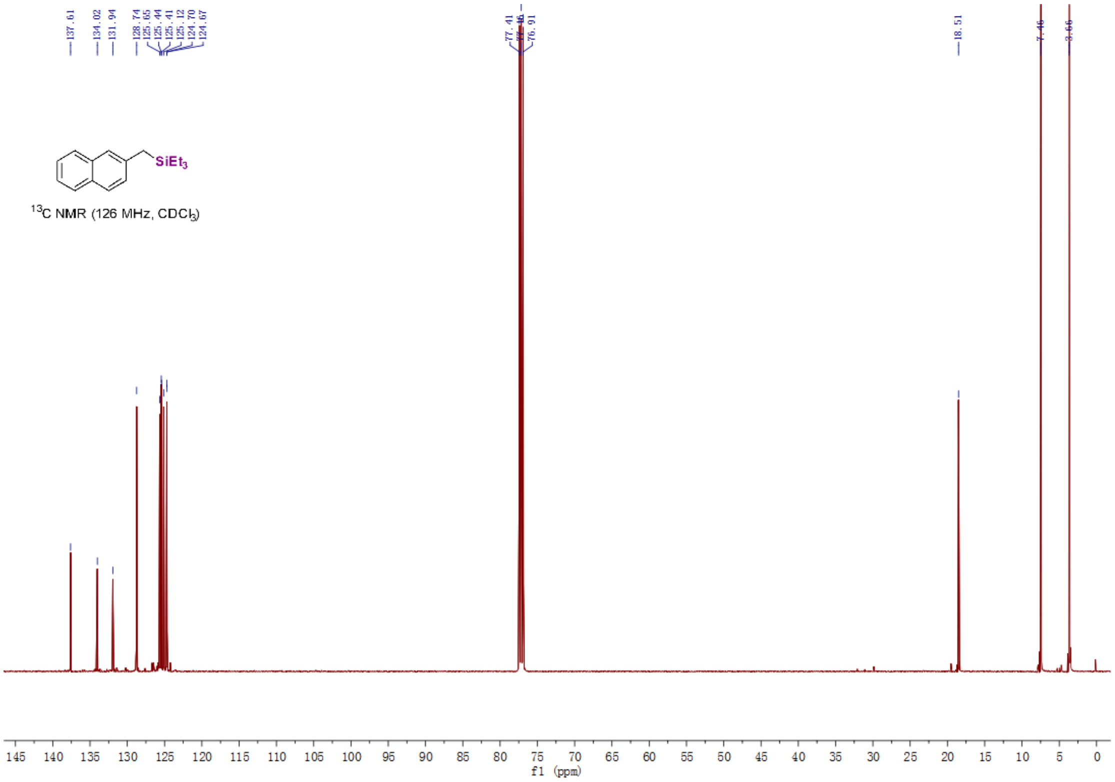


**Supplementary Figure 72**. NMR Spectra of triethyl(naphthalene-2-ylmethyl)silane.

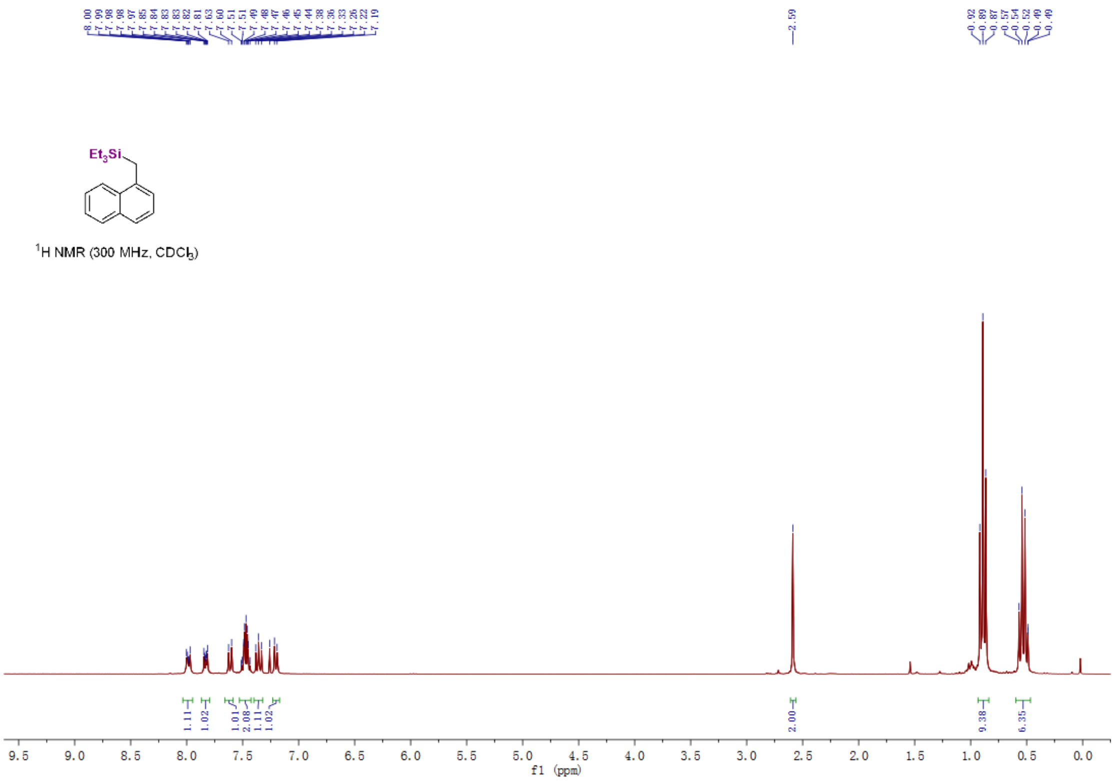


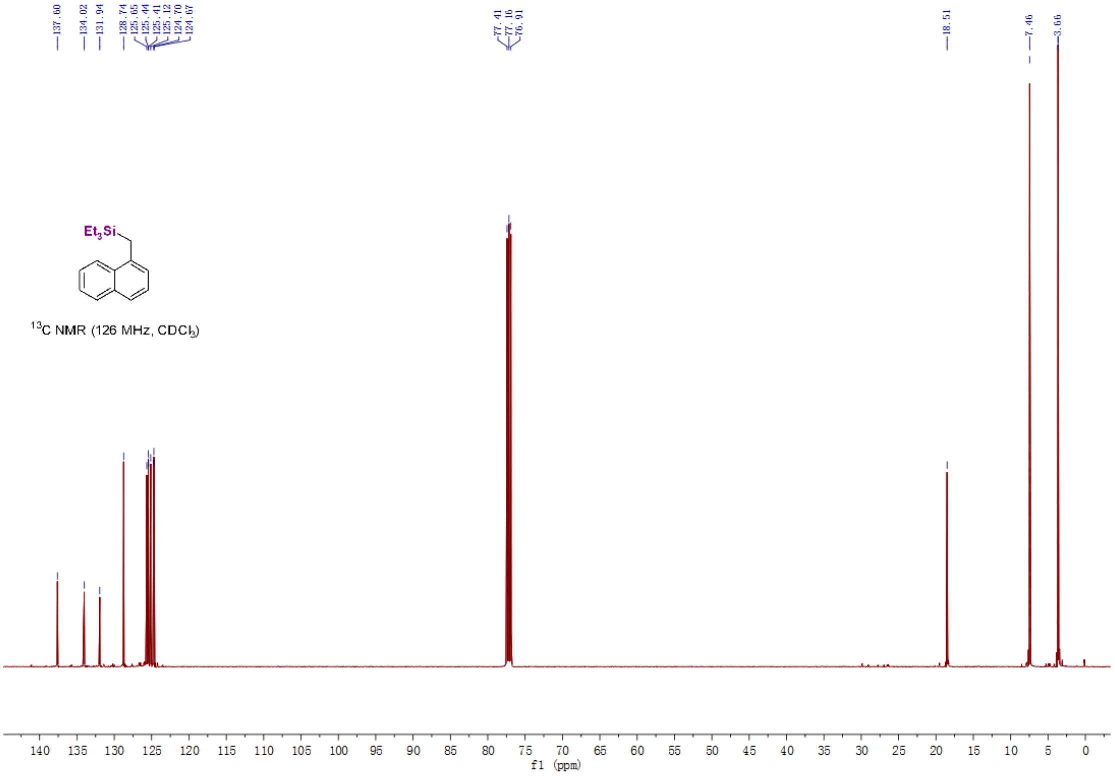


**Supplementary Figure 73**. NMR Spectra of triethyl(naphthalen-1-ylmethyl)silane.

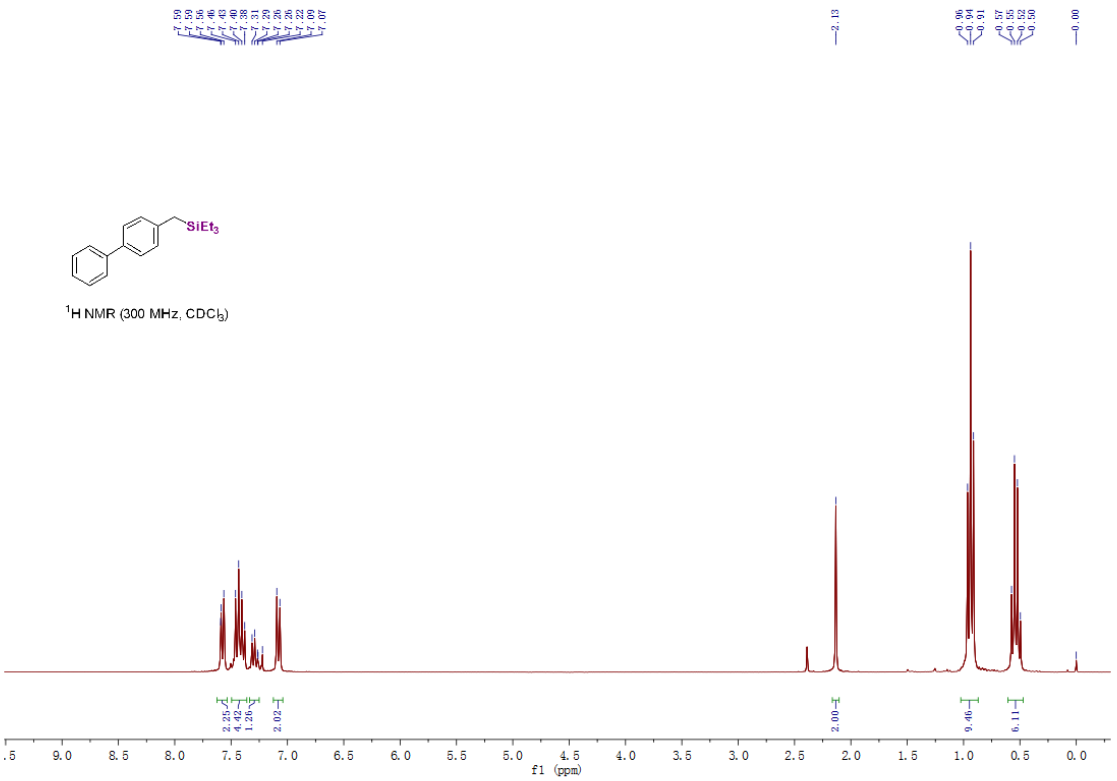


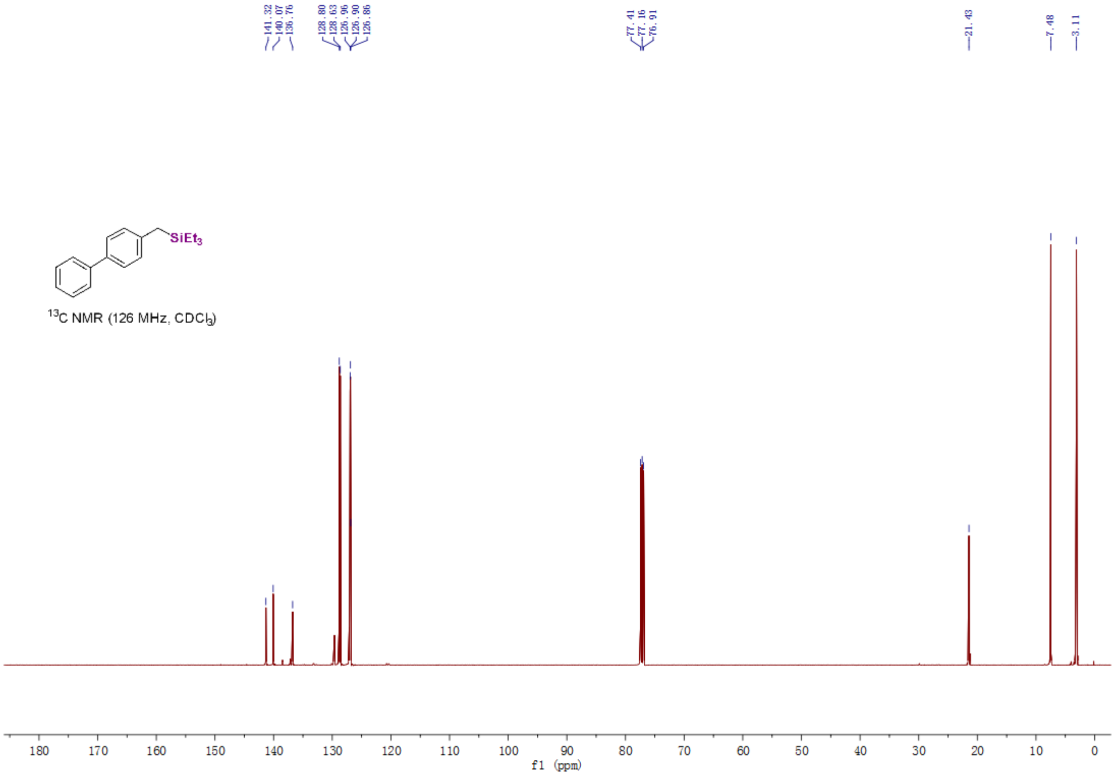


**Supplementary Figure 74**. NMR Spectra of ([1,1'-biphenyl]-4-ylmethyl)triethylsilane.

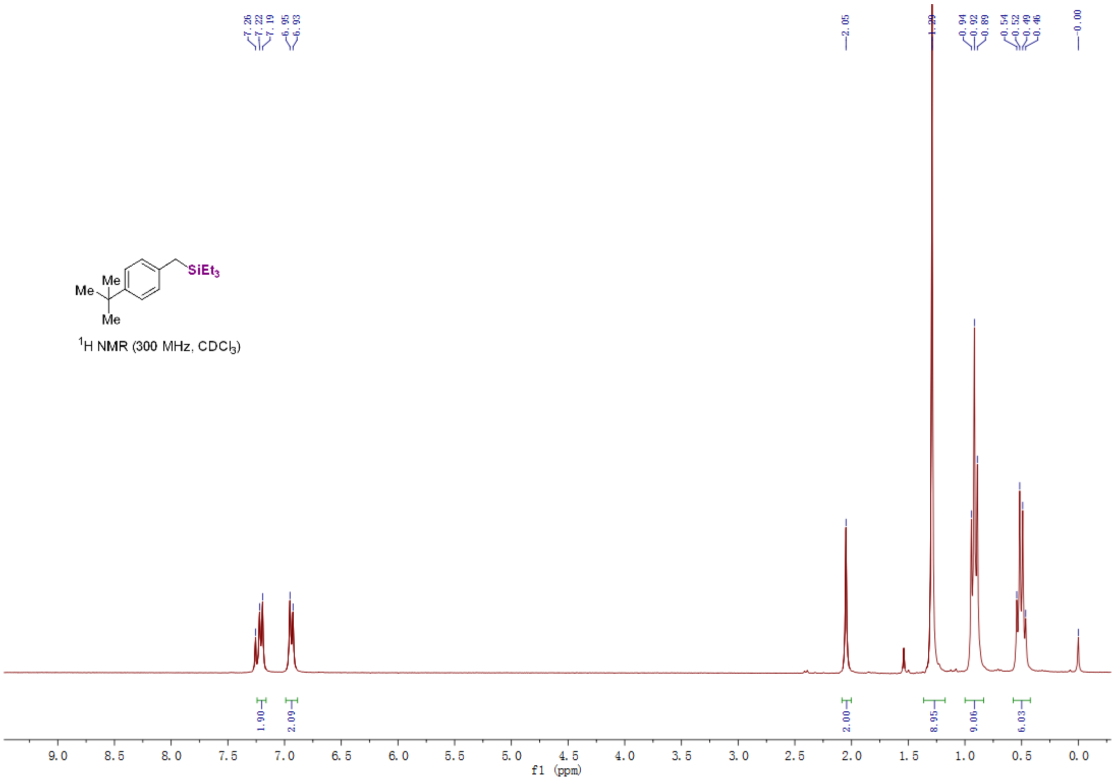


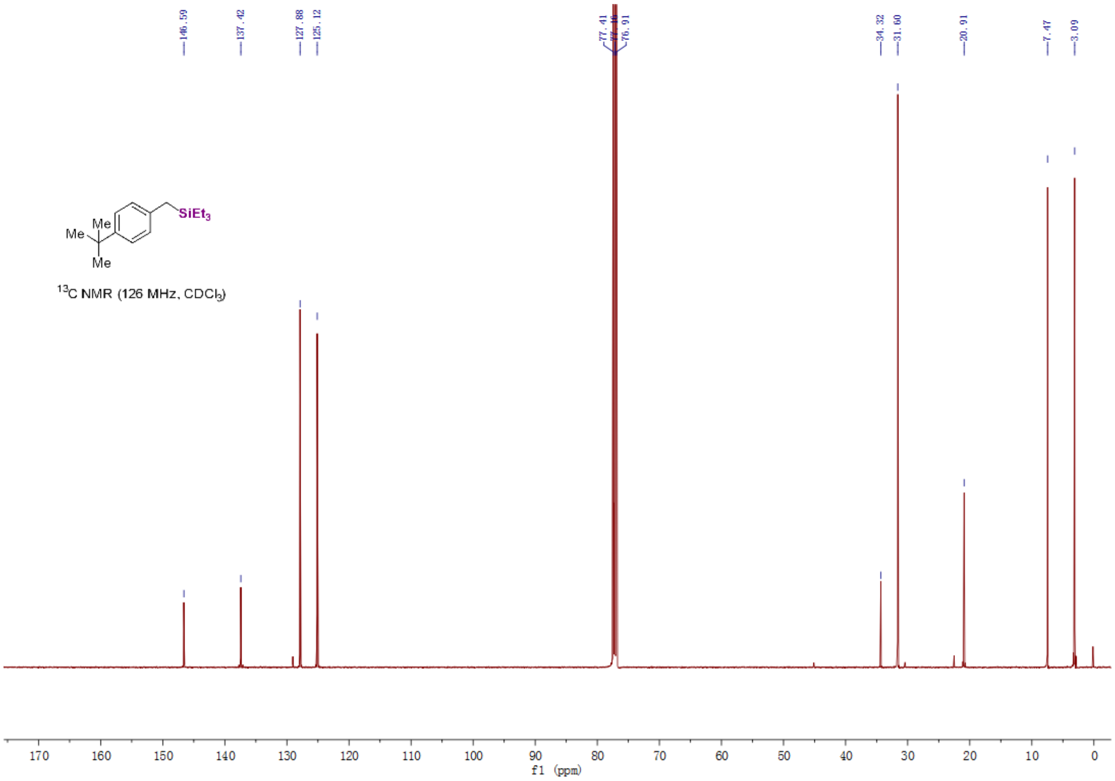


**Supplementary Figure 75**. NMR Spectra of (4-(*tert*-butyl)benzyl)triethylsilane.

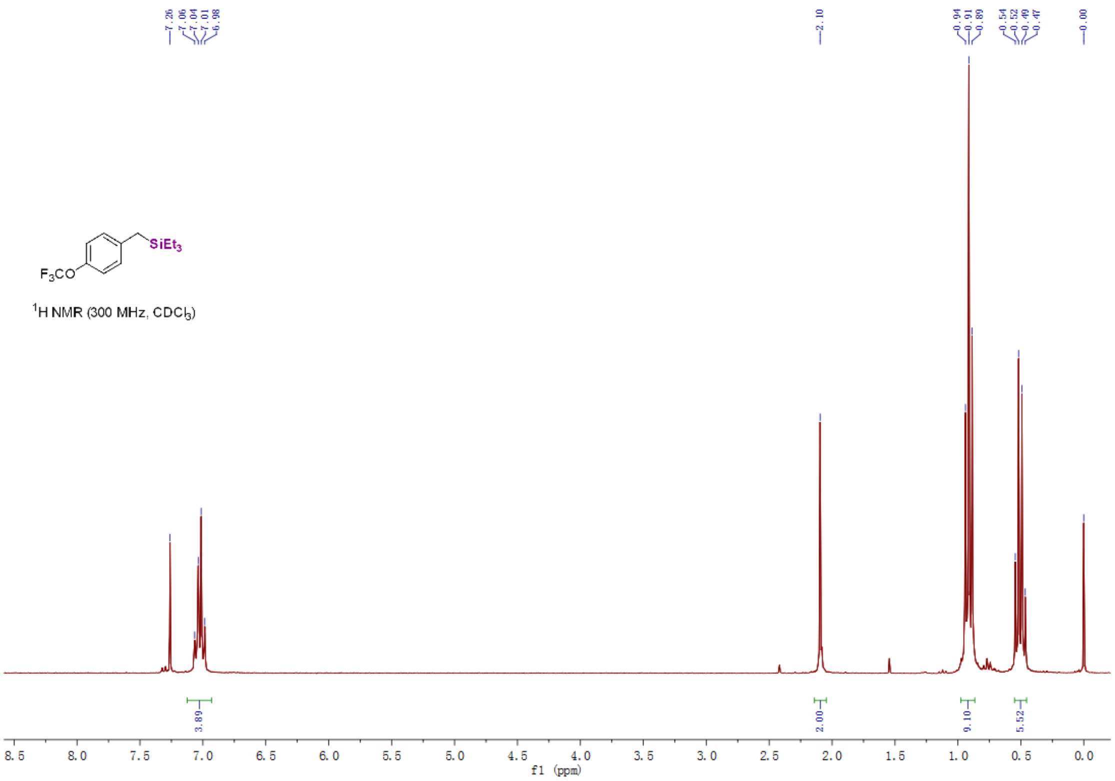
7.26
7.06
7.04
7.01
6.98
2.10
0.94
0.91
0.89
0.54
0.52
0.49
0.47
0.00
SiEt3
F3CO
1H NMR (300 MHz, CDCl3)
3.89
2.00
9.10
5.52
8.5
8.0
7.5
7.0
6.5
6.0
5.5
5.0
4.5
4.0
3.5
3.0
2.5
2.0
1.5
1.0
0.5
0.0
f1 (ppm)

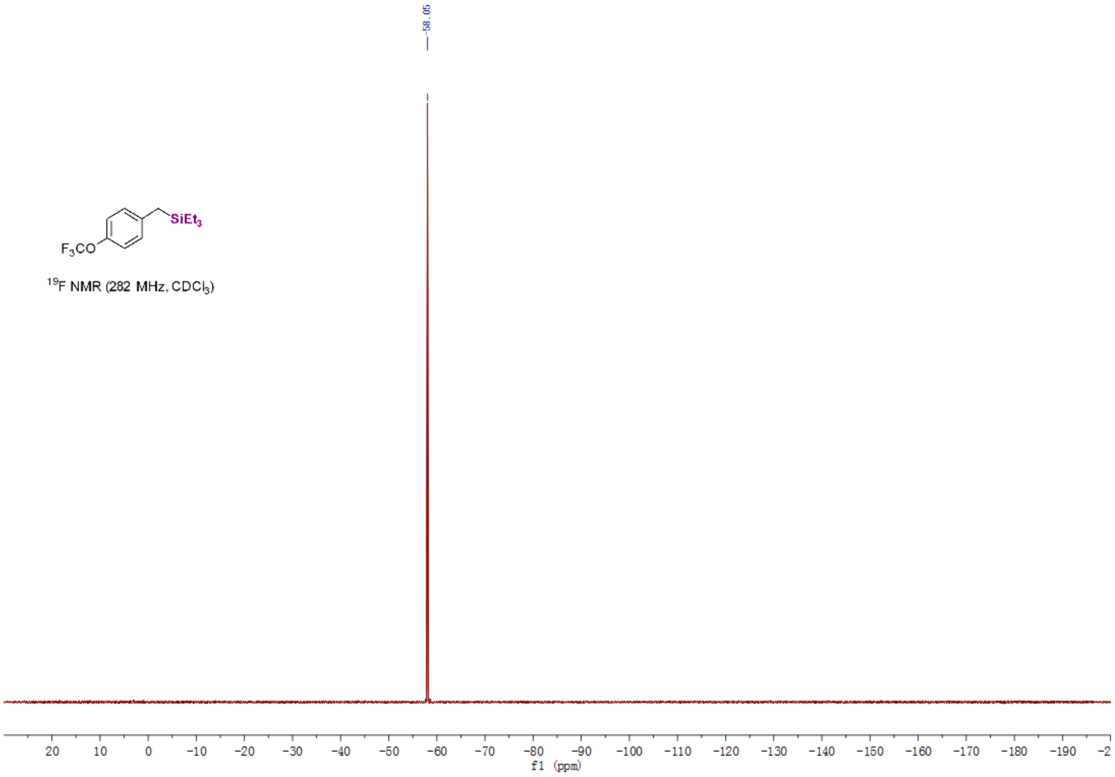
-58.05
SiEt3
F3CO
19F NMR (282 MHz, CDCl3)
20
10
0
-10
-20
-30
-40
-50
-60
-70
-80
-90
-100
-110
-120
-130
-140
-150
-160
-170
-180
-190
f1 (ppm)

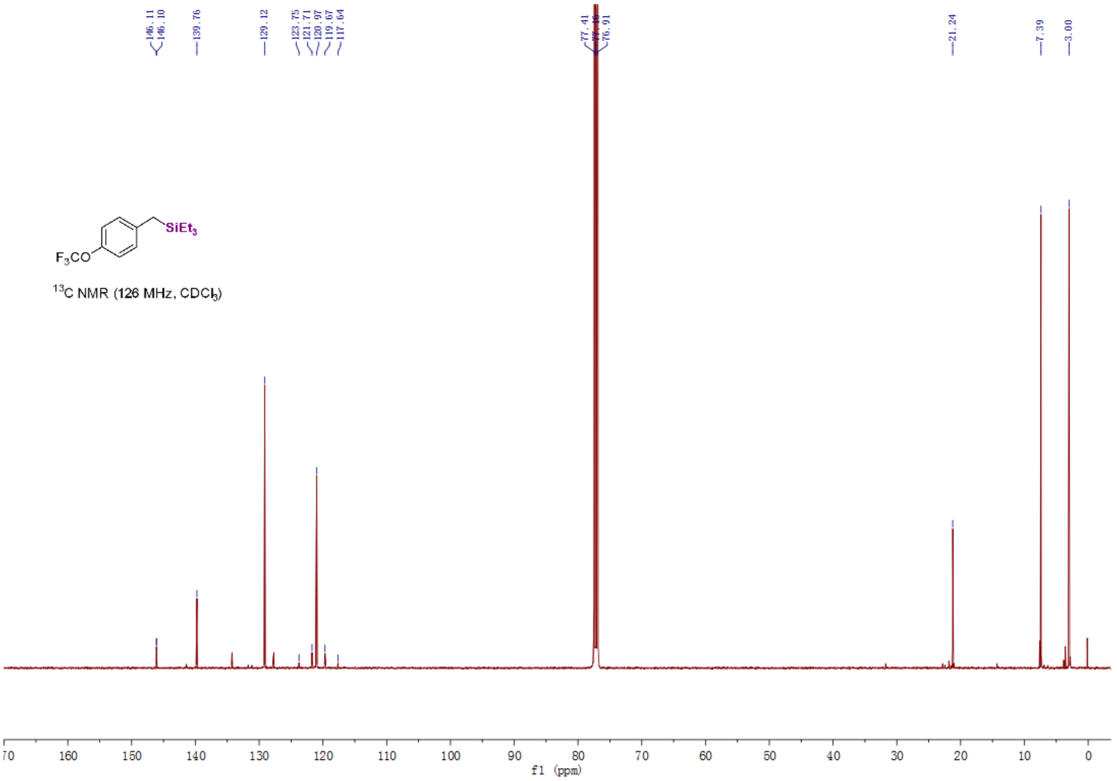


**Supplementary Figure 76**. NMR Spectra of triethyl(4-(trifluoromethoxy)benzyl)silane.

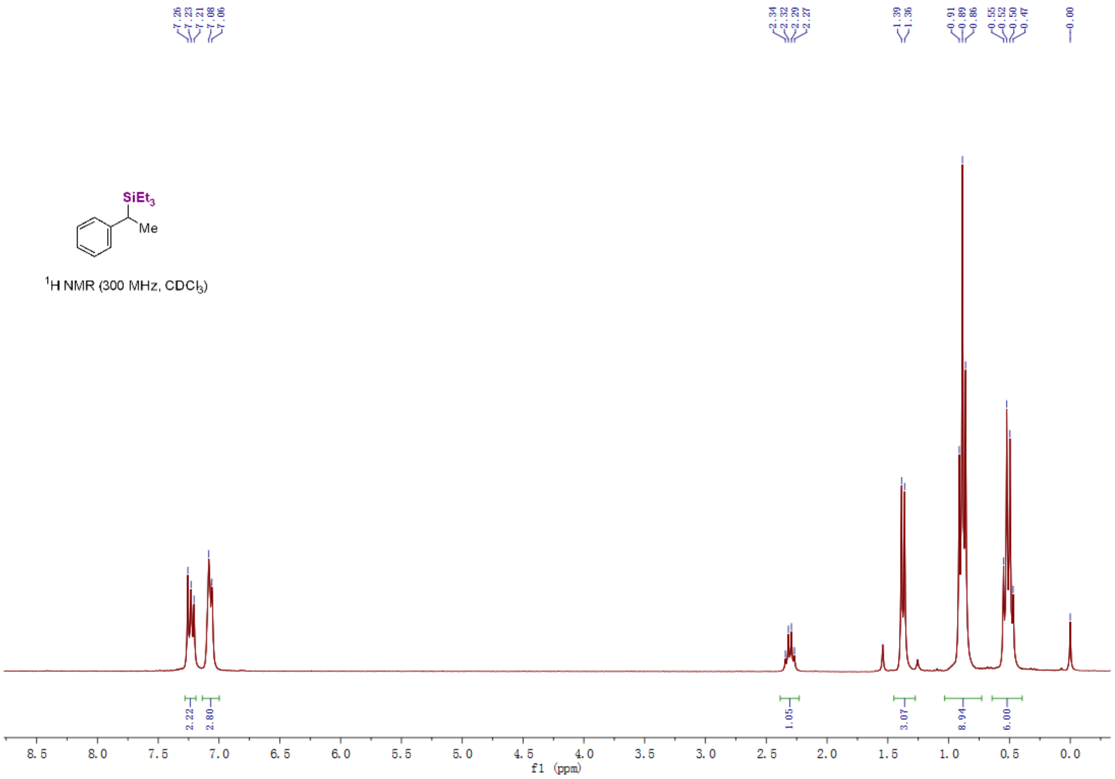


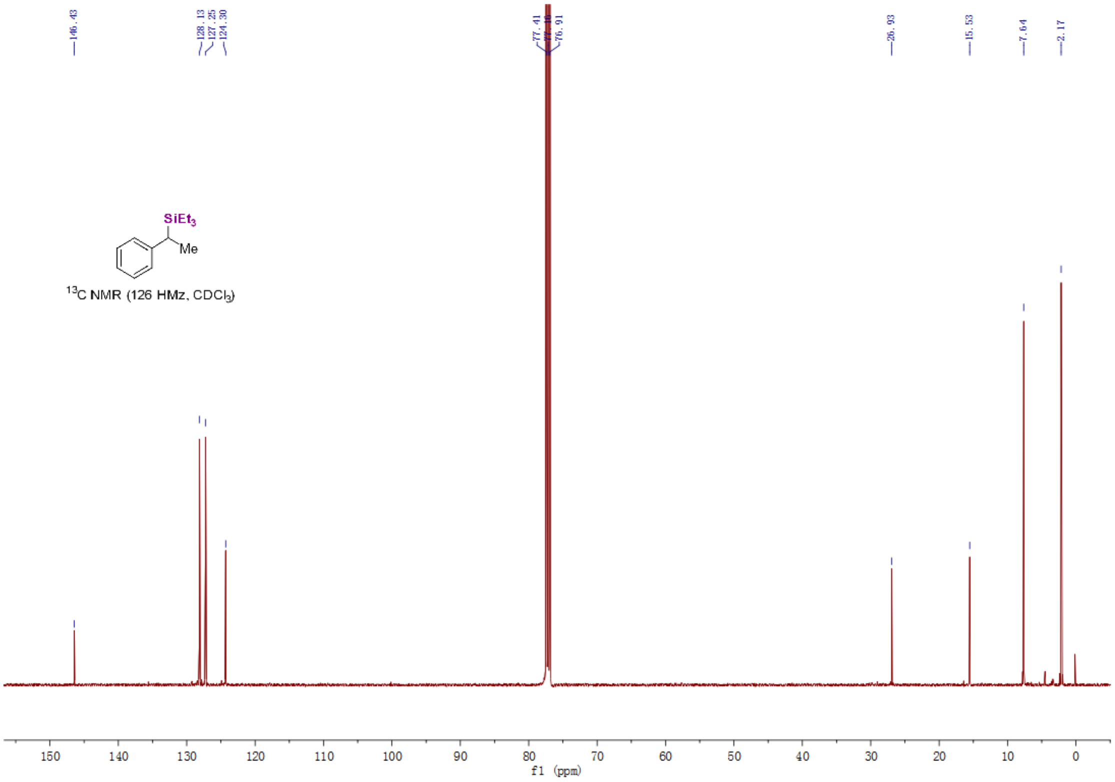


**Supplementary Figure 77**. NMR Spectra of triethyl(1-phenylethyl)silane.

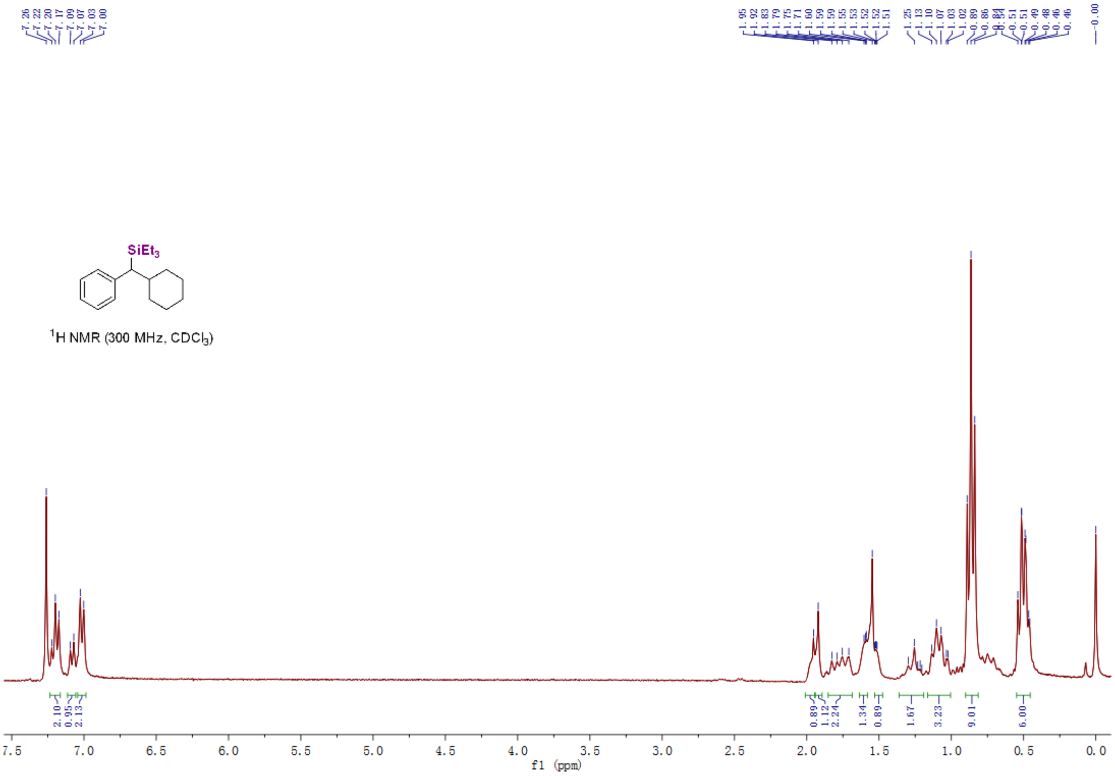


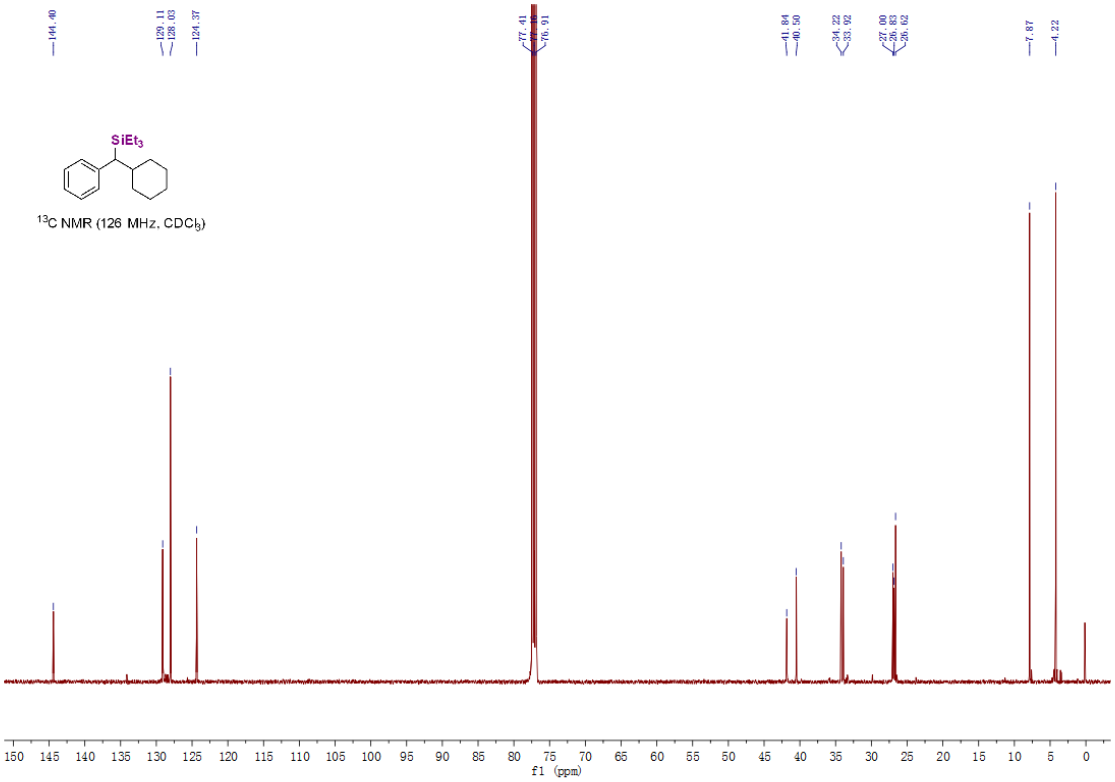


**Supplementary Figure 78**. NMR Spectra of (cyclohexyl(phenyl)methyl)triethylsilane.

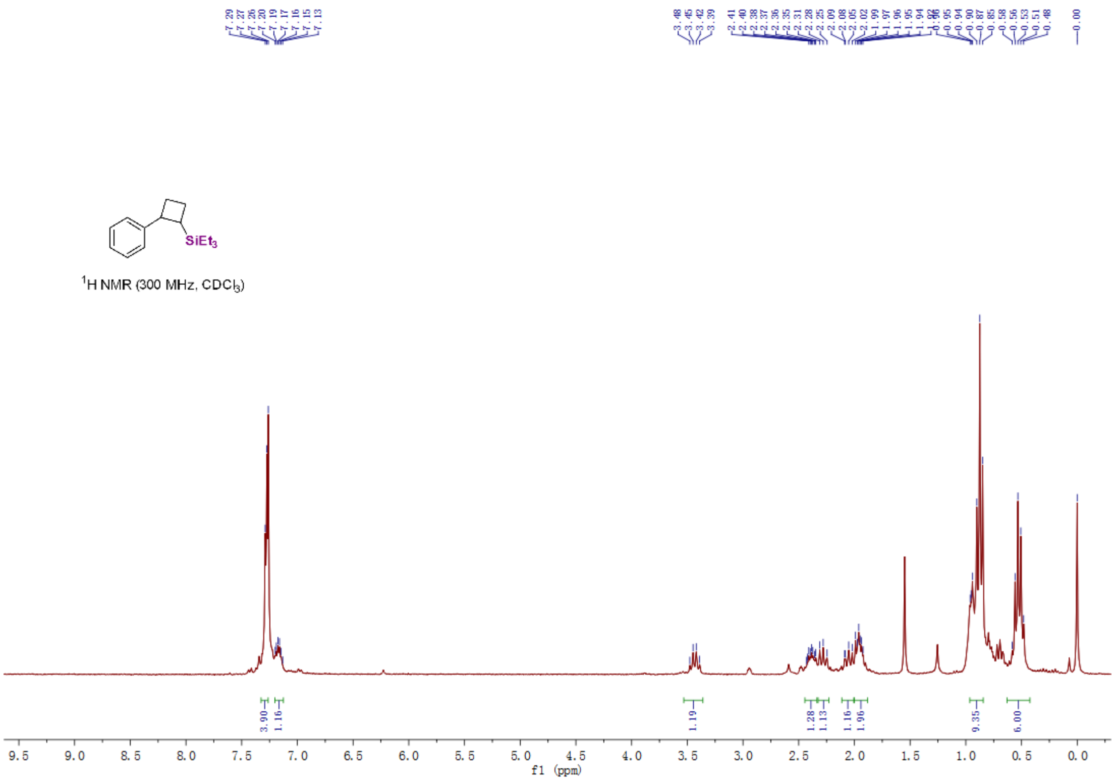


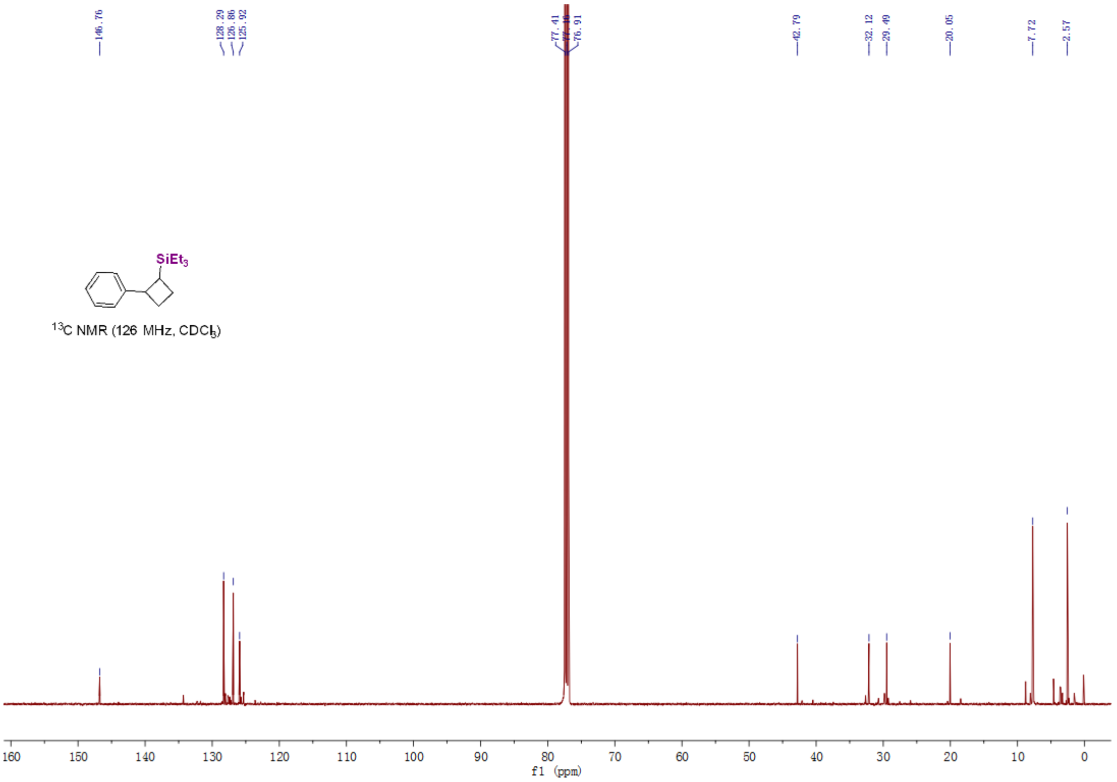


**Supplementary Figure 79**. NMR Spectra of (2-phenylcyclobutyl)triethylsilane.

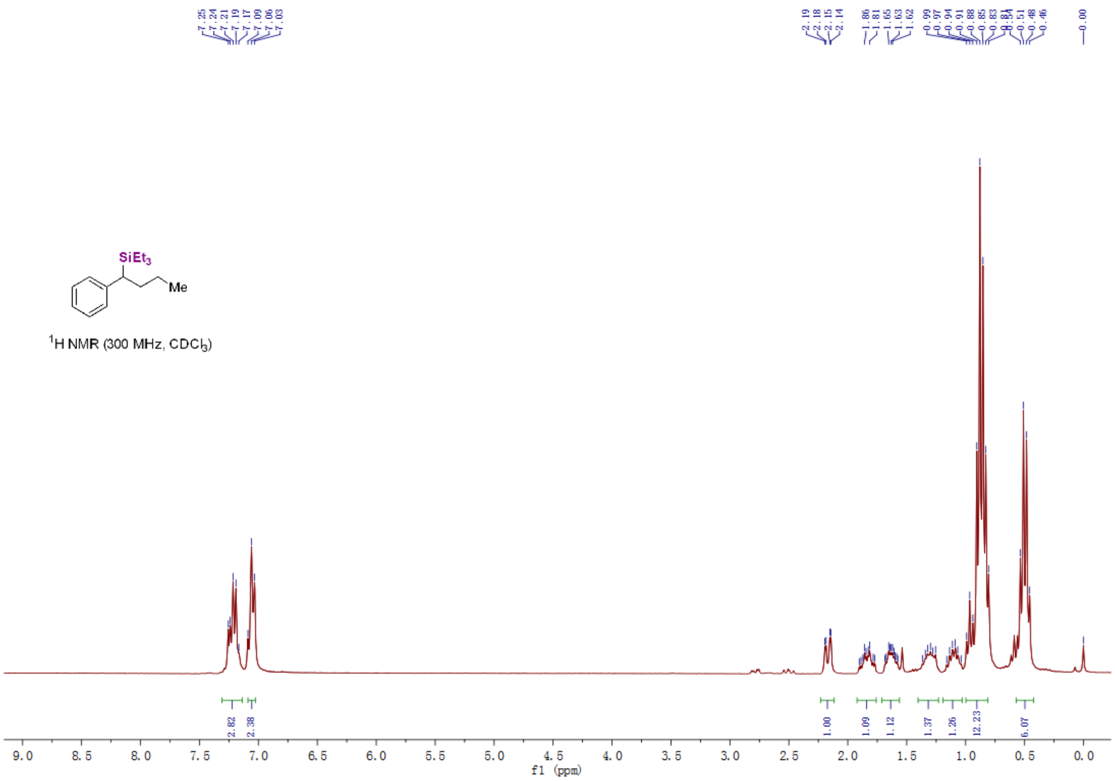


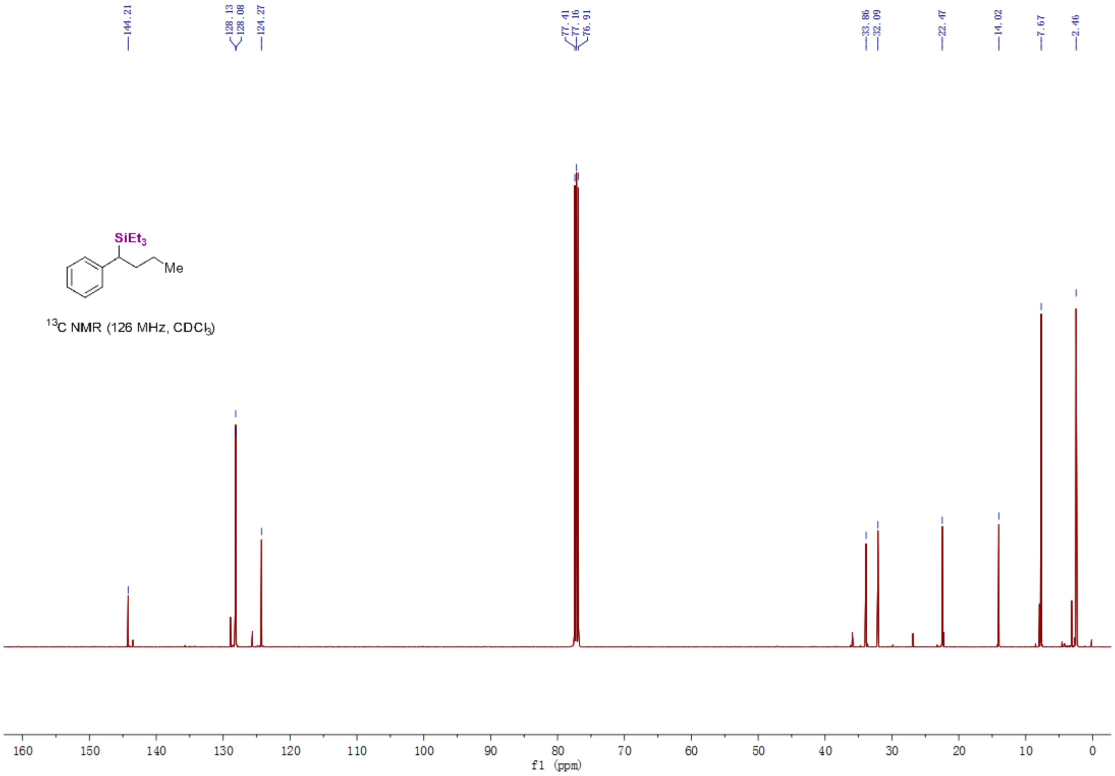


**Supplementary Figure 80**. NMR Spectra of (4-phenylbutyl)triethylsilane.

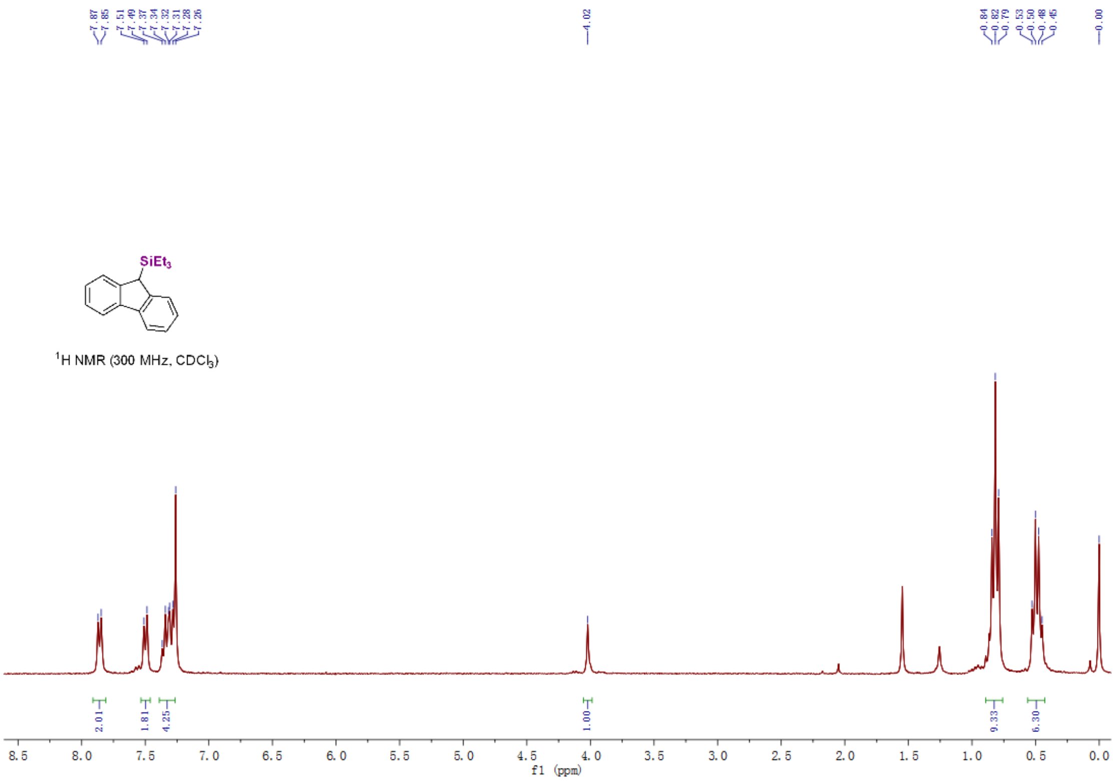


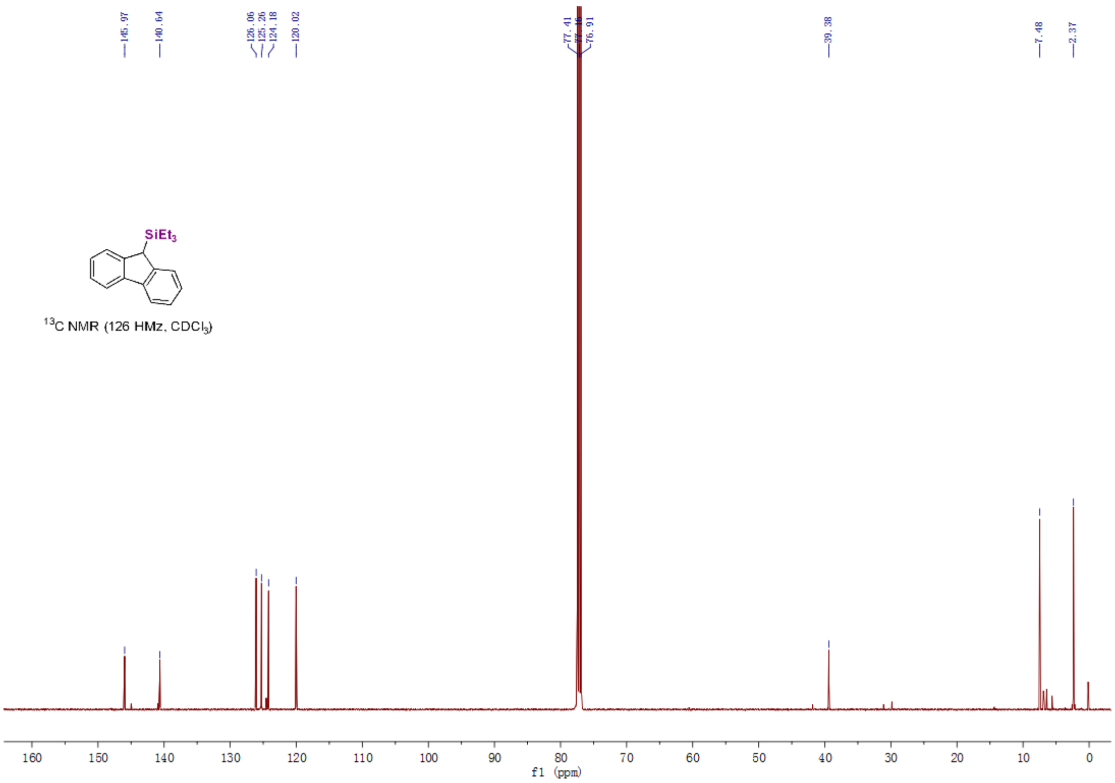


**Supplementary Figure 81**. NMR Spectra of (9*H*-fluoren-9-yl)triethylsilane.

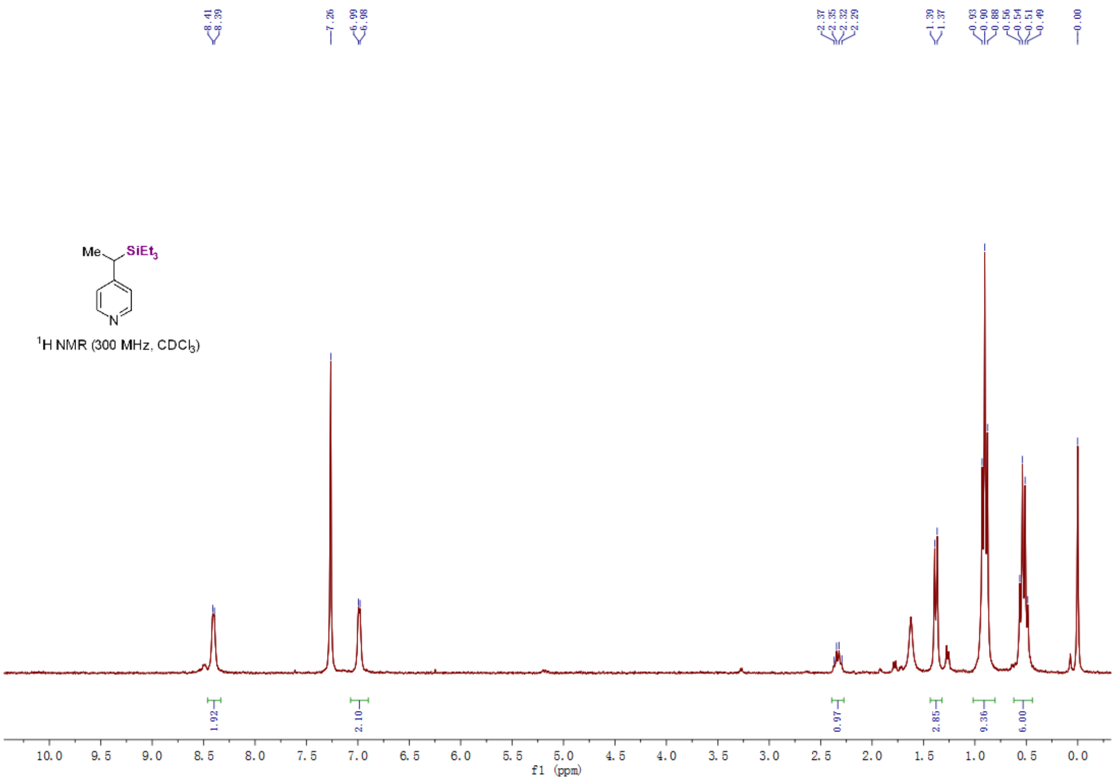


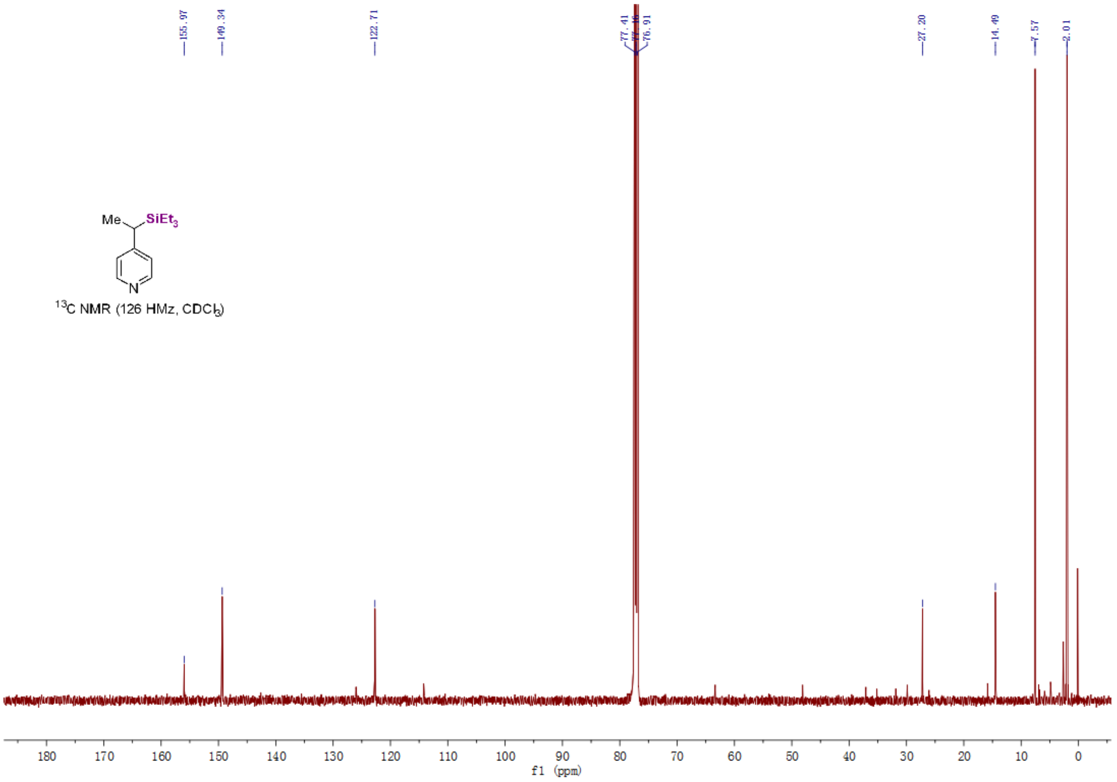


**Supplementary Figure 82**. NMR Spectra of 4-(1-(triethylsilyl)ethyl)pyridine.

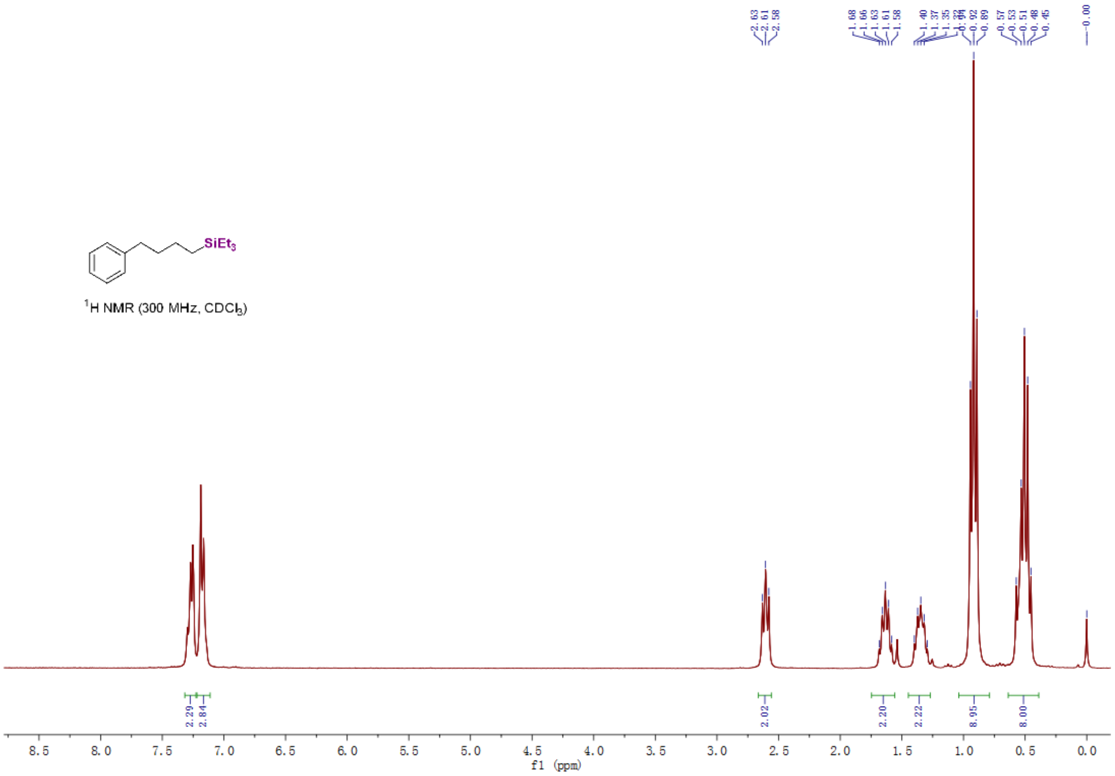


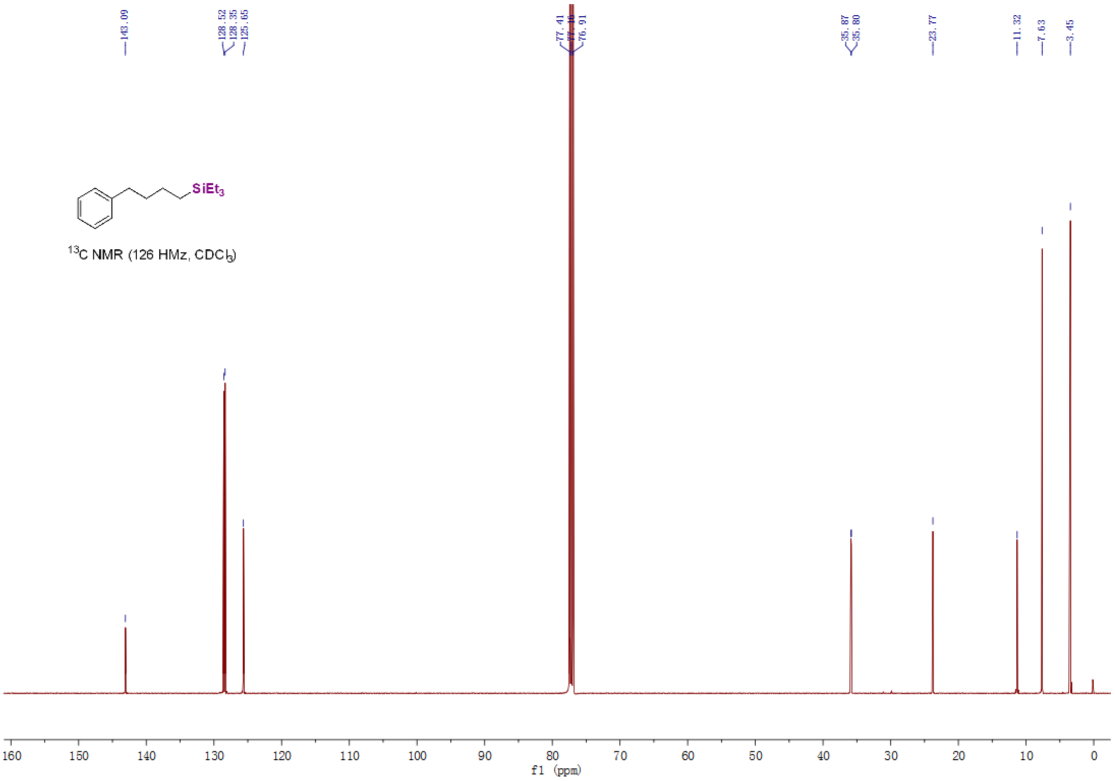


**Supplementary Figure 83**. NMR Spectra of (4-phenylbutyl)triethylsilane.

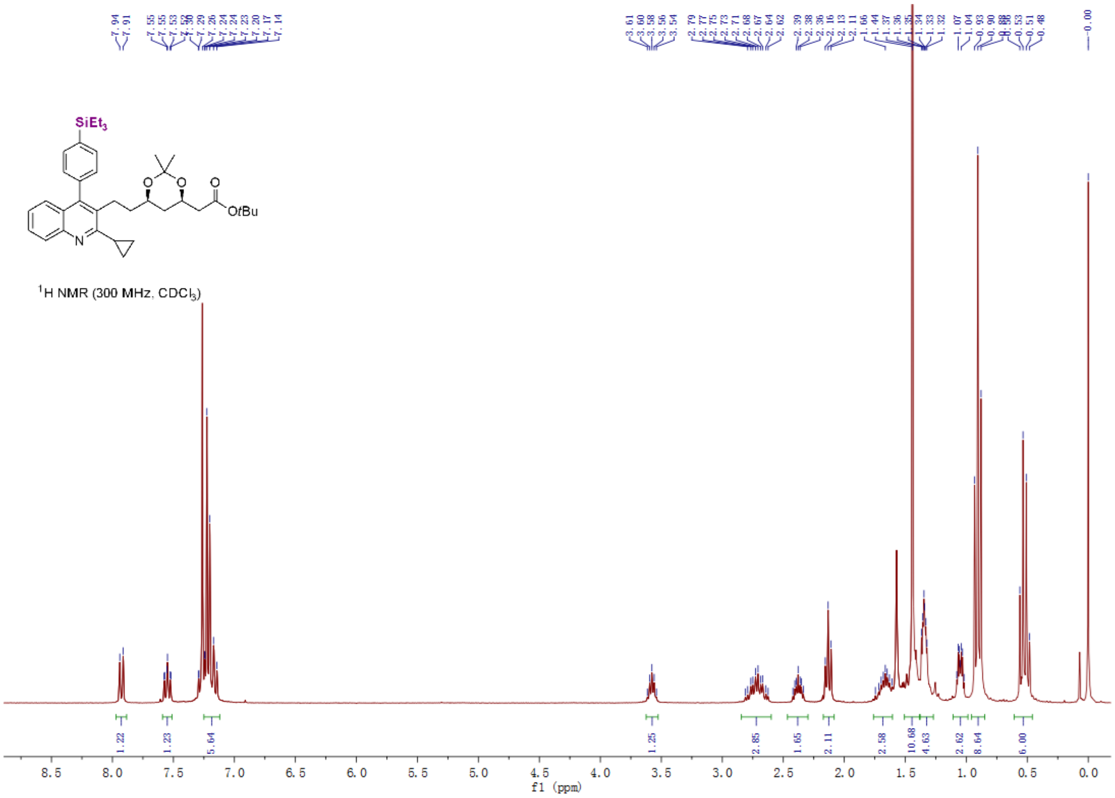


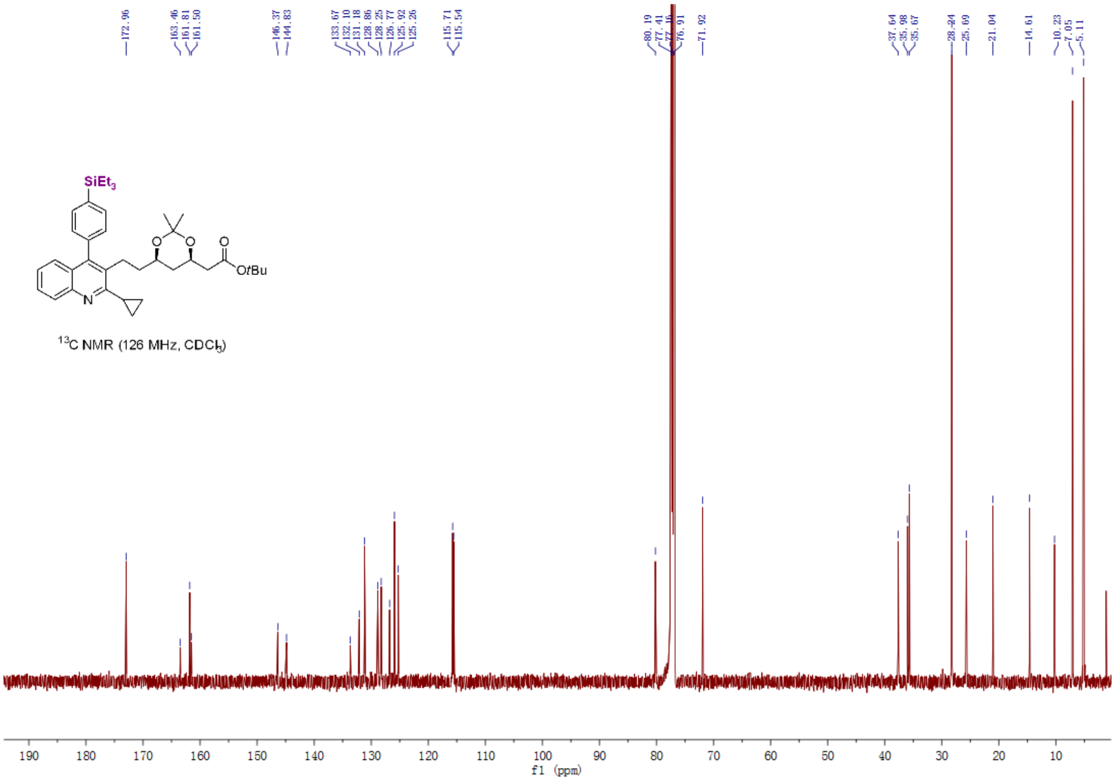


**Supplementary Figure 84**. NMR Spectra of *tert*-butyl 2-((4*R*,6*R*)-6-(2-(2-cyclopropyl-4-(4-fluorophenyl)quinolin-3-yl)ethyl)-2,2-dimethyl-1,3-dioxan-4-yl)acetate.

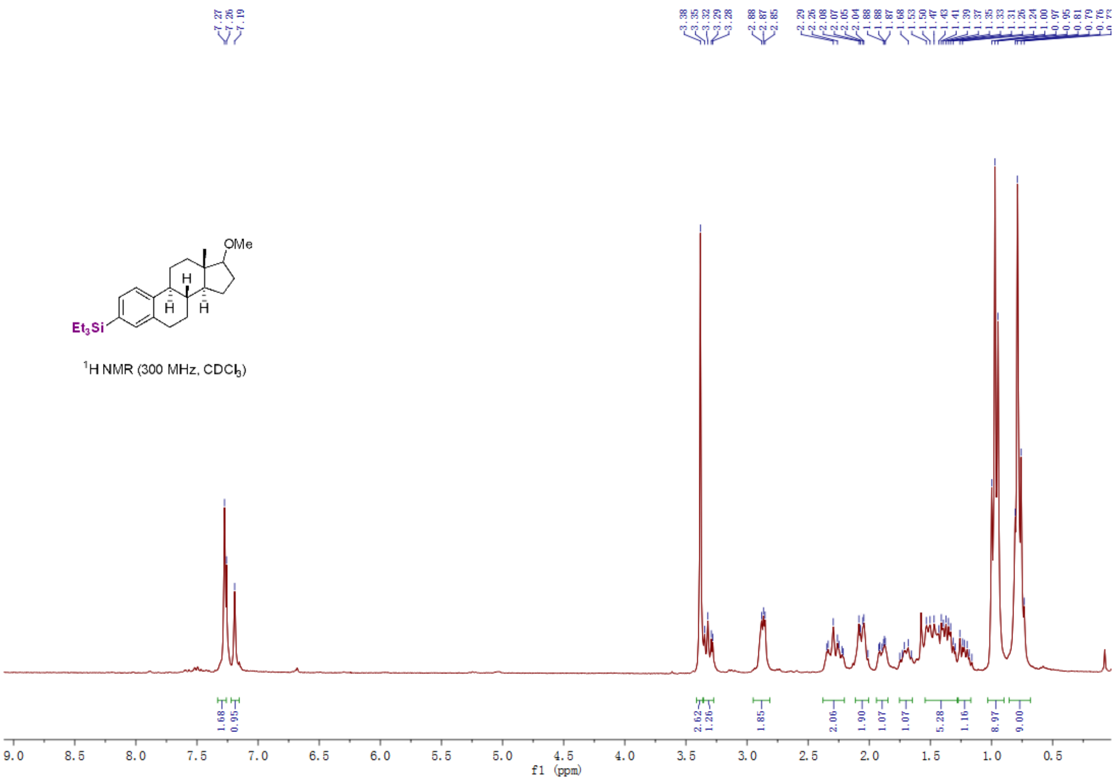


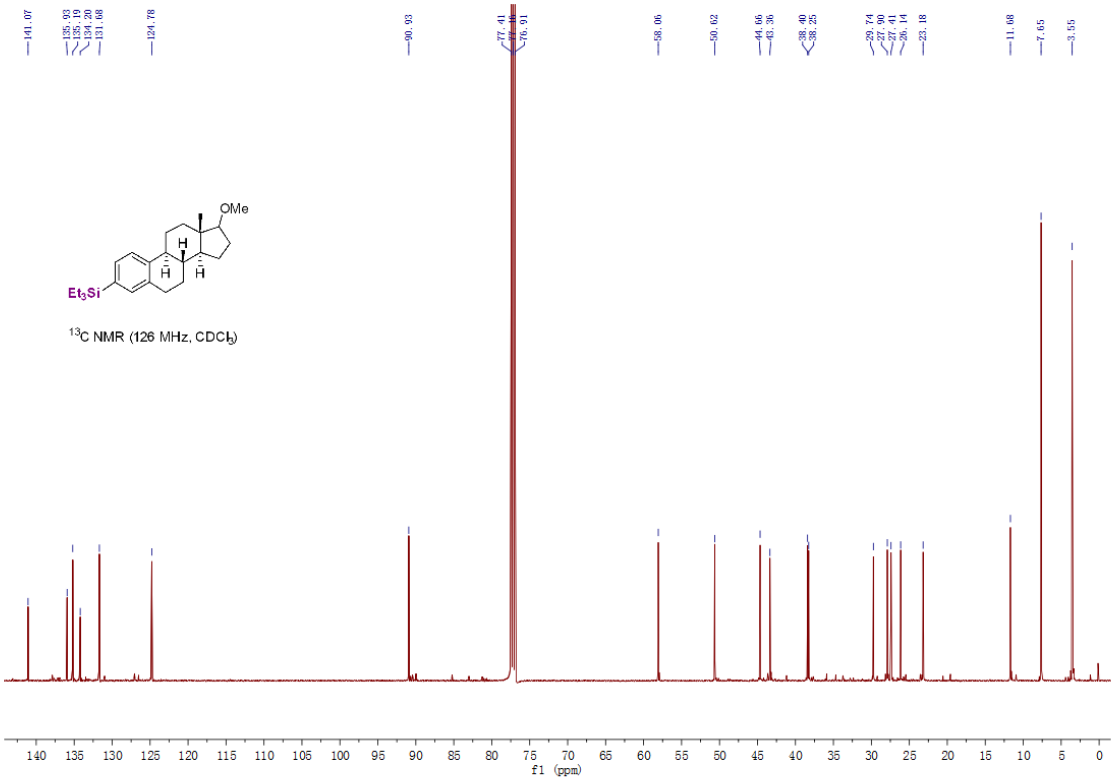


**Supplementary Figure 85**. NMR Spectra of triethyl((8*R*,9*S*,13*S*,14*S*)-17-methoxy-13-methyl-7,8,9,11,12,13,14,15,16,17-decahydro-6*H*-cyclopenta[a]phenanthren-3-yl)silane.

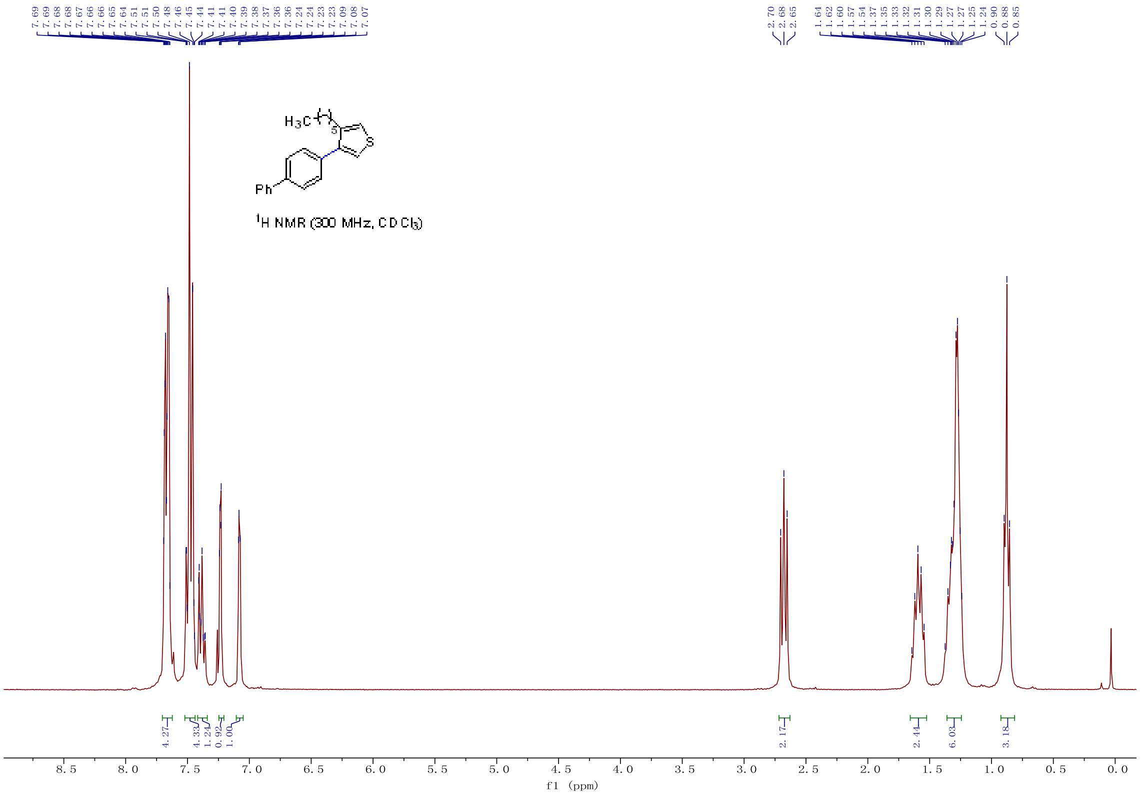


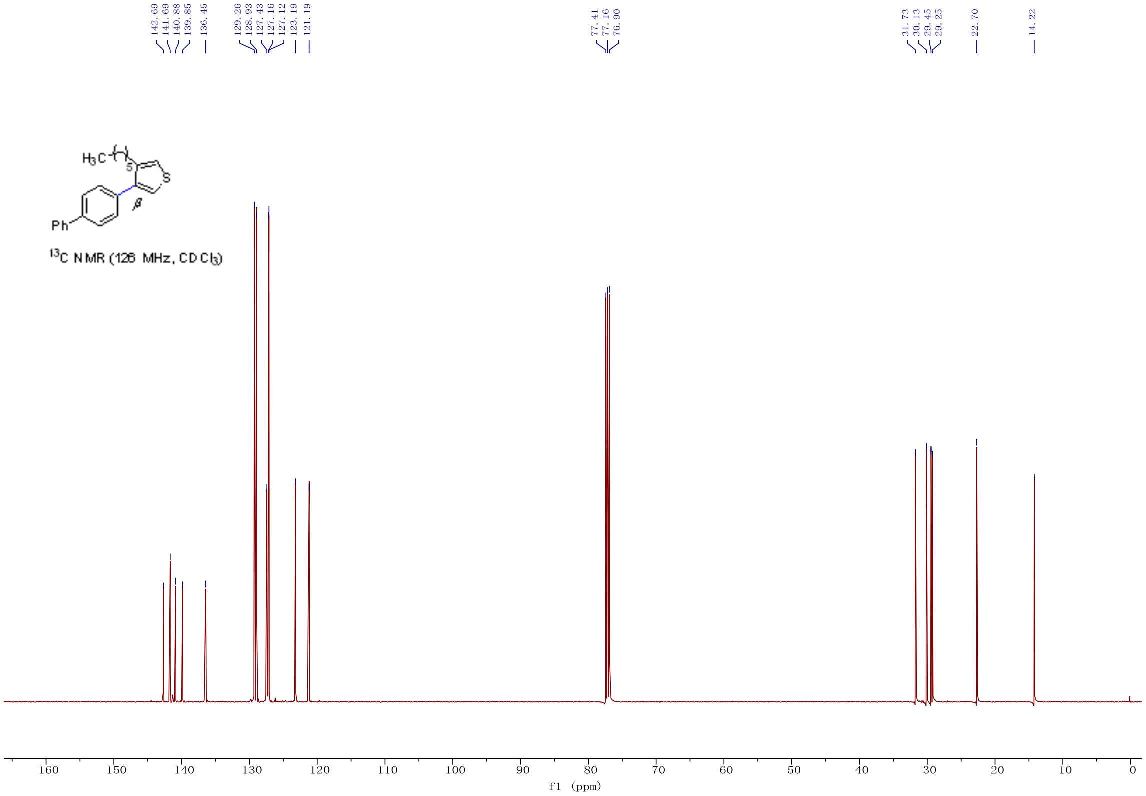


**Supplementary Figure 86**. NMR Spectra of 3-([1,1'-biphenyl]-4-yl)-4-hexylthiophene.

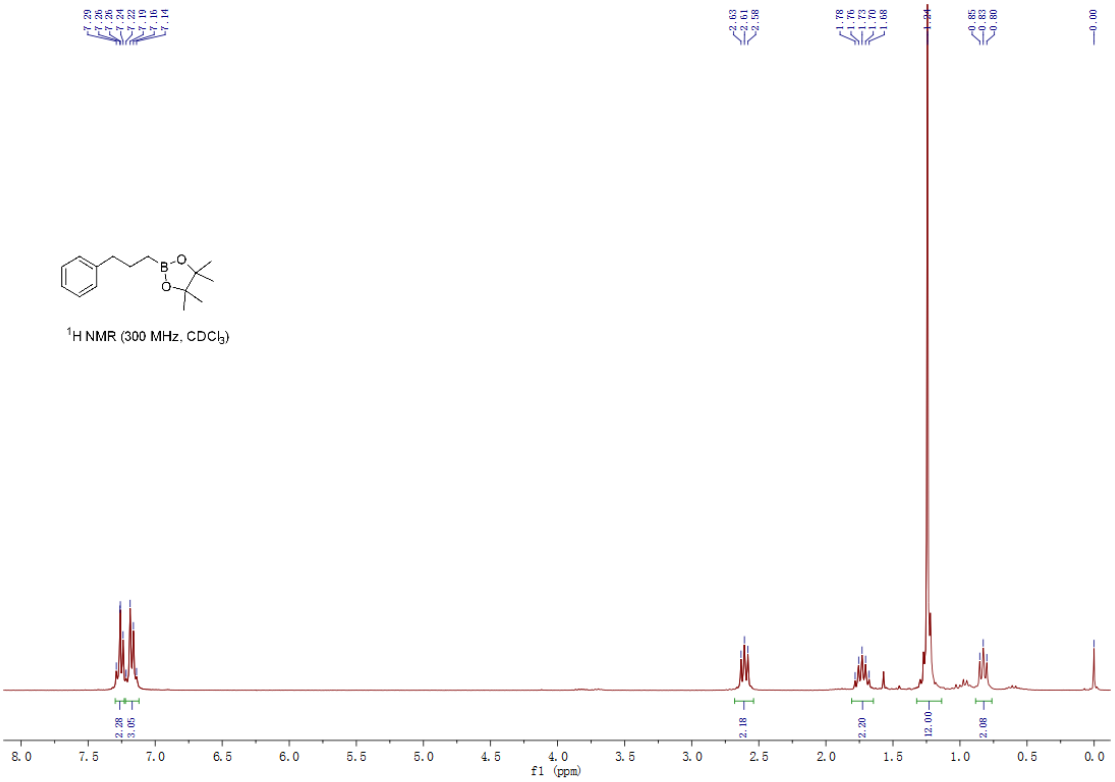

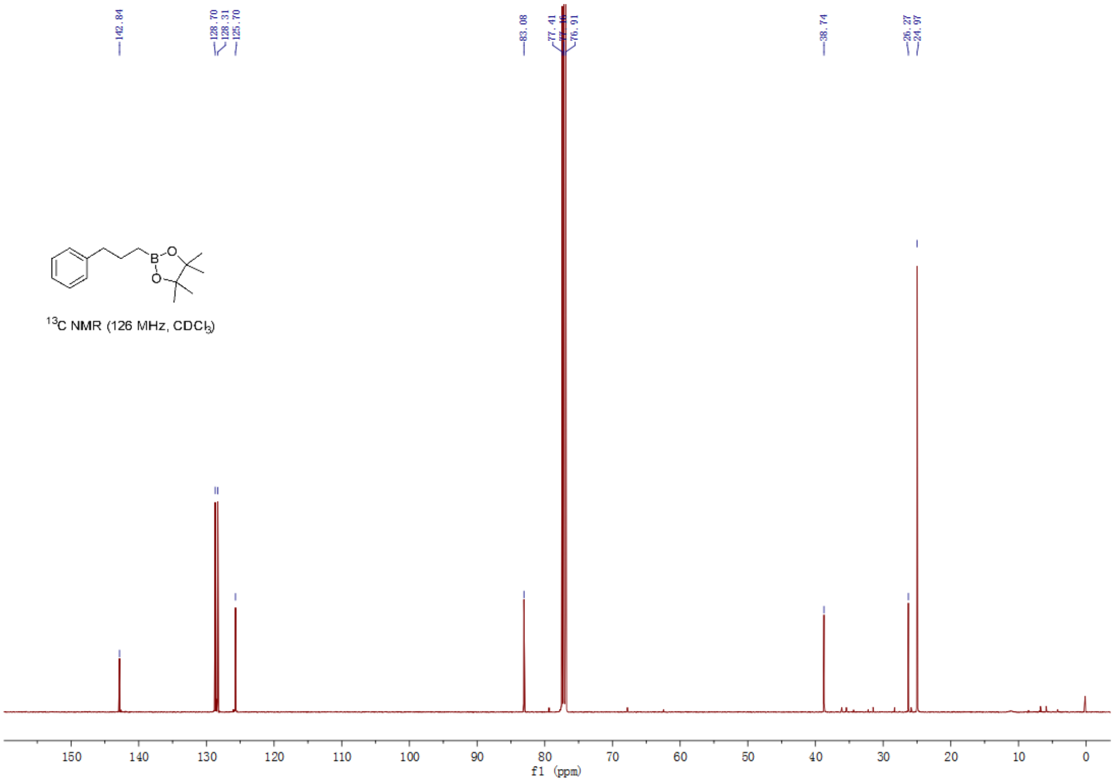

**Supplementary Figure 87**. NMR Spectra of 4,4,5,5-tetramethyl-2-(3-phenylpropyl)-1,3,2-dioxaborolane.

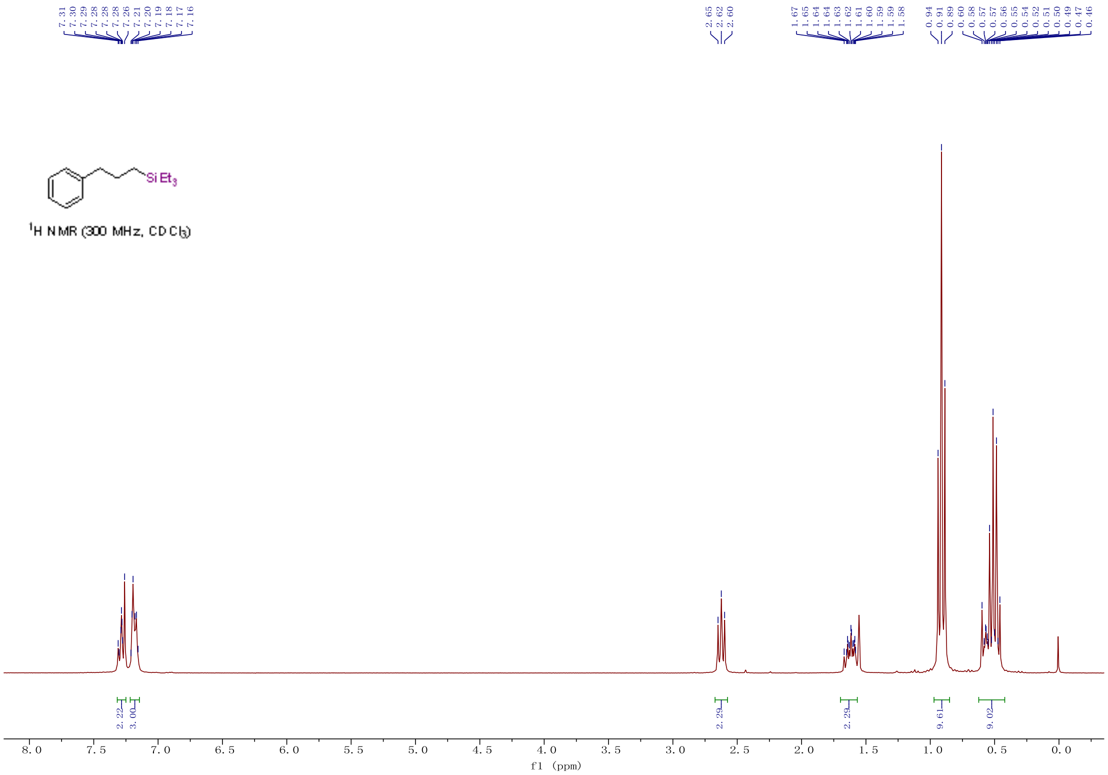

**Supplementary Figure 88**. NMR Spectra of triethyl(3-phenylpropyl)silane.

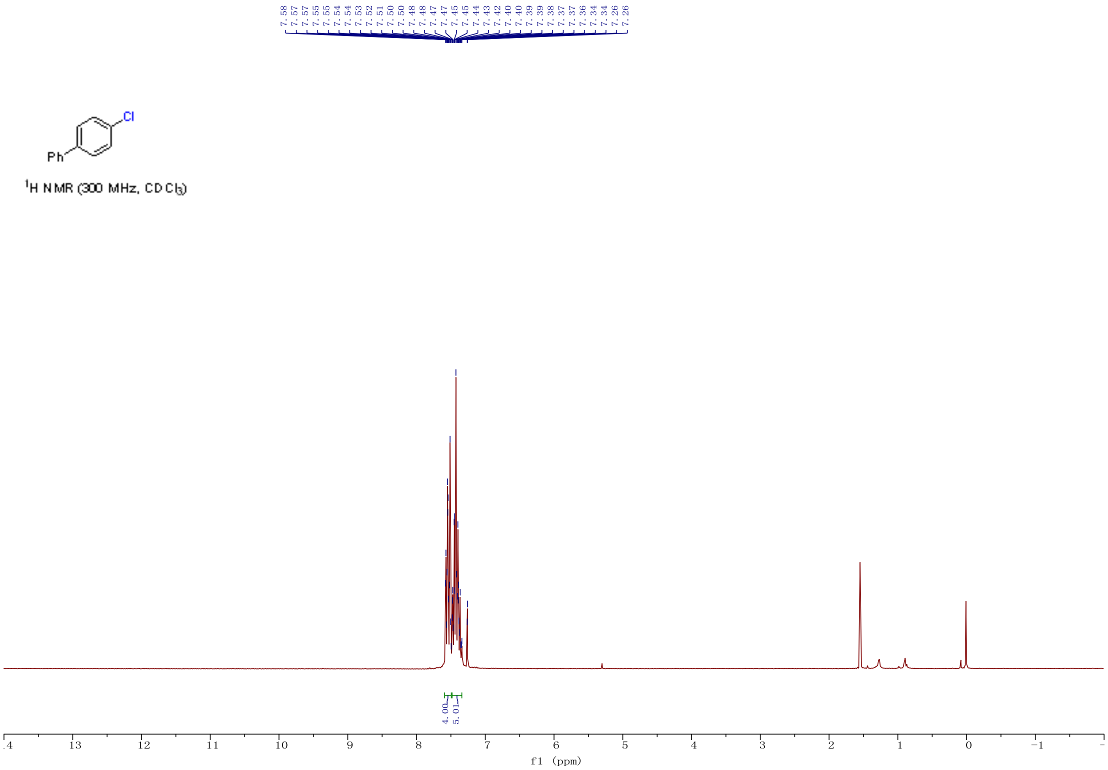

**Supplementary Figure 89**. NMR Spectra of 4-chloro-1,1'-biphenyl.

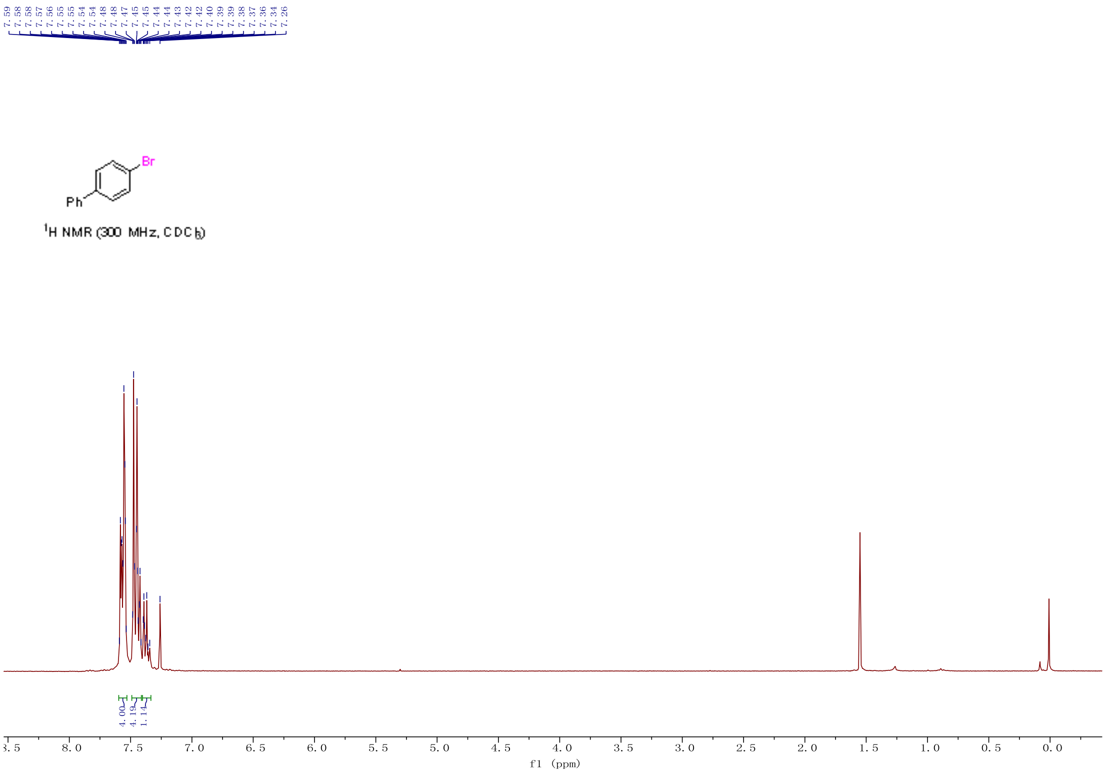


**Supplementary Figure 90**. NMR Spectra of 4-bromo-1,1'-biphenyl.

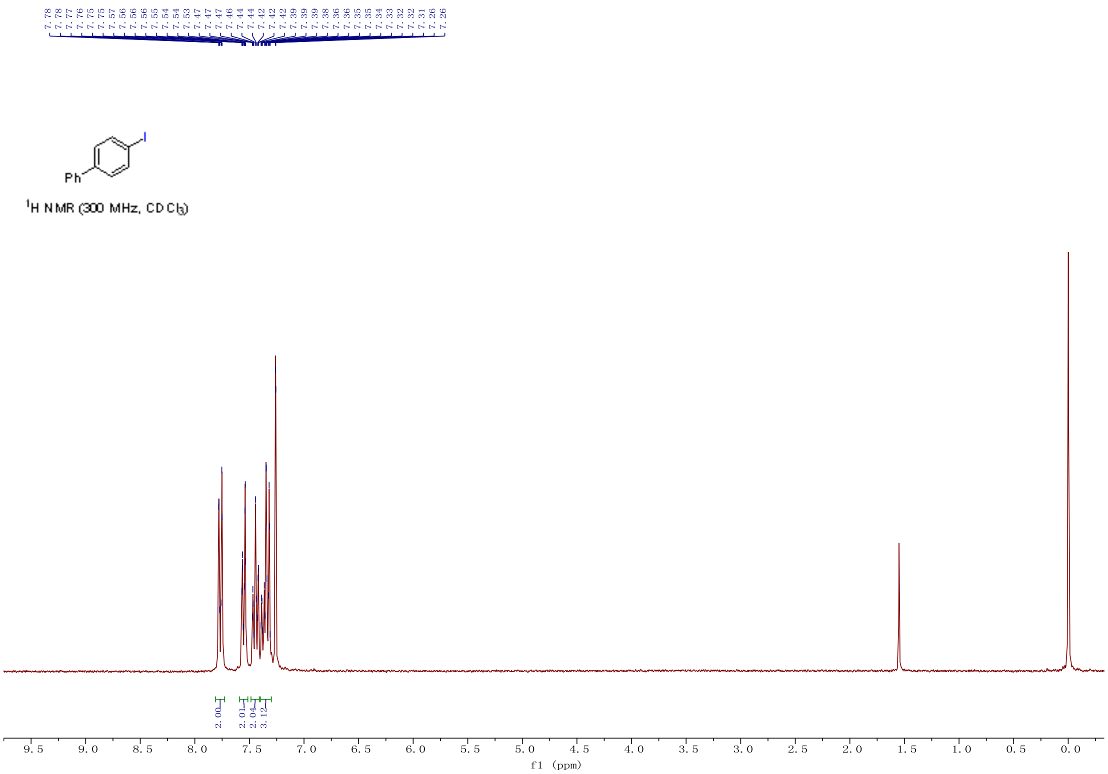


**Supplementary Figure 91**. NMR Spectra of 4-iodo-1,1'-biphenyl.

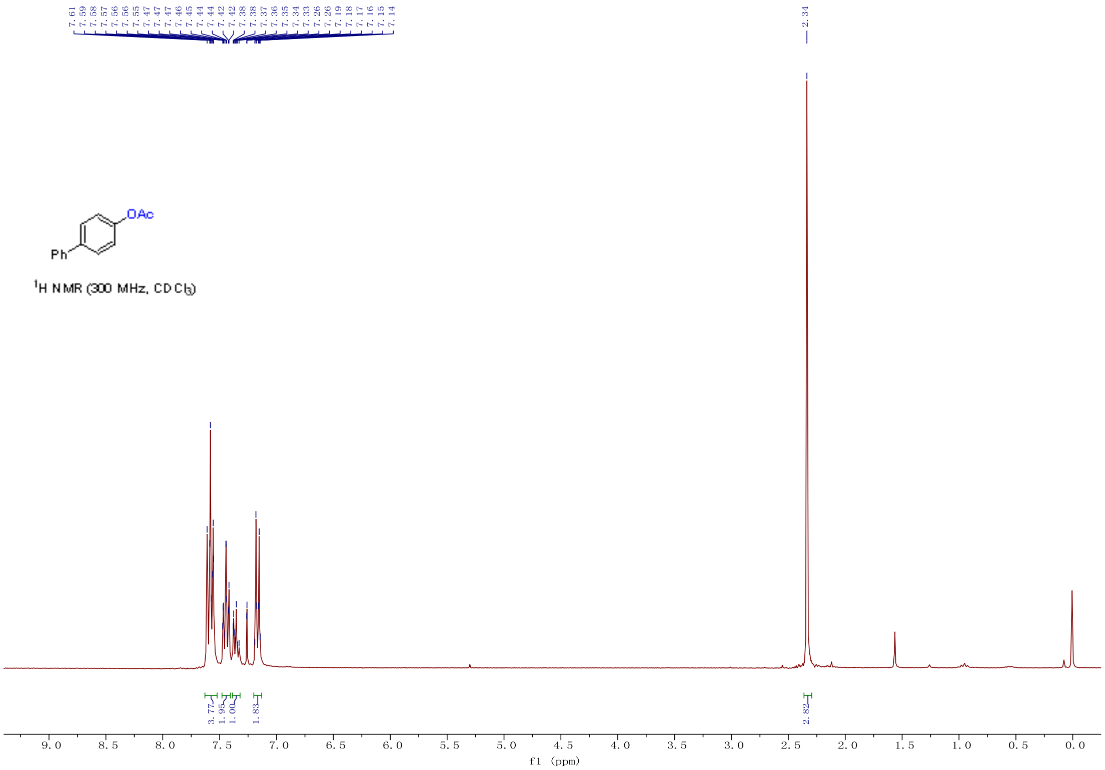

**Supplementary Figure 92**. NMR Spectra of [1,1'-biphenyl]-4-yl acetate.

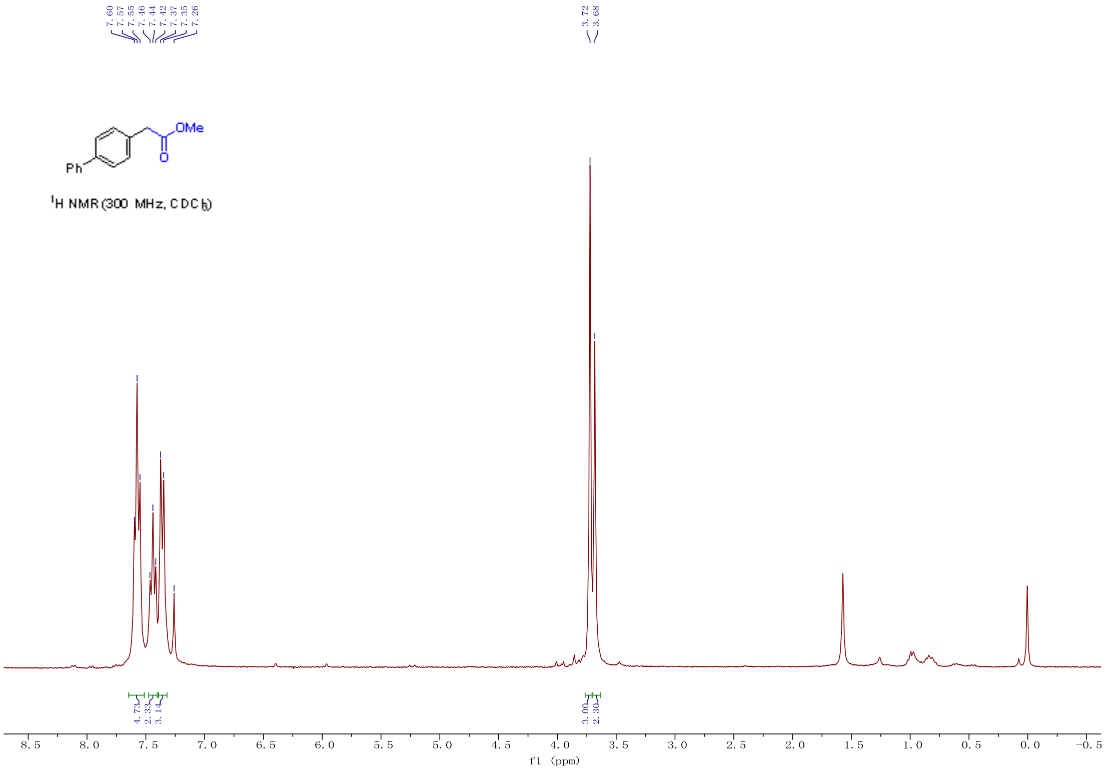

**Supplementary Figure 93**. NMR Spectra of methyl 2-([1,1'-biphenyl]-4-yl)acetate.

## Supplementary References


1. Boebel, T. A. & Hartwig, J. F. Iridium-catalyzed preparation of silylboranes by silane borylation and their use in the catalytic borylation of arenes. *Organometallics* **27**, 6013-6019 (2008).
2. Niwa, T., Ochiai, H., Watanabe, Y. & Hosoya, T. Ni/Cu-catalyzed defluoroborylation of fluoroarenes for diverse C-F bond functionalizations. *J. Am. Chem. Soc.* **137**, 14313-14318 (2015).
3. Tosatti, P. & Pfaltz, A. Iridium-catalyzed asymmetric hydrogenation of benzo[*b*]thiophene 1,1-dioxides. *Angew. Chem., Int. Ed.* **56**, 4579-4582 (2017).
4. Chen, X., Zheng, G., Li, Y., Song, G. & Li, X. Rhodium-catalyzed site-selective coupling of indoles with diazo esters: C4-alkylation versus C2-annulation. *Org. Lett.* **19**, 6184-6187 (2017).
5. Chang, J. W. W., Chee, S., Mak, S., Buranaprasertsuk, P., Chavasiri, W. & Chan, P. W. H. Copper-catalyzed Ullmann coupling under ligand- and additive- free conditions. Part 1: O-Arylation of phenols with aryl halides. *Tetrahedron Lett.* **49**, 2018-2022 (2008).
6. Ratnikov, M. O. & Doyle, M. P. Mechanistic investigation of oxidative mannich reaction with *tert*-butyl hydroperoxide. The Role of transition metal salt. *J. Am. Chem. Soc.* **135**, 1549-1557 (2013).
7. Cheng, Y., Dong, W., Wang, L., Parthasarathy, K. & Bolm, C. Iron-catalyzed hetero-cross-dehydrogenative coupling reactions of sulfoximines with diarylmethanes: A new route to *N*-alkylated sulfoximines. *Org. Lett.* **16**, 2000-2002 (2014).
8. Pinet, S., Liautard, V., Debiais,M. & Pucheault, M. Radical metal-free borylation of aryl iodides. *Synthesis* **49**, 4759-4768 (2017).
9. Kumar, M. R., Park, K. & Lee, S. Synthesis of amido-*N*-imidazolium salts and their applications as ligands in Suzuki–Miyaura reactions: Coupling of hetero- aromatic halides and the synthesis of milrinone and irbesartan. *Adv. Synth. Catal.* **352**, 3255-3266 (2010).
10. Dobele, M., Vanderheiden, S., Jung, N. & Brase, S. Synthesis of aryl fluorides on a solid support and in solution by utilizing a fluorinated solvent. *Angew. Chem., Int. Ed.* **49**, 5986-5988 (2010).
11. Guo, P., Joo, J. M., Rakshit, S. & Sames, D. C–H arylation of pyridines: high regioselectivity as a consequence of the electronic character of C–H bonds and heteroarene ring. *J. Am. Chem. Soc.* **133**, 16338-16341 (2011).
12. Kim, D. W., Jeong, H.-J., Lim, S. T. & Sohn, M.-H. Tetrabutylammonium tetra(*tert*-butyl alcohol)-coordinated fluoride as a facile fluoride source. *Angew. Chem., Int. Ed.* **47**, 8404-8406 (2008).
13. Blessley, G., Holden, P., Walker, M. & Brown, J. M., Gouverneur, V. Palladium-catalyzed substitution and cross-coupling of benzylic fluorides. *Org. Lett.* **14**, 2754-2757 (2012).
14. Xia, J. B., Chen, Z. & Chen, C. Visible light-promoted metal-free C-H activation: Diarylketone-catalyzed selective benzylic mono- and difluorination. *J. Am. Chem. Soc*. 17494-17500 (2013).
15. Colas, K., Martín - Montero,R. & Mendoza, A. Intermolecular pummerer coupling with carbon nucleophiles in non-electrophilic media. *Angew. Chem., Int. Ed.* **56**, 16042-16046 (2017).

16. Fuchibe, K., Mitomi, K., Suzuki, R. & Akiyama, T. C-C coupling reactions of superstrong $CF_3$ groups with C($sp^2$)–H bonds: reactivity and synthetic utility of zero-valent niobium catalyst. *Chem. Asian J.* **3**, 261-271 (2008).
17. Meanwell, M., Nodwell, M. B., Martin, R. E. & Britton, R. A convenient late-stage fluorination of pyridylic C-H bonds with *N*-fluorobenzenesulfonimide. *Angew. Chem., Int. Ed.* **55**, 13244-13248 (2016).
18. Kirihara, M., Takuwa, T., Kambayashi, T., Momose, T. & Takeuchi, Y. Novel fluorination of small-ring tertiary cycloalkanols: Reaction of diethylaminosulfur trifluoride with tertiary cyclobutanols. *J. Chem. Research (S)*, 652-653 (1998).
19. Prakash, G. K. S., Chacko, S., Vaghoo, H., Shao, N., Gurung, L., Mathew, T. & Olah, G. A. Efficient nucleophilic fluoromethylation and subsequent transformation of alkyl and benzyl halides using fluorobis(phenylsulfonyl)methane. *Org. Lett.* **11**, 1127-1130 (2009).
20. Zarate, C., Nakajima, M. & Martin, R. A Mild and ligand-free Ni-catalyzed silylation via C-OMe cleavage. *J. Am. Chem. Soc.* **139**, 1191-1197 (2017).
21. Guo, H., Chen, X., Zhao, C. & He, W. Suzuki-type cross coupling between aryl halides and silylboranes for the syntheses of aryl silanes. *Chem. Commun.* **51**, 17410-17412 (2015).
22. Wiensch, E. M., Todd, D. P. & Montgomery, J. Silyloxyarenes as versatile coupling substrates enabled by nickel-catalyzed C–O bond cleavage. *ACS Catal.* **7**, 5568-5571 (2017).
23. Pu, X., Hu, J., Zhao, Y. & Shi, Z. Nickel-catalyzed decarbonylative borylation and silylation of esters. *ACS Catal* **6**, 6692-6698 (2016).
24. Hilt, G. & Janikowski, J. Regiocontrolled cobalt-catalyzed Diels−Alder reactions of silicon-functionalized, terminal, and internal alkynes. *Org. Lett.* **11**, 773-776 (2009).
25. Yamanoi, Y. & Nishihara, H. Direct and selective arylation of tertiary silanes with rhodium catalyst. *J. Org. Chem.* **73**, 6671-6678 (2008).
26. Bai, L. & Wang, J.-X. Reusable, Polymer-supported, palladium-catalyzed, atom- efficient coupling reaction of aryl halides with sodium tetraphenylborate in water by focused microwave irradiation. *Adv. Synth. Catal.* **350**, 315-320 (2008).
27. Felpin, F.-X. & Fouquet, E. Efficient and practical cross-coupling of arenediazonium tetrafluoroborate salts with boronic acids catalyzed by palladium(0)/barium carbonate. *Adv. Synth. Catal.* **350**, 863-868 (2008).
28. Pallavicini, M., Budriesi, R., Fumagalli, L., Ioan, P., Chiarini, A., Bolchi, C., Ugenti, M. P., Colleoni, S., Gobbi, M. & Valoti, E. WB4101-related compounds: new, subtype-selective $\alpha_1$-adrenorecept or antagonists (or inverse agonists?). *J. Med. Chem.* **49**, 7140-7149(2006).
29. Wang, J., Burdzinski, G., Kubicki, J. & Platz, M. S. Ultrafast UV−Vis and IR studies of *p*-biphenylyl acetyl and carbomethoxy carbenes. *J. Am. Chem. Soc.* **130**, 11195-11209 (2008).
30. Dobereiner, G. E., Yuan, J., Schrock, R. R., Goldman, A. S. & Hackenberg, J. D. Catalytic synthesis of *n*-alkyl arenes through alkyl group cross-metathesis. *J. Am. Chem. Soc.* **135** (2013).
31. Ito, H., Horita, Y. & Yamamoto, E. Potassium *tert*-butoxide-mediated regioselective silaboration of aromatic alkenes. *Chem. Commun*. **48**, 8006-8008 (2012).
32. Phillips, W. D. & Miller, H. C., Muetterties, E. L. $B^{11}$ magnetic resonance study of boron compounds. *J. Am. Chem. Soc.* **81**, 4496-4500 (1959).
33. Nöth, H. & Vahrenkamp, H., Kernresonanzuntersuchungen an bor-verbindungen, I. $^{11}B$ - kernresonanzspektren von boranen mit substituenten aus der ersten achterperiode des periodensystems. *Chem. Ber.* **99** , 1049-1067 (1966).
